# Enhancement of the Antileukemic Action of the Inhibitors of DNA and Histone Methylation: 5-Aza-2′-Deoxycytidine and 3-Deazaneplanocin-A by Vitamin C

**DOI:** 10.3390/epigenomes5020007

**Published:** 2021-03-24

**Authors:** Richard L. Momparler, Sylvie Côté, Louise F. Momparler

**Affiliations:** 1 Département de Pharmacologie-Physiologie, Université de Montréal, Montréal, QC H3C 3J7, Canada; 2 Centre de Recherche, CHU Sainte-Justine, Montréal, QC H3T 1C5, Canada; sylvie_cote@bell.net (S.C.); louise.momparler@gmail.com (L.F.M.)

**Keywords:** epigenetics, leukemia, chemotherapy, DNA methylation, histone methylation, 5-aza-2′-deoxycytine, 3-deazaneplanocin-A, vitamin C, EZH2

## Abstract

Epigenetic gene silencing by DNA methylation and histone methylation by EZH2 play an important role in the development of acute myeloid leukemia (AML). EZH2 catalyzes the trimethylation of histone H3-lysine 27-trimethylated (H3K27me3). These epigenetic alterations silence the expression of the genes that suppress leukemogenesis. Reversal of this gene silencing by 5-aza-2′-deoxycytidine (5-Aza-CdR), an inhibitor of DNA methylation, and by 3-deazaneplanocin-A (DZNep), an inhibitor of EZH2, results in synergistic gene reactivation and antileukemic interaction. The objective of this study is to determine if the addition of another epigenetic agent could further enhance the antileukemic action of these inhibitors of DNA and histone methylation. Vitamin C (Vit C) is reported to enhance the antineoplastic action of 5-Aza-CdR on AML cells. The mechanism responsible for this action of Vit C is due to its function as a cofactor of alpha-ketoglutarate-dependent dioxygenases (α-KGDD). The enhancement by Vit C of the catalytic activity of α-KGDD of the ten eleven translocation (TET) pathway, as well as of the Jumonji C histone demethylases (JHDMs), is shown to result in demethylation of DNA and histones, leading to reactivation of tumor suppressor genes and an antineoplastic effect. This action of Vit C has the potential to complement the antileukemic action of 5-Aza-CdR and DZNep. We observe that Vit C remarkably increases the antineoplastic activity of 5-Aza-CdR and DZNep against myeloid leukemic cells. An important step to bring this novel epigenetic therapy to clinical trial in patients with AML is the determination of its optimal dose schedule.

## 1. Introduction

Epigenetic alterations play a key role in the development of acute myeloid leukemia (AML) and consist primarily of gene silencing by DNA and histone methylation [1,2]. The role of these epigenetic changes is to silence the expression of genes that suppress malignancy. Embryonic stem cells (ESCs) use epigenetic mechanisms to program the genome, where genes of specific differentiation phenotypes are activated by several mechanisms, including demethylation of DNA and histones. Quite the opposite, the genes that program other differentiation phenotypes are silenced primarily by DNA and histone methylation.

The importance of DNA methylation in the development of AML is shown by the identification of more than 5000 additional 5-methycytosine residues in the genome of leukemic cells as compared to normal white blood cells [3]. These epigenetic markers are very good targets for chemotherapeutic intervention. The treatment of AML patients with the inhibitor of DNA methylation 5-aza-2′-deoxycytidine (5-Aza-CdR, decitabine) results in DNA demethylation, tumor suppressor gene (TSG) reactivation, and induction of complete remissions [4,5]. However, the duration of remission in these AML patients is limited.

Although 5-Aza-CdR is effective against AML cells by its action on the demethylation of DNA, its effectiveness is limited if a second epigenetic gene-silencing marker is present, such as histone H3-lysine 27-trimethylated (H3K27me3) [6]. Elevated levels of H3K27me3 and EZH2 often are present in many types of cancer and contribute to the development of malignancy [2]. Preclinical studies have shown that the treatment of AML cells with the inhibitor of EZH2, 3-deazaneplanocin A (DZNep), induces differentiation and inhibits cellular proliferation [7]. Additionally, the survival time of mice with AML is prolonged by DZNep therapy [7]. This caveat can be overcome by the use of a combination of 5-Aza-CdR and DZNep. When these two epigenetic agents are combined they exhibit a synergistic antileukemic effect [8]. This novel epigenetic therapy using 5-Aza-CdR in combination with DZNep merits clinical investigation in patients with advanced AML.

The objective of this report is to investigate if vitamin C (Vit C) can enhance the antileukemic action of 5-Aza-CdR and DZNep. The molecular mechanism action of 5-Aza-CdR is due to its inhibition of DNA methyltransferase I (DNMT1), which results in a reduction in DNA methylation. The primary mechanism of action of DZNep is to inhibit EZH2, which results in the reduction of H3K27me3. This demethylation of DNA and H3K27me3 results in gene reactivation and inhibition of the proliferation of AML. Cells harbor enzymes that can demethylate both DNA and histones. The enzymatic action of the TET family can lead to DNA demethylation and gene reactivation [9]. The loss of function (LOF) of TET2 can lead to the development of AML. Likewise, the JHDM, UTX (KDM6B), can demethylate H3K9me2, also leading to gene reactivation [10]. LOF mutations of UTX also favor the development of AML. To summarize, the enzymatic catalytic function of TET2 and UTX can exhibit antileukemic activity similar to chemotherapy with 5-Aza-CdR and DZNep. Both the TET enzymes and the JHDM enzyme UTX are members of the α-ketoglutarate-dioxygenases [11]. Since Vit C is an important cofactor of these enzymatic reactions, it enhances their catalytic activity [12]. The importance of this interaction is shown by the enhancement of the antileukemic action of 5-Aza-CdR by Vit C [13]. Here, we investigate the potential of Vit C to enhance the antileukemic action of the combination of 5-Aza-CdR and DZNep.

## 2. Results

### Inhibition of the Colony Formation of HL-60 Leukemic Cells by 5-Aza-CdR, DZNep, and/or Vit C

**Figure 1:**
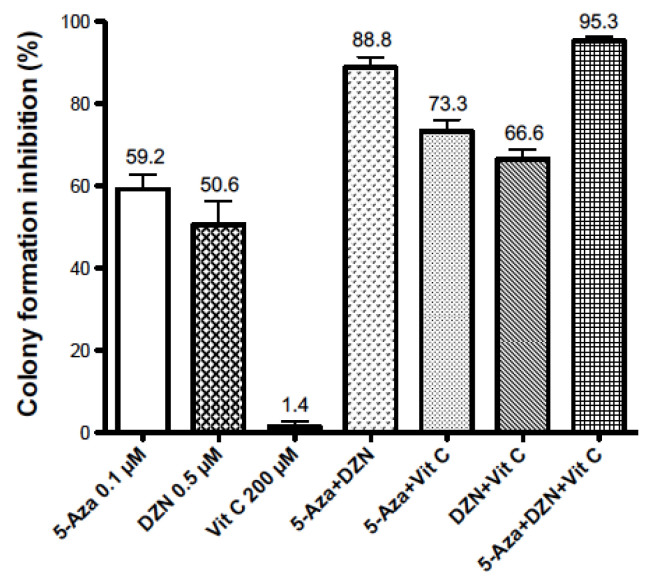
Enhancement by vitamin C (Vit C) of the antileukemic action of 5-Aza-CdR (5-Aza) and DZNep (DZN) on HL-60 myeloid leukemic cells. The leukemic cells were treated for 24 h with 0.1 µM of 5-Aza, 0.5 µM of DZN, and/or 200 µM of Vit C. Cell counts were made at the end of treatment and cells were placed in a soft agar medium to determine cell survival using a colony assay. The values shown are the inhibition of colony formation (mean ± S.E.; *n* = 3).

The results of the reduction in colony formation of HL-60 leukemic cells by a 24 h treatment of 5-Aza-CdR, DZNep, and/or Vit C are shown in Figure 1. The mean values ± SE of colony formation inhibition were: 59.2 ± 3.6% for 0.1 µM of 5-Aza-CdR (5-Aza); 50.6 ± 5.7% for 0.5 µM of DZNep (DZN); 1.4 ± 1.4% for 200 µM of Vit C. Colony formation inhibition (mean ± SE) was significantly greater when these agents were used in combination: 88.8 ± 2.5% for 5-Aza-CdR + DZNep; 73.3 ± 2.9% for 5-Aza-CdR + Vit C; 66.6 ± 2.4% for DZNep + Vit C; 95.3 ± 1.0% for 5-Aza-CdR +DZN + Vit C. The method of Valeriote and Lin was used to evaluate the drug interactions [14]. The interactions between Vit C with 5-Aza-CdR, Vit C with DZNep, and DZNep with 5-Aza-CdR were synergistic.

Statistical analysis: 5-Aza + DZN > 5-Aza or DZN, *p* < 0.01; 5-Aza + Vit C > 5-Aza, *p* < 0.05; 5-Aza + Vit C > Vit C, *p* < 0.01; DZN + Vit C > DZN, *p* < 0.05; DZN + Vit C > Vit C, *p* < 0.01; 5-Aza + DZN + Vit C > 5-Aza + Vit C, *p* < 0.01; 5-Aza + DZN + Vit C > 5-Aza + DZN (non-significant).

## 3. Discussion

One of the major characteristics of AML is the block in differentiation due to the epigenetic gene silencing by DNA methylation and/or histone methylation [1]. The reversal of gene silencing by DNA methylation by 5-Aza-CdR can lead to the induction of remissions in AML patients [5]. However, most AML patients treated with 5-Aza-CdR relapse, indicating the need to improve the efficacy of this epigenetic therapy by using this analogue in combination with another epigenetic agent. An interesting agent to use for this purpose is DZNep, which reduces the level of H3K27me3 by its inhibition of EZH2, leading to gene activation and inhibition of the growth of AML cells [7]. The addition of DZNep to 5-Aza-CdR therapy results in a synergistic antineoplastic action against AML cells [8] and a synergistic reactivation of many genes that suppress leukemogenesis [15].

5-Aza-CdR can induce complete remission in patients with AML, but the duration of remission is limited [5]. Comprehensive analysis of the epigenetics of AML cells may offer some insight into how to increase the effectiveness of 5-Aza-CdR against AML. 5-Aza-CdR may be very effective for targeting AML cells that have only one key epigenetic gene-silencing marker, such as DNA methylation. However, if the target AML cells contain a second gene-silencing marker, such as H3K27me3, some cells may escape the therapeutic action of 5-Aza-CdR [6]. When AML cells contain two gene-silencing markers, such as DNA methylation and H3K27me3, a “double lock” mechanism [6], a combination of epigenetic agents, has to be used to overcome this barrier. DZNep is an ideal agent to use in this case. The positive synergistic antineoplastic interaction of 5-Aza-CdR in combination with DZNep against AML cells, as shown in Figure 1, fully supports this approach.

Curative therapy of AML may require the eradication of >10^9^ leukemic stem cells (LSCs), a very difficult task. Will the addition of another agent to the combination of 5-Aza-CdR and DZNep increase its chemotherapeutic potential? An interesting agent to use for this purpose is Vit C). Supporting this approach is the report that Vit C enhances the antileukemic action of 5-Aza-CdR on AML cells [13]. The mechanism of the antineoplastic action of Vit C is very interesting. Vit C is an important cofactor for alpha-ketoglutarate-dependent dioxygenases (α-KGDD). This family of enzymes has the capacity to increase the catalytic activity of the TET family of enzymes and the JHDMs (UTX). They have the capacity to demethylate DNA and histones (H3K27me3) leading to reactivation of the expression of genes that suppress leukemogenesis [10]. High concentrations of Vit C (0.25–1 mM) were reported to inhibit proliferation and induce apoptosis in AML cell lines [16]. This action of Vit C is due to its oxidation of glutathione and subsequent accumulation hydrogen peroxide. The antileukemic action of Vit C alone at 200 µM was not significant but became apparent by its remarkable enhancement of the antileukemic action of 5-Aza-CdR and/or DZNep (Figure 1). This observation supports the notion that at low concentrations Vit C acts as a co-factor for the enzymes that demethylate DNA and histones.

We confirmed that Vit C enhances the antineoplastic action of 5-Aza-CdR on AML cells using a colony assay (Figure 1). This observation is in accordance with the clinical report that Vit C prolongs the survival time of AML patients treated with a chemotherapeutic regimen containing 5-Aza-CdR [17]. Additionally, Vit C also enhanced the antileukemic action of DZNep and the combination of 5-Aza-CdR and DZNep. The combination of 5-Aza-CdR, DZNep, and Vit C exhibited a remarkable antileukemic effect, reducing the colony formation of AML cells greater than 90% (Figure 1). To conclude, our data support the use of Vit C to enhance the effectiveness of epigenetic therapy using 5-Aza-CdR and DZNep, and merits clinical investigation in patients with advanced AML.

However, the optimal dose schedule of the combination of these epigenetic agents needs to be determined [18,19]. One of the standard dose schedules for the treatment of AML patients with 5-Aza-CdR is 15 mg/m^2^ intravenously over 1 h daily, five days a week for two consecutive weeks [20]. This schedule of administration of 5-Aza-CdR is not optimal, since this nucleoside analogue is an S phase-specific drug [21]. The plasma half-life of 5-Aza-CdR is approximately 20 min, which means there is a 22 h interval between iv injections where its concentration is below the level required for antileukemic activity. The significance of this fact is that many AML cells pass through the S phase window without exposure to adequate levels of 5-Aza-CdR, thereby escaping its chemotherapeutic action. Supporting this hypothesis is our observation that when the intervals between treatments of AML cells with the S phase-specific agent cytosine arabinoside were increased, the antileukemic action of this analogue decreased [22]. One approach to overcome this impediment is to administer 5-Aza-CdR by continuous iv infusion. Bone marrow toxicity is a major side effect of 5-Aza-CdR and the selection of AML patients with an adequate hematological status is recommended for clinical investigation. We administered 5-Aza-CdR by iv infusion for up to 60 h to AML patients at a rate of drug infusion that gave a plasma level in the range of 1 µM, and we observed interesting antileukemic effects without unacceptable toxicity [23]. However, the responses due to the DNA demethylation action of 5-Aza-CdR were suboptimal, perhaps due to the presence of a second epigenetic gene-silencing marker, such as H3K27me3, which permitted some leukemic stem cells to escape the chemotherapeutic action of this analogue. This analysis suggests that a second epigenetic agent that reduces the level of H3K27me3 should be used in combination with 5-Aza-CdR.

This report and our previous publication suggest that DZNep is a very good candidate to reduce H3K27me3 by its inhibition of EZH2 [8]. Our study indicates that DZNep is a more potent antileukemic agent than the specific inhibitors of EZH2. Some of these EZH2 inhibitors are in clinical trial in patients with cancer [24]. However, DZNep is not available for clinical investigation and needs approval by the Federal Drug Administration (FDA) for clinical use. Enclosed in the Supplementary Materials is the Investigator’s Brochure 3-Deazaneplanocin-A (R.L. Momparler). This document contains a summary of the pharmacology of DZNep, its preclinical antineoplastic on many types of cancer, and its potential use to enhance the effectiveness of the immunotherapy of cancer. This document will be helpful to obtain the approval for an investigation of the application of a new drug for DZNep by the FDA.

Regarding the initial phase I study on AML patients, we recommend the use of 5-Aza-CdR in combination with DZNep as a 36 h IV infusion using doses that provide a plasma level of these analogues in the range of 0.1–1.0 µM. The duration of this IV infusion can be extended in a step-wise manner depending on the response and adverse effects. Concerning the clinical use of Vit C, we recommend that it be administered by oral administration every 8 h during the infusion of 5-Aza-CdR and DZNep at a dose that provides a peak plasma level of Vit C in the range of 100 µM [25]. This novel epigenetic therapy has immense potential for the treatment of AML and merits serious consideration for clinical investigation.

## 4. Materials and Methods

HL-60 human myeloid leukemia cells were obtained from ATCC, Manassas, VA, USA and maintained in RPMI-1640-HEPES medium (Thermo Fischer Scientific, Invitrogen, Waltham, MA, USA) with 10% fetal bovine serum (FBS). 5-Aza-CdR was provided by Dr. Alois Piskala, Czechoslovak Academy of Sciences (Prague, Czech Republic). DZNep was provided by Dr. Victor E. Marquez, Chemical Biology Laboratory NIH (Frederick, MD, USA). Vitamin C (A7506, l-ascorbic acid) was obtained from Sigma-Aldrich (Oakville, ON, Canada). 5-Aza-CdR, DZNep, and Vit C were dissolved in 50% phosphate-buffered saline pH 6.8 (Invitrogen), sterilized by filtration and stored at −20 °C.

HL-60 leukemic cells (50,000 or 100,000 cells/mL) were placed in 25 cm^2^ flasks (Sarstedt Inc., Saint-Leonard, QC, Canada) and the drugs added at the indicated concentration. Following a 24 h drug exposure, a cell count was performed using the Beckman Model Z Coulter Counter (Beckman Coulter Counter, Montreal, QC, Canada). Regarding colony assay, 100 cells were placed in 0.36% soft agar medium containing 20% FBS in RPMI 1640 medium. The number of colonies (>500 cells) was counted after 18–21 days of incubation. The cloning efficiency was in the range of 60–70%. Statistical analysis of the data was performed using Prism GraphPad version 7 and Tukey’s multiple comparison test.

## 5. Conclusions

Vit C markedly enhanced the antileukemic action of the inhibitor of DNA methylation, 5-Aza-CdR, and DZNep, the inhibitor of histone methylation by EZH2. This observation suggests that there exists an important cooperative interaction between DNA and histone methylation to silence gene expression. This interaction plays a key role in leukemogenesis and provides a solid rationale to use epigenetic agents that reverse gene silencing by DNA and histone methylation for the therapy of AML. The enzymes of the TET pathway and JHDMs also have the potential, by their demethylating action, to reverse gene silencing by DNA methylation and H3k72me3, leading to a loss of the self-renewal capacity of LSCs [26]. These natural intrinsic enzymatic mechanisms may also may have the potential to further enhance the effectiveness of the epigenetic therapy of AML using 5-Aza-CdR, DZNep, and Vit C.

## Data Availability

The data presented in this study are available on request from the corresponding author.

## Supplementary Materials

The following are available online at https://www.mdpi.com/2075-4655/5/2/7/s1, Investigator’s Brochure: 3-Deazaneplanocin-A by Richard L. Momparler.

## References

[1] Wouters B.J., Delwel R. (2016). Epigenetics and approaches to targeted epigenetic therapy in acute myeloid leukemia. Blood.

[2] Kim K.H., Roberts C.W.M. (2016). Targeting EZH2 in cancer. Nat. Med..

[3] The Cancer Genome Atlas Research Network (2013). Genomic and epigenomic landscapes of adult de novo acute myeloid leukemia. N. Eng. J. Med..

[4] Baylin S.B., Jones P.A. (2011). A decade of exploring the cancer epigenome—Biological and translational mplications. Nat. Rev. Cancer.

[5] Lübbert M., Rüter B.H., Claus R., Schmoor C., Schmid M., Germing U., Kuendgen A., Rethwisch V., Ganser A., Platzbecker U. (2012). A multicenter phase II trial of decitabine as first-line treatment for older patients with acute myeloid leukemia judged unfit for induction chemotherapy. Haematologica.

[6] Si J., Boumber Y.A., Shu J., Qin T., Ahmed S., He R., Jelinek J., Issa J.-P.J. (2010). Chromatin remodeling is required for gene reactivation after decitabine-mediated DNA hypomethylation. Cancer Res..

[7] Fiskus W., Wang Y., Sreekumar A., Buckley K.M., Shi H., Jillella A., Ustun C., Rao R., Fernandez P., Chen J. (2009). Combined epigenetic therapy with the histone methyltransferase EZH2 inhibitor 3-deazaneplanocin A and the histone deacetylase inhibitor panobinostat against human AML cells. Blood.

[8] Momparler R.L., Idaghdour Y., Marquez V.E., Momparler L.F. (2012). Synergistic antileukemic action of inhibitors of DNA methylation and histone methylation. Leuk. Res..

[9] Cimmino L., Dolgalev I., Wang Y., Yoshimi A., Martin G.H., Wang J., Ng V., Xia B., Witkowski M.T., Mitchell-Flack M. (2017). Restoration of TET2 function blocks aberrant self-renewal and leukemia progression. Cell.

[10] Lu C., Ward P.S., Kapoor G.S., Rohle D., Turcan S., Abdel-Wahab O., Edwards C.R., Khanin R., Figueroa M.E., Melnick A. (2012). IDH mutation impairs histone demethylation and results in a block to cell differentiation. Nature.

[11] Lee Chong T., Ahearn E.L., Cimmino L. (2019). Reprogramming the epigenome with vitamin C. Front. Cell Dev. Biol..

[12] Yin R., Mao S.Q., Zhao B., Chong Z., Yang Y., Zhao C., Zhang D., Huang H., Gao J., Li Z. (2013). Ascorbic acid enhances Tet-mediated 5-methylcytosine oxidation and promotes DNA demethylation in mammals. J. Am. Chem. Soc..

[13] Liu M., Ohtani H., Zhou W., Ørskov A.D., Charlet J., Zhang Y.W., Shen H., Baylin S.B., Liang G., Grønbæk K. (2016). Vitamin C increases viral mimicry induced by 5-aza-2’-deoxycytidine. Proc. Natl. Acad. Sci. USA.

[14] Valeriote F., Lin H.S. (1975). Synergistic interaction of anticancer agents: A cellular perspective. Cancer Chemother. Rep..

[15] Momparler R.L., Côté S., Momparler L.F., Idaghdour Y. (2017). Inhibition of DNA and histone methylation by 5-aza-2’-deoxycytidine (decitabine) and 3-deazaneplanocin-A on antineoplastic action and gene expression in myeloid leukemic cells. Front. Oncol..

[16] Park S., Han S.S., Park C.H., Hahm E.R., Lee S.J., Park H.K., Lee S.H., Kim W.S., Jung C.W., Park K. (2004). L-Ascorbic acid induces apoptosis in acute myeloid leukemia cells via hydrogen peroxide-mediated mechanisms. Int. J. Biochem. Cell Biol..

[17] Zhao H., Zhu H., Huang J., Zhu Y., Hong M., Zhu H., Zhang J., Li S., Yang L., Lian Y. (2018). The synergy of Vitamin C with decitabine activates TET2 in leukemic cells and significantly improves overall survival in elderly patients with acute myeloid leukemia. Leuk. Res..

[18] Lemaire M., Chabot G.G., Raynal N.J., Momparler L.F., Hurtubise A., Bernstein M.L., Momparler R.L. (2008). Importance of dose-schedule of 5-aza-2′-deoxycytidine for epigenetic therapy of cancer. BMC Cancer.

[19] Karahoca M., Momparler R.L. (2013). Pharmacokinetic and pharmacodynamic analysis of 5-aza-2′-deoxycytidine (decitabine) in the design of its dose-schedule for cancer therapy. Clin. Epigenet..

[20] Issa J.-P.J., Garcia-Manero G., Giles F.J., Mannari R., Thomas D., Faderl S., Bayar E., Lyons J., Rosenfeld C.S., Cortes J. (2004). Phase 1 study of low-dose prolonged exposure schedules of the hypomethylating agent 5-aza-2’-deoxycytidine (decitabine) in hematopoietic malignancies. Blood.

[21] Momparler R.L. (2005). Pharmacology of 5-Aza-2’-deoxycytidine (Decitabine). Semin. Hematol..

[22] Leclerc J.-M., Momparler R.L. (1984). Importance of the interval between exposures to cytosine arabinoside on its cytotoxic action on HL-60 myeloid leukemic cells. Cancer Treat. Rep..

[23] Momparler R.L., Rivard G.E., Gyger M. (1985). Clinical trial on 5-aza-2′-deoxycytidine in patients with acute leukemia. Pharmacol. Ther..

[24] Momparler R.L., Côté S., Momparler L.F., Marquez V.E. (2020). Comparison of the antineoplastic action of 3-deazaneplanocin-A and inhibitors that target the catalytic site of EZH2 histone methyltransferase. Cancer Rep. Rev..

[25] Gillberg L., Ørskov A.D., Nasif A., Ohtani H., Madaj Z., Hansen J.W., Rapin N., Mogensen J.B., Liu M., Dufva I.H. (2019). Oral vitamin C supplementation to patients with myeloid cancer on azacitidine treatment: Normalization of plasma vitamin C induces epigenetic changes. Clin. Epigenet..

[26] Momparler R.L., Côté S., Momparler L.F. (2020). Epigenetic modulation of self-renewal capacity of leukemic stem cells and implications for chemotherapy. Epigenomes.

