# Supplementary material for: Enhancement of the Antileukemic Action of the Inhibitors of DNA and Histone Methylation: 5-Aza-2′-Deoxycytidine and 3-Deazaneplanocin-A by Vitamin C
Source: Epigenomes. 2021 Mar 24;5(2):7. doi: 10.3390/epigenomes5020007 (PMC8594729; doi:10.3390/epigenomes5020007)
Supplement: Supplementary file 1 [file epigenomes-05-00007-s001.pdf]

# INVESTIGATOR’S BROCHURE

# 3-DEAZANEPLANOCIN A

Richard L. Momparler, Ph.D.

Department of Pharmacology-Physiology
University of Montreal

and

Service of Hematology-Oncology
University Hospital Centre Sainte-Justine

Montreal, Quebec H3T 1C5
Canada

16 November 2020

Page

TABLE OF CONTENTS 2

1. SUMMARY 5

2. INTRODUCTION 6

3. PHYSICAL, CHEMICAL AND PHARMACEUTICAL PROPERTIES 8

4. CHEMICAL SYNTHESIS OF 3-DEAZANEPLANOCIN A 9

5. MOLECULAR MECHANISM OF ACTIVITY 10

6. IN VITRO ANTICANCER ACTION 13

- 6.1 Leukemia-1 13
- 6.2 Leukemia-2 15
- 6.3 Leukemia-3 16
- 6.4 Leukemia-4 21
- 6.5 Lymphoma 25
- 6.6 T-cell Leukemia 26
- 6.7 Breast Cancer 27
- 6.8 Chondrosarcoma Cells 30
- 6.9 Colon Cancer-1 34
- 6.10 Colon Cancer-2 36
- 6.11 Colon Cancer-3 38
- 6.12 Gastric Cancer 41
- 6.13 Glioblastoma 42
- 6.14 Hepatocellular Carcinoma Cells 43
- 6.15 Lung Cancer-1 46
- 6.16 Lung Cancer-2 49
- 6.17 Lung Cancer-3 50
- 6.18 Mesothelioma 52
- 6.19 Multiple Myeloma-1 53
- 6.20 Multiple Myeloma-2 54
- 6.21 Neuroblastoma 56
- 6.22 Breast & Colon Tumors 58
- 6.23 Osteosarcoma 60
- 6.24 Ovarian Cancer 61
- 6.25 Prostate Cancer-1 62
- 6.26 Prostate Cancer-2 63
- 6.27 Rhabdomyosarcoma 65
- 6.28 Tongue Cancer 66

**7. SUMMARY TABLE IN VITRO ANTICANCER ACTIVITY** **69**

**8. IN VIVO ANTICANCER ACTIVITY** **70**

**8.1 Leukemia-1** **70**
**8.2 Leukemia-2** **71**
**8.3 Leukemia-3** **72**
**8.4 Leukemia-4** **73**
**8.5 Lung Cancer** **74**
**8.6 Lymphoma** **75**
**8.7 Glioblastoma** **76**
**8.8 Hepatocellular Carcinoma** **77**
**8.9 Mesothelioma** **78**
**8.10 Multiple Myeloma** **79**
**811 Neuroblastoma** **80**
**8.12 Prostate Cancer** **81**

**9. SUMMARY TABLE IN VIVO ANTICANCER ACTIVITY** **82**

**10. COMBINATION CHEMOTHERAPY** **83**
**10.1 Leukemia-1** **83**
**10.2 Leukemia-2** **85**
**10.3 Leukemia-3** **88**
**10.4 Lung Cancer-1** **90**
**10.5 Lung Cancer-2** **91**
**10.6 Prostate Cancer** **92**
**10.7 Ovarian Cancer** **93**

**11. BIOCHEMICAL PHARMACOLOGY: GENE EXPRESSION** **95**

**11.1 Leukemia** **95**
**11.2 Breast Cancer** **96**

**12. BIOCHEMICAL PHARMACOLOGY: PROTEIN EXPRESSION** **97**

**12.1 Leukemia** **97**
**12.2 Breast Cancer** **99**
**12.3 Breast & Bladder Cancer** **101**
**12.4 Ovarian Cancer** **102**

**13. ANALYLICAL QUANTITATION AND PHARMACOKINETICS: MICE** **103**

**13.1 Quantification of DZNep by HPLC-mass spectrometric detection.** **103**
**13.2 Chemical stability** **103**
**13.3 Linearity of detection; accuracy and precision** **103**

**13.4 Typical chromatogram of HPCL-MS/MS analysis 104**
**13.5 Example of pharmacokinetic data. 105**

**14. PHARMACOKINETICS AND METABOLISM: RATS 106**
**14.1 Abstract 106**
**14.2 Plasma conc. & Pharmacokinetics parameters after i.v. injection 106**
**14.3 Distribution in organs 109**
**14.4 Excretion in urine and feces 111**

**15. GENERAL TOXICITY: RATS 112**
**15.1 Body Weight 112**
**15.2 Blood Chemistry 113**
**15.3 Hematology 114**

**16. GENERAL TOXICITTY: MICE 115**
**16.1 Tissue injury and body weight loss in mice by DZNep treatment 115**
**16.2 Hematological analysis in mice after treatment with DZNep 116**

**17. GENERAL TOXICITY: MICE 117**
**17.1 Body weight changes in mice during DZNep treatment 117**

**18. GENERAL TOXICITY: MONKEYS 118**
**18.1 Experimental Protocol and Letter to Marquez from Bray 119**
**18.2 DZNep 0.3 mg/kg i.m. qd x 4: blood chemistry, liver function, hematology 121**
**18.3 DZNep 0.6 mg/kg i.m. qd x 4: blood chemistry, liver function, hematology 126**
**18.4 DZNep 1.2 mg/kg i.m. day 1; 0.6 mg/kg days 2,3,4 blood, liver, hematology 134**

**19. POTENTIAL OF DZNep FOR IMMUNOTHERAPY OF CANCER 139**
**19.1 Inhibition of EZH2 synergizes with immunotherapy of cancer 139**
**19.2 Inhibition of DNA methylation and EZH2 enhancement of immunotherapy 142**
**19.3. Inhibition of EZH2 enhances cancer-testis antigen expression 145**
**19.4. Epigenetic modulation of immunotherapy 149**

**20. COMPARISON OF ANTICANCER ACTIVITY OF DZNep WITH INHIBITORS OF EZH2 151**
**20. DISCUSSION 154**
**21. REFERENCES 157**
**22. ACKNOWLEDGEMENTS 160**

## 1. SUMMARY

3-Deazaneplanocin A (DZNep) is a carboscyclic nucleoside analogue of adenosine which was originally investigated for its antiviral activity against the EBOLA virus. The molecular mechanism of action is related to its inhibition of adenosyl homocysteine hydrolase (AHH), one of the key enzymes involved in the biosynthesis of S-adenosyl-methionine (SAM). The inhibition of AHH by DZNep results in the accumulation of the metabolite, adenosyl homocysteine (AH), which in turn inhibits specific SAM-dependent methylations. SAM acts as the methyl donor in enzymatic methylation reactions. AH acts as a competitive inhibitor of these reactions. One of the preferential targets for inhibition by DZNep is EZH2, the histone methyltransferase that methylates lysine 27-histone H3 (H3K27me). EZH2 is a subunit of the polycomb repressive complex-2 (PCR2). The methylation of H3K27 to its trimethylated form (H3K27me3) in the promoter region of target genes results in gene silencing.

The In vitro treatment of cancer cells with DZNep at concentrations 1 to 5 μM reduces the level of H3K27me3 and results in the activation of many genes that suppress malignancy. DZNep at concentrations of 1 to 5 μM inhibit the in vitro of leukemic and tumor cells. Significant antineoplastic activity against leukemia and tumors in mice was observed at doses of 1 to 5 mg/kg of DZNep.

The pharmacokinetics of DZNep in mice following i.v. and i.p. injections was described by a two-compartment model. DZNep exhibited a clearance of 0.09L/min/kg and a terminal half-life of 54 min. The exposures, as measured by the area under the plasma concentration-time curve, were 21.6 μg-min/mL for i.v. for i.v. and 37.0 μg-min/mL for i.p. injections, resulting in a bioavailability of 1.7.

The toxicity of DZNep was investigated in rats following an i.v. injection at doses of 10, 15 and 20 mg/kg. At these dose levels there were no significant changes in organ weights except for the kidney, which showed a 21.5% decrease in weight at the dose of 20 mg/kg. Blood chemistry analysis showed that at these dose levels there were no significant changes, except at the dose of 20 mg/kg, which exhibited a significant increase in the BUN and creatinine levels. The i.v. injection of DZNep to rats at a dose of 10 mg/kg body weight did not cause any obvious signs of toxicity or mortality. Therefore, the NOAEL (no observable adverse effect level) of DZNep determined was 10 mg/kg body weight, the highest single safe dose tested in rats with intravenous bolus injection. In a pilot study DZNep at doses of 1.2 or 2 mg/kg daily for 4 days by intramuscular injection caused transient elevation of alanine aminotransferase and aspartate aminotransferase, suggestive of minor hepatotoxicity. These values returned to normal after cessation of therapy.

## 2. INTRODUCTION

Neplanocin A was isolated from the culture filtrate of the soil organism, Ampullariella regularis, by Yaginuma et al. in 1981 and was found effective in prolonging the life-span of mice infected with L1210 leukemia. In a screen to evaluate its anticancer activity, Neplanocin A was found effective in prolonging the life-span of mice infected with L1210 leukemia (Borchardt & Keller, 1986). The free nucleoside of Neplanocin A was shown to exhibit a potent inhibition of adenosyl homocysteine hydrolase (AHH). In cells Neplanocin A was readily phosphorylated to the triphosphate level which led to the formation of toxic metabolites, which at high levels were associated with cytotoxicity.

In order to avoid the toxic effects characteristic of Neplanocin A, the synthetic adenosine analogue, 3-deazaneplanocin A (DZNep), was synthesized (Glazer et al., 1986a). DZNep was not a substrate for adenosine kinase and a very potent inhibitor of AHH (Glazer et al., 1986b). Inhibition of AHH by DZNep results in the accumulation of adenosyl homocysteine, which in turn inhibits specific S-adenosyl-L-methionine (SAM)-dependent methylations. DZNep showed excellent antiviral activity in cell culture against several viruses and was effective in vivo against vaccinia virus. (Tseng et al., 1989). The antiviral action of DZNep resulting from the inhibition of AHH correlated with the inhibition of methylation of the 5'-cap of viral messenger RNA.

In 2002, Mike Bray of the United States Army Medical Research Institute of Infectious Diseases, discovered that a single dose of DZNep administered in early infection prevented illness and death in Ebola virus-infected mice. The protective effect in infected mice was due to a massive increase in production of interferon-α in infected mice, which did not occur in uninfected animals. (Bray et al.,, 2002).

In 2007, scientists at The Genome Institute of Singapore screened a National Cancer Institute (NCI) Library of 4000 compounds and identified DZNep as a compound capable of inducing cell death in breast, colorectal, and other cancer cell lines, while being completely innocuous to normal cells. These investigators found that DZNep actually worked by selectively inhibiting the methylation of histone H3 Lys 27 (H3K27) and depleting cellular levels of PRC2 components EZH2, SUZ12 and EED (Tan et al., 2007). DZNep is also able to induce the expression of the ubiquitin (Ub) ligase, PRAJA1, which targets PRC2 subunits for proteasomal degradation. (Zoabi et al., 2011). The reduction in the level of methylated H3K27, a gene-silencing marker, by DZNep results in the upregulation of many genes that program differentiation. Therefore, DZNep appears to be a unique chromatin remodeling compound countering the activity of EZH2 which orchestrates transcriptional silencing of gene networks involved in cell cycle regulation, senescence, cell differentiation and cancer. Compounds like DZNep which are able to interfere with chromatin remodeling are categorized as epigenetic drugs because they only affect patterns of altered gene expression that are independent of the primary DNA sequence. Epigenetic alterations play an important role in the development of malignancy (Baylin & Jones, 2011).

In conclusion, these observations point out that DZNep merits clinical investigation in patients with cancer. In support of this premise are the reports that DZNep exhibits both in vitro and in vivo antineoplastic action against many different types of cancer (Momparler & Côté 2014). The major target of DZNep, EZH2, exhibits overexpression in different malignancies (Kim

& Roberts, 2016). In addition, DZNep shows activity against cancer stem cells (Benoit et al., 2013;Crea et al., 2011; Hibino et al., 2014; Li et al., 2013) and shows very little toxicity in animal models of cancer (Wee et al., 2014).

The development of drug resistance is one of the major problems of single agent therapy of cancer. In this regard, it is interesting to note the synergistic activity against cancer when DZNep is used in combination with other epigenetic agents, such as an inhibitor of histone deacetylase (Fiskus et al., 2009) or an inhibitor of DNA methylation (Momparler et al., 2012). Of special note is the remarkable synergistic activation of genes by DZNep in combination with 5-aza-2'-deoxycytidine (decitabine), which correlates with its synergistic antileukemic action (Momparler et al., 2017). This combination of epigenetic agents merits clinical investigation with high priority in patients with leukemia.

# 3. PHYSICAL, CHEMICAL AND PHARMACEUTICAL PROPERTIES

Physical and chemical Data

GENERIC NAME: DZNep

CHEMICAL NAME: (-)-1-[(1*R*,4*R*,5*S*)-3-(Hydroxymethyl)-4,5-dihydroxy-2-cyclopenten-1-yl]-4-aminoimidazo[4,5-c]pyridine Hydrochloride

OTHER NAMES: 3-DEAZANEPLANOCIN A•HCL (NSC 617989)

CAS NUMBER: 120964-45-6 (hydrochloride); 102052-95-9 (free base)

IUPAC NAME: (1S,2R,5R)-5-(4-aminoimidazo[4,5-c]pyridin-1-yl)-3-(hydroxymethyl)cyclopent-3-ene-1,2-diol

PubChem CID: 73087

CHEMICAL STRUCTURE (free base):

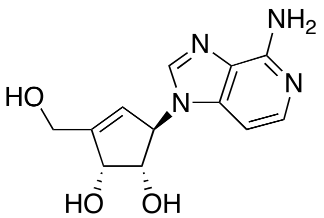


Figure 1: chemical structure of DZNep

MOLECULAR WEIGHT: 298.73 (hydrochloride); 262.30 (free base)

MOLECULAR FORMULA: $C_{12}H_{14}N_4O_3$•HCl (hydrochloride); $C_{12}H_{14}N_4O_3$ (free base)

TECHNICAL DATA (hydrochloride);

White solid, Mp 168-169 °C

Solubility: Soluble to 10 mM in water

Storage: Store at -20 °C

# 4. CHEMICAL SYNTHESIS OF 3-DEAZANEPLANOCIN A

Tseng CK, Marquez VE et al., Synthesis of 3-deazaneplanocin A, a powerful inhibitor of S-adenosylhomocysteine hydrolase with potent and selective in vitro and in vivo antiviral activities. J Med Chem. 1989;32:1442-1446.

Choi WJ, Jeong LS et al., Preparative and Stereoselective Synthesis of the Versatile Intermediate for Carbocyclic Nucleosides: Effects of the Bulky Protecting Groups to Enforce Facial Selectivity J. Org. Chem 2004;69:2634-2636.

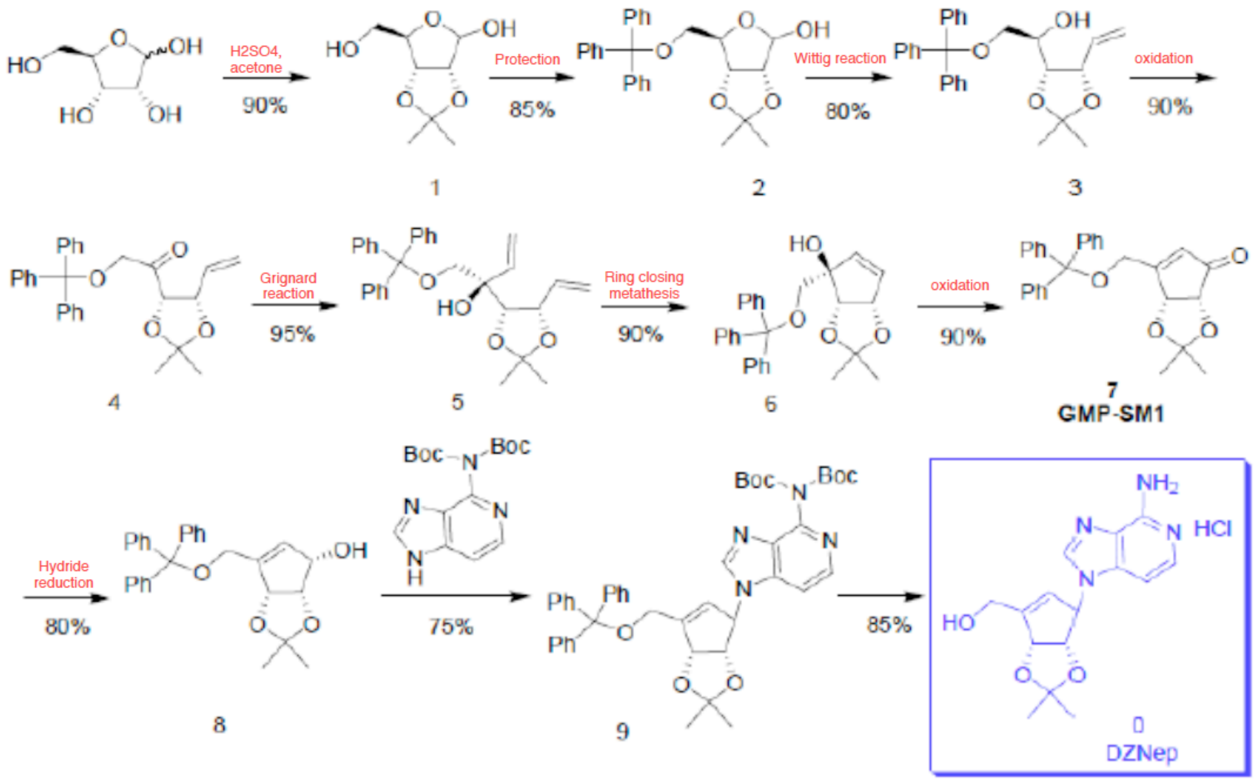


The original method for the chemical synthesis of DZNep by Tseng et al., (1989) was modified by Choi et al., (2004) so as to increase the yield of the synthesis.

**5. MOLECULAR MECHANISM 0F ACTION**

Glazer RI,et al. 3-Deazaneplanocin: a new and potent inhibitor of S-adenosylhomocysteine hydrolase and its effects on human promyelocytic leukemia cell line HL-60. Biochem Biophys Res Commun 1986;135:688-694.

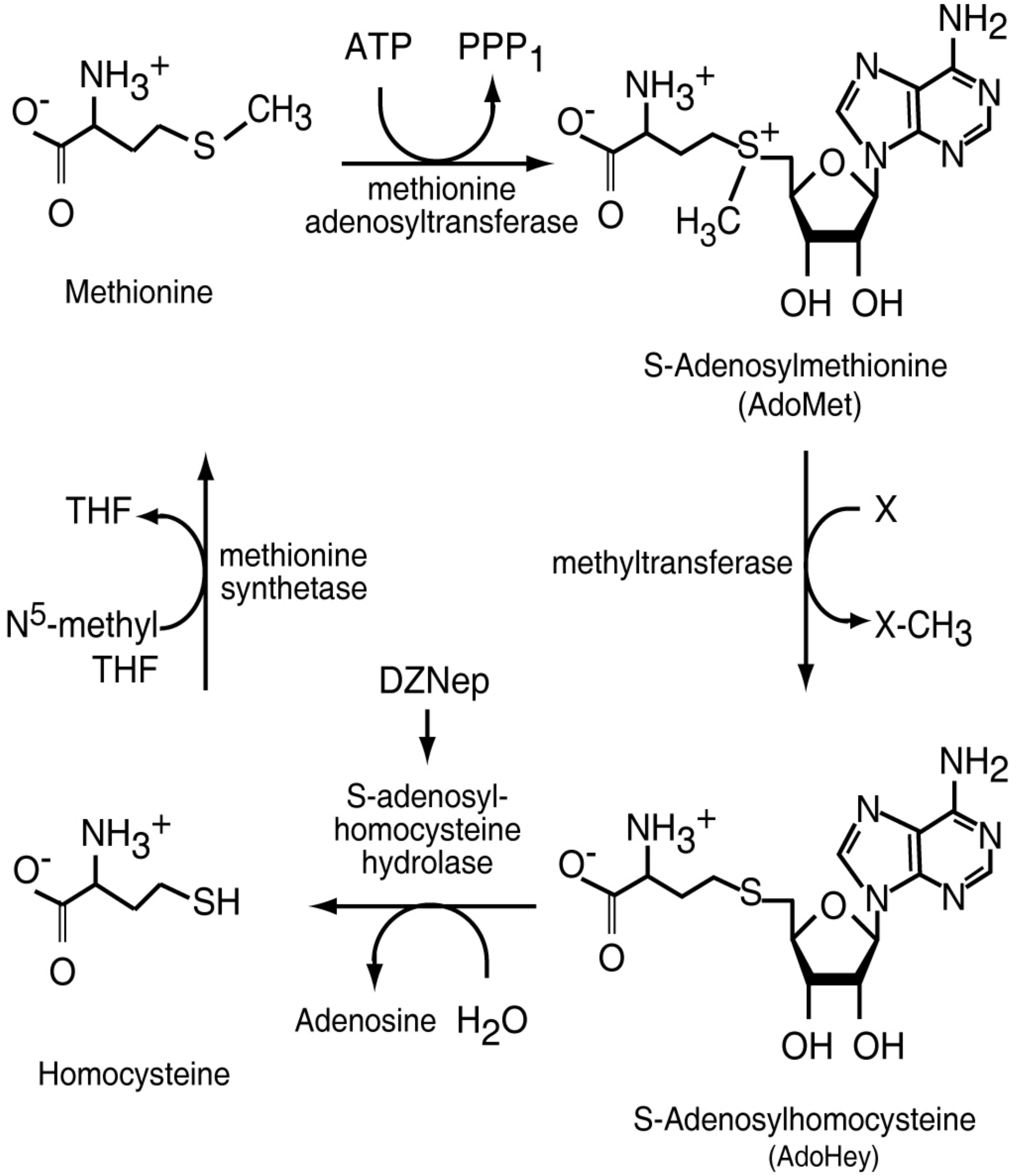


DZNep is a potent competitive inhibitor of S-adenosyl-homocysteine (AdoHey) hydrolase with an estimated Ki of 5 x $10^{-11}$M with adenosine as substrate (**Fig. 1**). The inhibition of this enzymatic reaction leads to an accumulation of AHH which acts as a competitive inhibitor of S-adenosyl-methionine, the methyl donor in enzymatic methylation reactions. DZNep ($c^3$Nep) reduced the viability (**Fig. 2B**) and induced differentiation (**Fig. 2C**) of the leukemic cells.

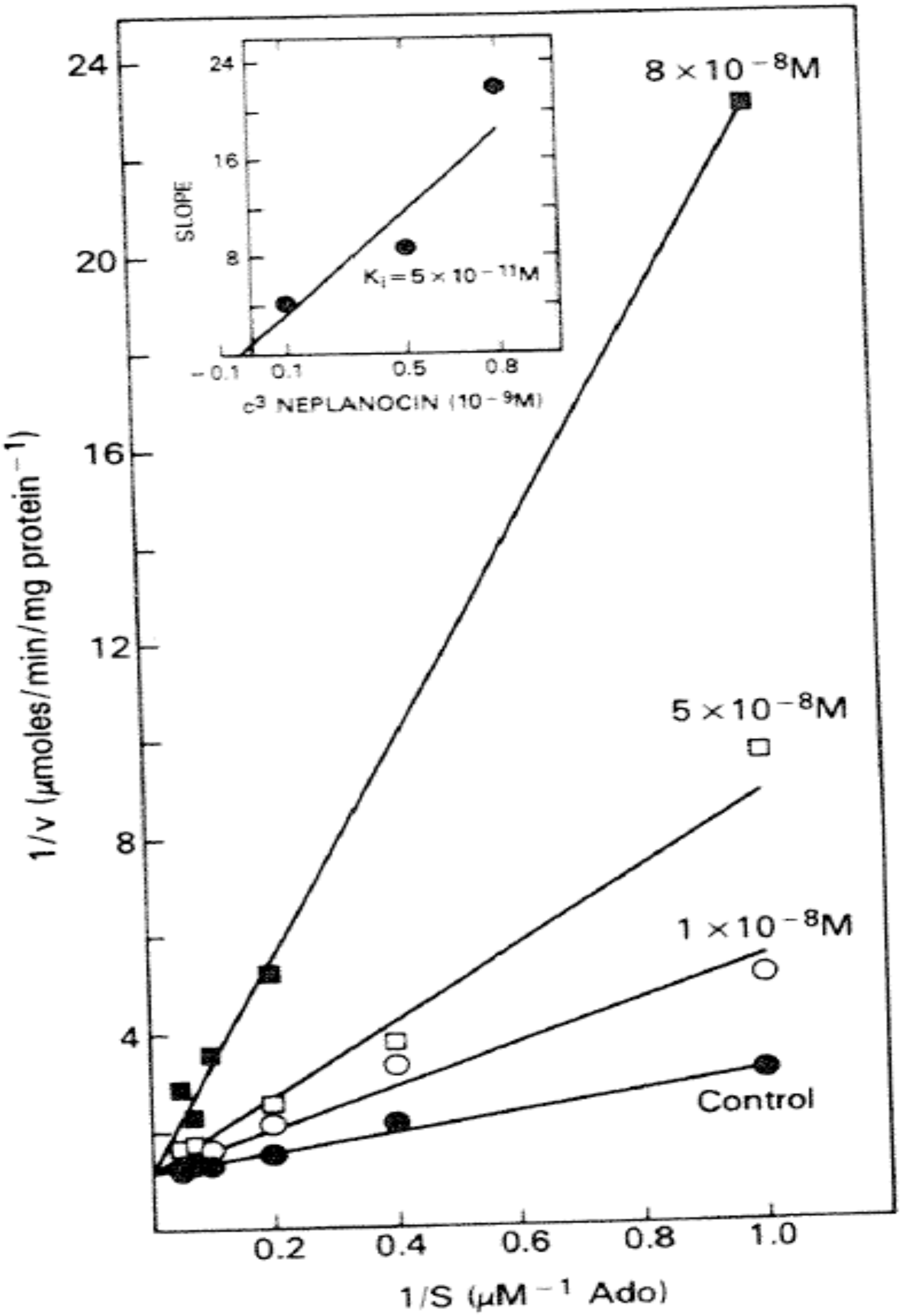


**Fig. 1.** Competitive inhibition by 3-deazaplanocin (c³ NEPLANOCIN) of hamster liver AdoHcy hydrolase.

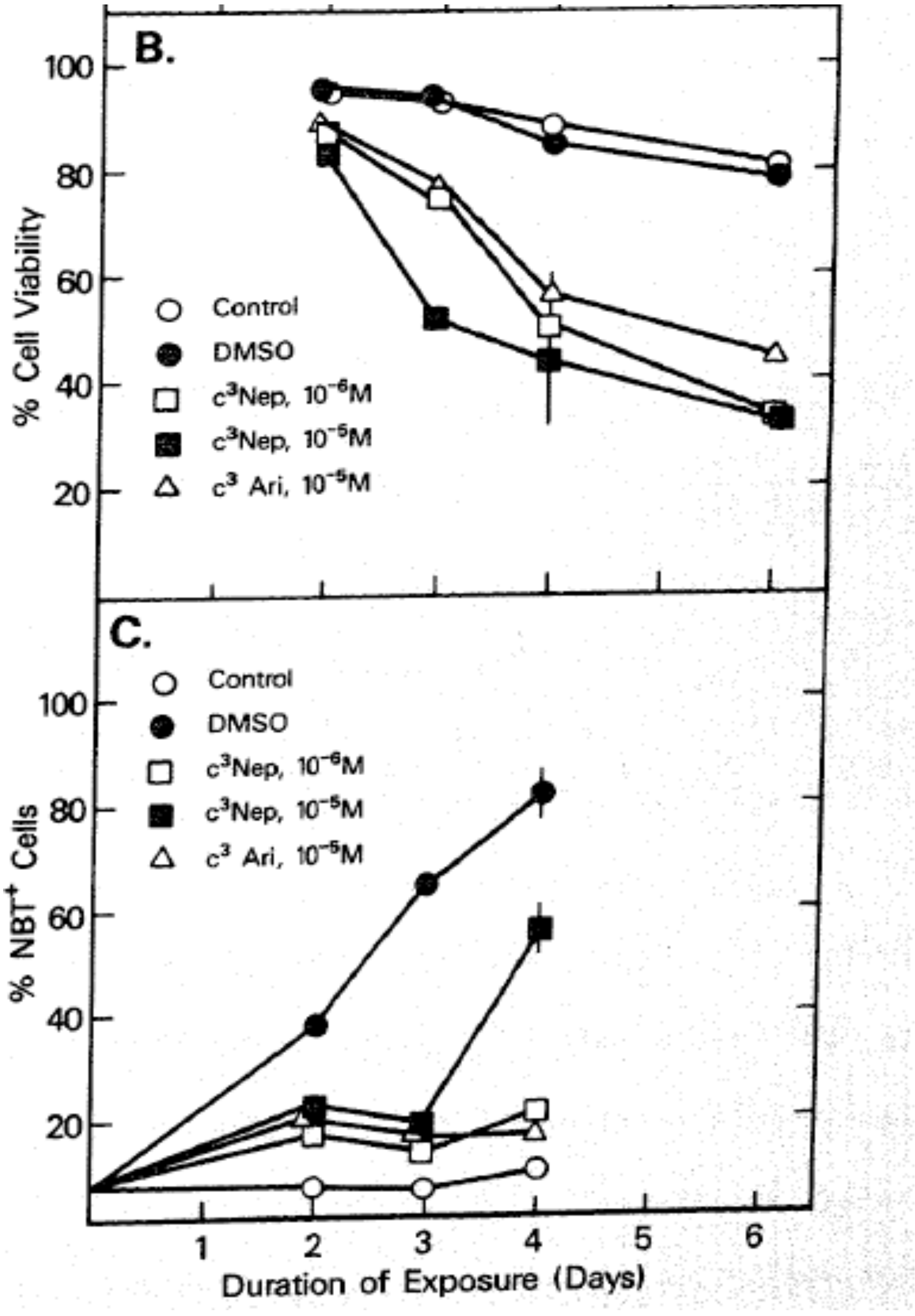


**Fig. 2.** Cell viability and differentiation of HL-60 leukemic cells following treatment with 3-deazaneplanocin ($c^3$Nep) or 3-dezaaristeromycin ($c^3$Ari). Cells were treated continuously with $10^{-6}$ or $10^{-5}$ M of these agents. (**B**) Cell viability was determined by trypan blue exclusion. (**C**) Differentiation was determined by NBT reduction.

# 6. IN VITRO ANTICANCER ACTIVITY

## 6.1 LEUKEMIA-1

Fiskus W et al. Combined epigenetic therapy with the histone methyltransferase EZH2 inhibitor 3-deazaneplanocin A and the histone deacetylase inhibitor panobinostat against human AML cells . Blood 2009 114: 2733-2743.

Treatment of OCI-AML3 cells with DZNep (1.0 µM) resulted in an accumulation of cells in the G0/G1 phase (58.5%) with a decrease in the number of cells in S phase (35.2%) and G2/M phases (6.3%) of the cell cycle ($P < .05$) (**Figure 1A**). Exposure to DZNep dose dependently induced apoptosis to a greater extent in OCI-AML3 than HL-60 cells (**Figure 1B**). As shown in **Figure 1C**, DZNep treatment also induced caspase-dependent cleavage of PARP more in HL-60 than in OCI-AML3 cells (Figure 1C). In addition, treatment with DZNep (200 nM to 2.0 µM) for 48 hours, dose dependently, inhibited colony growth of OCI-AML3 and HL-60 cells (**Figure 1D**).

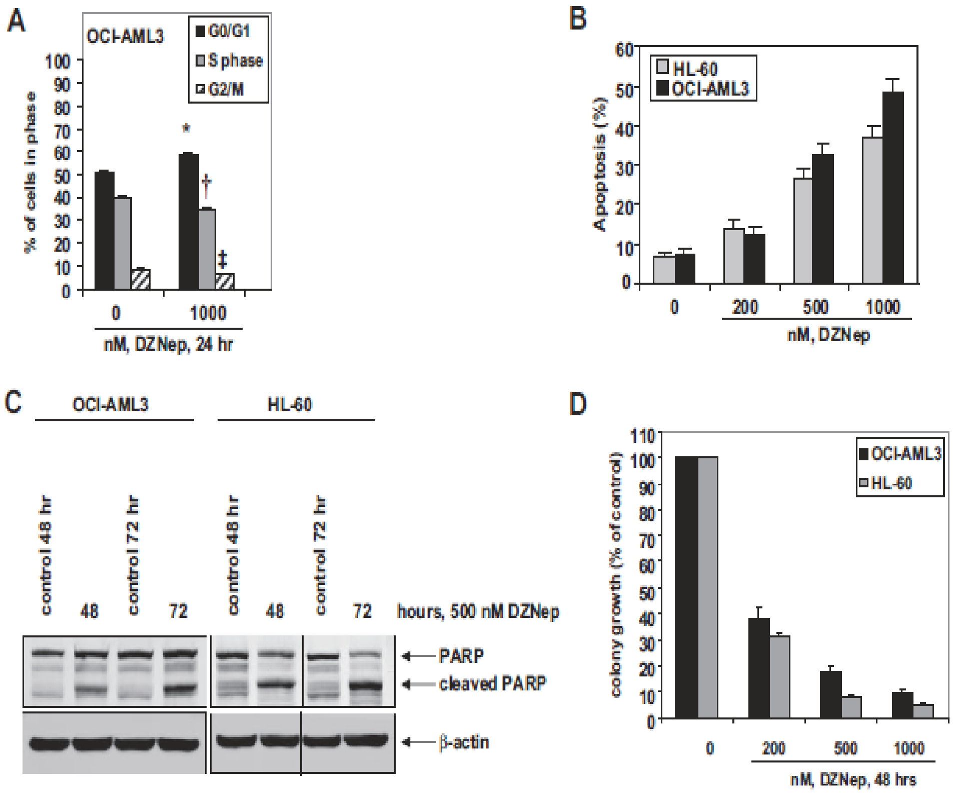


**Figure 1.** Treatment with DZNep induces apoptosis in a dose- and time-dependent manner and markedly reduces clonogenic survival of AML cells. (**A**) OCI-AML3 cells were treated with DZNep for 24 hours, then stained with propidium iodide, and cell-cycle status was determined by flow cytometry. *G0/G1, †S-phase and ‡G2/M values significantly different from untreated cells; (**B**) HL-60 and OCI-AML3 cells were treated with DZNep for 72 hours. Then, the cells were stained with annexin V, and apoptotic cells were determined by flow cytometry. Columns represent the mean of 3 experiments; bars represent SEM. (**C**) OCI-AML3 and HL-60cells were treated with 500 nmol/L DZNep, for 48 and 72 hours. Cell lysates were prepared and immunoblot analysis was performed for PARP. The expression levels of ß-actin in the lysates served as the loading control. (**D**) OCI-AML3 and HL-60 cells were treated with DZNep for 48 hours. After treatment, colony growth in semisolid media was assessed after 7 days. Bar graphs represent the mean percentage values SEM of untreated colony growth.

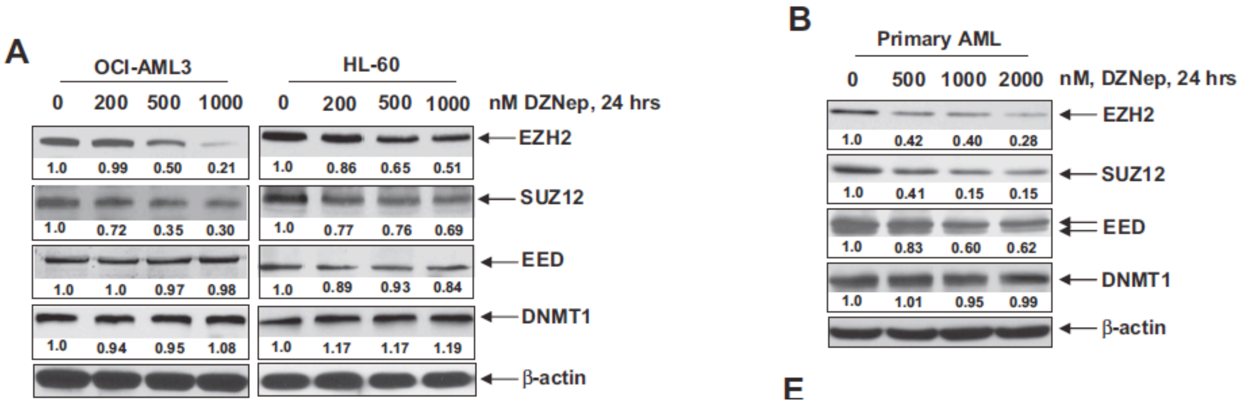


**Figure 2**. Treatment with DZNep depletes expression of polycomb group proteins EZH2, SUZ12, and EED in cultured and primary AML cells. (**A**) OCI-AML3 and HL-60 cells were treated with DZNep for 24 hours. Immunoblot analysis was performed for EZH2, SUZ12, EED, and DNMT1. The expression levels of β-actin served as the control. (**B**) PrimaryAML cells were treated with DZNep for 24 hours. Immunoblot analysis was performed for EZH2, SUZ12, EED, and DNMT1. The expression levels of β-actin served as the control.

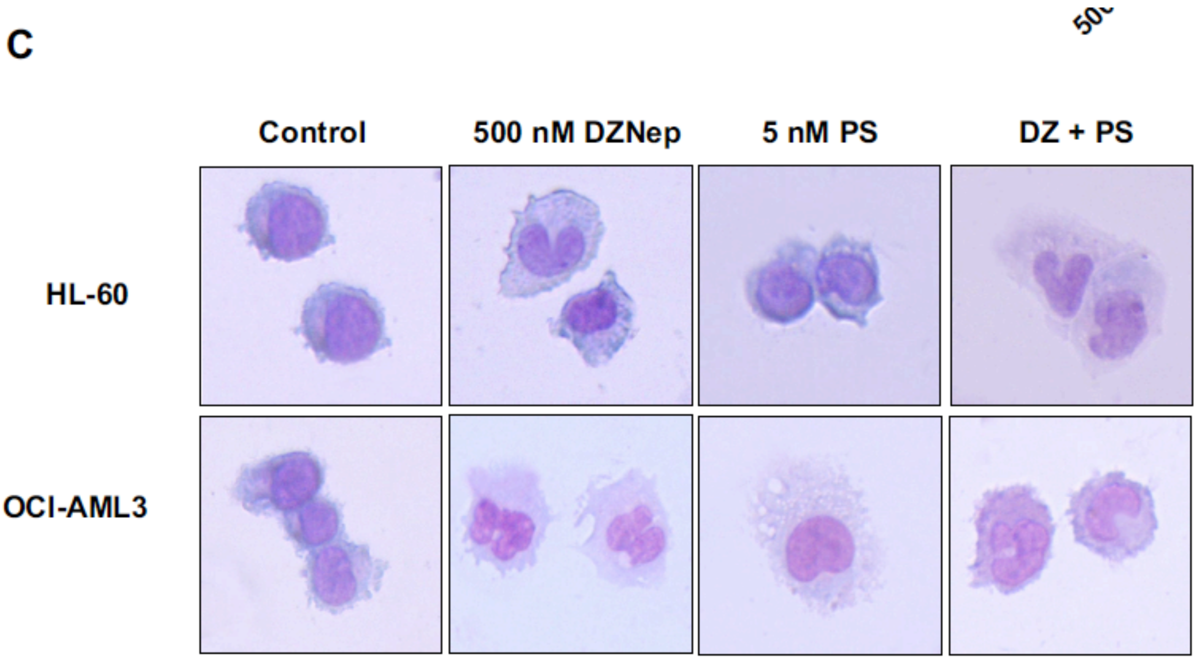


**Figure 6.** **(C**) HL-60 and OCI-AML3 cells were treated with the indicated concentrations of DZNep and/or PS for 72 hours. After treatment, the cells were cytospun onto glass slides,Wright stained, and observed with a microscope to assess cellular morphology. The DZNep treatment increased the differentiation of the HL-60 andOCI-AML3 cells.

**6.2 LEUKEMIA-2 (IN VITRO ANTICANCER ACTIVITY)**

Momparler RL et al., Synergistic antileukemic action of a combination of inhibitors of DNA methylation and histone methylation. Leuk Res. 2012;36:1049-1054.

A remarkable synergy was observed against HL-60 myeloid leukemic cells by 5AZA+DZN.

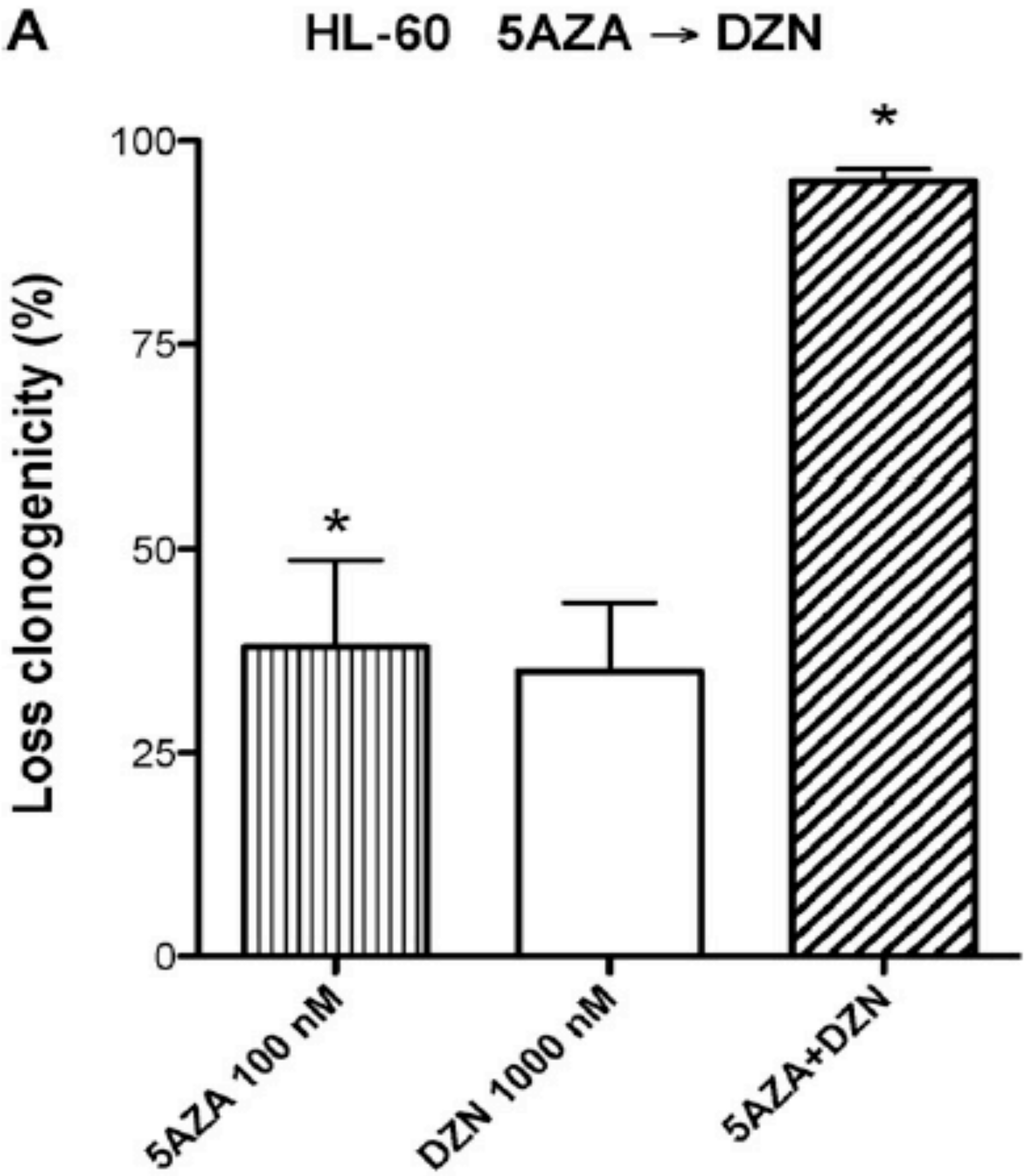


**Figure. 1**. Clonogenic assay of leukemic cells after sequential treatment with 5-AZACdR (5AZA) and DZNep (DZN). (A) HL-60 cells were treated with 100 nM 5AZA and/or at 24 h 1000 nM DZN was added to the medium. At 48 h cells were placed in soft agar medium for colony assay. The results are expressed as mean ± SEM, n = 3.

### 6.3 LEUKEMIA-3 (IN VITRO ANTICANCER ACTIVITY)

Fujiwara T et al., 3-Deazaneplanocin A (DZNep), an inhibitor of S-adenosylmethionine-dependent methyltransferase, promotes erythroid differentiation. J Biol Chem. 2014;289(12):8121-8134.

The DZNep treatment reduced the level of EZH2 significantly (**Fig. 1A**) and a moderate decrease in the level of trimethyl H3K27 (**Fig. 2C**) in K562 leukemic cells. There was a delay in the inhibition of growth of the leukemic cells by DZNep, which became more apparent at 48 h and very significant at 72 h (**Fig. 1D**). An increase in the protein expression of p27 was observed after DZNep treatment (**Fig. 1E**) The DZNep treatment also exhibited an arrest in the progression of G1 phase cells to enter the S phase (**Fig. 1 F**). DZNep treatment significantly induced erythroid differentiation of K562 leukemic cells, as assessed by benzidine staining (**Fig. 2A**) and quantitative RT-PCR analysis for representative erythroid-related genes (**Fig. 2C,D**). The effects of DZNep in human primary erythroblasts derived from cord blood CD34-positive cells showed that the treatment significantly induced erythroid-related genes. DZNep-treated K562 leukemic cells revealed a marked up-regulation of SLC4A1, EPB42 and ALAS2.

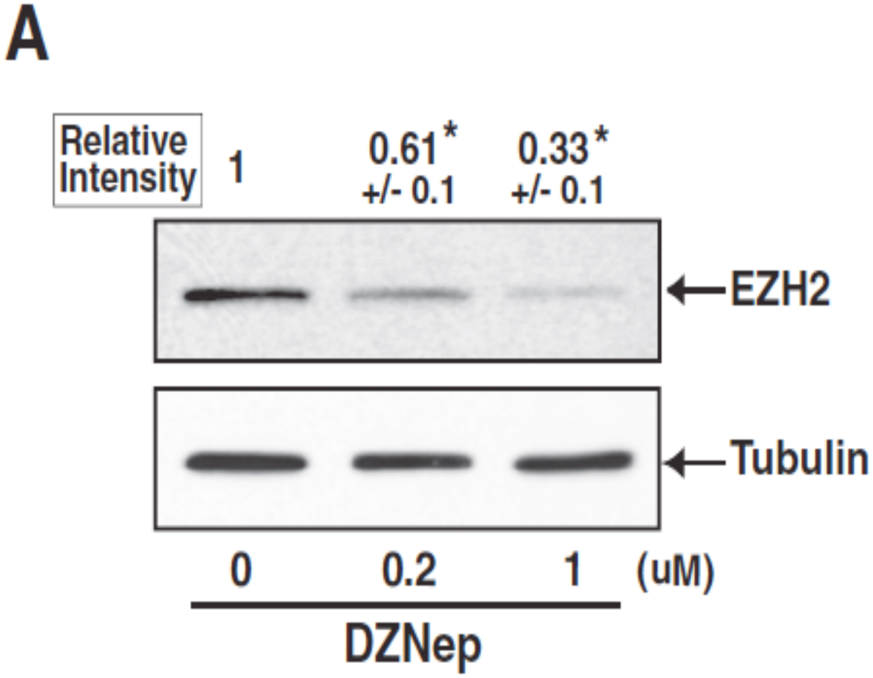


**FIGURE 1. A,** DZNep treatment suppressed proliferative activity of K562 cells. Western blotting to detect EZH2 protein in DZNep-treated K562 cells. The K562 cells were treated with DZNep (0.2 and 1 μM, respectively) for 72 h. EZH2 protein signal was quantified and normalized to that of α-tubulin. Statistical analyses were conducted by comparing with control signal (defined as 1). mRNA expression was normalized relative to that of GAPDH.

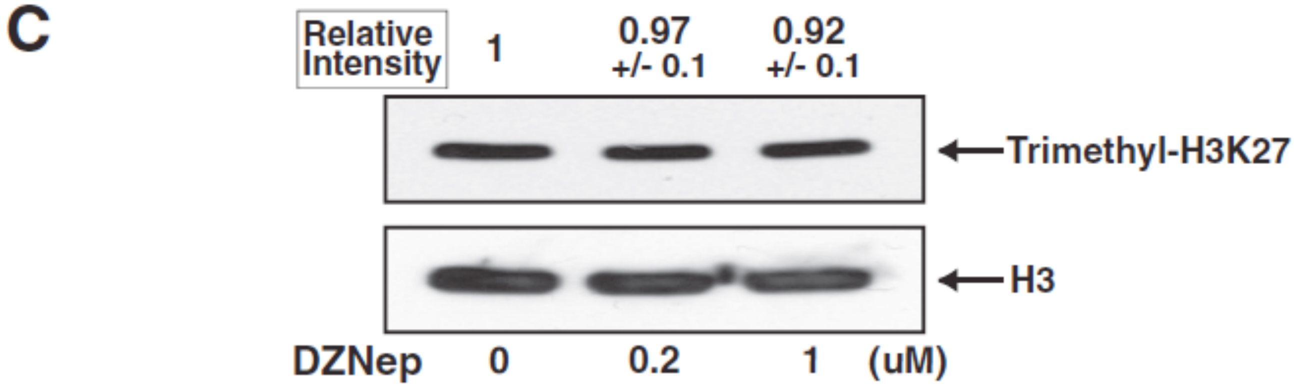


**FIGURE 1. C,** Western blotting to assess H3K27me3 level in DZNep-treated 0.2 and 1 μM, respectively) for 72 h K562 cells. K3K27me3 level was quantified and normalized to that of H3. Statistical analyses were conducted by comparing with control signal (defined as 1).

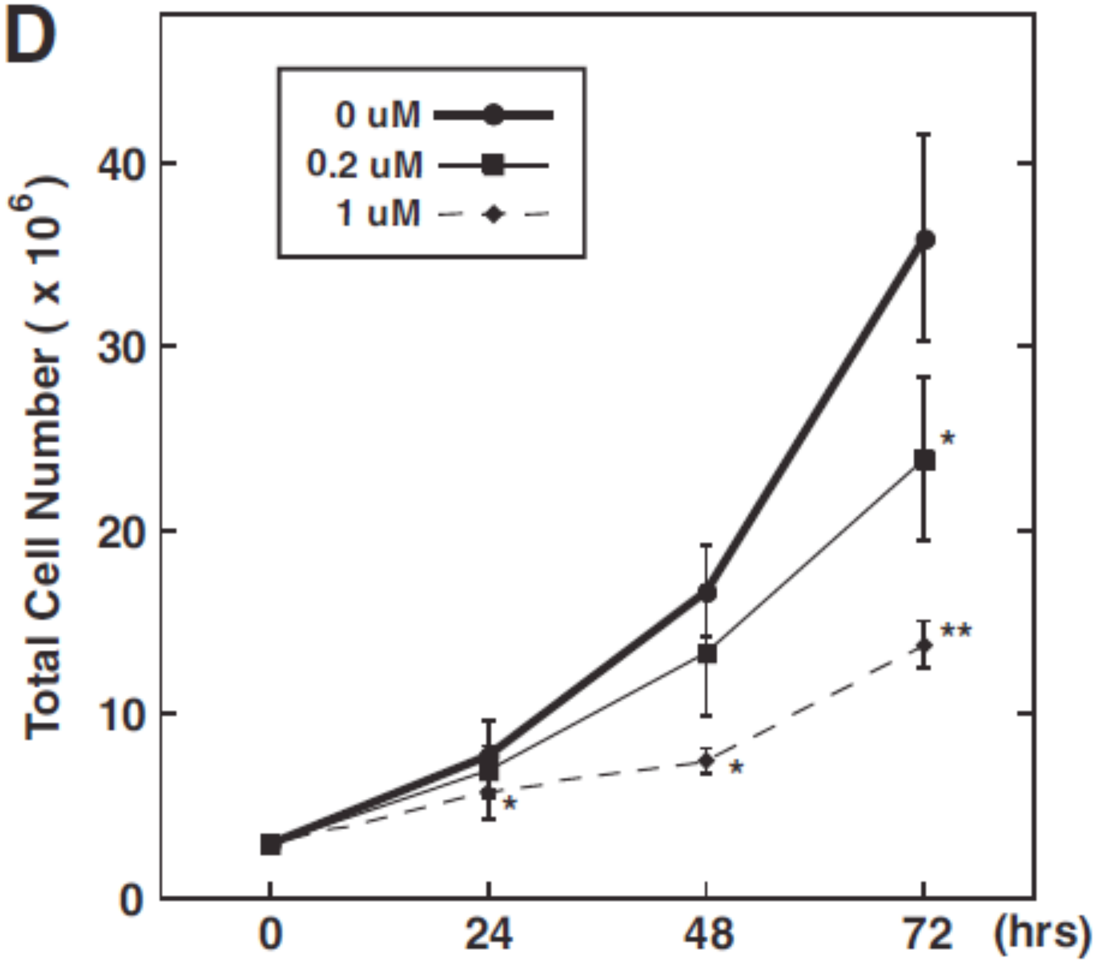


**FIGURE 1. D,** Changes in total cell number after DZNep treatment.

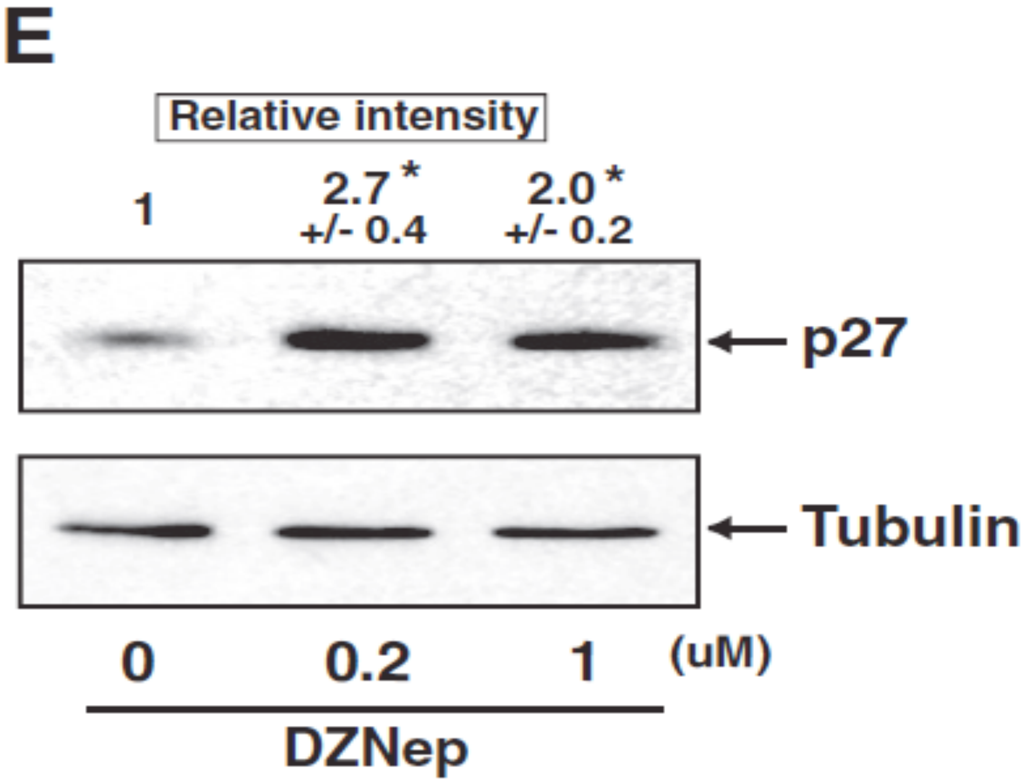


**FIGURE 1**. **E,** Western blotting to detect p27 protein in DZNep-treated K562 cells. p27 protein signal was quantified and normalized to that of α-tubulin. Statistical analyses were conducted by comparing with control signal (defined as 1).

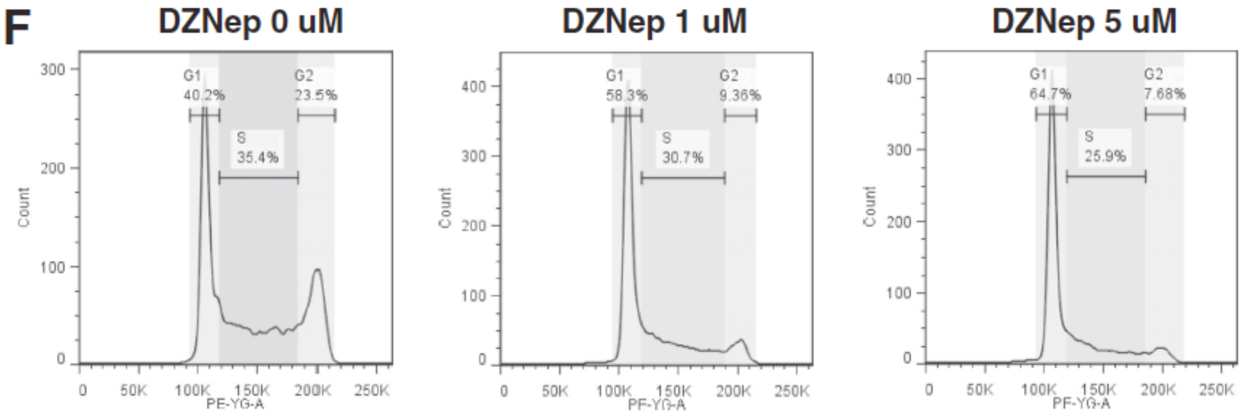


**FIGURE 1. F,** Effect of DZNep (1 and 5 μM, respectively, for 24 h) on cell cycle in K562 cells. The percentage of cells in each cell cycle was measured using a flow cytometer. In all analyses, we observed increased G1 as well as decreased G2 phase cells.

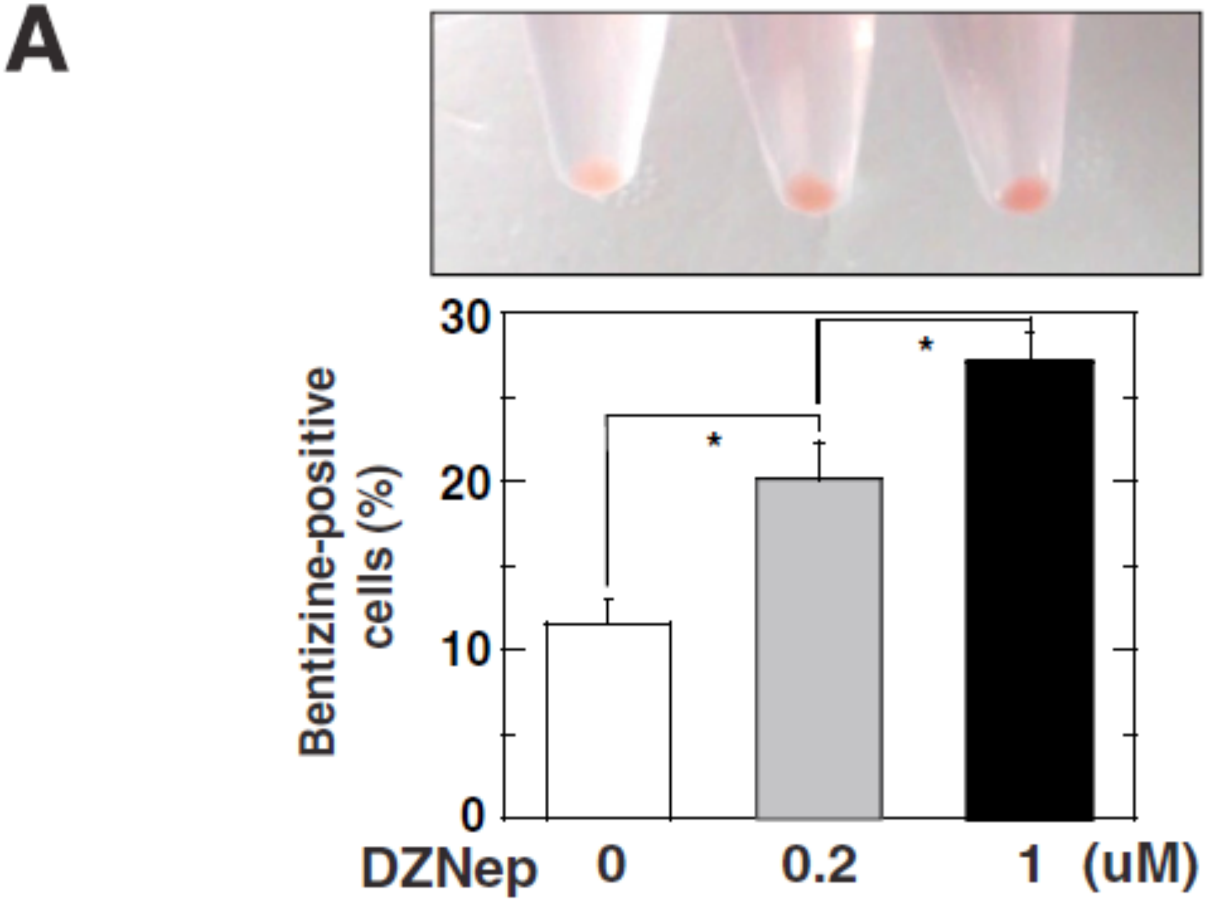


**FIGURE 2. A,** DZNep treatment induces erythroid differentiation of K562 cells. A, cell pellet (top) and the percentage of benzidine stain-positive cells (bottom) after DZNep treatment in K562 cells. The cells were treated with DZNep at doses of 0.2 and 1 μM for 72 h.

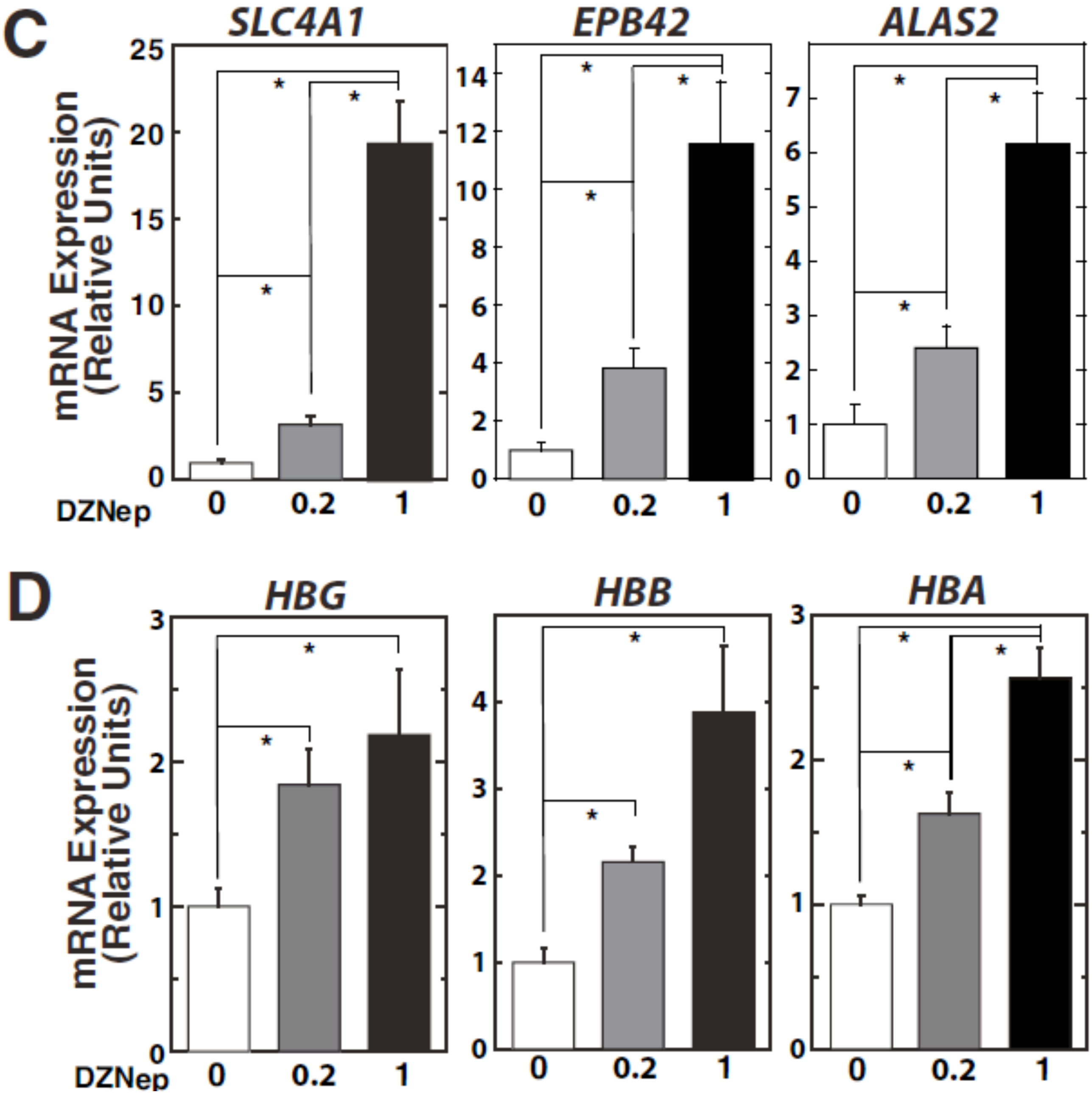


**FIGURE 2. C**, **D**, Quantitative RT-PCR-based (mean ± S.D.) of mRNA levels of erythoid-related genes in the leukemic cells after treatment with DZNep. The levels of mRNA were normalized relative to GAPDH.

## 6.4 LEUKEMIA-4 (IN VITRO ANTICANCER ACTIVITY)

Zhou J et al., The histone methyltransferase inhibitor, DZNep, up-regulates TXNIP, increases ROS production, and targets leukemia cells in AML. Blood. 2011;118 (10):2830-2839.

DZNep treatment of leukemic cells increased apoptosis (**Fig. 1A**), reduced EZH2 and Suz12 protein expression and reduced the level of H3K27me3 (**Fig. 2B**). DZNep reduced colony formation by primary leukemic stem cells (LSC) to a greater extent than normal hematopoietic stem cells (HSC) (**Fig. 2B**). DZNep increased TXNIP protein expression and reactive oxygen species (ROS) production in MOLM-4 leukemic cell line. TXNIP = thioredoxin-binding protein 2. Increase in TXNIP caused an increase in reactive oxygen species (ROS) production.

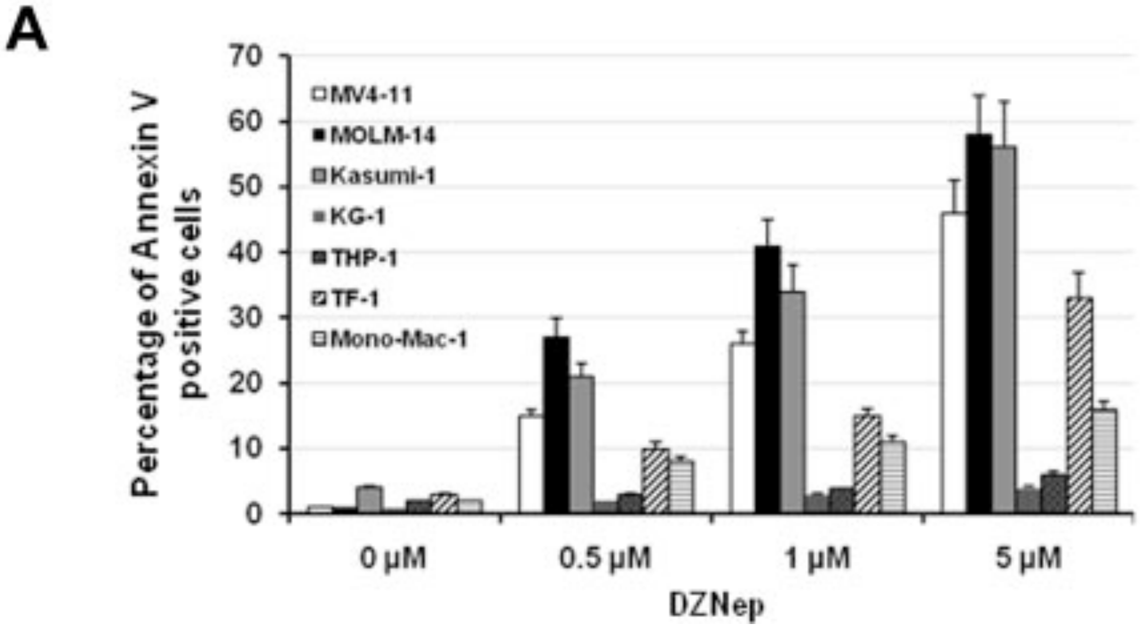


**Figure 1A.** AML cell lines were treated with different concentrations of DZNep for 48 hours. Cells were stained with annexin V/PI and analyzed by flow cytometry for induction of apoptosis.

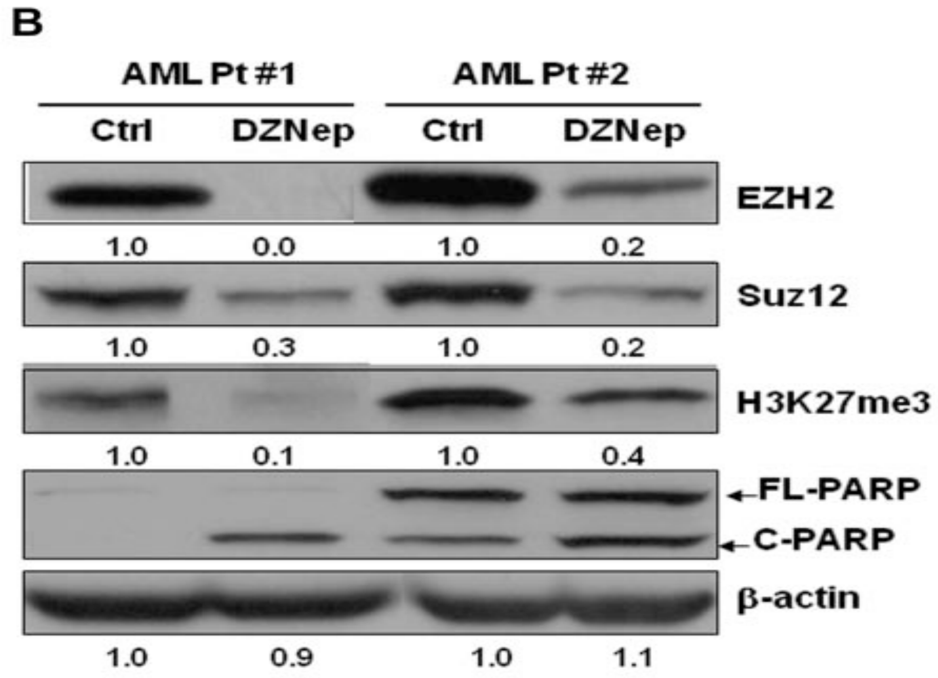


**Figure 2B.** Primary AML cells were treated with 5 μM DZNep for 48 hours. Cells were lysed and subjected to immunoblot analysis using the indicated primary antibodies.

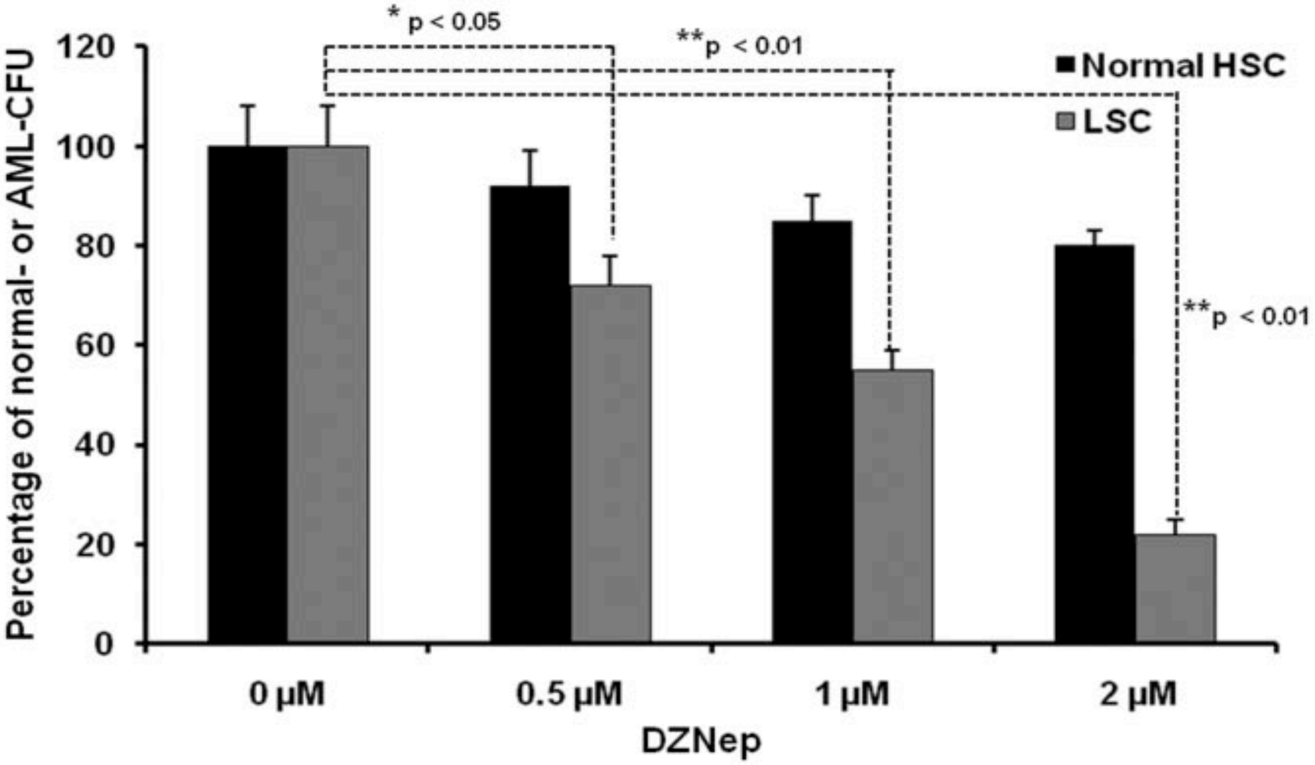


**Figure 3.** The effect of 14 day treatment of DZNep on colony formation of leukemic stem cells (LSC) from AML patients and normal hematopoietic stem cells (HSC) from healthy donor. Methylcellulose-based colony assay was performed on CD34+CD38- bone marrow cells.

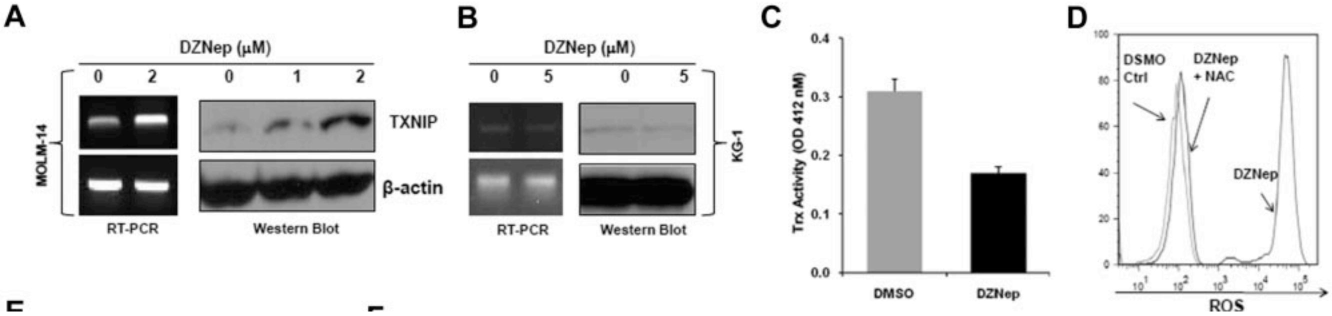


**Figure 4.** DZNep treatment increased TXNIP expression and ROS production. TXNIP expression was determined by RT-PCR and Western blot analysis in MOLM-14 (**A**) and KG-1 (**B**) leukemic cells treated with either DMSO control or DZNep for 24 hours. (**C**) Trx activity in MOLM-14 cells treated with DMSO control or 2 μM DZNep for 48 hours was measured at 412 nM. (**D**) FACS analysis of ROS production in MOLM-14 cells treated with DMSO control, DZNep, or NAC + DZNep. Trx activity = thioredoxin "insulin-reducing" assay. ROS = reactive oxygen species. NAC = N-acetyl-l-cysteine.

## 6.5 LYMPHOMA (IN VITRO ANTICANCER ACTIVITY)

Fiskus W et al. Superior efficacy of a combined epigenetic therapy against human mantle cell lymphoma cells. Clinical Cancer Res 2012;18:6227-6238.

3-Deazaneplanocin A (DZNep) induces cell cycle arrest and apoptosis against human mantle cell lymphoma cells (MCL).

**Table 1.** Treatment with DZNep induces cell-cycle arrest of MCL cells in a dose-dependent manner

| | % of cells | | |
|---|---|---|---|
| Cells and treatment | $G_0$–$G_1$ | S | $G_2$/M |
| MO2058 | | | |
| Untreated | 35.51 ± 1.20 | 60.98 ± 0.47 | 3.51 ± 0.77 |
| 0.5 µmol/L DZNep | 36.75 ± 0.56 | 58.26 ± 0.52 | 4.99 ± 0.26 |
| 1.0 µmol/L DZNep | 46.63 ± 1.87 | 46.67 ± 1.90 | 6.70 ± 0.11 |
| 2.0 µmol/L DZNep | 52.89 ± 1.23 | 39.42 ± 0.86 | 7.69 ± 0.47 |
| JeKo-1 | | | |
| Untreated | 49.08 ± 1.12 | 48.70 ± 1.02 | 2.22 ± 0.12 |
| 0.5 µmol/L DZNep | 60.22 ± 0.10 | 36.46 ± 0.23 | 3.32 ± 0.32 |
| 1.0 µmol/L DZNep | 67.77 ± 0.98 | 28.76 ± 1.21 | 3.47 ± 0.28 |
| 2.0 µmol/L DZNep | 72.06 ± 0.54 | 24.18 ± 0.70 | 3.76 ± 0.20 |

NOTE: Cell-cycle status of MO2058 and JeKo-1 cells treated with the indicated concentrations of DZNep for 24 hours. Values represent the mean of 3 independent experiments ± SEM.

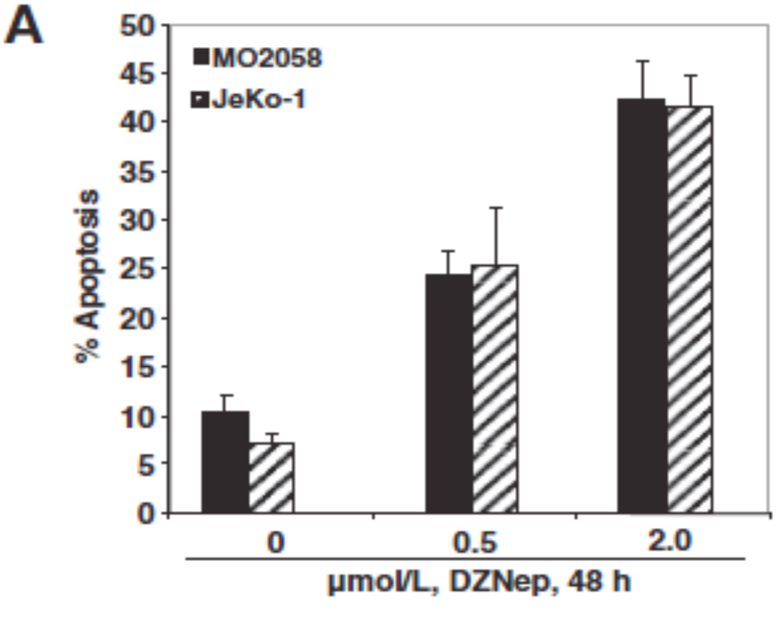


**Figure 3A.** Treatment with DZNep induces dose-dependent apoptosis of mantel cell lymphoma (MCL) cells. MO2058 and JeKo-1 cells were treated with DZNep as indicated for 48 hours. The percentages of annexin-V–positive apoptotic cells were determined by flow cytometry. Columns, mean of 3 experiments; bars, SEM.

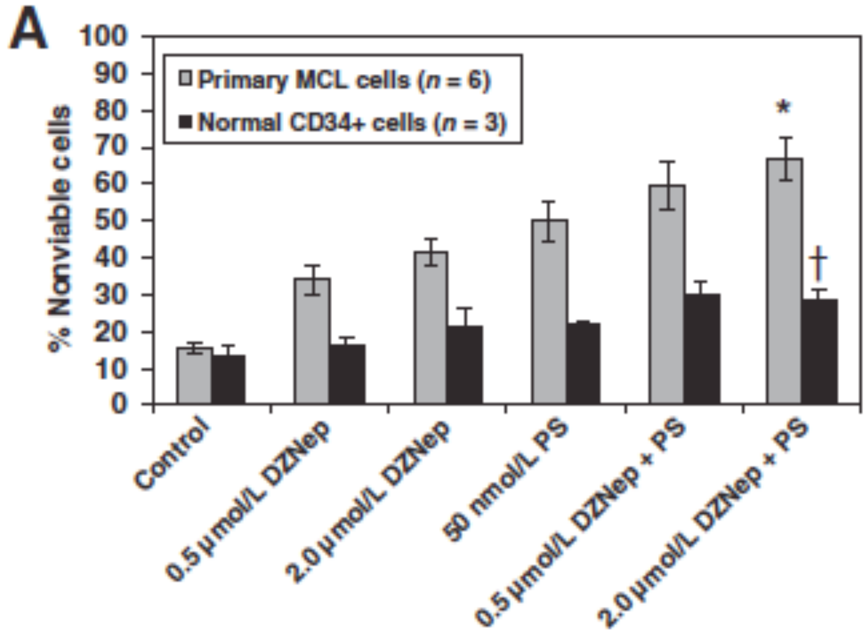


**Figure 7A**. Cotreatment with DZNep and panobinostat (PS) exerts synergistic cytotoxic effects against primary mantel cell lymphoma (MCL) cells. Primary MCL (n = 6) and normal CD34+ cells (n = 3) were treated with DZNep and/or PS for 48 hours. *, loss of viability values significantly greater ($P < 0.05$) than those resulting from treatment with either agent alone. †, values significantly less in normal CD34þ progenitor cells than those observed in primary MCL cells. Nonviable cells were determined by trypan blue dye uptake.

### 6.6 T-CELL LEUKEMIA (IN VITRO ANTICANCER ACTIVITY)

Shen J et al., Growth Inhibition Accompanied by MOB1 Upregulation in Human Acute Lymphoid Leukemia Cells by 3-Deazaneplanocin A. Biochem Genet (2015) 53:268–279.

The objection of this study was to investigate the effect of 3-deazaneplanocin A (DZNep) on human T-cell acute lymphoid leukemia (T-ALL) cells. The human T-ALL cell line Molt4 was treated with DZNep, and cell proliferation was examined. For a 48 h exposure the inhibition of the growth of Molt4 cells increased with the concentration of DZNep (Fig 16). The results indicate that DZNep is a promising therapeutic compound for the treatment of human T-ALL.

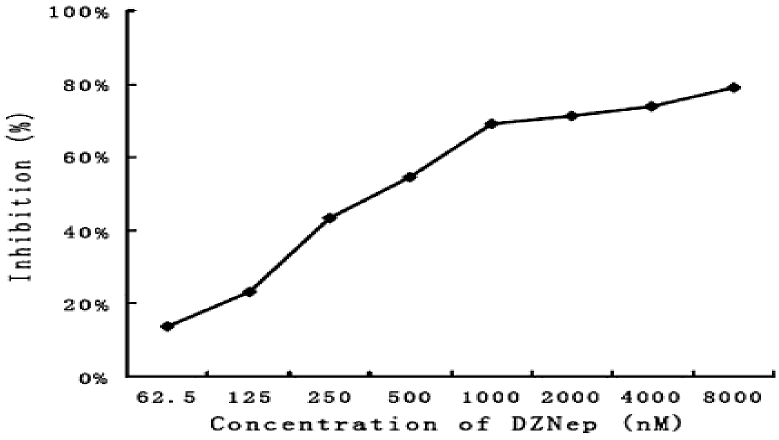


**Figure 16.** Inhibitory effect of DZNep on the growth of Molt4 cells. Molt4 cells were treated with DZNep at concentrations ranging from 62.5 nM to 8,000 nM for 48 h. The number of living cells was determined by measuring the absorbance at 450 nm with addition of CCK-8 reagent. Untreated Molt4 cells were used as the blank control. Differences in the number of living cells between the treatment groups and the control group were expressed as percentage of inhibition. Data derived from two separate experiments with triplicate wells per condition.

## 6.7 BREAST CANCER (IN VITRO ANTICANCER ACTIVITY)

Puppe J et al., BRCA1-deficient mammary tumor cells are dependent on EZH2 expression and sensitive to Polycomb Repressive Complex 2-inhibitor 3-deazaneplanocin A. Breast Cancer Res 2009;11:R63.

The BRCA1-deficient mammary tumor cells are more sensitive to the growth inhibitory action of DZNep than the BRCA1-proficient mammary tumor cells (**Fig 3d**).

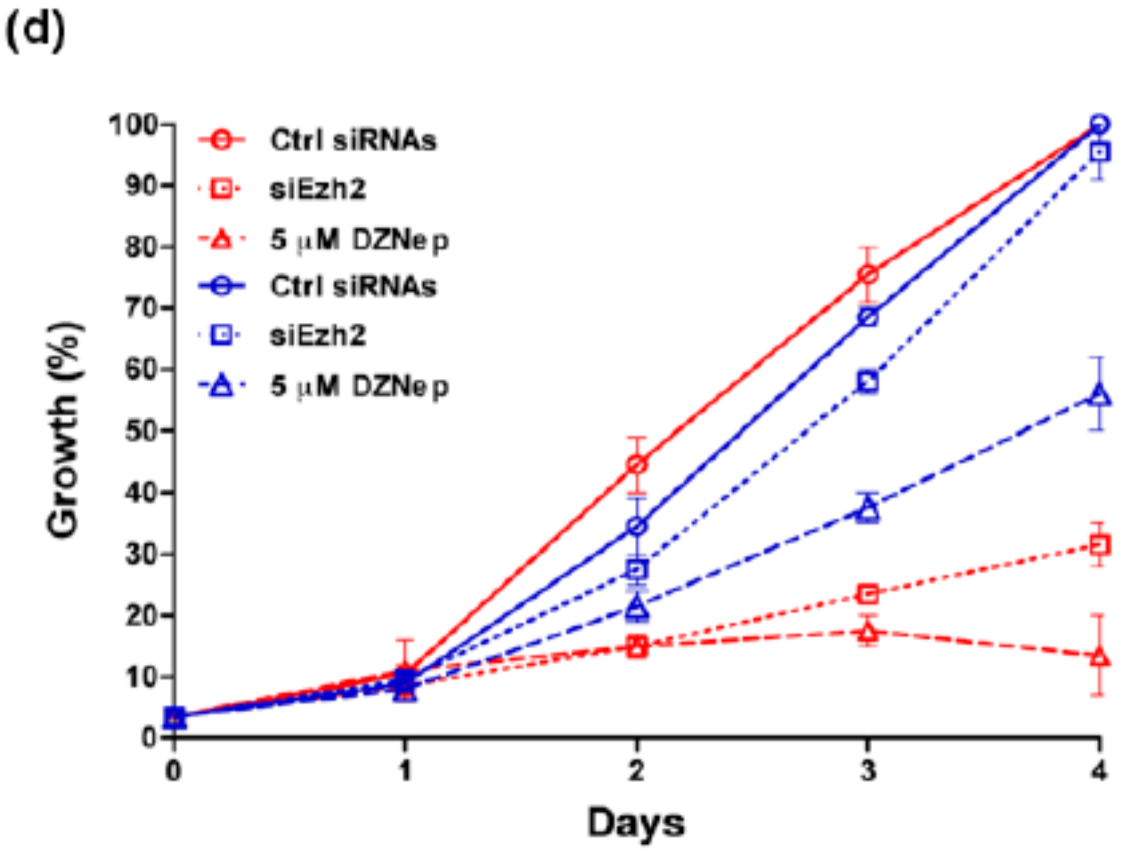


**Figure 3(d)** Growth curves of mammary tumor cells: KB1P (BRCA1-deficient depicted in red) and KP (BRCA1-proficient depicted in blue) treated with control (ctrl siRNAs), siRNAs against Ezh2 or 5 μM DZNep. Data measured by Cell Titer Blue and represented as the mean ± standard error of the mean (three independent experiments). KB1P cells, deficient in BRAC1; KP cells, wild type BRAC1.

The BRCA1-deficient mammary tumor cells are more sensitive to the growth inhibitory action of DZNep than the BRCA1-proficient mammary tumor cells (**Fig 4a**).

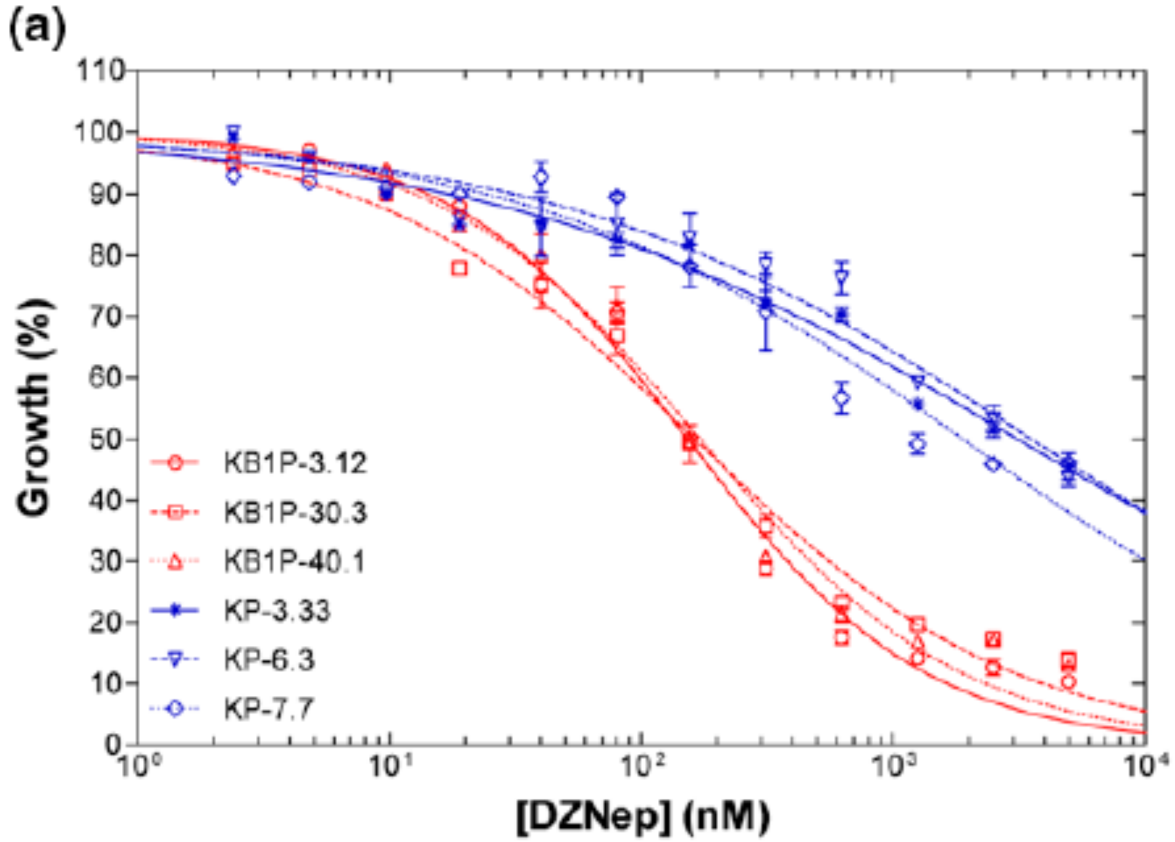


**Figure 4a.** Chemical EZH2-inhibitor DZNep selectively kills BRCA1-deficient mammary tumor cells. Representative growth inhibition curves for BRCA1-deficient cell lines (KB1P), in red) and BRCA1-proficient cell lines (KP), in blue) treated with 3-deazaneplanocin A (DZNep). A serial dilution of DZNep was added to the cells and cell viability was measured five days later (each data point represents the mean ± SEM of 3 experiments).

The $IC_{50}$ values of the BRCA1-deficient mammary tumor cells are much lower than the $IC_{50}$ values of the BRCA1-proficient mammary tumor cells (**Table 1**).

**Table 1 $IC_{50}$ values of DZNep on BRCA1-proficient vs BRCA1-deficient mammary tumor cells**

| Cell line | nM DZNep (SEM) |
|---|---|
| *BRCA1-proficient* | |
| KP-3.33 | 3202 (284) |
| KP-6.3 | 3467 (277) |
| KP-7.7 | 2163 (63) |
| *BRCA1-deficient* | |
| KB1P-3.12 | 154 (14) |
| KB1P-30.3 | 163 (13) |
| KB1P-40.1 | 171 (9) |

## 6.8 CHONDROSARCOMA CELLS (IN VITRO ANTICANCER ACTIVITY)

Girard N et al., 3-Deazaneplanocin A (DZNep), an inhibitor of the histone methyltransferase EZH2, induces apoptosis and reduces cell migration in chondrosarcoma cells. PLoS One. 2014;9(5):e98176.

Chondrosarcomas highly express EZH2. In vitro, DZNep inhibits EZH2 protein expression, and subsequently reduces the trimethylation of lysine 27 on histone H3 (H3K27me3). DZNep induces cell death of chondrosarcoma cell lines by apoptosis, while it slightly reduces growth of normal chondrocytes. In addition, DZNep reduces tumor cell migration. These results indicate that an epigenetic therapy that pharmacologically targets EZH2 via DZNep may constitute a novel approach to treat chondrosarcomas.

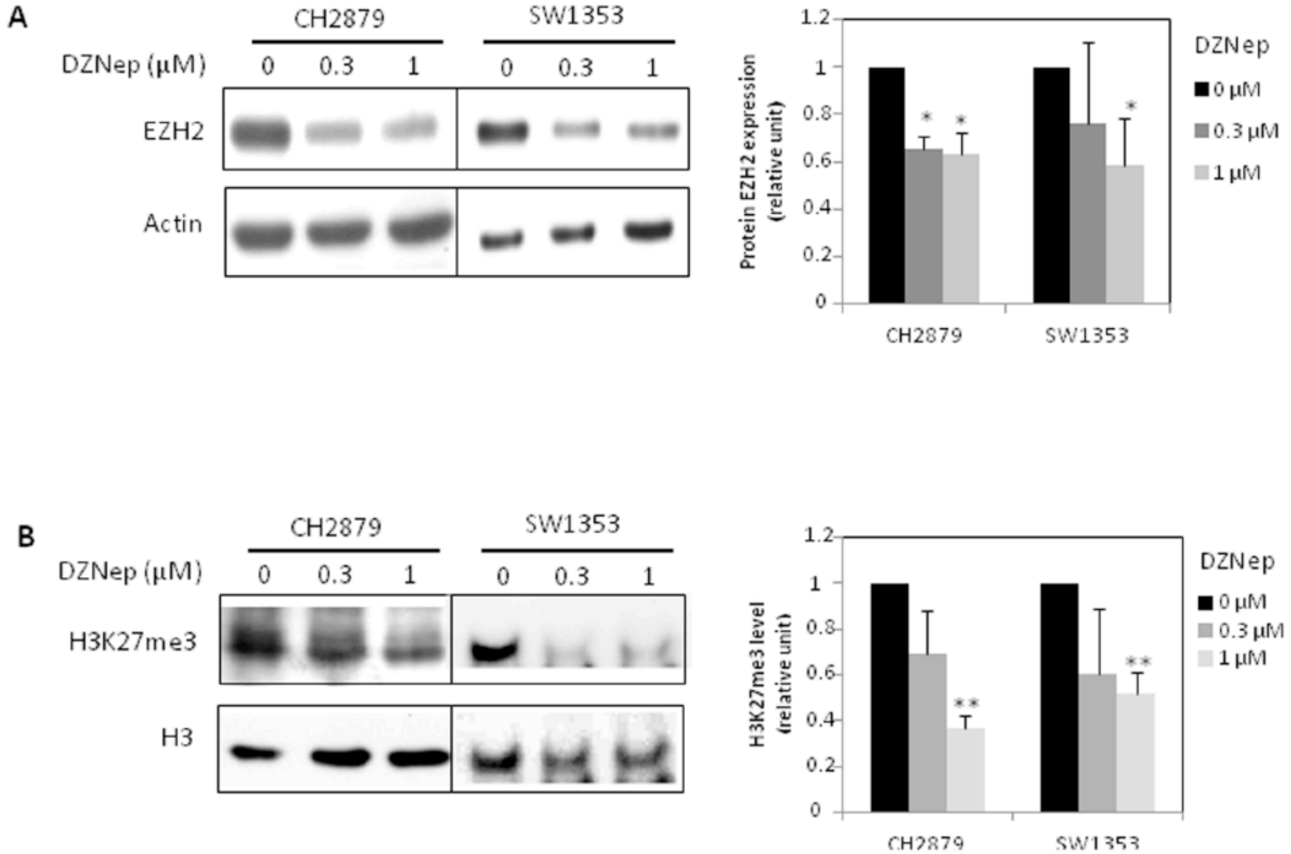


**Figure 2.** DZNep reduces EZH2 protein expression and H3K27me3. SW1353 and CH2879 cells were treated with DZNep (1 mM) for 72 h. A) EZH2 protein expression was analyzed by Western blot. B) H3K27me3 was also analyzed by Western blot. H3.

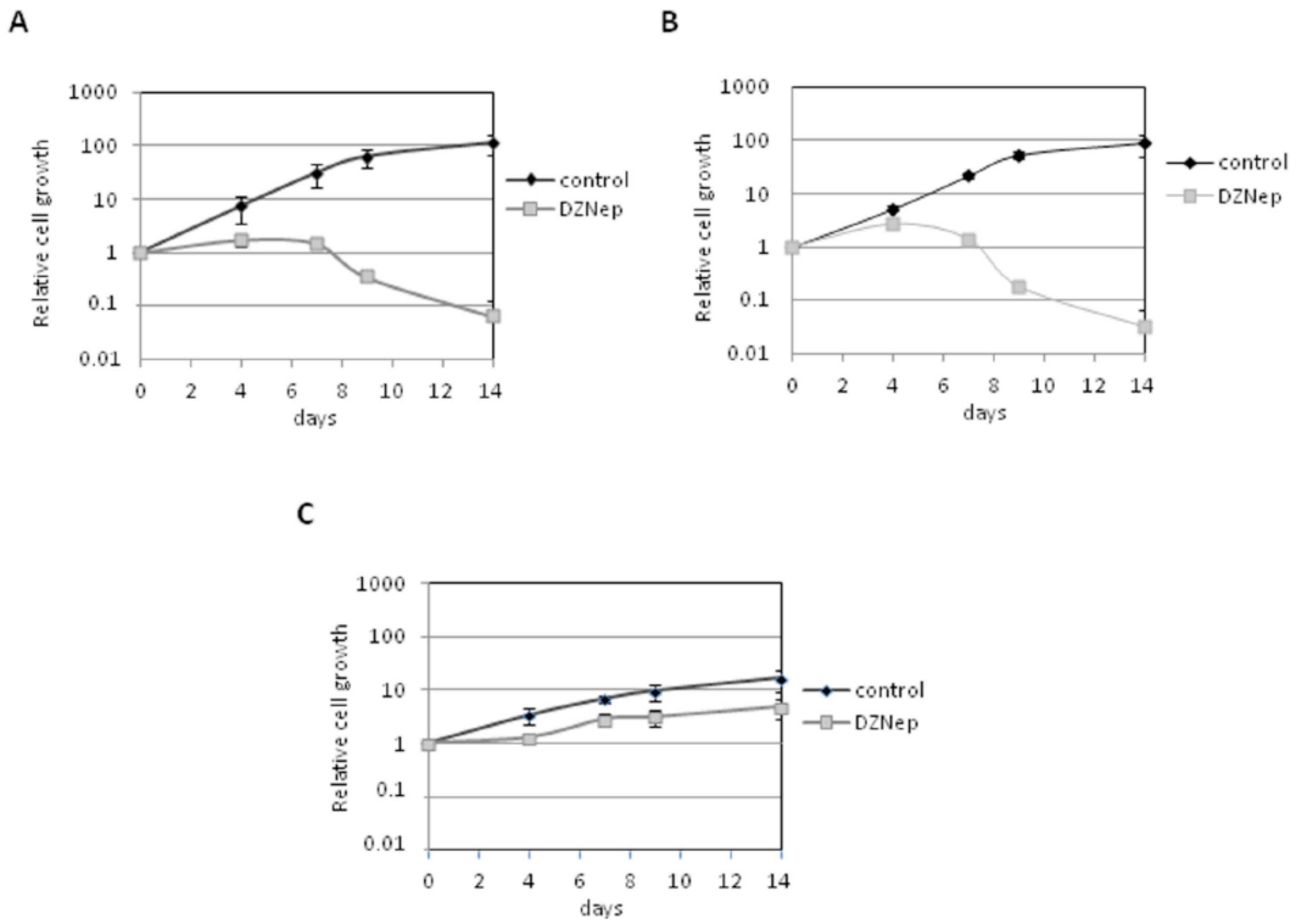


**Figure 3**. DZNep induces death in chondrosarcoma but not chondrocytes. CH2878 cells (A), SW1353 (B), or chondrocytes (C) were treated with DZNep (1 µM) for 14 days.

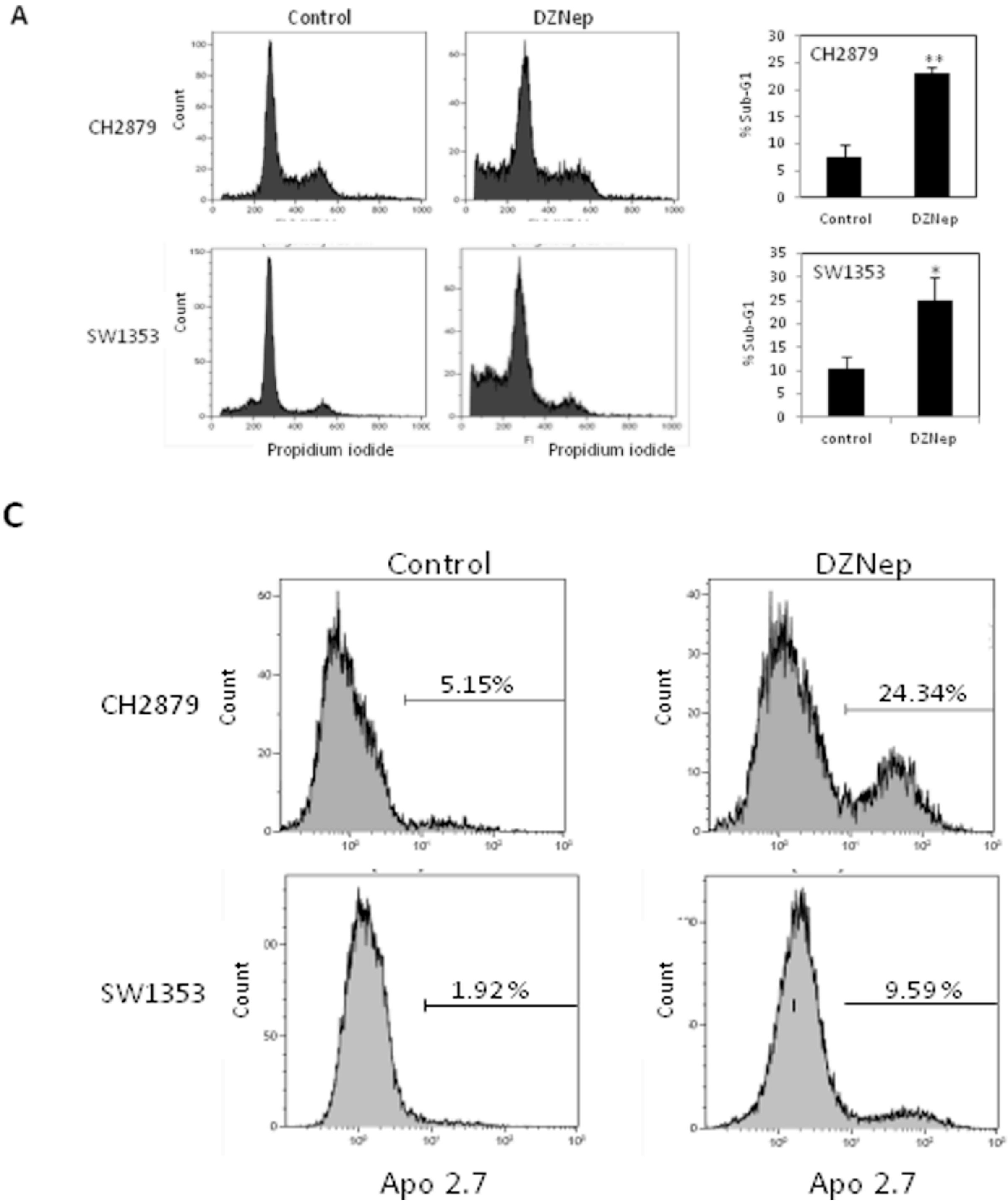


**Figure 4.** DZNep induces apoptosis in SW1353 and CH2879 chondrosarcoma cell lines. Cells were treated with DZNep (1 μ M) for 7 days (A and C) or 5 days (B). A) At day 7, cells were fixed and cell cycle determined by flow cytometry. Histograms represent the sub-G1 phase percentage from three independent experiments. C) At day 7, cells were stained with Apo 2.7 antibody coupled to phycoerythrin and analyzed by flow cytometry

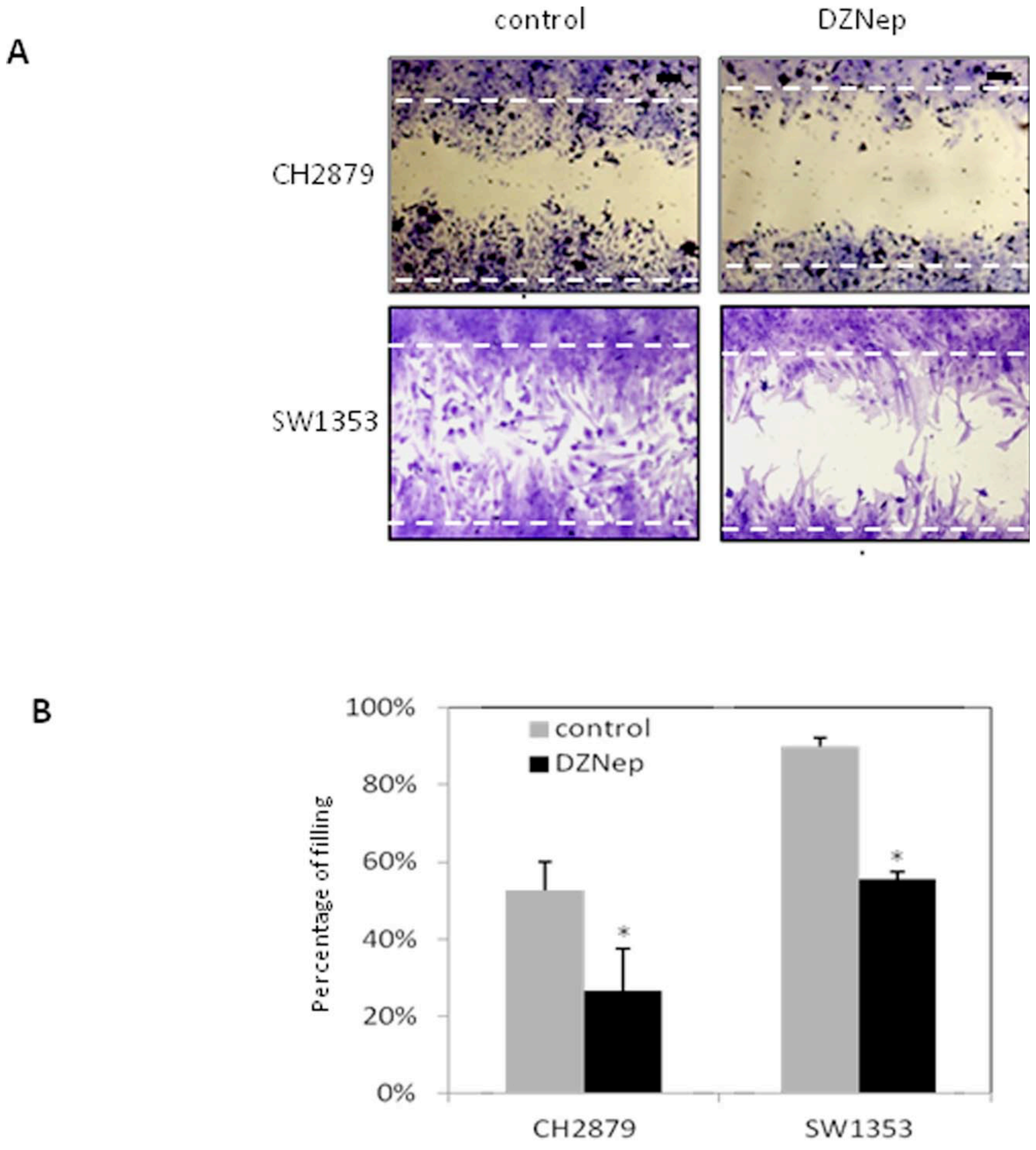


**Figure 5.** DZNep reduces SW1353 and CH2879 chondrosarcoma migration. Cells were pretreated 4 days with DZNep (1 µM) and a straight scratch was made in individual 6-wells dishes with a 200 mL pipette tip. A) Microscopic observations were recorded 24 hours after scratching the cell surface. Dotted lines showed the initial mark of the scratch. B) Graph represents the percentage of filling 24 hours after the wound.

## 6.9 COLON CANCER-1 (IN VITRO ANTICANCER ACTIVITY)

Sha M et al., DZNep inhibits the proliferation of colon cancer HCT116 cells by inducing senescence and apoptosis. Acta Pharmaceutica Sinica B. 2015;5:(3):188–193.

In this study, the authors demonstrate that DZNep can inhibit the growth and survival of colon cancer HCT116 cells by inducing cellular senescence and apoptosis. The study provides a novel view of anti-cancer mechanisms of DZNep in human colon cancer cells.

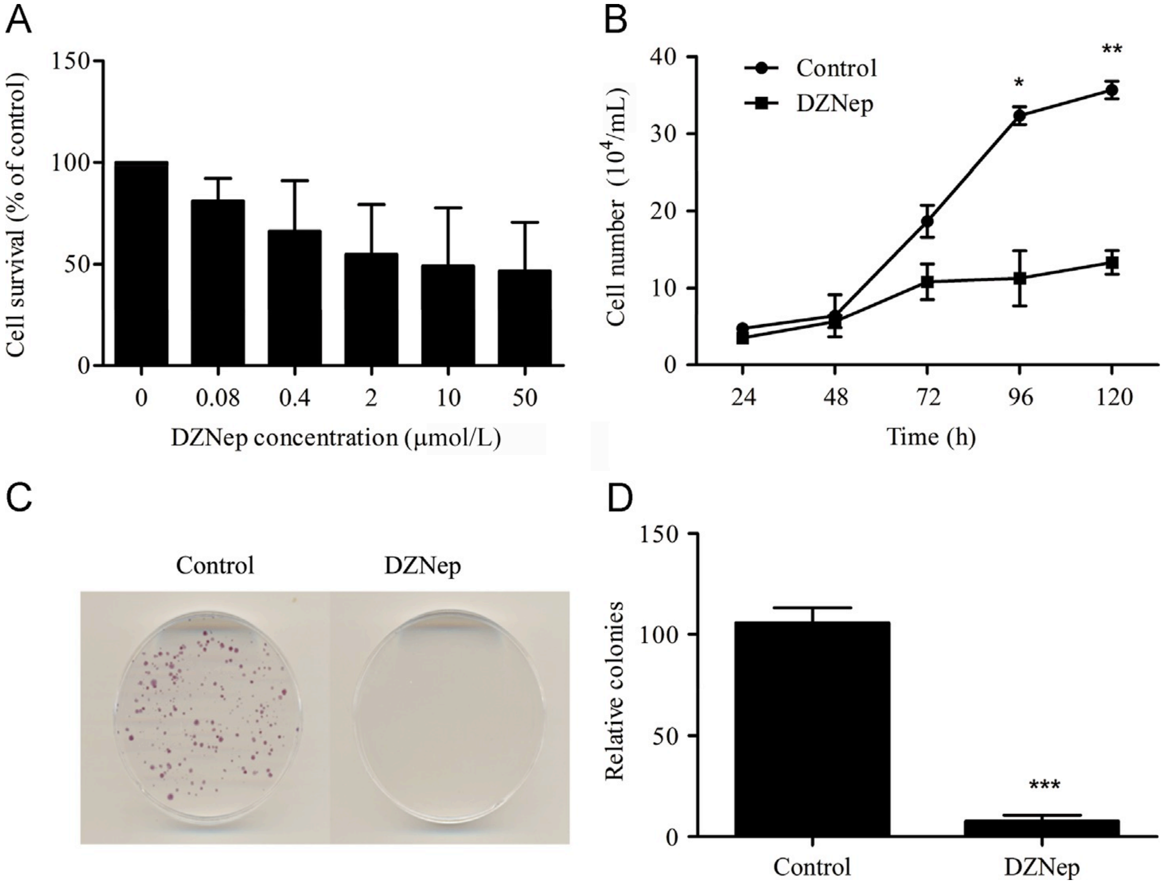


**Figure 1**. DZNep inhibited the proliferation of HCT116 cells. (**A**) Effect of DZNep on the proliferation of HCT116 determined by MTT assay. The cells were treated in 96-well plates for 48h with DZNep at the indicated concentration. (**B**) Effect of DZNep on the growth curve. The cells were seeded, into 24-well plates with $1x10^4$ cells/well for2 4h, and then treated with or without 5 μmol/L DZNep and counted at indicated points (*Po0.05, **P = 0.01 vs. control, n = 3). (**C**) Colony formation upon DZNep treatment. The cells were seeded in 35 mm dishes with 500 cells per dish, and then treated with 5 μmol/L DZNep for 2 weeks. (**D**) The percentage of the colonies was normalized relative to the control (***Po0.001 vs. control, n = 3).

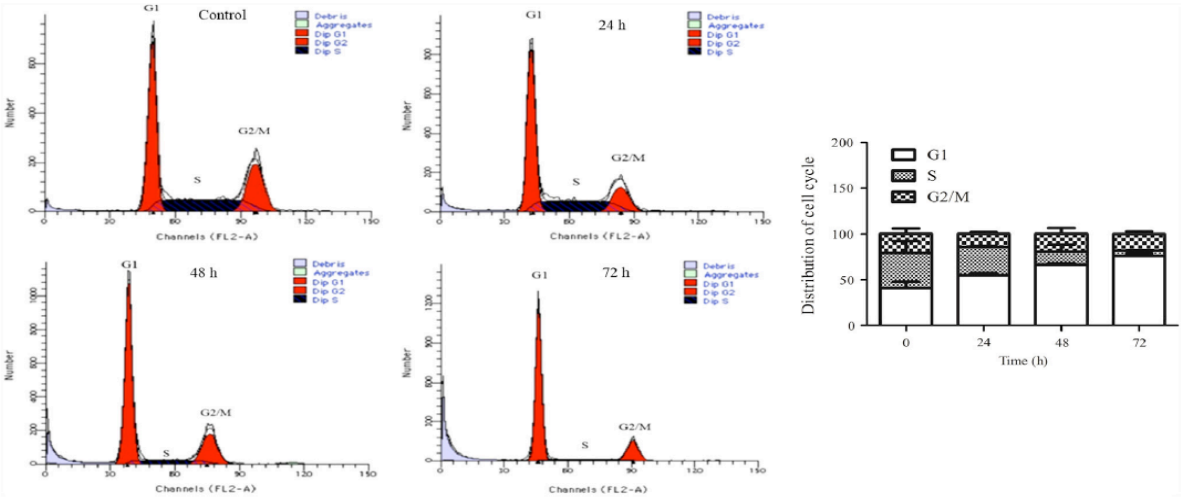


**Figure 2** DZNep induced cell cycle arrest in HCT116 cells. The cells were treated with 5 μmol/L DZNep for the indicated time before being subjected to PI staining and FACS analysis as described in Section 2.

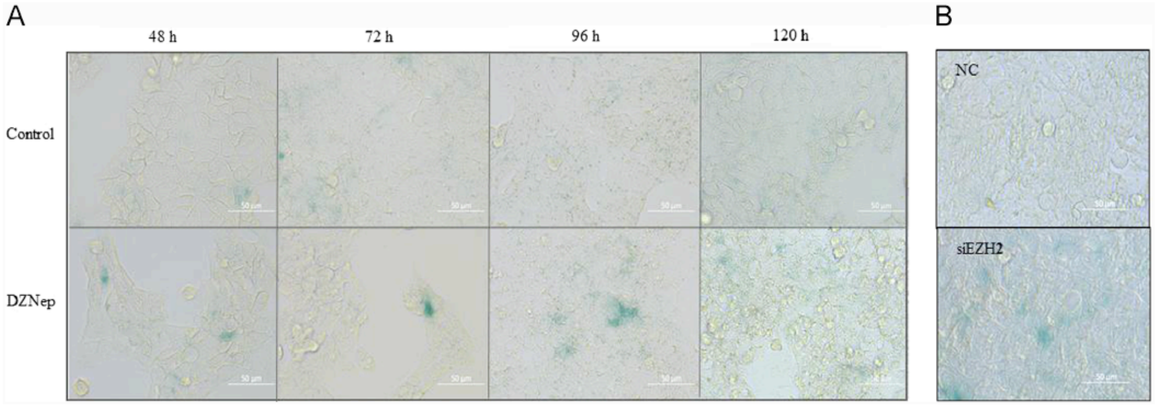


**Figure 3** Inhibition the expression of EZH2 induced cellular senescence in HCT116 cells. The cells were treated with 5 μmol/L DZNep (A) or with siRNA (B) for the indicated time, and senescence was analyzed with SA-*β*-gal staining and photographed (40 ×). Scale bar: 50 μm.

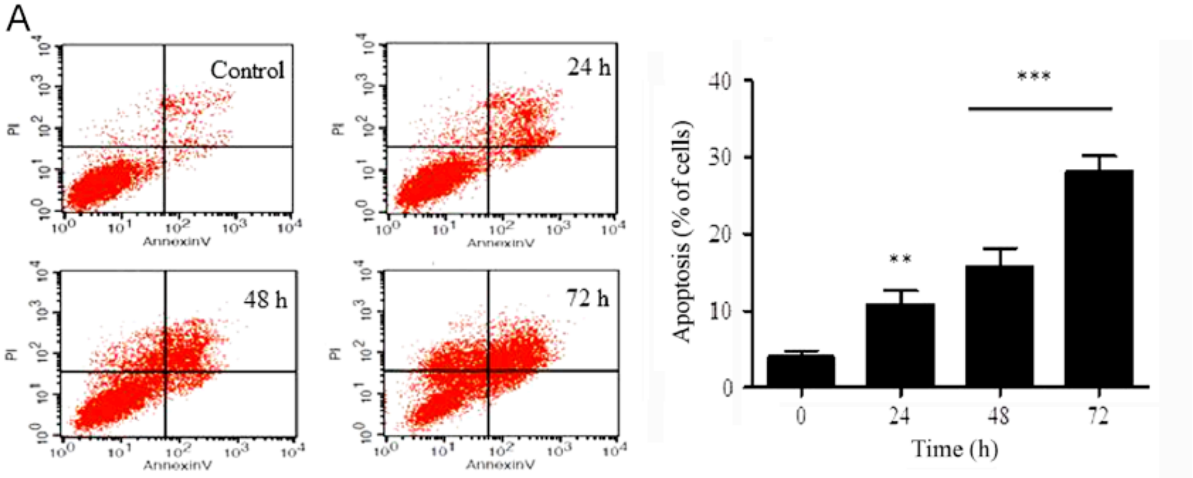


**Figure 5** DZNep induced apoptosis in HCT116 cells. (A) Apoptosis induced by DZNep was detected by AnnexinV/PI staining. The cells were seeded in 6-well plates and treated with 5 μmol/L DZNep for the indicated time, and then subjected to FACS analysis to determine the percentage of early apoptosis cells (lower right quadrant) and late apoptosis cells (upper right quadrant) ($^{**}P<0.01$, $^{***}P<0.001$ *vs.* control). (B) PARP

## 6.10 COLON CANCER-2 (IN VITRO ANTICANCER ACTIVITY)

Benoit YD et al., Inhibitors of enhancer of zeste homolog 2 (EZH2) activate tumor-suppressor microRNAs in human cancer cells. Exp Cancer Res 2013;319:1463-1470.

Recent studies reported the presence of cancer stem cell sub-populations within human colon cancer cell lines, such as HT29. In these cases, cell sorting experiments based on high expression of CCSC markers, including CD133 and CD44,led to an enrichment in drug resistant, highly tumorigenic cells expressing stem cell pluripotency factors, such as SOX2. We showed by indirect immunofluorescence staining the presence of SOX2 high-expressing ($SOX2^{Hi}$) cells in HT29 monolayers, at an average abundance of 0.66% (**Fig. 1A**). DZNep treatment of HT29 cells in monolayer culture caused a decrease in this proportion of $SOX2^{Hi}$ cells to 0.19% (**Fig. 1A**).

In order to assess apoptosis incidence in the sub-population of $SOX2^{Hi}$ cells present in HT29 cultures following PRC2 HMTase by DZNep inhibition, the investigators used SOX2 immunofluorescence and TUNEL co-labeling experiments. They observed an increased rate of apoptosis in $SOX2^{Hi}$ cells in DZNep treated cells (74.7%) compared to controls (11.1%) (**Fig. 1B**).

By using xenograft tumor formation assays in NOD/SCID mice, the investigators observed that HT29 treated in vitro with DZNep formed smaller tumors (~50%) than the control group, suggesting a reduced in vivo tumorigenic potential (**Fig. 1C**). These results suggest a potential role for the polycomb group proteins in the regulation of CCSC survival.

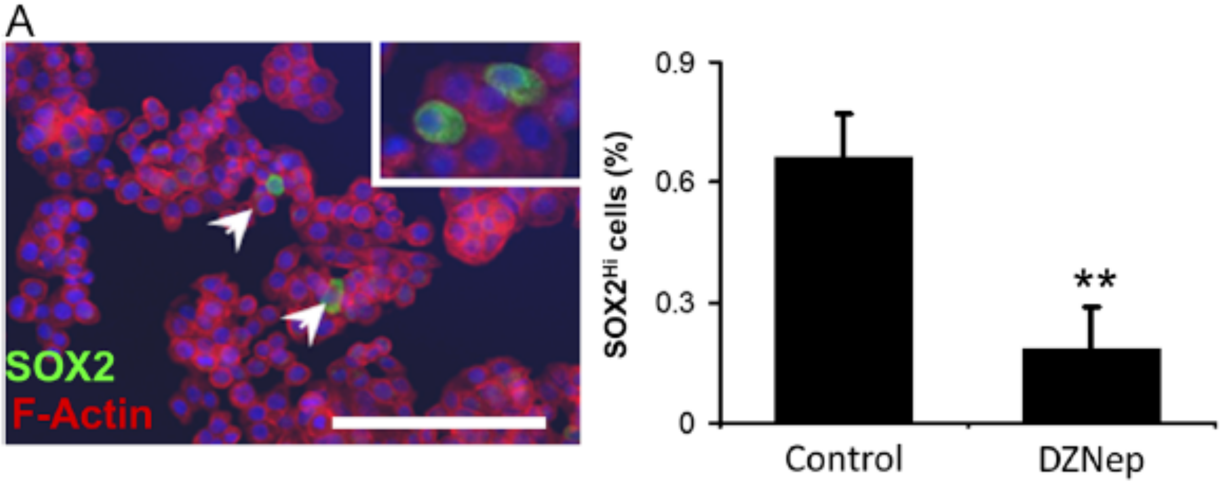


**Fig. 1A –** Effect of DZNep on the HT29SOX2Hi sub-population. Immunofluorescence staining of SOX2 (green) was performed to highlight a cancer stem cell subpopulation in the human colon cancer cell line HT29 (left panel)(bar=50 µm). Percentages (%) of $SOX2^{Hi}$ cells present in control (vehicle) and DZNep treated (5 µM, 48h) HT29 cultures are represented in the histogram (n=7).

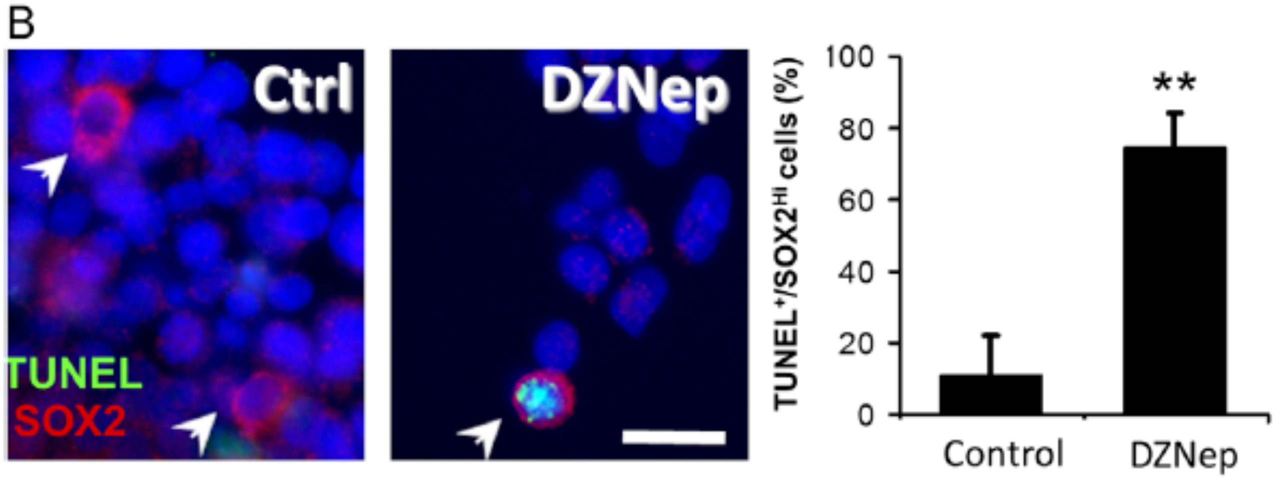


**Fig. 1B –** SOX2 (red) and TUNEL (green) co-labeling was performed on control (vehicle) and DZNep treated (5 µM, 24h) HT29 cultures (bar=10 µm). The histogram shows the percentages of $SOX2^{Hi}$ cells that are TUNEL-positive in control and DZNep treated HT29 cultures (n=5).

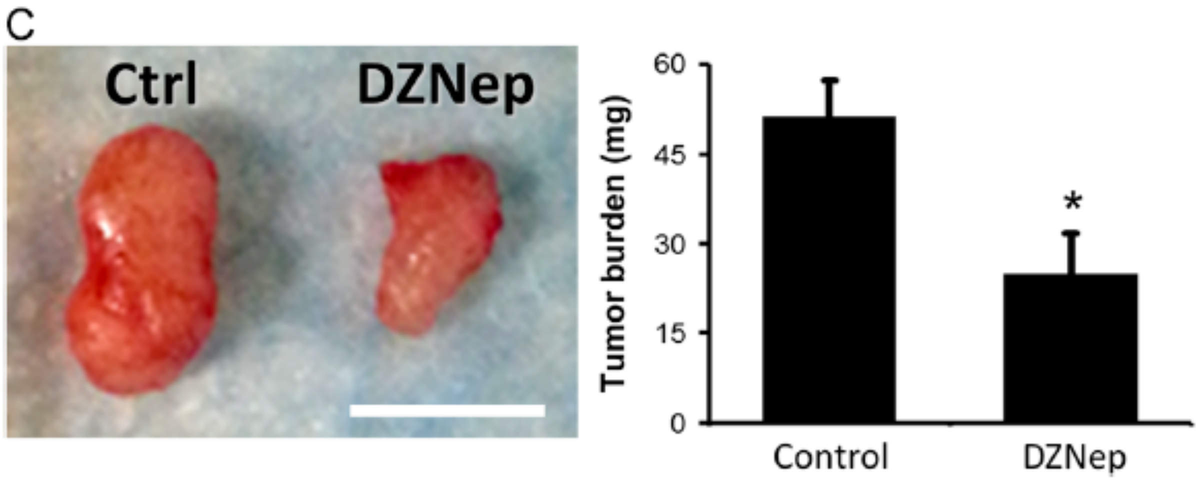


**Fig. 1C –** Representative picture of 21 days post-injection tumor xenografts from NOD/SCID mice injected with control (vehicle) and DZNep treated in vitro (5 µM) HT29 cells (bar=3 mm). Tumor burden measurements are represented as a histogram (n=6).

## 6.11 COLON CANCER-3 (IN VITRO ANTICANCER ACTIVITY)

Yao Y et al., Downregulation of Enhancer of Zeste Homolog 2 (EZH2) is essential for the Induction of Autophagy and Apoptosis in Colorectal Cancer Cells. Genes (Basel). 2016;7(10):E83.

These investigators reported previously that the expression level of EZH2 was higher in colorectal cancer (CRC) tumor tissues than in normal tissues. The protein expression of EZH2 in the CRC cell lines RKO and HCT116 treated with DZNep (3 µmol/L) for 72 was reduced as shown by Western blot analysis (**Figure 3e,f**).

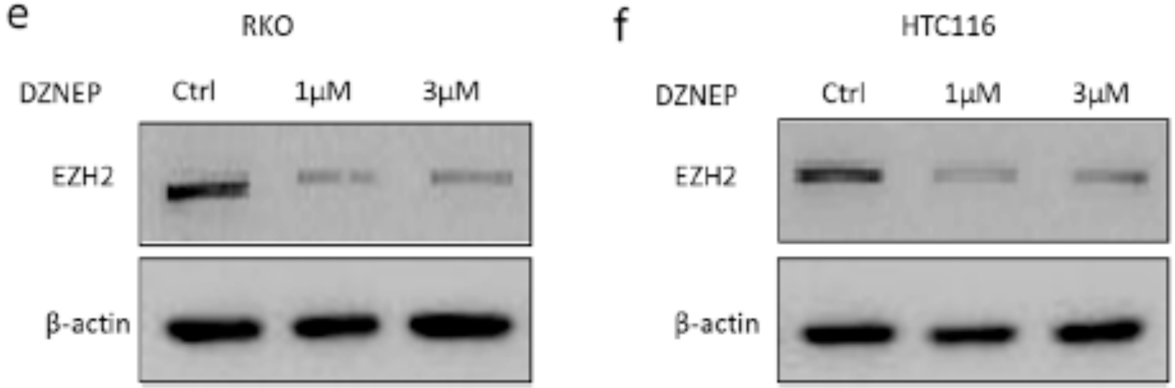


**Figure 3.** The reduction of EZH2 protein expression by DZNep.
RKO and HCT116 cells were treated with DZNep (1 or 3 µmol/L) for 72 h. EZH2 protein expression was analyzed by Western blot in RKO (**e**) and HCT116 (**f**) cells.

Colony formation by RKO and HCT116 cells treated with DZNep (3 µmol/L) for 72 h was reduced.

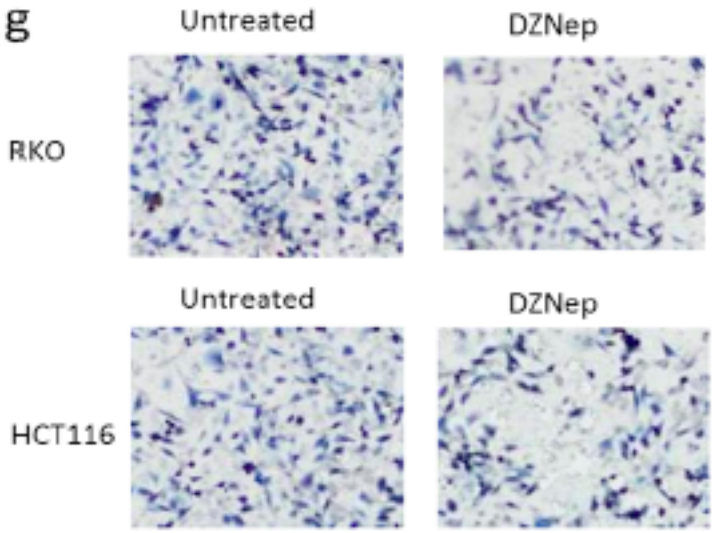


**Figure 4g.** Reduction in colony formation by RKO and HCT116 cells by DZNep.
RKO and HCT116 cells were treated with DZNep (3 µmol/L) for 72 h. Cell colonies were stained with 0.1% crystal violet and the colonies contained were counted manually under a microscope.

DZNep treatment of RKO and HCT116 cells induced autophagy as indicated by the autophagy markers, LC3-II and AmbraI.

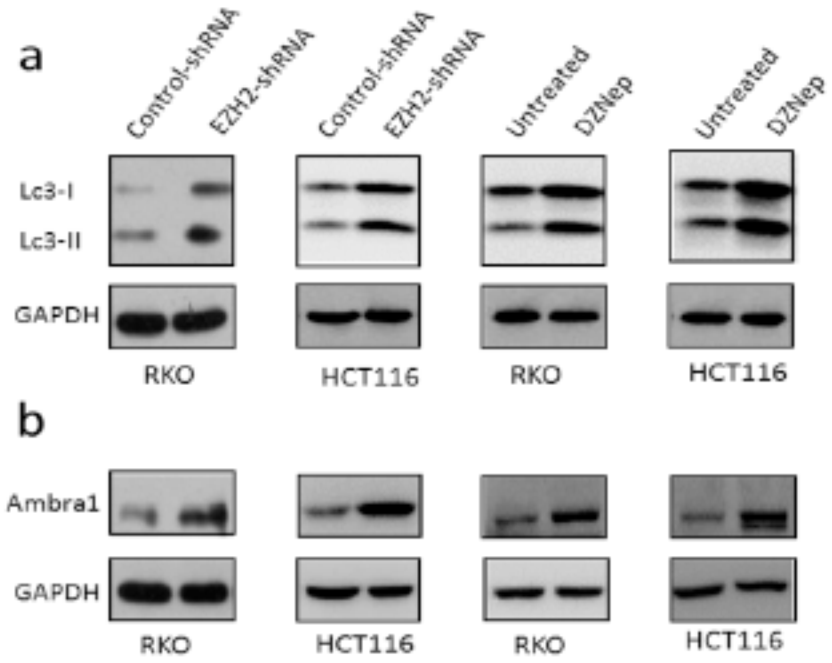


**Figure 5.** Depletion of EZH2 by specific shRNA or DZNep induced autophagy in RKO and HCT116 cells. RKO and HCT116 cells were transfected with control-shRNA or EZH2-shRNA or treated with DZNep (3 µmol/L) for 72 h. Western blot for LC3-II (a) or Ambral1 (b) protein levels are shown. The expression of LC3-II and/or Ambral1 are positive markers for autophagy.

DZNep treatment of RKO and HCT116 cells induced apoptosis as shown by staining with Annexin V and PI.

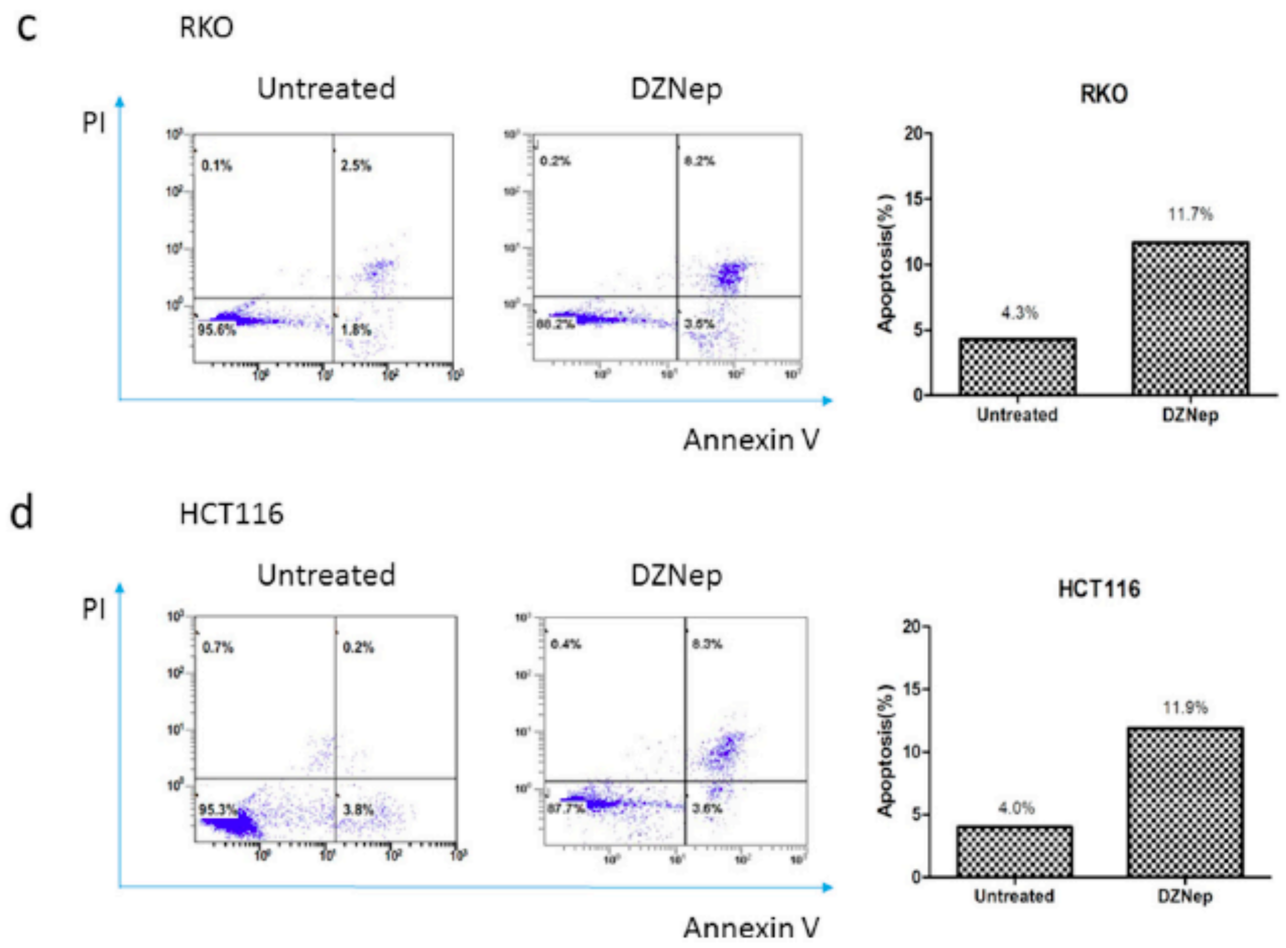


**Figure 6.** EZH2-shRNA or DZNep alters apoptosis rate in RKO and HCT116 cells.
Cells were transfected with EZH2-shRNA or control EZH2-shRNA (a,b) or treated with 3 µmol/L DZNep and at 72 h the cells were stained with Annexin V and PI for flow cytometer analysis.

## 6.12 GASTRIC CANCER (IN VITRO ANTICANCER ACTIVITY)

Cheng LLet al., TP53 genomic status regulates sensitivity of gastric cancer cells to the histone methylation inhibitor 3-deazaneplanocin A (DZNep). Clin Cancer Res 2012;18:4201-4212.

To explore the in vitro growth response of gastric cancer cell lines to 3-Deazaneplanocin A (DZNep), the lines were treated with increasing concentrations of DZNep from 0.5 mmol/L to 100 mmol/L, and determined $GI_{50}$, (drug concentration required for 50% growth inhibition after 72 hours treatment. DZNep caused very distinct growth responses: IM95, YCC6, AZ521, and AGS cells showed the highest sensitivity to DZNep ($GI_{50}$ = 0.3–2.0 mmol/L) whereas other lines such as TMK1, YCC7, Ist1, and MKN7 did not exhibit cell growth alterations upon DZNep treatment. The growth inhibition by 5 μM DZNep for different exposure times is shown in Fig1D. TP53 genomic status was significantly associated with DZNep cellular responsiveness, with TP53 wild-type (WT) cell lines being more sensitive ($P < 0.001$).

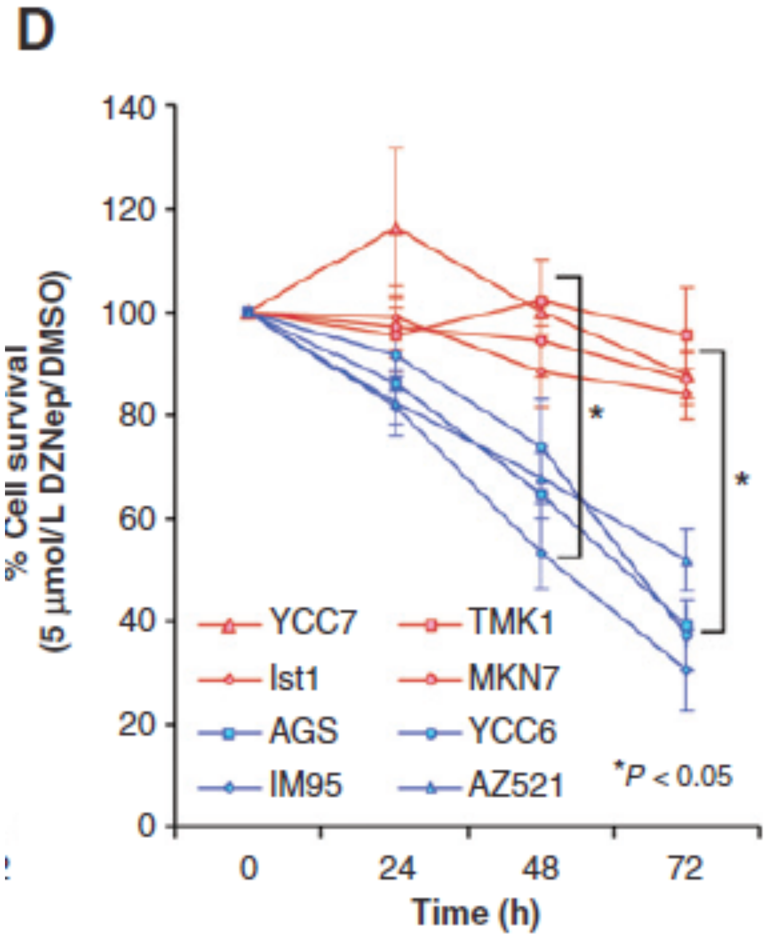


**Figure 1D**. Proliferation effects of DZNep in sensitive (blue) and resistant (red) gastric cancer cell lines. DZNep (5 mmol/L) and control treated cells were monitored for cell survival at 0, 24, 48, and 72 hours. Each point represents the mean ± SD from 3 independent experiments. Differential cell growth responses are observed between sensitive and resistant cells at 48 and 72 hours post-DZNep treatment (* indicates $P < 0.05$, Student t test).

## 6.13 GLIOBLASTOMA (IN VITRO ANTICANCER ACTIVITY)

Suvà M-L et al., EZH2 Is Essential for Glioblastoma Cancer Stem Cell Maintenance. Cancer Res 2009; 69:(24):9211–9218

The authors show that targeted pharmacologic disruption of EZH2 by the S-adenosylhomocysteine hydrolase inhibitor 3- deazaneplanocin A (DZNep), or its specific downregulation by short hairpin RNA (shRNA), strongly impairs glioblastoma (GBM) cancer stem cell (CSC) self-renewal in vitro and tumor-initiating capacity in vivo.

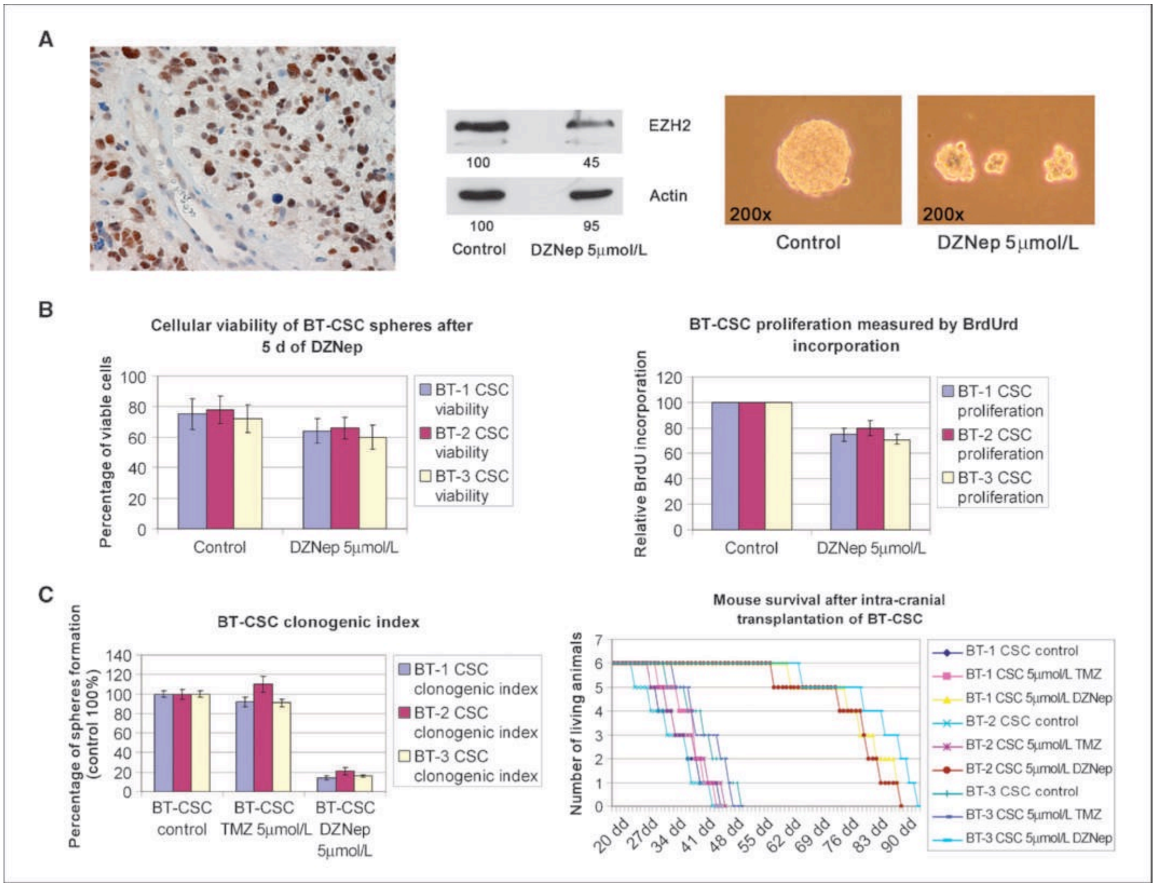


**Figure 19**. Pharmacologic depletion of EZH2 reduces the self-renewal and tumor-initiating properties of GBM CSCs. EZH2 expression in primary human glioblastoma samples (**A, left**). Treatment of GBM CSC spheres with 5 μmol/L of DZNep for 5 d reduces EZH2 protein levels, as assessed by Western blot analysis (**A, middle**) and alters sphere shape (**A, right**). DZNep at 5 μmol/L for 5 d does not significantly impair GBM CSC viability (B, left) or proliferation (**B, right**). GBM CSC treated with 5 μmol/L DZNep or temozolomide (TMZ) were assessed for their CSC properties, namely clonogenic index (**C, left**) and tumor-initiating potential (**C, right** demonstrating that DZNep, but not temozolomide, treatment efficiently decreases the clonogenic index of GBM CSC and extends the survival of all the animals injected with the corresponding cells.

## 6.14 HEPATOCELLULAR CARCINOMA CELLS (IN VITRO ANTICANCER ACTIVITY)

Chiba T et al. 3-Deazaneplanocin A is a promising therapeutic agent for the eradication of tumor-initiating hepatocellular carcinoma cells. Int J Cancer. 2012;130:2557-67.

3-Deazaneplanocin A (DZNep) treatment impaired cell growth and anchorage-independent sphere formation of hepatocellular carcinoma cells (HCC) in culture.

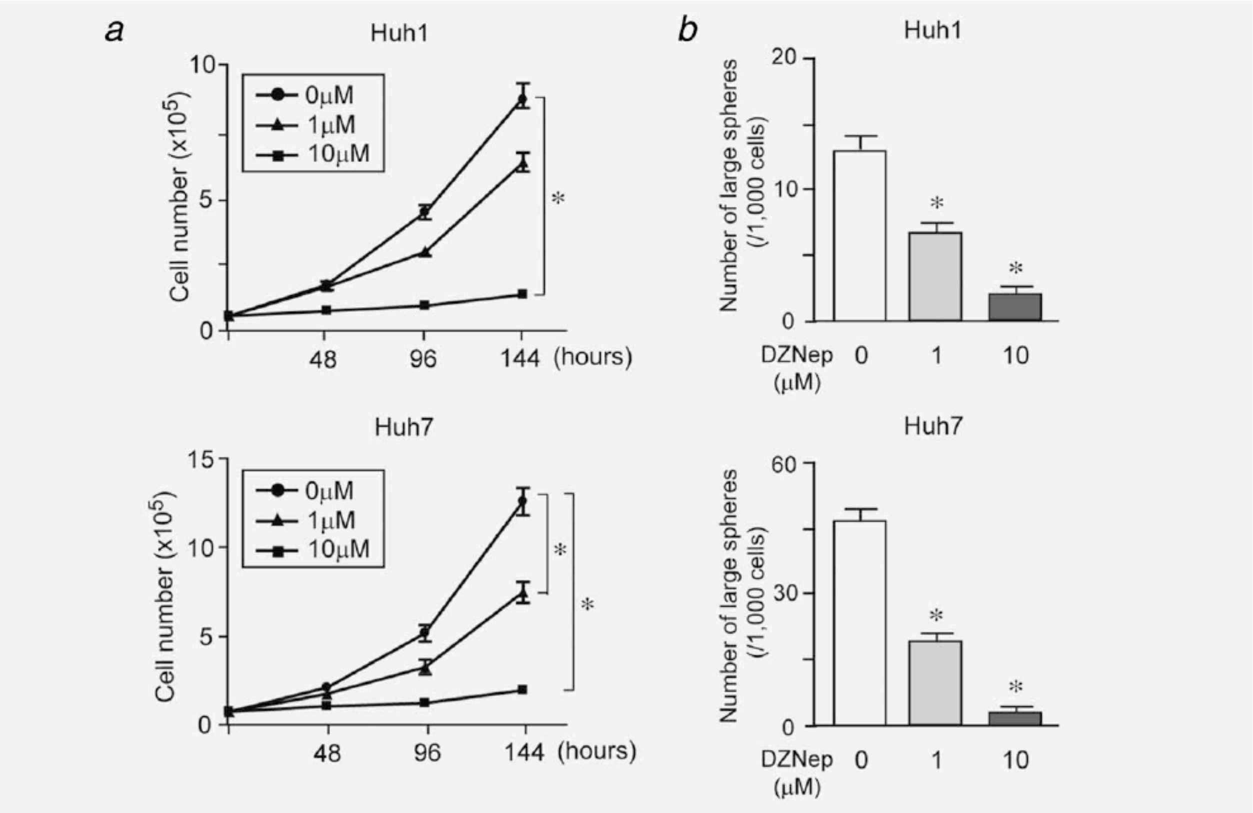


**Figure 4.** In vitro assays of HCC cells treated with DZNep. (a) Dose-dependent inhibition of proliferation in DZNep-treated HCC cells. *Statistically significant ($p < 0.05$). (b) Number of large spheres generated from 1,000 HCC cells at day 14 of culture. *Statistically significant ($p < 0.05$).

Flow cytometric analyses revealed that DZNep treatment decreased the number of epithelial cell adhesion molecule (EpCAM)(+) tumor-initiating cells. These results suggest that DZNep inhibits self-renewal of tumor initiating HCC cells.

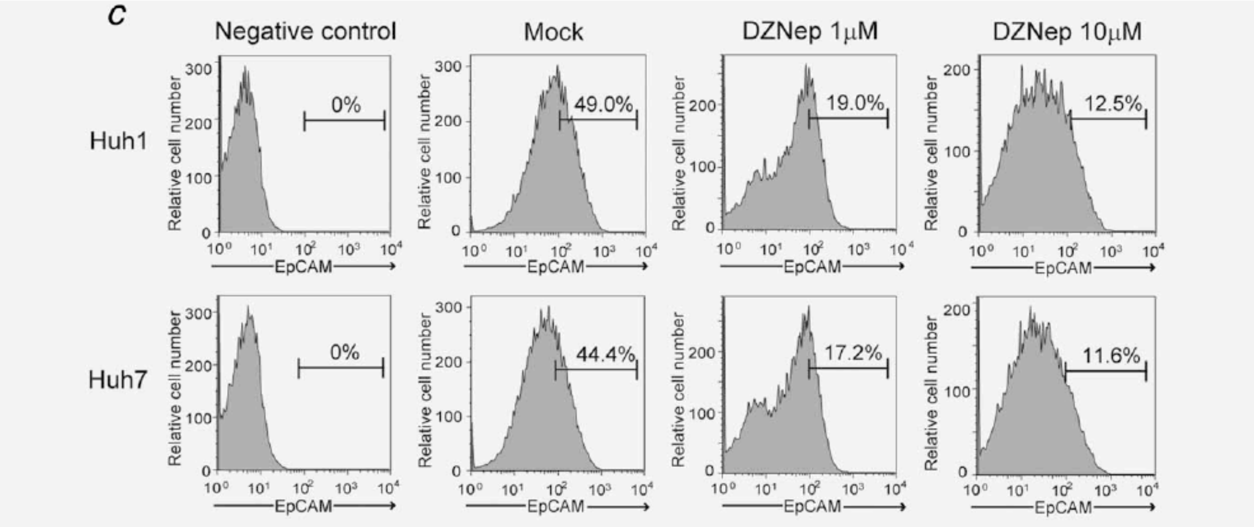


**Figure 4C.** Flow cytometric profiles of HCC cells treated with DZNep (1 or 10 μM) for 144 hr. The percentages of EpCAM high fraction are shown as the mean values for three independent analyses.

Induction of differentiation and apoptosis by DZNep in HCC cells.

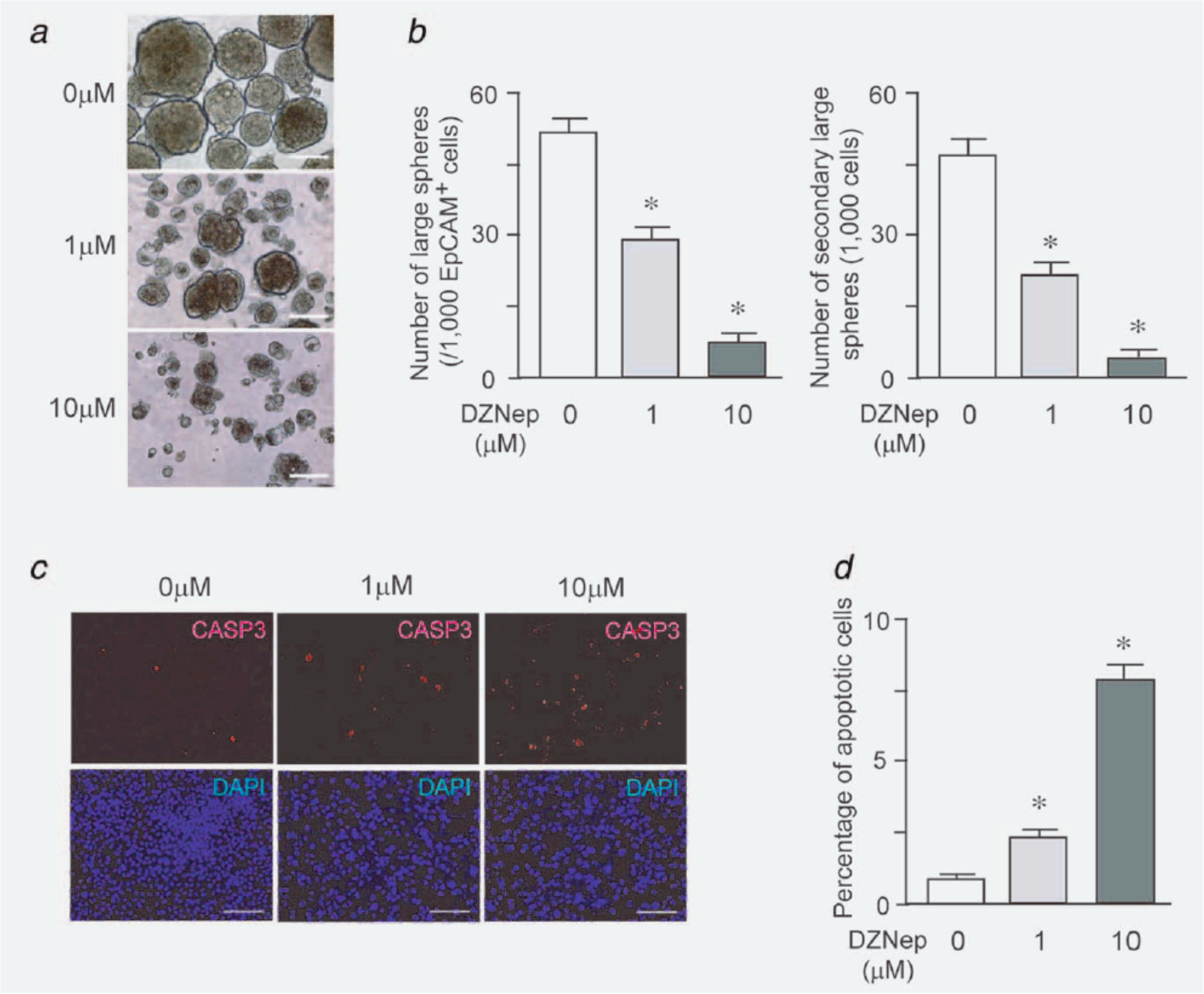


**Figure 5**. Effect of DZNep in tumor-initiating EpCAMþ cells. (a) Bright-field images of non-adherent spheres on day 14 of culture. Scale bar = 100 μm. (b) Number of original spheres generated from 1,000 EpCAMþ cells at day 14 of culture and secondary spheres 14 days after replating. *Statistically significant ($p < 0.05$). (c) Detection of apoptotic cell death by immunostaining of active caspase-3 (CASP3). Scale bar = 200 μm. (d) Quantification of the percentage of apoptotic cells is indicated at the right. *Statistically significant ($p < 0.05$).

### 6.15 LUNG CANCER-1 (IN VITRO ANTICANCER ACTIVITY)

Kikuchi J et al., Epigenetic therapy with 3-deazaneplanocin A, an inhibitor of the histone methyltransferase EZH2, inhibits growth of non-small cell lung cancer cells. Lung cancer .2012;78 (2):138-143.

The aim of this study was to determine the effect of DZNep in NSCLC cells (NCI-H1299, NCI-H1975, and A549). MTT assays demonstrated that DZNep treatment resulted in dose-dependent inhibition of proliferation in the NSCLC cell lines with a half maximal inhibitory concentration (IC50) ranging from 0.08 to 0.24 μM (**Table 6**). Immortalized, but non-cancerous, bronchial epithelial and fibroblast cell lines (HBEC3 KT, 16HBE14o-bronchial epithelial cell line, WI-38 VA-13 2RA fibroblast cell line) were less sensitive to DZNep than the NSCLC cell lines. Soft agar assays demonstrated that anchorage-independent growth was also reduced in all three NSCLC cell lines by DZNep (**Fig 2**). Flow cytometry analysis demonstrated that DZNep induced apoptosis and G1 cell cycle arrest in NSCLC cells (**Fig 3**). DZNep may constitute a novel approach to treatment of NSCLCs.

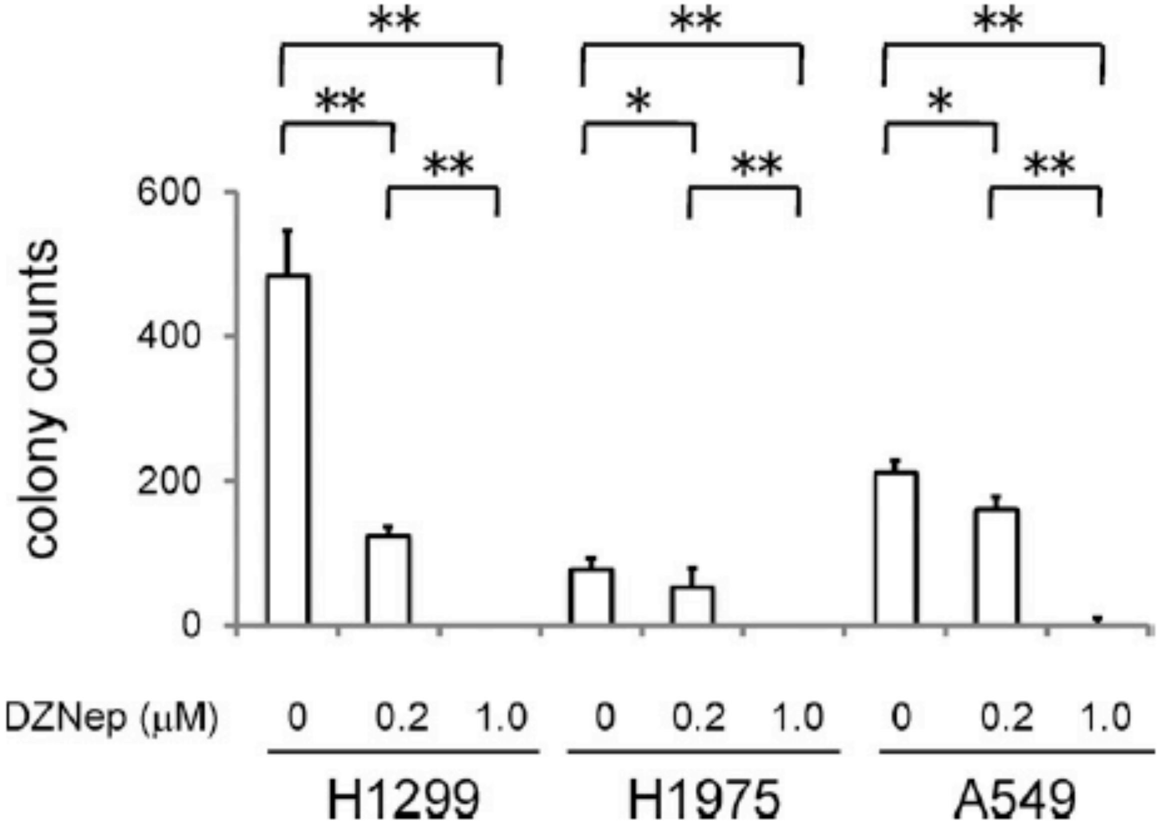


**Fig. 2.** Inhibition of anchorage-independent growth by DZNep. Representative data from one of three independent experiments is shown. Data are means ± SD of triplicate samples. Similar results were obtained from all three independent experiments. *$p < 0.05$ and **$p < 0.01$ between indicated groups by one-way ANOVA with Tukey's multiple comparison test.

**Table 6** : $IC_{50}$ of DZNep

| Cell types | Cell line | $IC_{50}$ of DZNep (μM) |
|---|---|---|
| NSLC cells | H1299 | 0.21 ± 0.01 |
| | H1975 | 0.08 ± 0.02 |
| | A549 | 0.24 ± 0.01 |
| | PC-3 | 0.19 ± 0.01 |
| Non cancer cells | HBEC3 KT | 0.58 ± 0.09** |
| | 16HBE14o- | 1.03 ± 0.11** |
| | WI-38 VA-13 2RA | 0.63 ± 0.07** |

Data are presented as mean ± SD of three independent experiments.
**$p < 0.001$ compared with each NSCLC cell line by one-way ANOVA with Tukey's multiple comparison test.

a. **DZNep induced cell-cycle arrest and apoptosis**

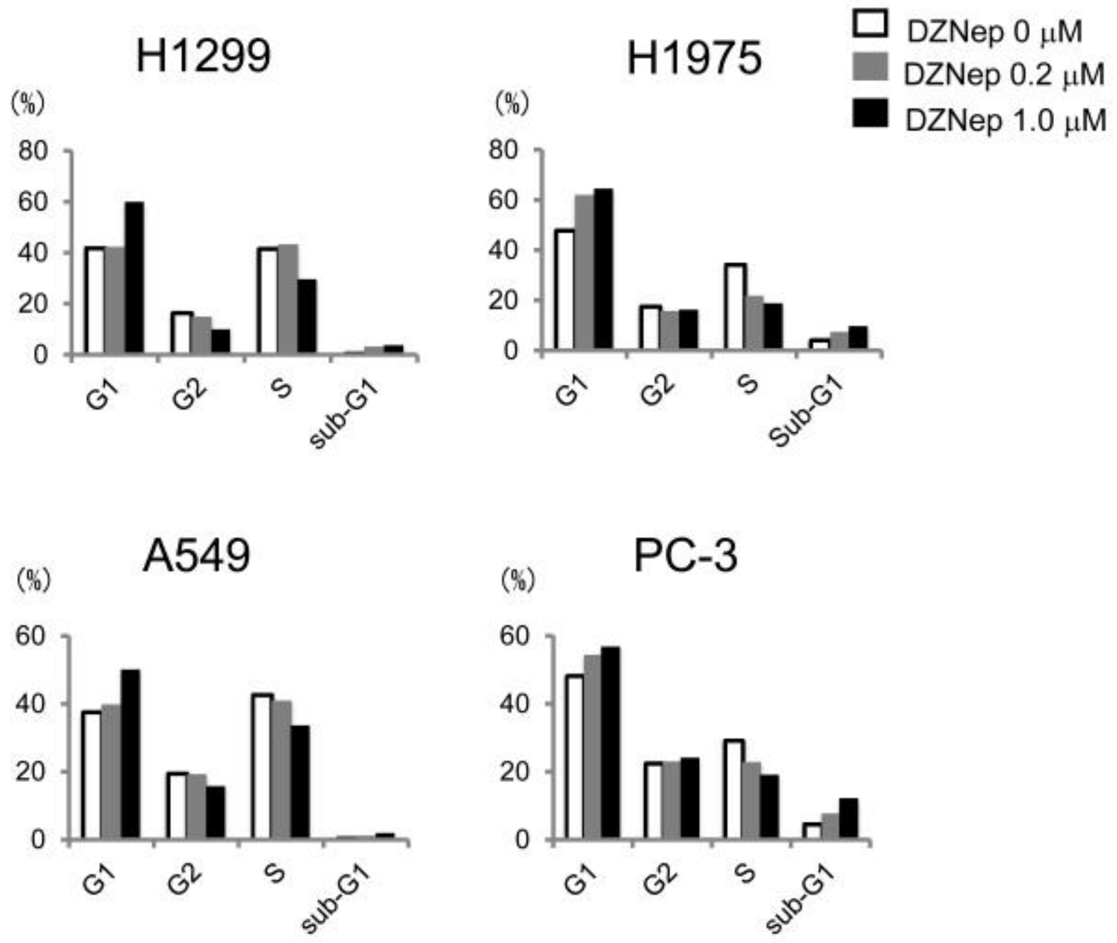


**Figure 3**. Effect of DZNep on cell cycle in NSCLC cells. Cells were transfected with indicated doses of DZNep. After 72 hrs, the percentage of cells in each cell cycle phase was measured using a FACS flow cytometer and ModFitLT software. Representative data from one of three independent experiments is shown. Similar results were obtained in all three independent experiments

## 6.16 LUNG CANCER-2 (IN VITRO ANTICANCER ACTIVITY)

Nascimento ASF et al., Synergistic antineoplastic action of 5-aza-2'deoxycytidine (decitabine) in combination with different inhibitors of enhancer of zeste homolog 2 (EZH2) on human lung carcinoma cells. J Cancer Res Ther. 2016, 4(5):42-49.

Human lung carcinoma cells (A549) were treated in vitro for different exposure time with 1 or 2.5 µM concentration of the EZH2 inhibitors: DZNep, Gsk-126 or U-4149 and cell survival determined by colony assay. Under these conditions DZNep exhibited greater antitumor activity than Gsk-126 or U-4149.

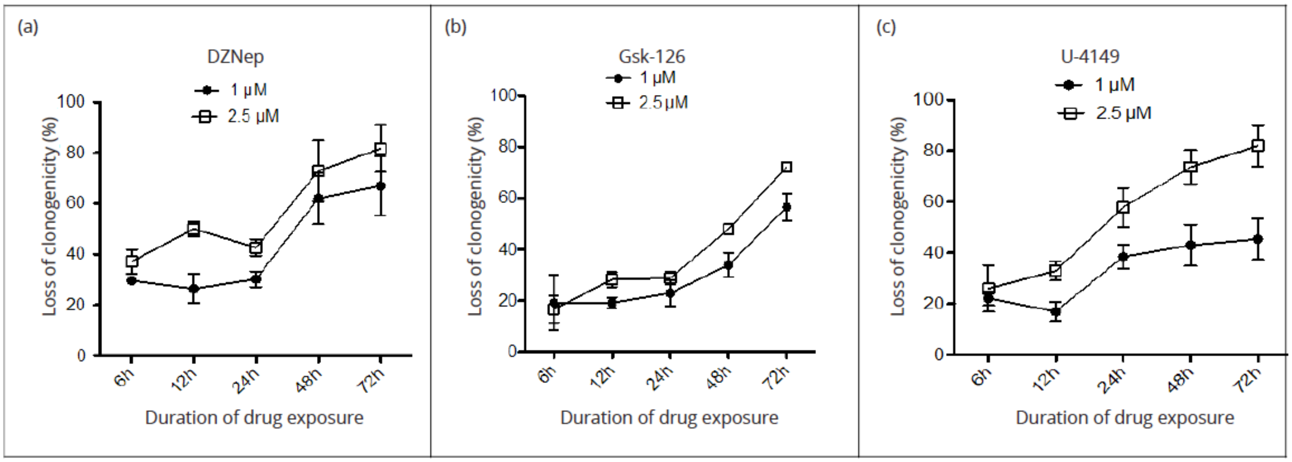


**Figure 2** Time-course evaluation of the antineoplastic activity of DZNep (a), Gsk-126 (b) and U-4149 (c) on A549 cells. The cells were exposed to drugs at indicated concentrations. At different times (6, 12, 24, 48 and 72 h) the drugs were removed and colony formation was determined on day 16-18. Graphs represent the percentage of loss of clonogenicity relative to control cells.

## 6.17 LUNG CANCER-3 (IN VITRO ANTICANCER ACTIVITY)

Fillmore CM et al., EZH2 inhibition sensitizes BRG1 and EGFR mutant lung tumours to TopoII inhibitors. Nature. 2015;520(7546):239-242.

Western Blot showed that 4 days of 1μM DZNep effectively reduced EZH2 protein and H3K27me3, and 10μM GSK126 for 4 days caused decrease in H3K27me3 levels yet EZH2 remained unchanged. HCC15, A549, H157 and PC9, termed 'sensitized' cell lines, had lower etoposide $IC_{50}$ when EZH2 was inhibited by DZNep or GSK126. Conversely, H460, H23 and Sw1573 cell lines, termed 'protected' lines, had higher etoposide $IC_{50}$.

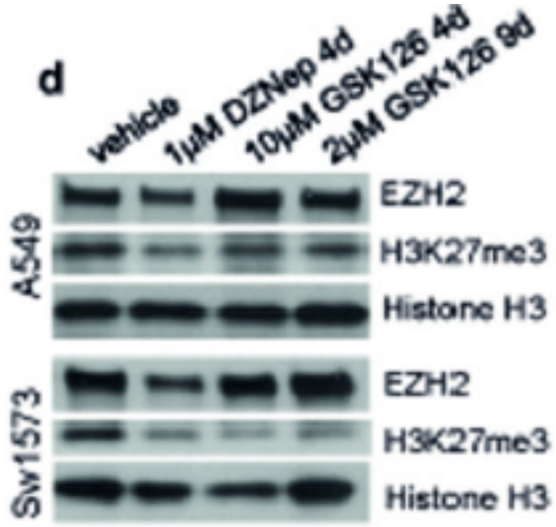


**Figure 1d** Western Blot for EZH2 and H3K27me3 on NSCLC cell lines treated with DZNep or GSK126 for 4 days.

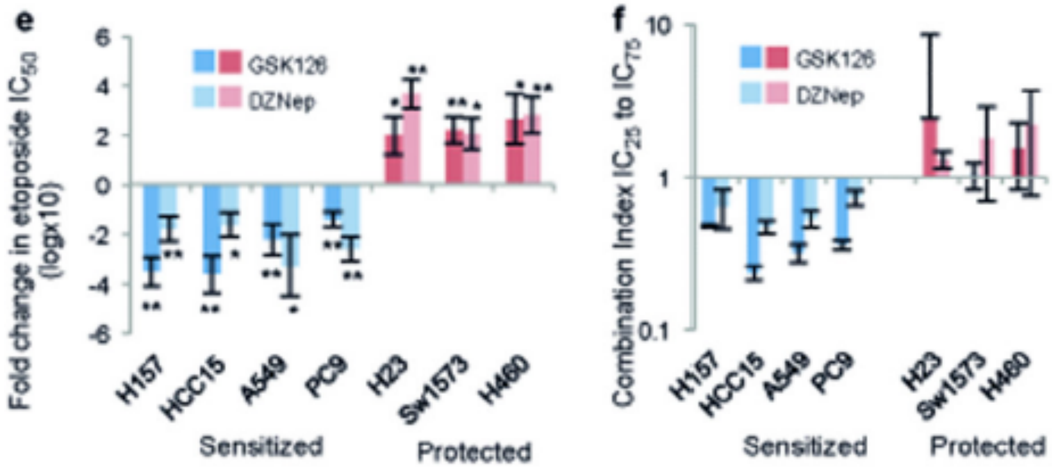


**Figure 1e**, Fold change +/− s.e.m. in etoposide $IC_{50}$ between vehicle treated and DZNep or GEK126 treated NSCLC cell lines for 4 days.

DZNep & GSK126 increased BRAG1 expression to a greater extent in PC9 cells with EGFR mutation than in the wild type cells.

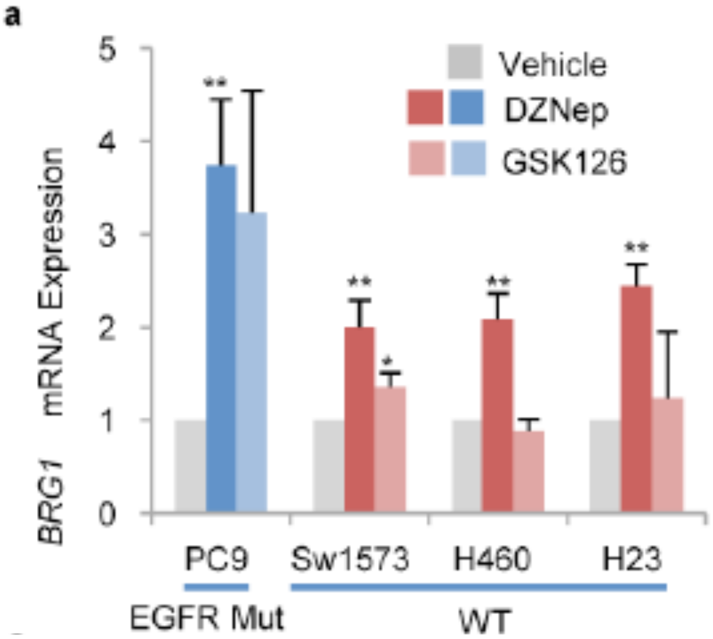


**Figure 3a** mRNA expression analysis by RT-qPCR for BRG1 levels +/− s.e.m. in NSCLC cell lines in response to 4 days of 1µM DZNep or 10µM GSK126, n=6 for DZNep, n=3 for GSK126.

While the protected lines (PC9, H157, A549) showed no difference in apoptotic levels in etoposide compared to dual treated cultures, the sensitized lines (Sw1573, H23) had significantly higher apoptotic fractions in dual treated cultures than in cultures treated with etoposide as a single agent (**Fig. 3c**).

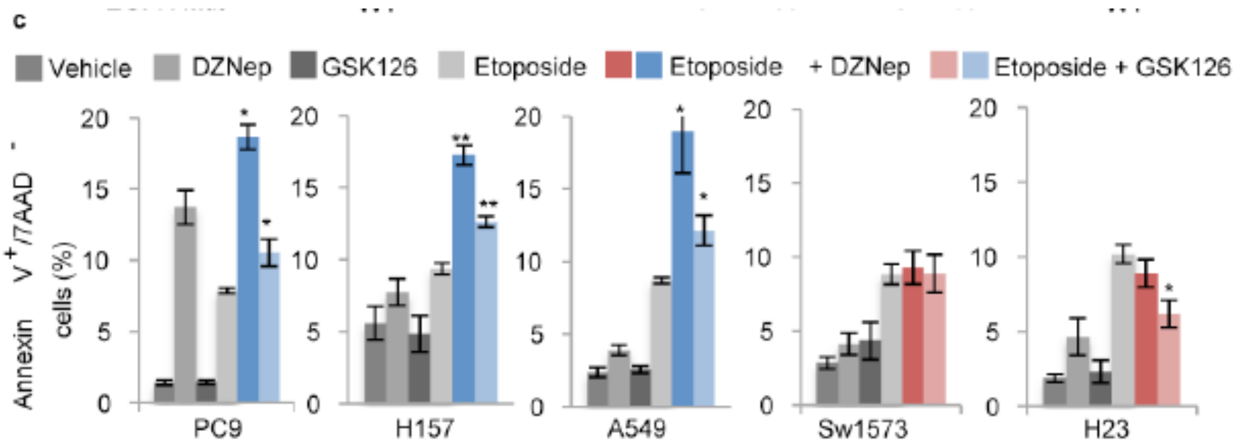


**Figure 3c**, Analysis of apoptosis by Annexin V+/7AAD− cells +/− s.e.m. quantified by flow cytometry on NSCLC cell lines cultured with DZNep, GSK126 +/− Etoposide for 3 days, n=4.

## 6.18 MESOTHIALOMA (IN VITRO ANTICANCER ACTIVITY)

Kemp CD, Rao M, Xi S, Inchauste S, Mani H, Fetsch P, et al. Polycomb repressor complex-2 is a novel target for mesothelioma therapy. Clinical Cancer Res 2012;18:77-90.

Immunoblot experiments revealed that 72-hour DZNep treatment mediated dose-dependent depletion of EZH2, EED, and H3K27Me3 in MES1-2, H28, and H2452 mesothelioma cells (**Fig. 5A**; bottom panel); these effects coincided with significantly decreased proliferation and migration of MPM cells (**Fig. 5A**; top panel, and **Fig. 5B**). Furthermore, DZNep significantly diminished soft agar clonogenicity of MES1 and H2452 cells (**Fig. 5C**).

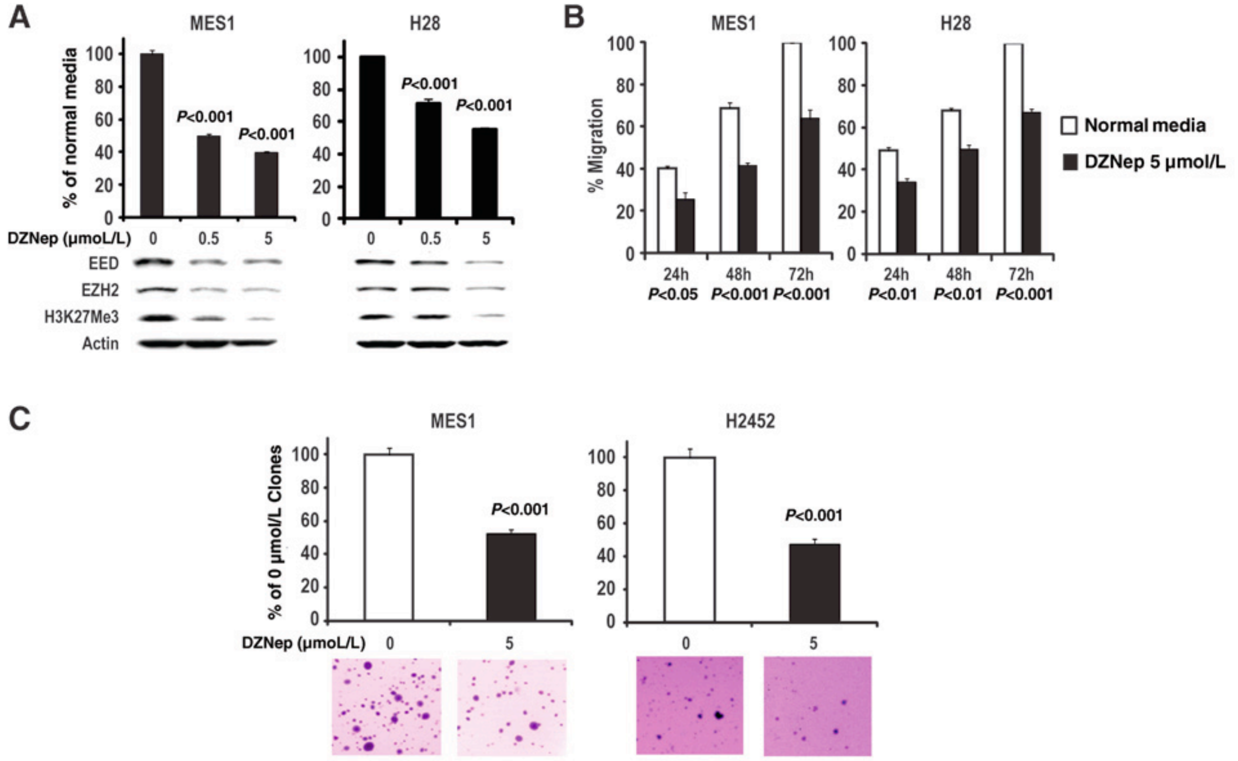


**Figure 5**. Effects of DZNep in MPM cells. **A**, DZNep mediates dose-dependent reductions in EED, EZH2, and global H3K27Me3 levels, which coincide with inhibition of proliferation of MES1 and H28 cells. Similar results were observed for MES2 and H2452 cells. **B**, effects of DZNep on migration of MPM cells. Similar results were observed for MES2 and H2452 cells. **C**, effects of DZNep on soft agar clonogenicity of MES1 and H2452 cells.

## 6.19 MULTIPLE MYELOMA-1 (IN VITRO ANTICANCER ACTIVITY)

Kalushkova A et al., Polycomb target genes are silenced in multiple myeloma. PLoS One. 2010;5(7):e11483.

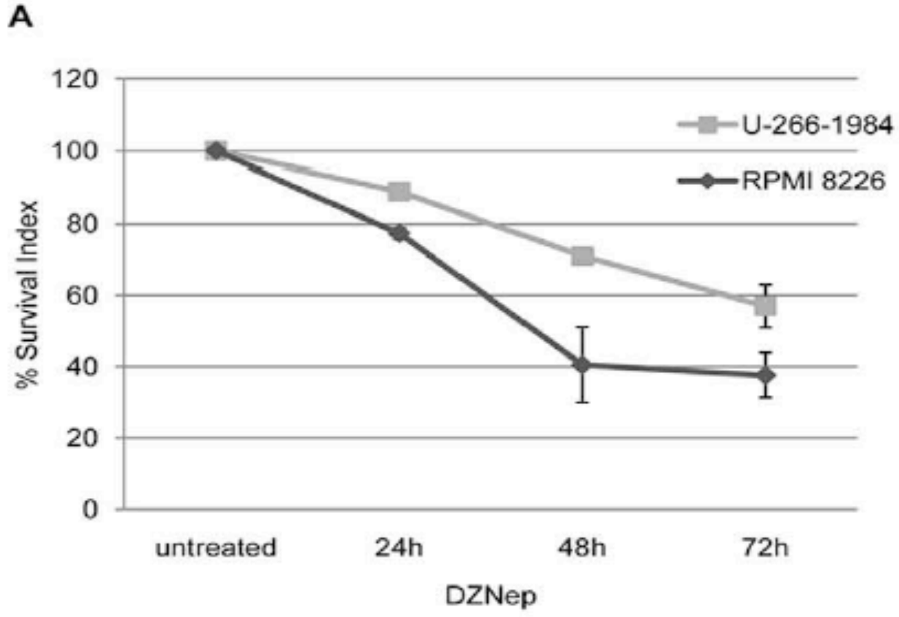


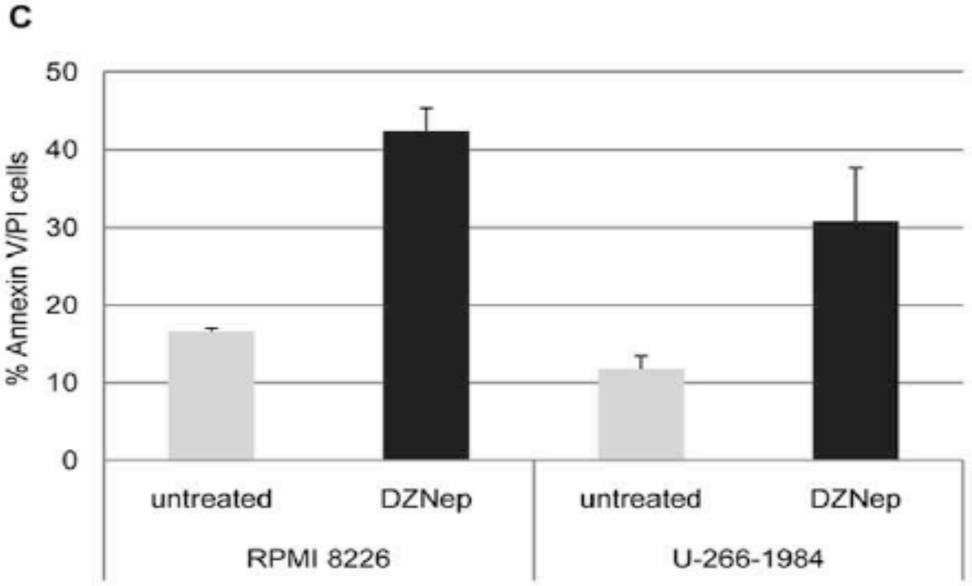


**Figure 5A,C.** DZNep reduce growth and induce apoptosis in MM cell lines. (**A**): U-266-1984 and RPMI 8226 cells were treated for 6, 24, 48 and 72 hours with DZNep (10 µM) followed by a resazurin assay. Data is presented as mean percentage of control. (**C**): U-266-1984 and RPMI 8226 cells were treated with DZNep (10 µM) for 72 hours followed by AV/PI staining and flow cytometry analysis. Data is presented as mean percentage apoptotic cells.

### 6.20 MULTIPLE MYELOMA-2 (IN VITRO ANTICANCER ACTIVITY)

Xie Z et al., Determinants of Sensitivity to DZNep Induced Apoptosis in Multiple Myeloma Cells. PLoS ONE 6(6):e21583.

Mulitple myeloma cell lines were treated with different concentrations of 3-deazaneplanocin A (DZNep), and cell proliferation was measured by MTS colorimetric assay (**Fig. 1A**). NCI-H929 and MM.1S, 2 of the 8 cell lines tested, were very sensitive to DZNep-induced growth inhibition in the nanomolar range. Cell apoptosis induced by DZNep was determined by subdiploid DNA content analysis using flow cytometry. Sub-G1 fraction undergoing apoptosis was significantly increased after DZNep treatment in NCI-H929 and MM.1S cell lines.

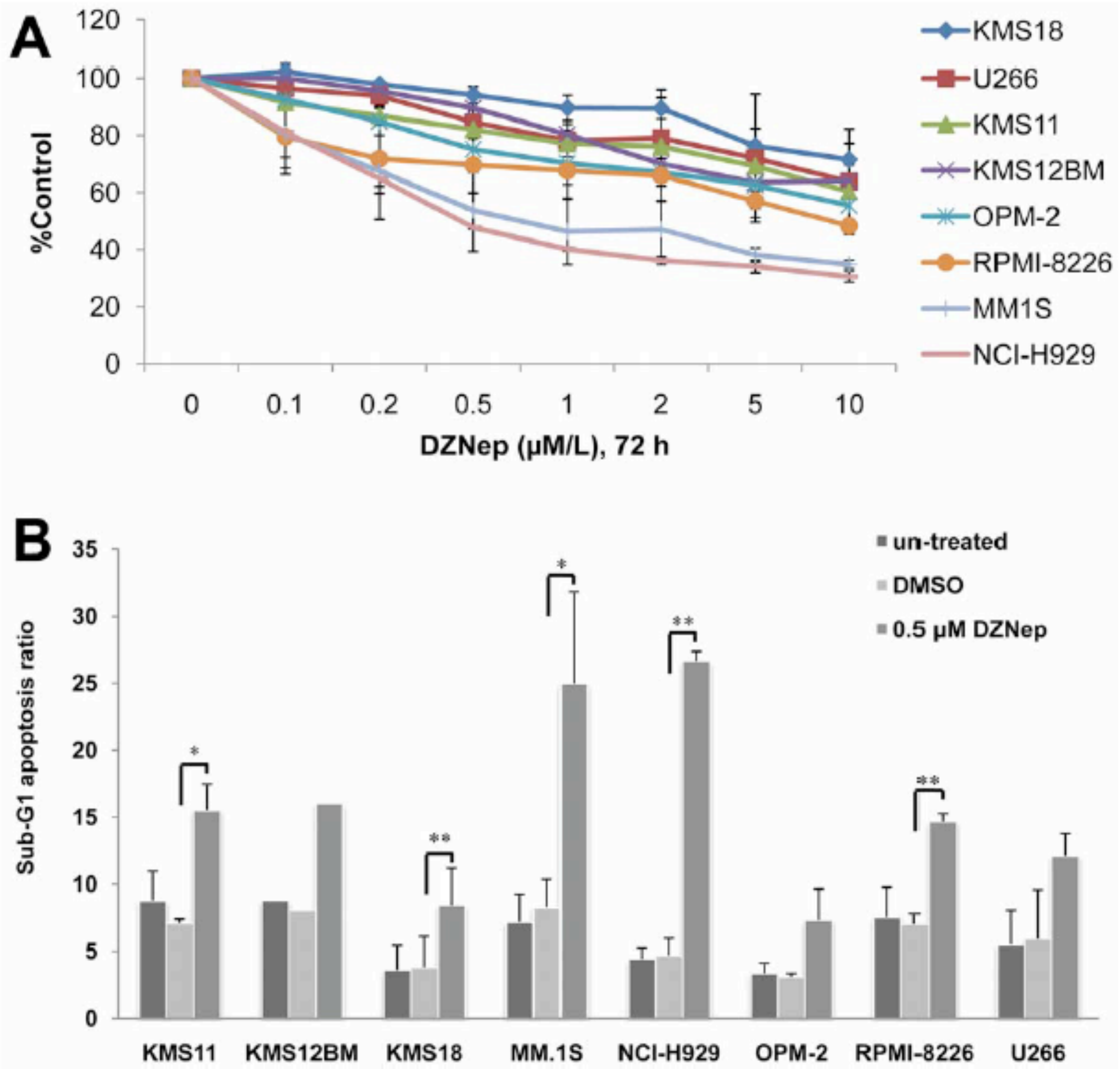


**Figure 1.** MM cell lines display differing degrees of sensitivity to DZNep. (**A**) Cells were treated with various concentrations of DZNep for 72 h. The proliferation was determined by MTS colorimetric assay (Promega). (**B**) Cells were treated with 0.5 μM DZNep for 72 h, and apoptosis was measured by flow cytometric sub-G1 analysis * indicates p,0.05, and ** indicates p,0.01

**Fig. S3A**

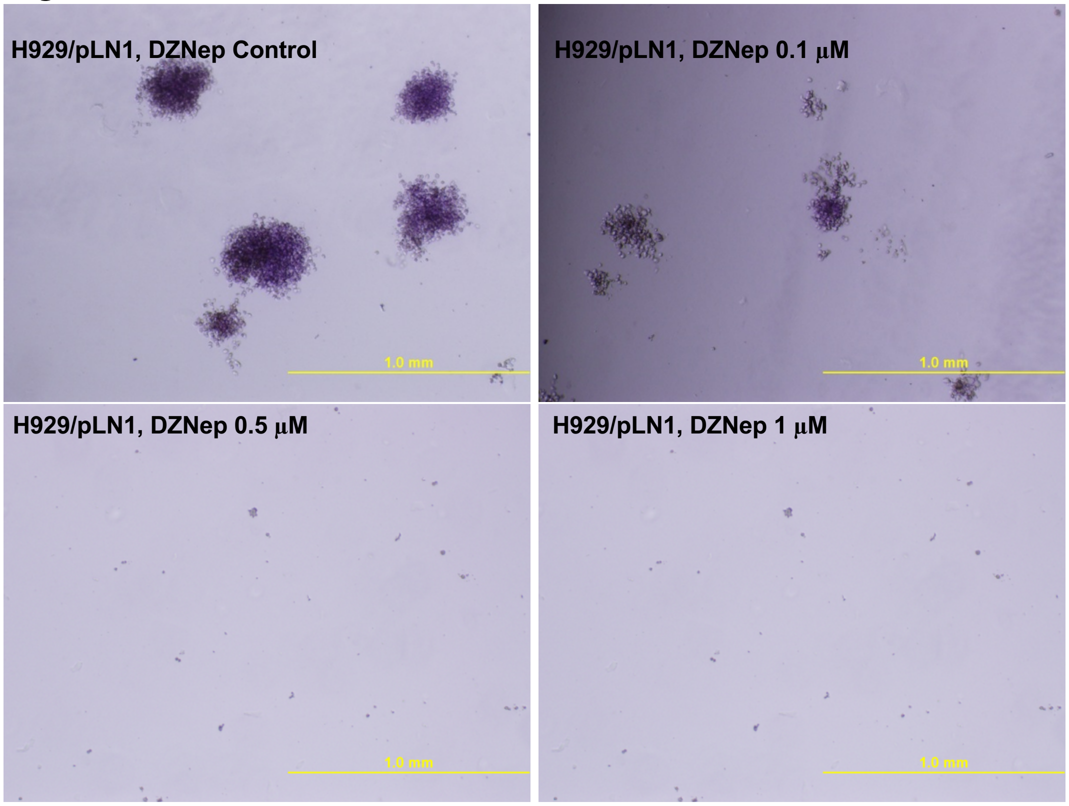


**Fig. S3C**

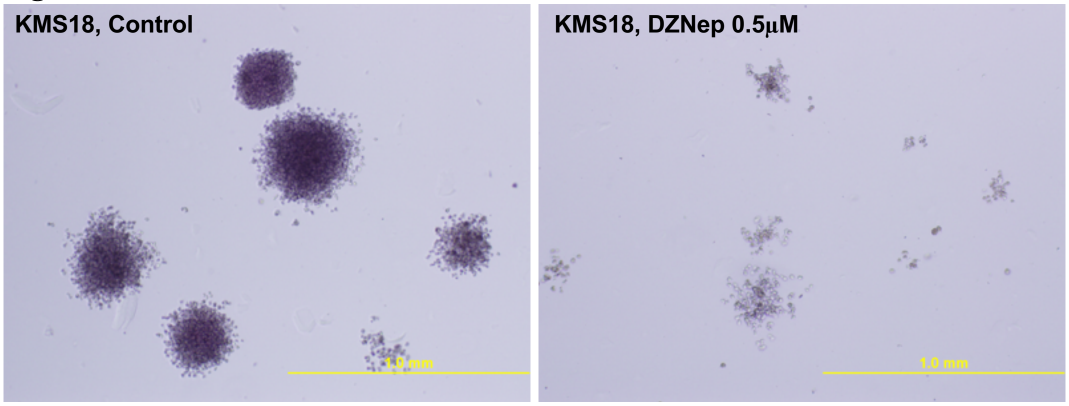


**Figure S3A,C. Colony formation assay in methylcellulose media**. Cells were plated in duplicate at a density of 1,500 or 3,000 cells/mL in 0.4 mL volume in 24-well plates. Plates were incubated at 37°C, 5% $CO_2$, and ≥95% humidity for 14 days. To observe colonies, cells were stained with 0.5 mg/mL metabolizable tetrazolium salt. After incubating at 37°C, 5% $CO_2$ for 1 h, images were acquired at 40X magnification with an Olimpus IX71 inverted microscope.

**16.21 NEUROBLASTOMA (IN VITRO ANTICANCER ACTIVITY)**

Wang C et al., EZH2 Mediates Epigenetic Silencing of Neuroblastoma Suppressor Genes CASZ1, CLU, RUNX3, and NGFR. Cancer Res 2011;72:315–324.

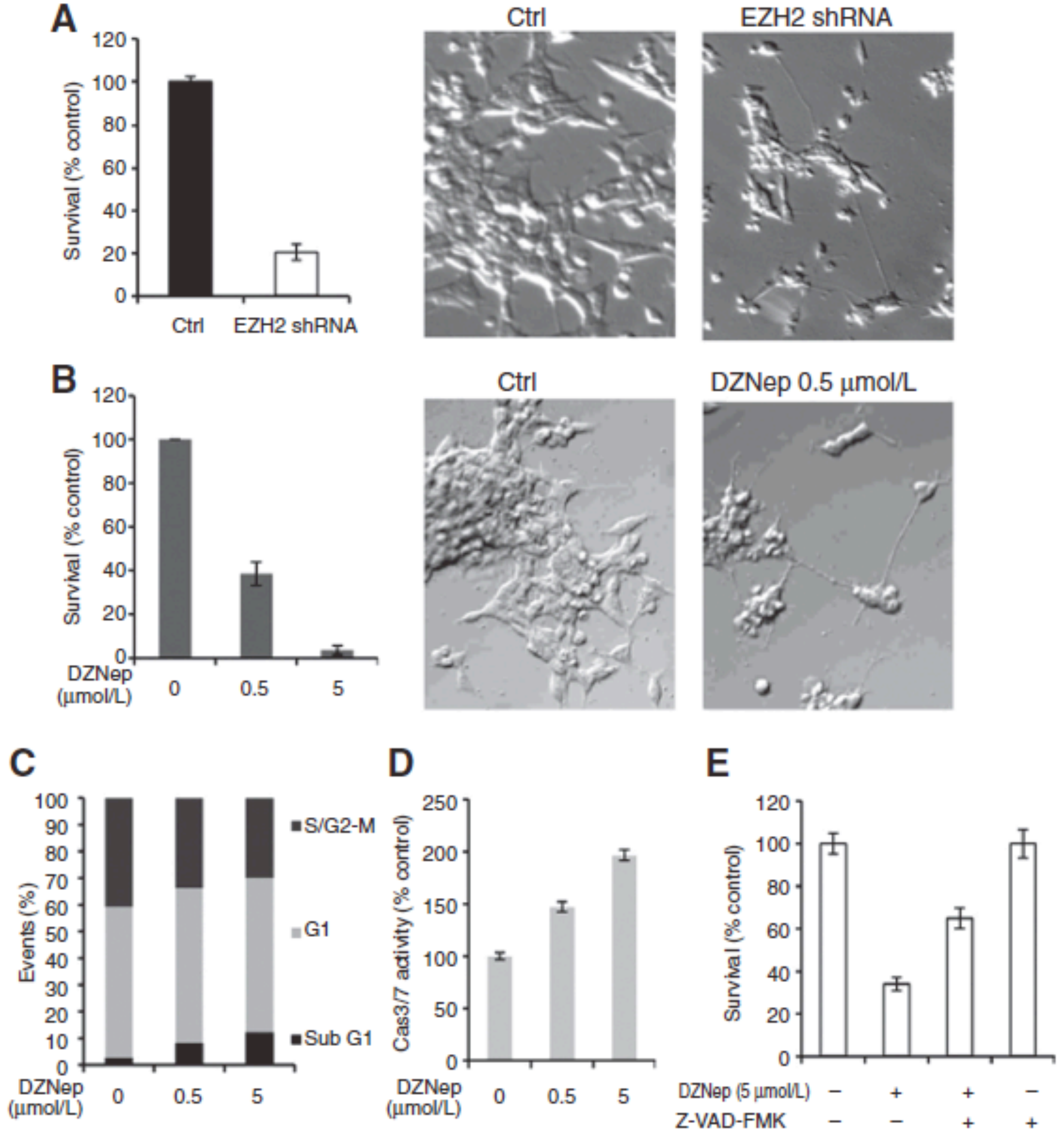


**Figure 7**. Decrease of EZH2 affects the cell growth and induces the neurite growth. **A,** cell survival in KCNR cells after a 3-day infection with EZH2 or nontarget shRNA lentivirus was assessed using a Cell-Titer Blue assay (left). The percentage of surviving cells was normalized by the absorbance value of the nontarget shRNAinfected cells (control). Representative images

(200x) of the nontarget shRNA-infected cells (ctrl, middle), EZH2 shRNA– infected cells (EZH2 shRNA, right). **B**, KCNR cells were treated with different concentration of DZNep for 96 hours. MTS assay was used to detected cell survival (left). The percentage of surviving cells was normalized by the absorbance value of the nontreated cells. Representative images (200x) of the nontreated cells (ctrl, middle) and 0.5 mmol/L DZNep-treated cells (DZNep 0.5 mmol/L, right). **C**, KCNR cells were treated with different concentration of DZNep for 96 hours. The cells were stained with propidium iodide and analyzed by flow cytometry. The data showed percentage of events in sub-G1, G1, and S/G2-M phase. **D**, caspase 3/7 activities were assessed after 48 hours with different concentration of DZNep in KCNR cells. The percentage of caspase 3/7 activity was graphed after normalization to nontreated cells. **E**, KCNR cells were treated with 5 mmol/L DZNep in the absence or presence of 100 mmol/L pan caspase inhibitor, Z-VADFMK, for 72 hours. The percentage of surviving cells was graphed after normalization to untreated control.

**COMMENTARY OF RESULTS**

Reducing EZH2 expression by RNA interference–mediated knockdown or pharmacologic inhibition with 3-deazaneplanocin A (DZNep) inhibited neuroblastoma (NB) cell growth, induced neurite extension and apoptosis. The growth of cells infected with the EZH2-shRNA was decreased to 20% after 3 days compared with control shRNA-infected cells (**Fig. 7A**). The NB cells displayed a concentration-dependent decrease in cell survival after 4-day DZNep treatment (**Fig. 7B**). There was also an increase in cells with neurite-like processes in the EZH2 shRNA–infected cells (Fig. 7A), and similar morphologic changes were observed in DZNep-treated cells (**Fig. 7B**). Cell-cycle analysis of DZNep-treated KCNR cells indicated a 4-fold increase in the subG1 phase of the cell cycle and a decrease in the growth fraction (S/G2-M; **Fig. 7C**). To assess whether DZNep-induced cell death via a caspase-dependent apoptotic pathway, we evaluated caspase-3/7 activity. There was up to a 2-fold increase in caspase-3/7 activity in DZNep treated NB cells (**Fig. 7D**). Incubation of cells with a pan caspase inhibitor (Z-VAD-FMK) partially blocked the DZNep-mediated decrease in NB cell survival (**Fig. 7E**). These data indicate that the DZNep-induced increases in cell death are partially due to induction of caspase-dependent apoptotic pathways.

## 6.22 BREAST & COLON TUMORS (IN VITRO ANTICANCER ACTIVITY)

Tan J, Yang X, Zhuang L, Jiang X, Chen W, Lee PL, et al. Pharmacologic disruption of Polycomb-repressive complex 2-mediated gene repression selectively induces apoptosis in cancer cells. Genes Dev. 2007;21:1050-63.

The authors found that DZNep at 5 µM induced time-dependent cell death in breast cancer MCF-7 and colorectal cancer HCT116 cells, as determined by propidium iodide (PI) staining and flow cytometry analysis (**Fig. 1B**).

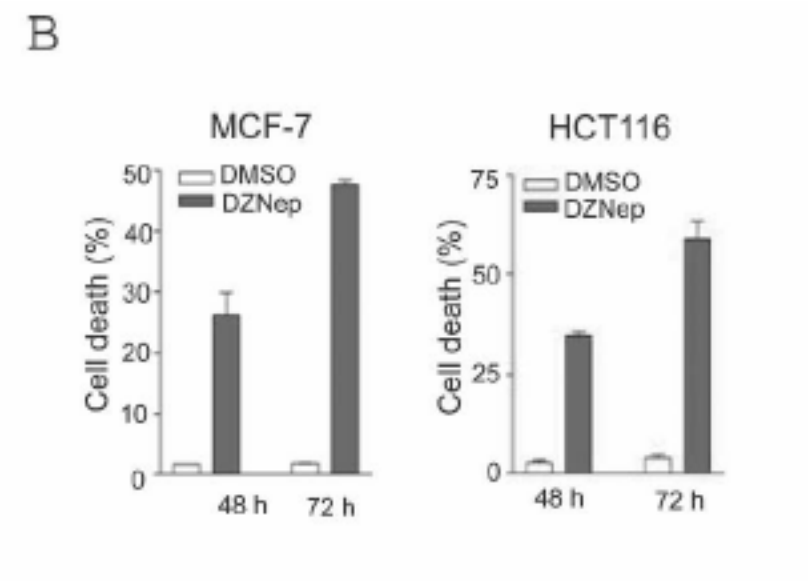


**Figure 1B.** MCF-7 and HCT1116 cells were treated with 5 µM DZNep for 48 and 72 h, followed by PI and FACS analysis

DZNep at 5 µM also induced cell death in a variety of other cancer cell lines, including breast cancer MB-468 cells, colorectal cancer, RKO, SW480, hepatoma Hep3B, and prostate cancer DU- 145 cells (**Fig. 1E**). In contrast, DZNep did not induce obvious cell death in normal cells, including noncancerous breast epithelial MCF-10A cells, lung epithelial IMR90 cells, primary human lung fibroblast cells MRC- 5, and human skin fibroblast cells T-HFF. Therefore, DZNep appeared to preferentially induce apoptosis in cancer cells.

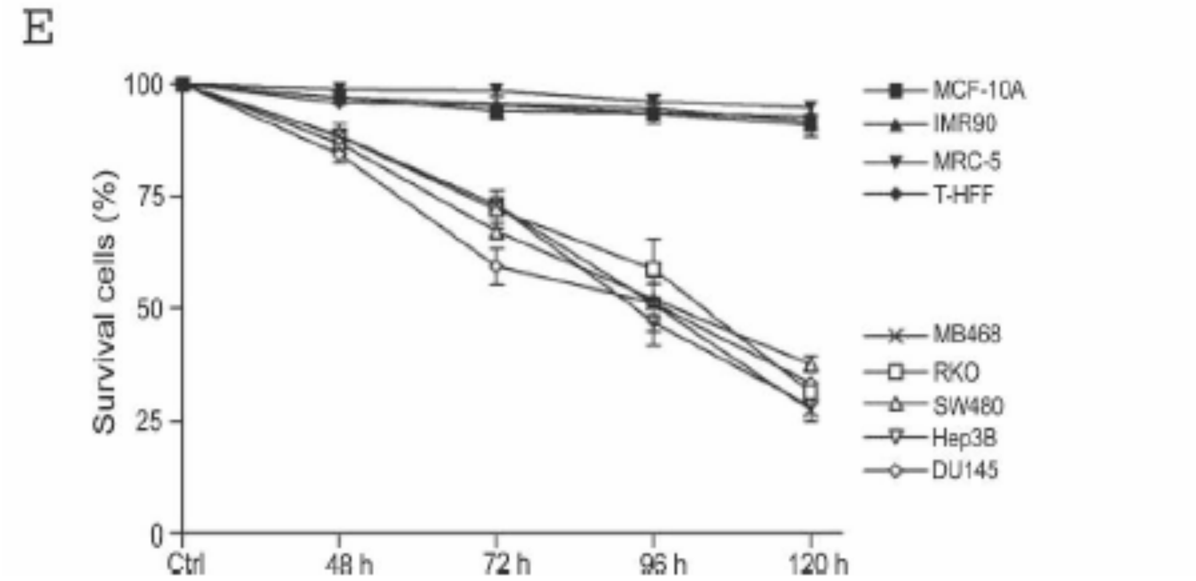


Figure 1E. Response of different cancer cells and normal cells to DZNep. Indicated cells were treated with 5 μM DZNep for up to 120 h and cell death was measured by PI staining and FACS analysis.

## 6.23 OSTEOSARCOMA (IN VITRO ANTICANCER ACTIVITY)

Sun R et al., Overexpression of EZH2 is associated with the poor prognosis in osteosarcoma and function analysis indicates a therapeutic potential. Oncotarget. 2016;7(25):38333-38346.

EZH2 silencing by siRNA inhibited osteosarcoma cell growth, proliferation, migration, and invasion. Moreover, suppression of EZH2 attenuated cancer stem cell functions. Similar results were observed in osteosarcoma cells treated with EZH2 specific inhibitor 3-deazaneplanocin A (DZNep), which inhibited tumor growth and exhausted cellular levels of EZH2. These results suggest that EZH2 is critical for the growth and metastasis of osteosarcoma, and an epigenetic therapy that pharmacologically targets EZH2 via specific inhibitors may constitute a novel approach to the treatment of osteosarcoma.

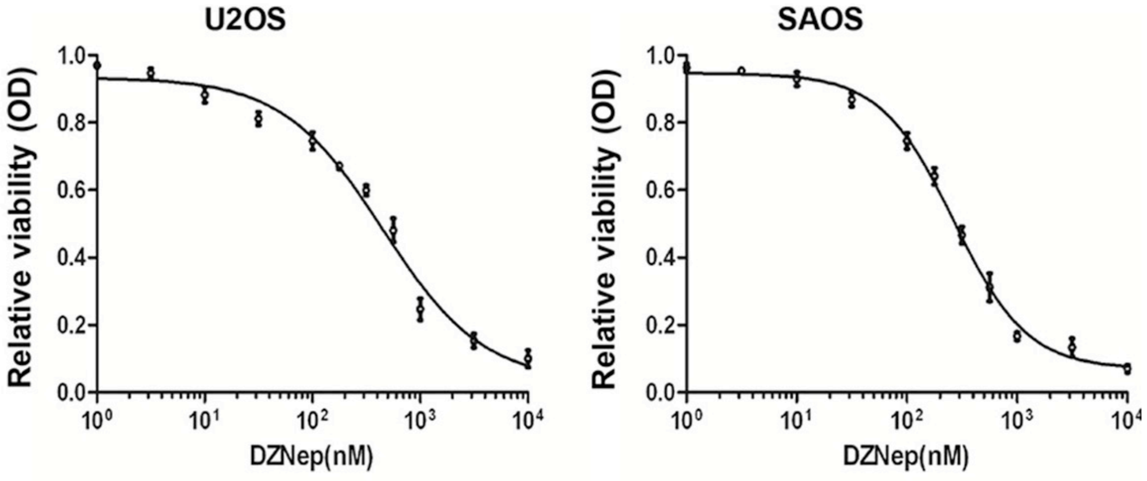


**Figure 6B. Effects of EZH2 inhibitor DZNep on the growth of osteosarcoma cell lines.** DZNep inhibited osteosarcoma cell proliferation. Cells were treated with DZNep at the indicated concentrations. The relative sensitivity of each line to DZNep was determined by the MTT assay.

**16.24 OVARIAN CANCER (IN VITRO ANTICANCER ACTIVITY)**

Shen L et al., 3-Deazaneplanocin A is a Promising Therapeutic Agent for Ovarian Cancer Cells. Asian Pac J Cancer Prev. 2013;14(5):2915-2918..

3-Deazaneplanocin A (DZNep) inhibits the proliferation of A2780 ovarian cancer cells. DZNep is a promising therapeutic agent for ovarian cancer, with potential to inhibit proliferation.

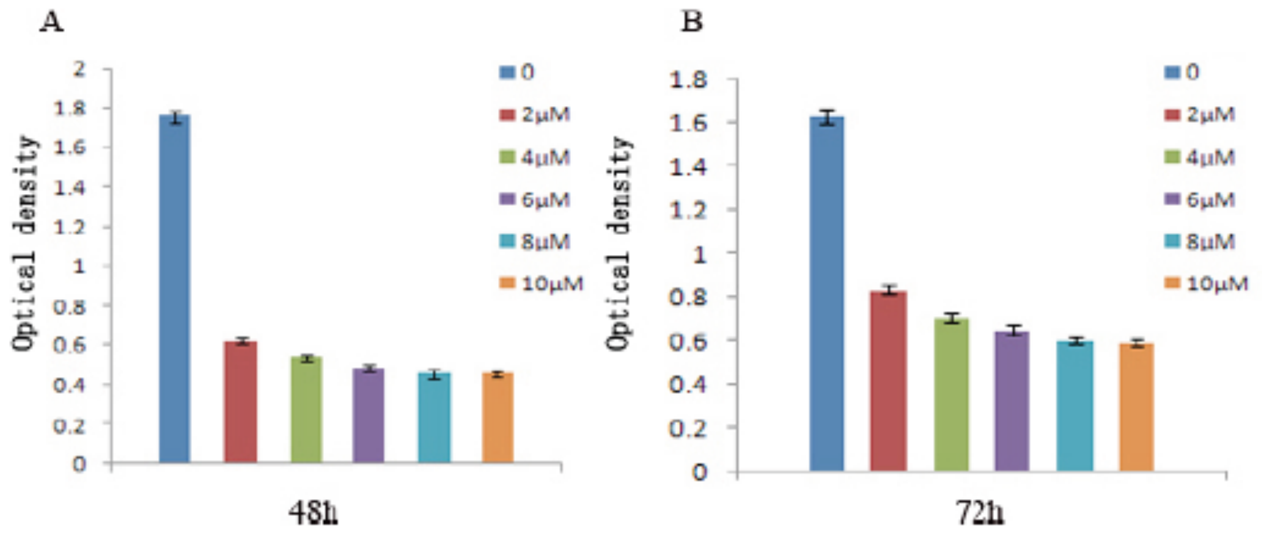


**Figure 1.** (**A**) Treatment of several different concentrations of DZNep on A2780 ovarian carcinoma cells for 48h. (**B**) Treatment of several different concentrations of DZNep on A2780 cells for 72h.

## 6.25 PROSTATE CANCER-1 (IN VITRO EX VIVO ANTICANCER ACTIVITY)

Crea F et al. Pharmacologic disruption of Polycomb Repressive Complex 2 inhibits tumorigenicity and tumor progression in prostate cancer. Mol Cancer. 2011;10:40.

The authors investigated the antineoplastic activity of 3-deazaneplanocin A (DZNep) against prostate cancer. DZNep (1□□M) inhibited prostatosphere (PS) growth in vitro by 95% (**Fig. 10A-B**). DZNep decreased the CD44-/24+ fraction, **suggesting DZNep is targeting the cancer stem cell population**. Treatment of LNCaP cells ex vivo with 1 and 10 µM DZNep to remove the cancer stem cell population, inhibited in vivo growth of these cells when subsequently injected into mice (**Fig. 10C-D**).

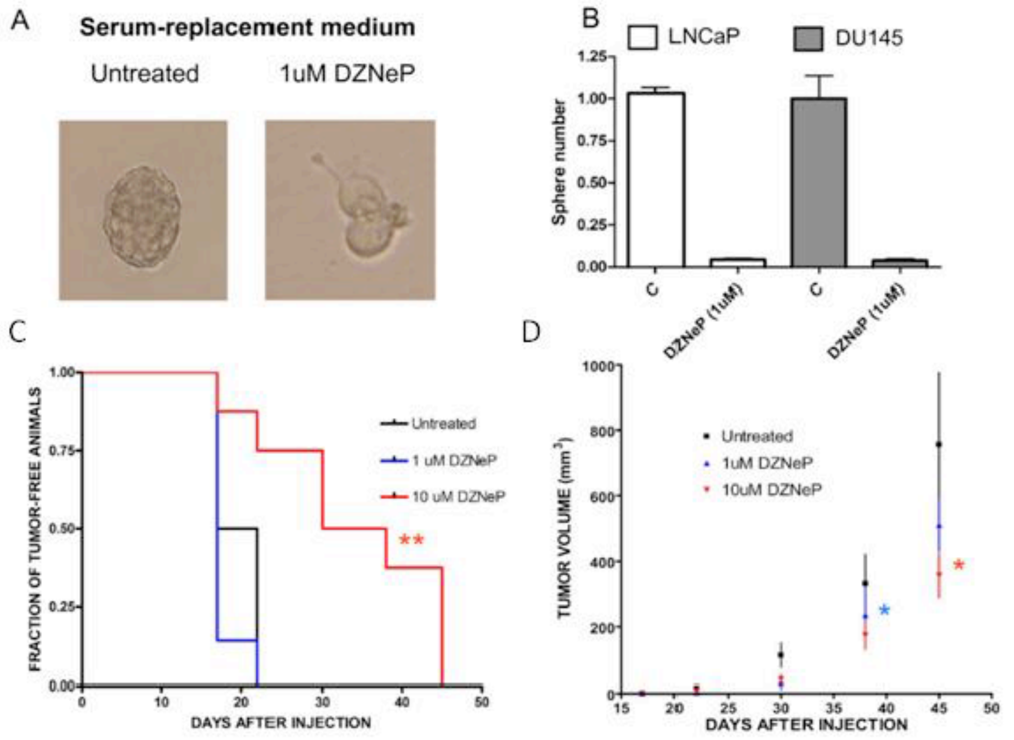


**Figure 10**. DZNep inhibits sphere formation and tumor growth of prostate cancer cells *ex vivo*. A. representative picture of cells grown in stem cell medium for 7 days. B. PS number in untreated and DZNep treated cells (1 µM, 7 days). Columns, mean volume; bars standard deviation. C-D. LNCaP cells were pretreated with DZNep (1 and 10 mM) and then transplanted into NOD/SCID mice. At 10 µM, DZNep significantly inhibited the time to tumor formation (C) and tumor volume (D). **p < 0.01 (log rank test) with respect to untreated cells. *p < 0.05 with respect to untreated cells (U test). Star colors refer to dose treatment (blue, 1 µM; red, 10 µM). Number of animals: untreated: 6; 1 and 10 µM: 8. Point, mean value; bar, standard deviation.

## 6.26 PROSTATE CANCER-2 (IN VITRO ANTICANCER ACTIVITY)

Wee ZN et al., EZH2-mediated inactivation of IFN-γ-JAK-STAT1 signaling is an effective therapeutic target in MYC-driven prostate cancer. Cell Rep. 2014;8(1):204-216.

The results show that pharmacological depletion of EZH2 by DZNep in DU145 cells was induced the expression of IFNGR1, which when combined with IFN-ϒ led to strong inductions of IFN genes (**Figure 5A**) as well as STAT1 phosphorylation (**Figure 5B**). DZNep was able to induce robust apoptosis when combined with increasing doses of IFN-ϒ in DU145 cells, but not LnCap, cells (**Figure 5C**).

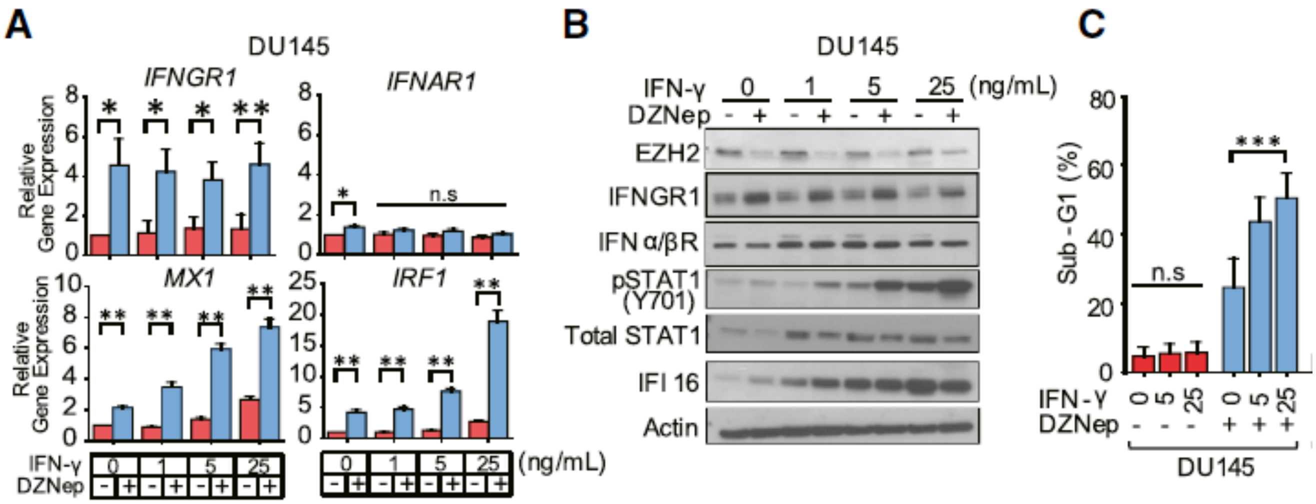


**Figure 5**. DZNep Restores IFN-ϒ Response for Growth Inhibition and Apoptosis
(**A**) qPCR showing the increase in gene expression of IFNGR1 and various IFN-responsive genes, after 72 hr of DZNep (2.5 µM) and IFN-ϒ treatment at the indicated doses. (**B**) Western blot analysis of IFNGR1 expression and IFN-ϒ signaling in DU145 cells treated with DZNep (2.5 µM), IFN-ϒ, or both for 3 days. (**C**) Sub-G1 DNA analysis in DU145 and LNCaP cells treated as in (B).

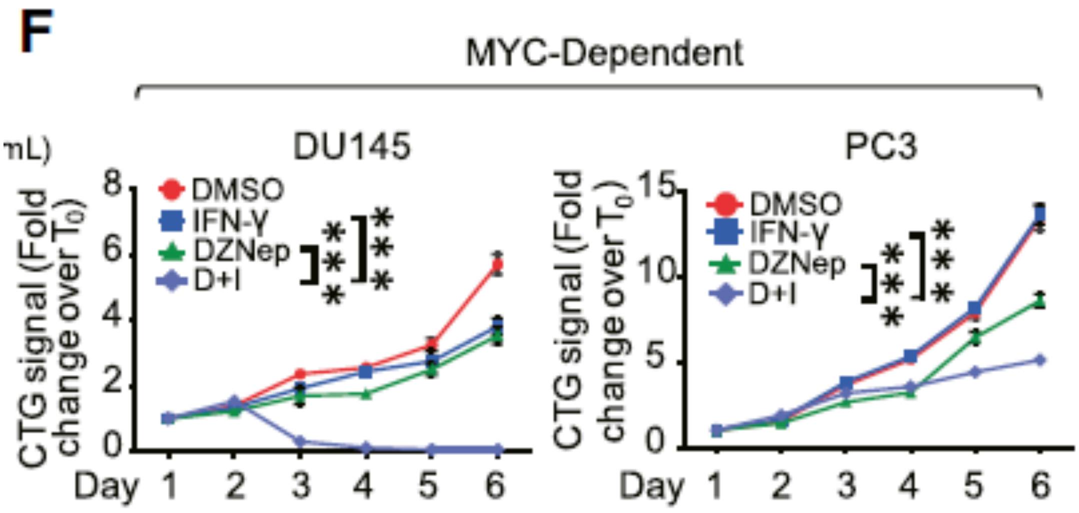


**Figure 5**. DZNep Restores IFN-ϒ Response for Growth Inhibition and Apoptosis

(**F**) Cell proliferation assay of MYC-dependent cell lines (DU145 and PC3) after treatment with IFN-ϒ, DZNep, or both for days as indicated. The proliferation of the cells is represented as the fold change after normalizing to the baseline CTG signal on day 0 (T0).

## 6.27 RHABDOMYOSARCOMA (IN VITRO ANTICANCER ACTIVITY)

Ciarapica R et al., Pharmacological inhibition of EZH2 as a promising differentiation therapy in embryonal RMS. BMC Cancer 2014, 14:139.

Pharmacological inhibition of EZH2 using 3-deazaneplanocin A (DZNep) prevented cell proliferation of rhabdomyosarcoma (RMS) cells.

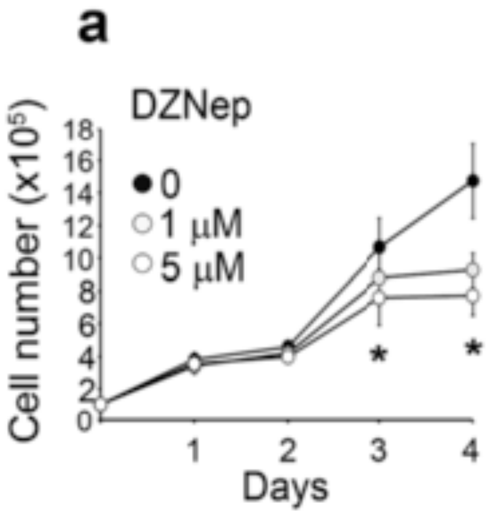


**Figure 5a** Pharmacological inhibition of EZH2 prevents embryonal RMS cell proliferation. RD cells cultured in proliferating growth medium (GM, i.e. supplemented with 10% of fetal calf serum) were treated daily with either the S-adenosyl-L-homocysteine hydrolase inhibitor 3-deazaneplanocin A (DZNep) and harvested and counted at the indicated time points. *P < 0.05 (Student's t-test); Bars, SD. Three independent experiments in duplicate.

## 6.28 TONGUE CANCER (IN VITRO ANTICANCER ACTIVITY)

Li et al., The polycomb group protein EZH2 is a novel therapeutic target in tongue cancer. Oncotarget 2013; 4:2532-2549.

DZNep induced a time-dependent and dose-dependent decrease of EZH2 in Cal27 and Tca8113 tongue cancer cells. The most significant inhibitions were observed when cells were treated with 5 µM DZNep and for 72h (**Fig.2A**). The abundance of H3K27me3 was pronouncedly downregulated following DZNep incubation (**Fig.2B**). EZH2 mRNA remained almost unchanged following DZNep exposure (**Fig.2C**), suggesting that DZNep does not inhibit the synthesis of its mRNA. The addition of proteosome inhibitor MG132 partially prevented the reduction of EZH2 induced by DZNep (**Fig.2D**), implying that DZNep inhibited EZH2 probably through increased protein degradation.

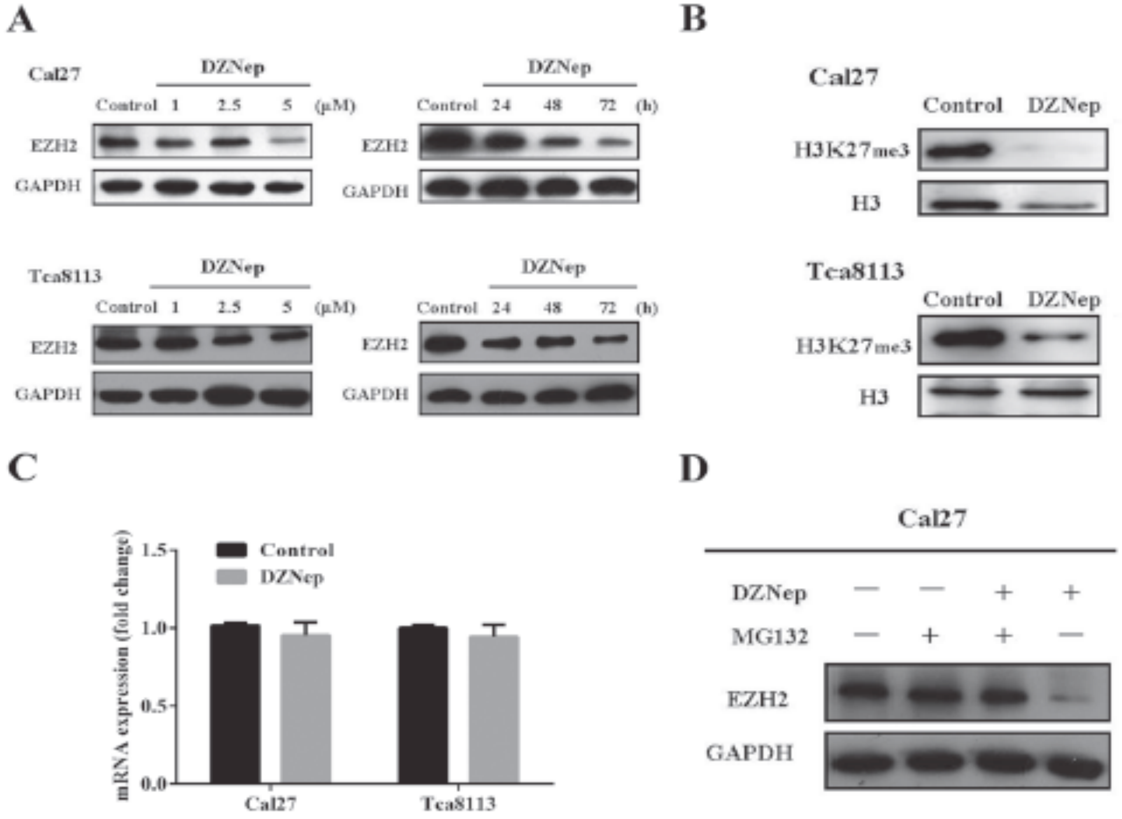


**Fig 2:** DZNep inhibits endogenous EZH2 by proteosome-mediated protein degradation. **A:** DZNep inhibits EZH2 in a dose and time-dependent manner in both Cal27 and Tca8113 cells. **B:** H3k27me3 was simultaneously reduced following DZNep exposure (5µM, 72h) in both Cal27 and Tca8113 cells. **C:** Real-time RT-PCR assay for EZH2 mRNA levels following DZNep exposure (5µM, 24h) in both Cal27 and Tca8113 cells. **D:** EZH2 protein was determined by Western blotting after Cal27 was exposed to DZNep or/and the proteosome inhibitor MG132 for

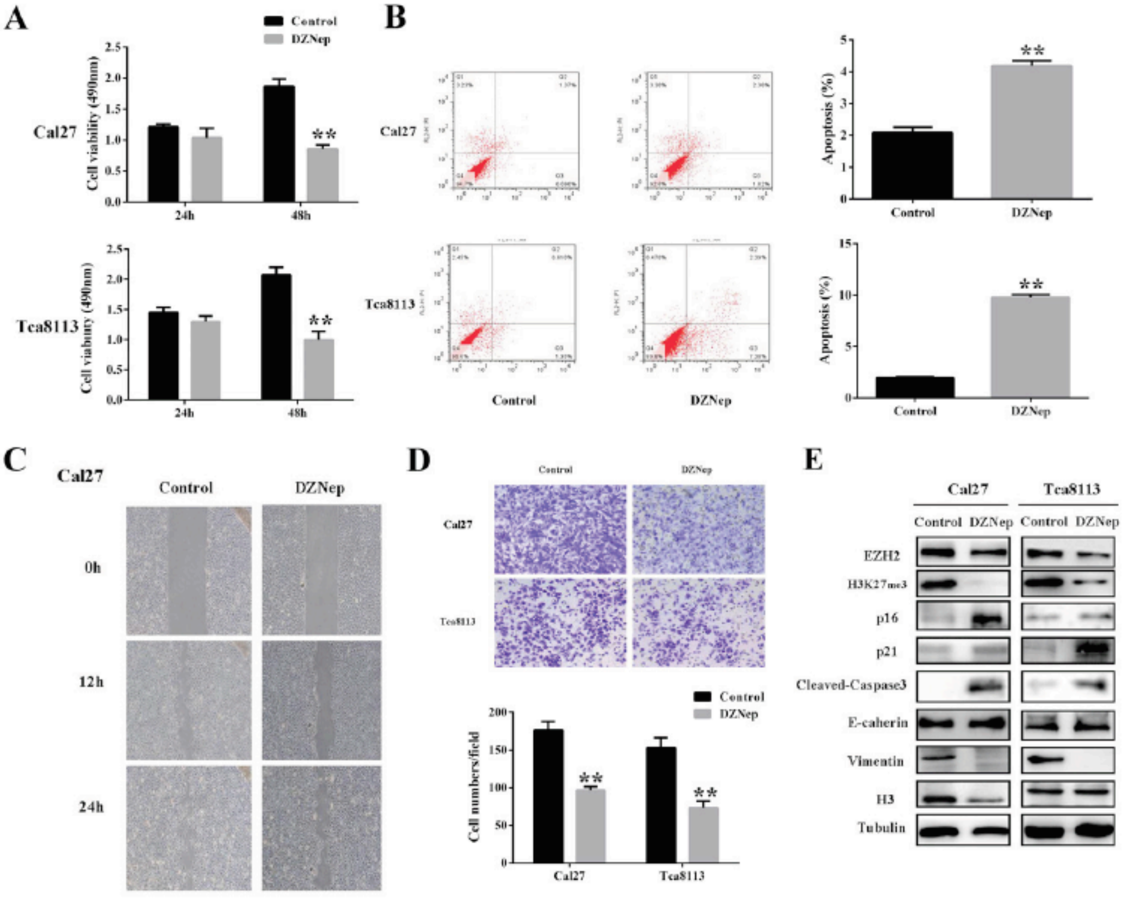


**Fig 3:** DZNep inhibits cell growth and migration and invasion, while induces cell apoptosis in tongue cancer cells. **A:** Cell proliferation was significantly impaired after DZNep treatment for 48h as measured by MTT assay. **B:** The percentages of apoptotic cells were remarkably increased after DZNep treatment as determined by flow cytometry. **C**: Migration potentials of Cal27 treated with DZNep and vehicle were determined by wound healing assay. D: Invasion potentials of Cal27 treated with DZNep and vehicle were determined by modified Boyden chamber assay. **E:** The protein abundances of several downstream effectors or targets of EZH2 before and after DZNep exposure were measured by Western blotting.

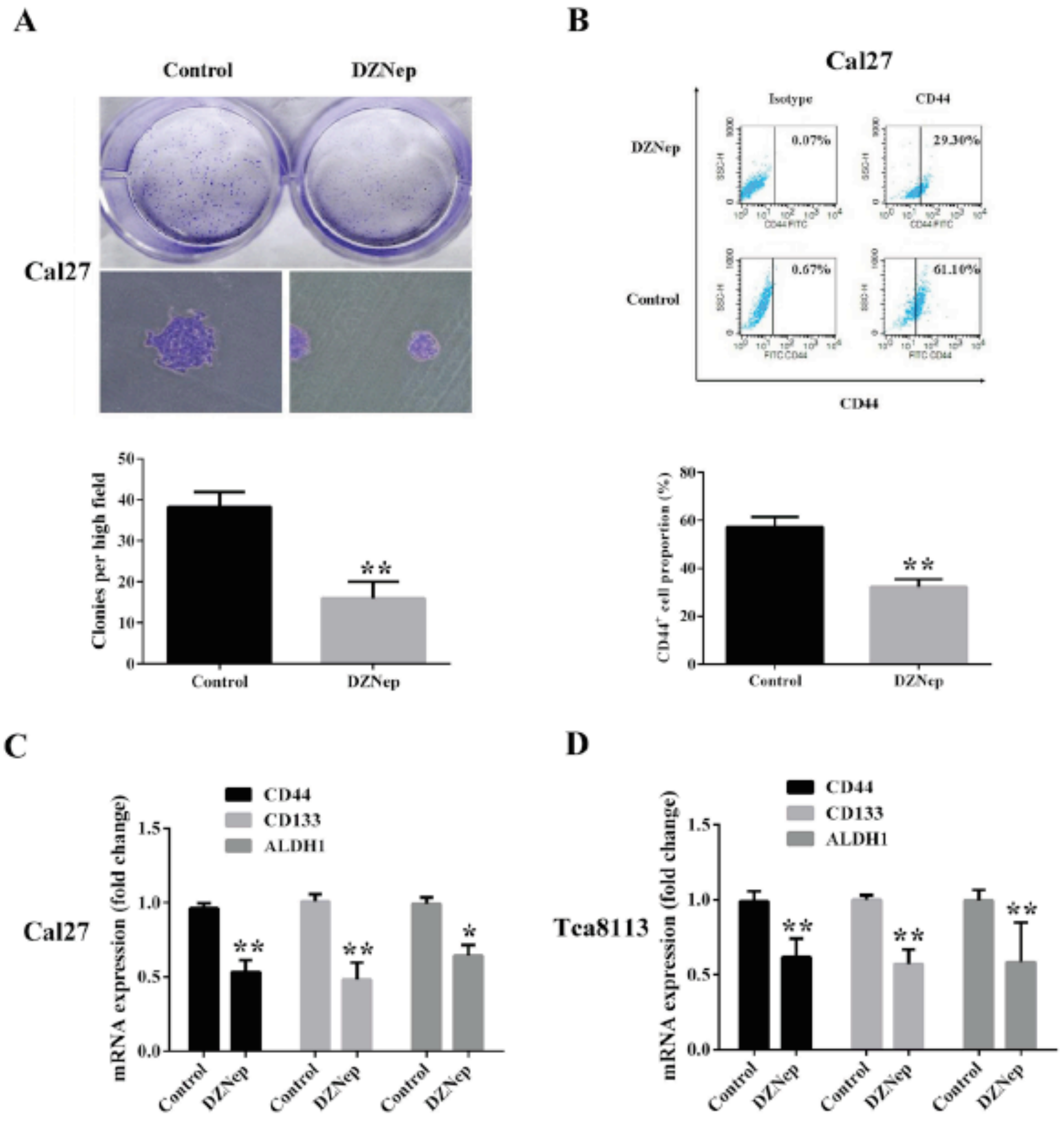


**Fig 4:** DZNep reduces colony formation and CD44+subpoputation in tongue cancer cells. **A:** The number and size of colonies formed by Cal27 pretreated with DZNep or vehicle as stained by crystal violet. **B:** The percentage of CD44 positive subpopulation in DZNep or vehicle treated Cal27 cells was determined by FACS. **C, D:** The mRNA levels of several common markers for cancer stem cell isolation were quantified using real-time RT-PCR assay.

# 7. SUMMARY TABLE OF IN VITRO ANTICANCER ACTIVITY DZNep

Momparler R & Côté S. Targeting of cancer stem cells by inhibitors of DNA and histone methylation. Expert Opin. Investig. Drugs 2015;24:8.

**Table 1.** ***In vitro*** **antineoplastic action of 3-deazaneplanocin-A (DZNep) on leukemic cells.**

| Type of cancer | Cell lines | Antineoplastic action by DZNep | Ref. |
|---|---|---|---|
| Leukemia | HL-60 | 1000 nM 24 h 35% reduction colony formation<br>1000 nM 24 h 30% inhibition of growth<br>1000 nM 24 h 15% induction of apoptosis | [29] |
| Leukemia | AML-3 | 500 nM 24 h 20% reduction colony formation<br>500 nM 24 h 15% inhibition of growth<br>500 nM 24 h 10% induction of apoptosis | [29] |
| Leukemia | HL-60 | 500 nM 72 h 30% induction of apoptosis<br>1000 nM 72 h 35% induction of apoptosis<br>200 nM 48 h 65% reduction colony formation<br>500 nM 48 h 90% reduction colony formation | [47] |
| Leukemia | AML-3 | 200 nM 72 h 8% induction of apoptosis<br>500 nM 72 h 35% induction of apoptosis<br>200 nM 48 h 60% reduction colony formation<br>500 nM 48 h 80% reduction colony formation | [47] |
| Leukemia | MOLM-14<br>MV4-11<br>Kazumi-1<br>TF-1<br>Mono-Mac-1 | 4.2 μM 48 h 50% induction of apoptosis<br>6.3 μM 48 h 50% induction of apoptosis<br>4.8 μM 48 h 50% induction of apoptosis<br>12.5 μM 48 h 50% induction of apoptosis<br>15.0 μM 48 h 50% induction of apoptosis | [71] |
| Leukemia<br>(Erythro-) | K562 | 1.0 μM 72 h 60% inhibition of growth<br>1.0 μM 72 h 16% induction of erythroid differentiation | [79] |
| Lymphoma<br>Mantle cell | MO2058<br>JeKo-1 | 2.0 μM 48 h 32% induction of apoptosis<br>2.0 uM 48 h 35% induction of apoptosis | [80] |

# 8. IN VIVO ANTICANCER ACTIVITY

## 8.1 LEUKEMIA-1 (IN VIVO ANTICANCER ACTIVITY)

Momparler RL, Idaghdour Y, Marquez VE, Momparler LF. Synergistic antileukemic action of a combination of inhibitors of DNA methylation and histone methylation. Leuk Res. 2012;36:1049-54.

The antineoplastic activity of DZNep was evaluated in vivo using the L1210 mouse model of leukemia (**Table1**). Mice were injected iv with L1210 leukemic cells on day 0 and administered sequentially 2 mg/kg 5-AZA-CdR as 12 h iv infusion on day 1 followed by ip injection of 2.5 mg/kg (Experiment #1) or 3.75 mg/kg (Experiment #2) DZNep at 1 and 6 h post-infusion. While the single treatment with either 5-AZA-CdR or DZNep resulted in an increased live span of 76.6 and 22.1%, respectively, compared to the untreated mice, the combination of 5-AZA-CdR and DZNep (at 5 or 7.5 mg/kg) produced 107% increase in life span.

Table 1. Antineoplastic activity of 5-aza-2′-deoxycytidine (5-AZA-CdR) and 3-deazaneplanocin A (DZNep) in mice with L1210 leukemia.

| Drug(s) | Dose (mg/kg) | Infusion time | Survival (days) | % increase life span |
|---|---|---|---|---|
| Experiment #1 | | | | |
| Control (no drug) | 0 | | 7.7 ± 0.2[*] | 0% |
| 5-AZA-CdR | 2 mg/kg (i.v.) | 12 h | 13.6 ± 0.5 | 76.6%[**] |
| DZNep | 5 mg/kg (i.p.) | | 9.4 ± 0.3 | 22.1% |
| 5-AZA-CdR + DZNep | 2 mg/kg + 5 mg/kg | 12 h | 15.9 ± 1.5 | 106.5%[**] |
| Experiment #2 | | | | |
| Control (no drug) | 0 | | 6.9 ± 0.4[*] | 0% |
| 5-AZA-CdR | 2 mg/kg (i.v.) | 12 h | 12.5 ± 0.3 | 81.2%[**] |
| DZNep | 7.5 mg/kg (i.p.) | | 9.1 ± 0.2 | 31.9% |
| 5-AZA-CdR + DZNep | 2 mg/kg + 7.5 mg/kg | 12 h | 14.3 ± 1.1 | 107.2%[**] |

CD2F1 male mice were injected i.v. with 10,000 L1210 leukemic cells on day 0. On day 1 the mice received n 12 h i.v. infusion of 5-AZA-CdR (2 mg/kg). DZNep 2.5 mg/kg (Experiment #1) or 3.75 mg/kg (Experiment #2) was injected i.p. at 1 h and 6 h after end of infusion giving a total dose of 5.0 and 7.5 mg/kg, respectively. There were 5 mice per group. Survival time was determined.

*Mean ± SEM.

**5-AZA-CdR vs 5-AZA-CdR + DZNep ($p < 0.05$; t test).

## 8.2 LEUKEMIA-2 (IN VIVO ANTICANCER ACTIVITY)

Fiskus W et al. Combined epigenetic therapy with the histone methyltransferase EZH2 inhibitor 3-deazaneplanocin A and the histone deacetylase inhibitor panobinostat against Human AML cells . Blood 2009 114: 2733-2743.

DZNep plus PS increased the survival time of mice injected with HL-60 leukemic cells to a greater extent than either agent alone. (**Figure 4D**)

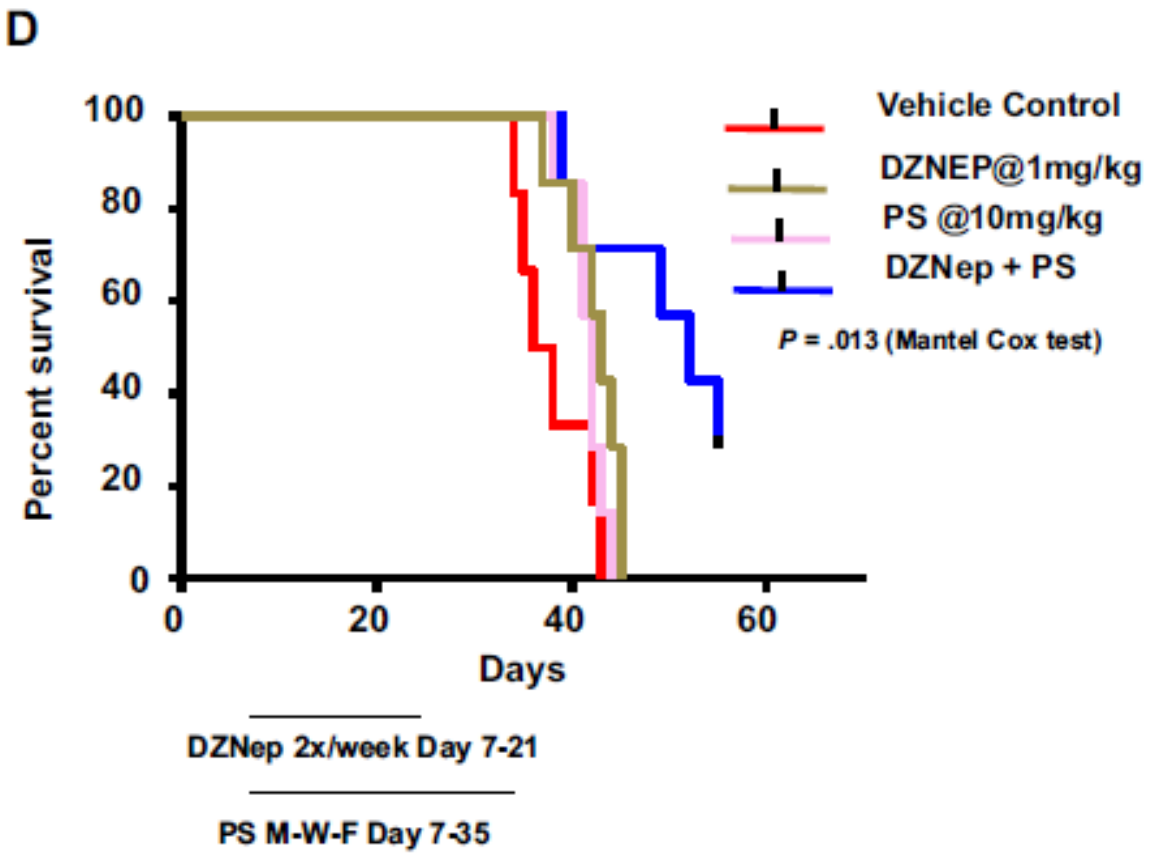


**Figure 4 (D)** Female NOD/SCID mice were injected in the lateral tail vein with HL-60 cells. The cells were allowed to engraft for 7 days before initiation of treatment. Mice were treated intraperitoneally with dimethylsulfoxide, 1 mg/kg DZNep 2 days per week for 2 weeks, and/or 10 mg/kg PS 3 days per week for 4 weeks. n=7 per group. Survival of the mice in all groups (vehicle, DZNep alone, PS alone, and combination) is represented by Kaplan-Meier plot.

### 8.3 LEUKEMIA-3 (IN VIVO ANTICANCER ACTIVITY)

Ueda K et al., Inhibition of histone methyltransferase EZH2 depletes leukemia stem cell of mixed lineage leukemia fusion leukemia through upregulation of p16. Cancer Sci 2014;105:512–519.

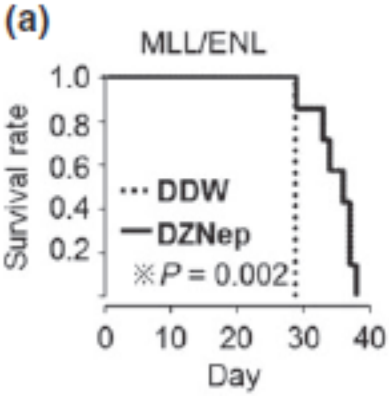


**Fig. 1a.** Administration of EZH2 inhibitor is therapeutically effective in MLL fusion leukemic mouse models. Mice were injected intravenously with 1 x $10^4$ of leukemia cells. From 7 days after injection, mice were treated intraperitoneally with DZNep, 2 mg/kg 3 days per week, In vivo administration of DZNep prolonged survival of MLL/ENL and MLL/ AF9 leukemic mice .

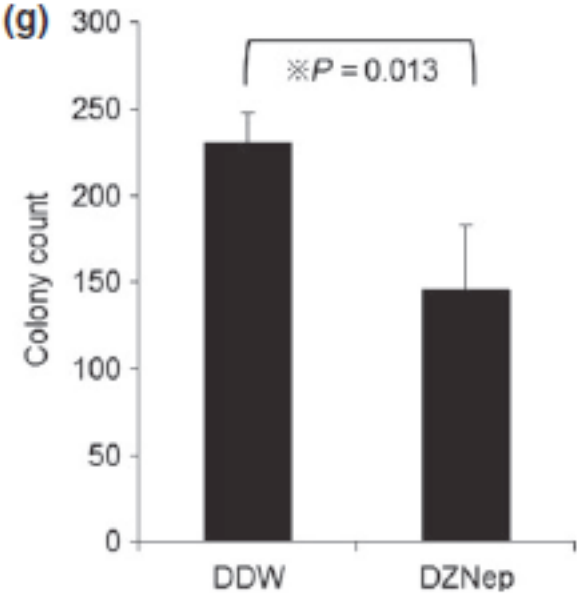


**Fig. 1g.** 1 x $10^4$ of sorted GFP positive bone marrow cells were placed in 1 ml of methocult M3434 and cultured for 5 days. Colony counts for each group are shown. The BM cells from MLL/ AF9 leukemic mice treated with DZNep had impaired capacity to reconstitute leukemia in vivo and attenuated colony forming capacity in vitro. **These data suggest that leukemia initiating cells (LIC) are decreased by DZNep administration** in MLL/ AF9 leukemic mice. Data not shown. The LIC cells were detected by flow cytometry using staining for CD34.

## 8.4 LEUKEMIA-4 (IN VIVO ANTICANCER ACTIVITY)

Zhou J et al., The histone methyltransferase inhibitor, DZNep, up-regulates TXNIP, increases ROS production, and targets leukemia cells in AML. Blood. 2011;118 (10):2830-2839.

DZNep treatment at 2 mg/kg/d significantly reduced MOLM-14 tumor growth (**Fig. 1B**) and prolonged the survival of NOD/SCID mice with AML (**Fig. 1C**) compared with the treatment with PBS.

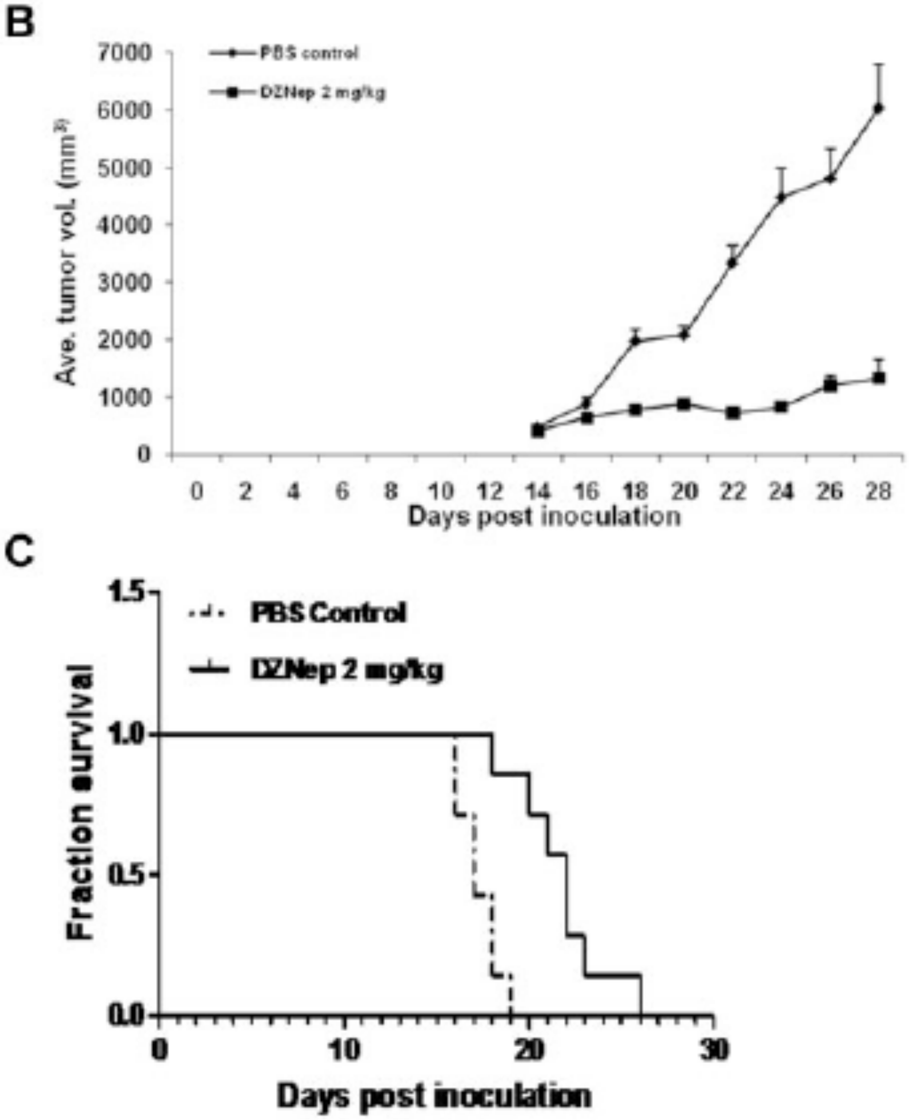


**Figure 1**. DZNep showed potent anti-AML efficacy in vitro. Mice were treated with PBS control or DZNep 2 mg/kg/d, respectively. (**B**) Tumor volume curves were constructed with measurements taken by caliper. Average of tumor volume was calculated as the average of 7 mice in each group SD. (**C**) Survival analysis showed that DZNep treatment improved the survival time of mice inoculated with human leukemia.

## 8.5 LUNG CANCER (IN VIVO ANTICANCER ACTIVITY)

Fillmore CM et al., EZH2 inhibition sensitizes BRG1 and EGFR mutant lung tumours to TopoI inhibitors. Nature. 2015;520(7546):239-242.

To determine if the protected and sensitized phenotypes could be observed in vivo, the xenograft-bearing mice were treated with etoposide +/− DZNep. For the sensitized cell line H157, treatment with etoposide and DZNep therapy prevented tumors from forming in 4/6 mice and more efficacious than etoposide or DZNep alone (**Fig. 2a**). In contrast, the protected H23 xenografts that received early dual therapy grew significantly larger than those treated with either DZNep or etoposide alone (**Fig. 2b**).

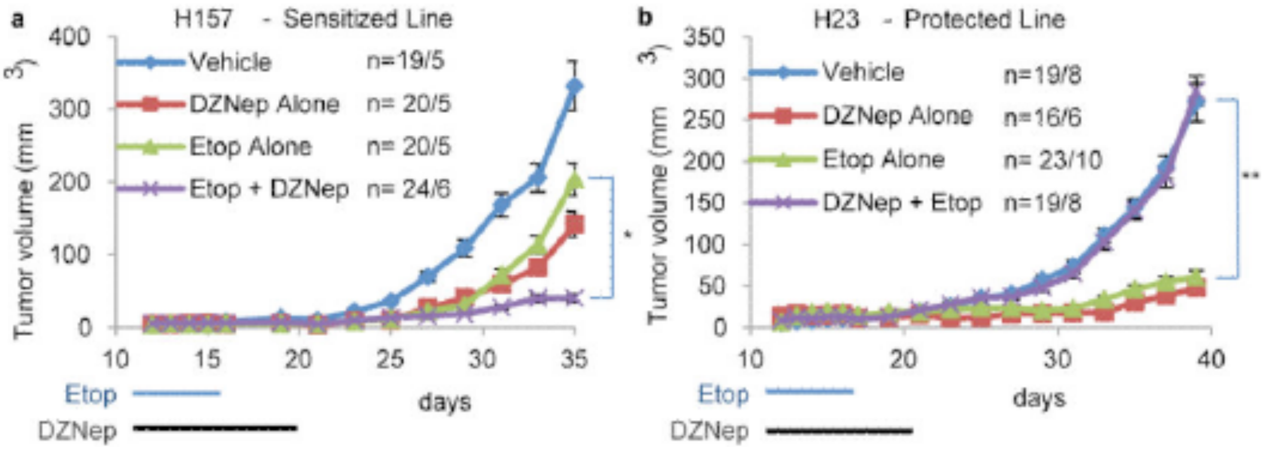


**Figure 2a,b** In vitro sensitivities to DZNep + TopoII inhibition predict in vivo responses
Either the H157 (**a**) or H23 **(b)** NSCLC cell line was injected into the flanks of Nude mice and tumors were allowed to form. On day 12, mice were randomly segregated into cohorts that received either placebo, DZNep (1 mg/kg/d x2/wk), etoposide or dual therapy for 2 weeks. DZNep tumor size reduction ~133% (Note: GSK-126 at 300 mg/kg/d x 2/wk ~ similar response as DZNep).

## 8.6 LYMPHOMA (IN VIVO ANTICANCER ACTIVITY)

Fiskus W et al. Superior efficacy of a combined epigenetic therapy against human mantle cell lymphoma cells. Clinical Cancer Res 2012;18:6227-6238.

The in vivo antitumor activity of 3-Deazaneplanocin A (DZNep) against human mantle cell lymphoma (MCL) cells was investigated. The authors determined the in vivo anti-MCL activity of treatment with DZNep and/or the histone deacetylases inhibitor panobinostat (PS) in JeKo-1 MCL cell xenografts in NOD/SCID mice. In cohorts of mice, JeKo-1 cells were implanted in the flanks, and the treatment with each agent or vehicle alone was begun after the flank tumors achieved a size of approximately 150 $mm^3$. As shown in Fig. 6D, although treatment with DZNep or PS alone also caused inhibition of tumor growth, combined treatment with DZNep and PS exerted superior growth inhibitory, antitumor effects against the JeKo-1 xenografts ($P < 0.05$). **Neither each agent alone nor the combination of DZNep and PS induced weight loss or other physical signs of toxicity in the xenograft bearing NOD/SCID mice.**

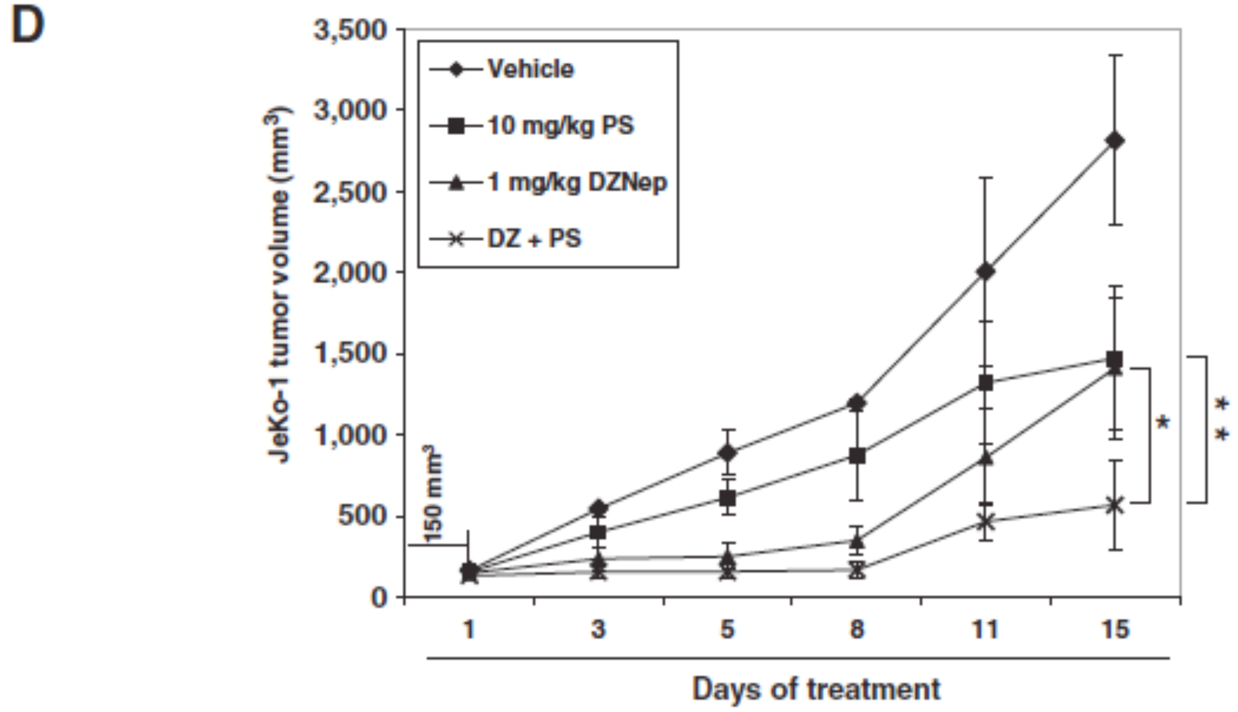


**Figure 6D.** Tumor growth of JeKo-1 cells MCL cells implanted in the flank of NOD/SCID mice and treated as indicated for 2 weeks. *, tumor volumes significantly less ($P = 0.02$) in the combination than treatment with DZNep (1 mg/kg; 2/wk x 2 wk) alone at the end of treatment. **, tumor volumes significantly less ($P = 0.008$) in combination than treatment with PS alone at the end of treatment. DZNep treatment reduced tumor size by 50%.

## 8.7 GLIOBLASTOMA (IN VIVO ANTICANCER ACTIVITY)

Smits M et al., miR-101 is down-regulated in glioblastoma resulting in EZH2-induced proliferation, migration, and angiogenesis. Oncotarget 2010;1:710-720.

The glioblastoma tumor volume of the mice treated with PBS increased over time, while the tumor volume in the mice treated with DZNep showed >70% reduced growth.

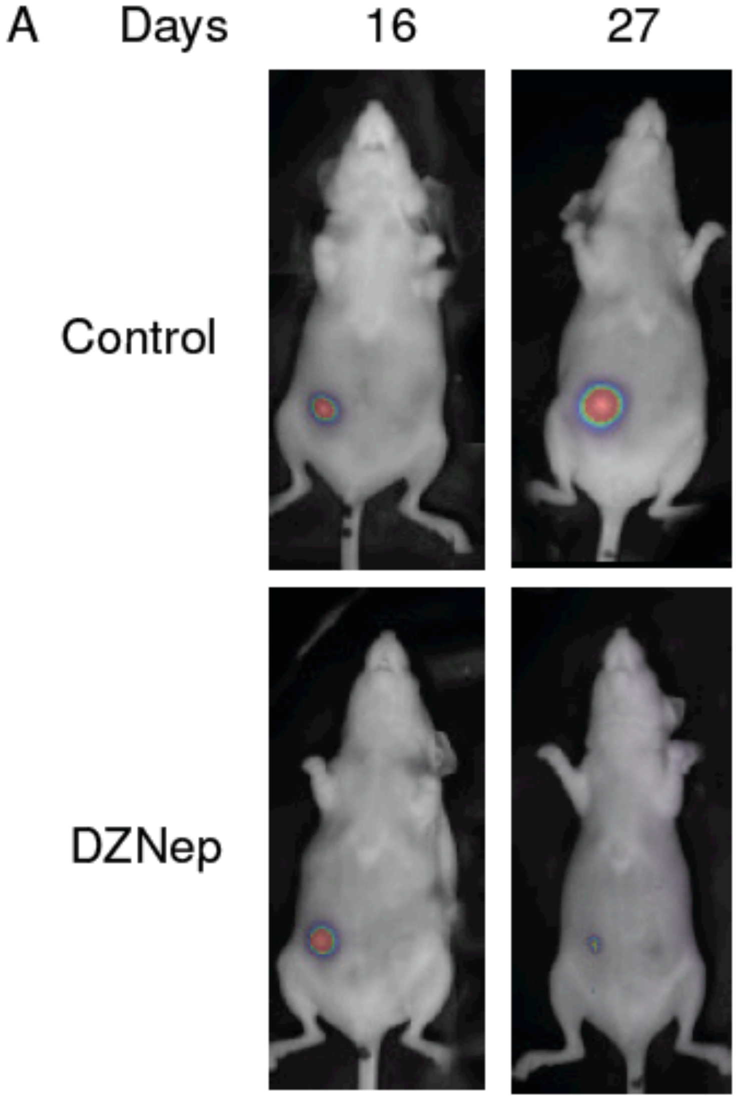


(**Fig.6A**).

**Figure 6A:** Inhibition of EZH2 affects GBM development in vivo. (**A**) 1 x $10^6$ U87-Fluc-mCherry glioblastoma cells were implanted s.c. in nude mice. Tumor growth was monitored by in vivo Fluc bioluminescence imaging. After implantation, one set of mice was injected i.v. with DZNep (0.07 mg/kg) and another set with PBS, at day 3, 5 and 7, followed by weekly injection. Mice treated with DZNep showed >70% reduced growth (**Fig.6A).**

## 8.8 HEPATOCELLULAR CARCINOMA (IN VIVO ANTICANCER ACTIVITY)

Chiba T et al. 3-Deazaneplanocin A is a promising therapeutic agent for the eradication of tumor-initiating hepatocellular carcinoma cells. Int J Cancer. 2012;130:2557-2567.

3-Deazaneplanocin A (DZNep) treatment inhibit HCC tumor growth in vivo. DZNep (1 and 5mg/Kg) administered twice a week suppressed tumor growth in a dose-dependent manner (greater than 30 and 85%respectively, p<0.05) (**Fig. 6B**).

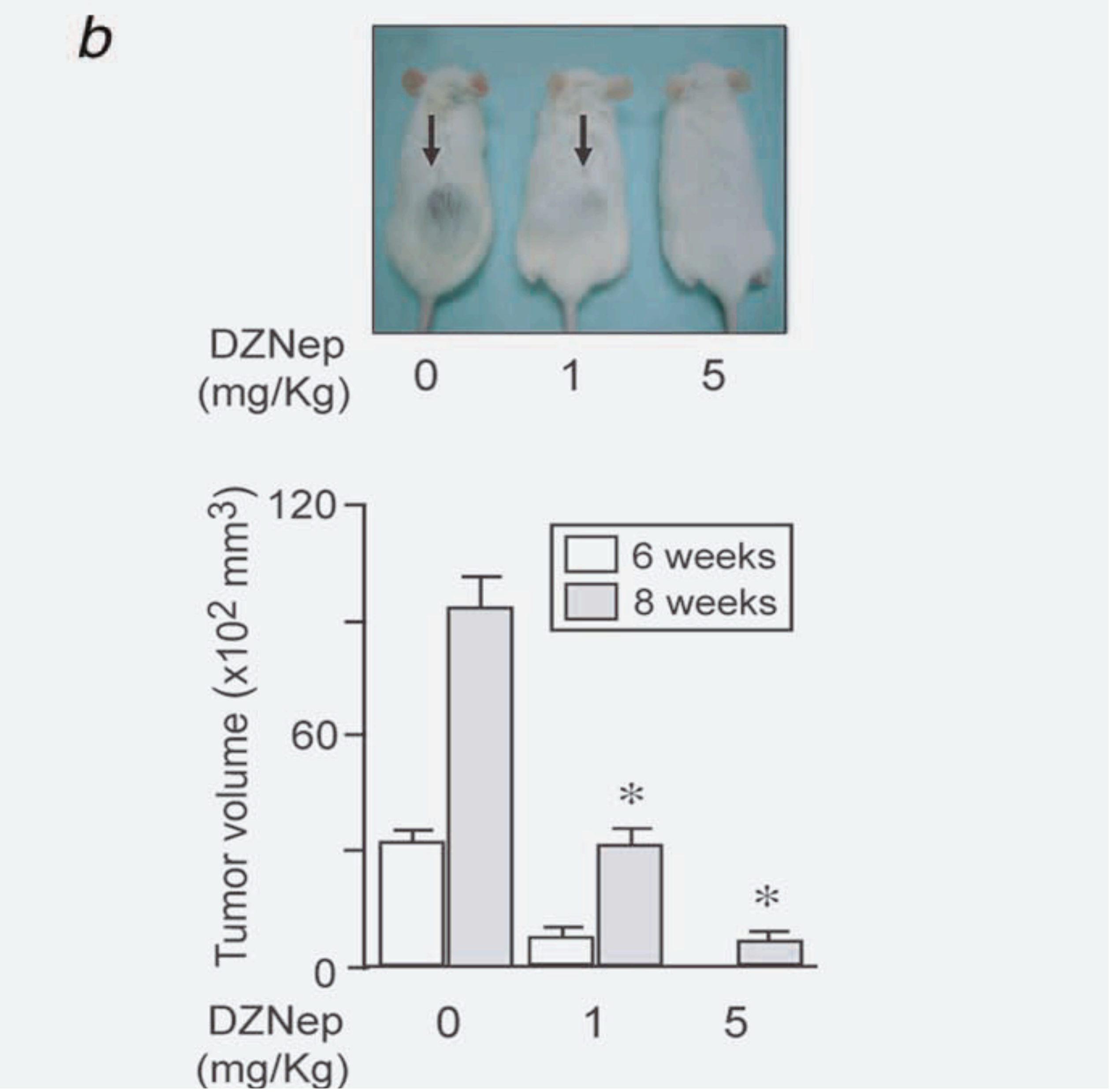


**Figure 6b** A total of 2 x $10^6$ Huh7 cells were transplanted into NOD/SCID mice. Tumor growth (arrows) was obviously suppressed by DZNep in a dose-dependent manner 6 weeks after the transplantation (upper panel. Tumor volume was determined at 6 and 8 weeks after transplantation (lower panel). *Statistically significant ($p < 0.05$).

## 8.9 MESOTHELIOMA (IN VIVO ANTICANCER ACTIVITY)

Kemp CD, Rao M, Xi S, Inchauste S, Mani H, Fetsch P, et al. Polycomb repressor complex-2 is a novel target for mesothelioma therapy. Clinical Cancer Res. 2012;18:77-90.

Experiments were carried out to examine whether DZNep could inhibit growth of established mesothelioma (MPM) xenografts. Preliminary experiments showed that the maximum tolerated dose of DZNep administered intraperitoneally in nude mice with tumor xenografts was 2.5 mg/kg twice a day. The nude mice were injected subcutaneously with MES1 cells sufficient to produce 100% tumor take at 7 days. Commencing on day 7, mice received 3 cycles of DZNep (2.5 mg/kg twice a day qd x3 q7d). This treatment regimen resulted in significant reduction of tumor size after each treatment cycle, with an approximate 50% reduction in tumor mass at the end of the treatment course and no visible systemic toxicity (**Fig. 5D**).

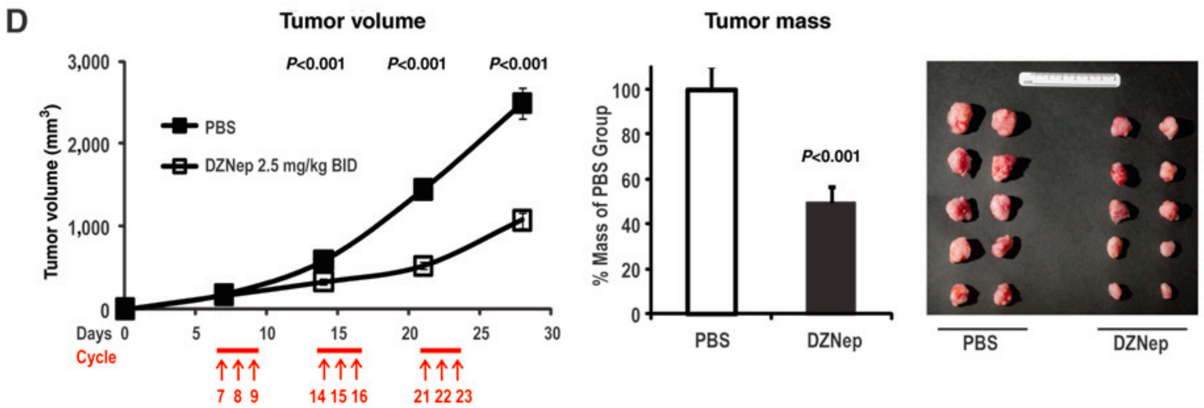


**Figure 5D,** Effects of intraperitoneal administration of DZNep on growth of established MES1 xenografts in nude mice.

## 8.10 MULTIPLE MYELOMA (IN VIVO ANTICANCER ACTIVITY)

Gaudichon J et al., Deazaneplanocin a is a promising drug to kill multiple myeloma cells in their niche. PLoS One. 2014;9(9):e107009.

To assess the activity of DZNep in vivo, the authors used the RPMI 8226-GFP-Luc MM cell line capable to graft into immunodeficient mice and to progress as myeloma tumor. Tumor localization and growth was monitored by non-invasive bioluminescence imaging. Moreover, at day 27 the DZNep-treated tumors stopped growing. The data demonstrated that, in vivo, DZNep delayed the engraftment and impaired the growth of MM cells in their physiological niche.

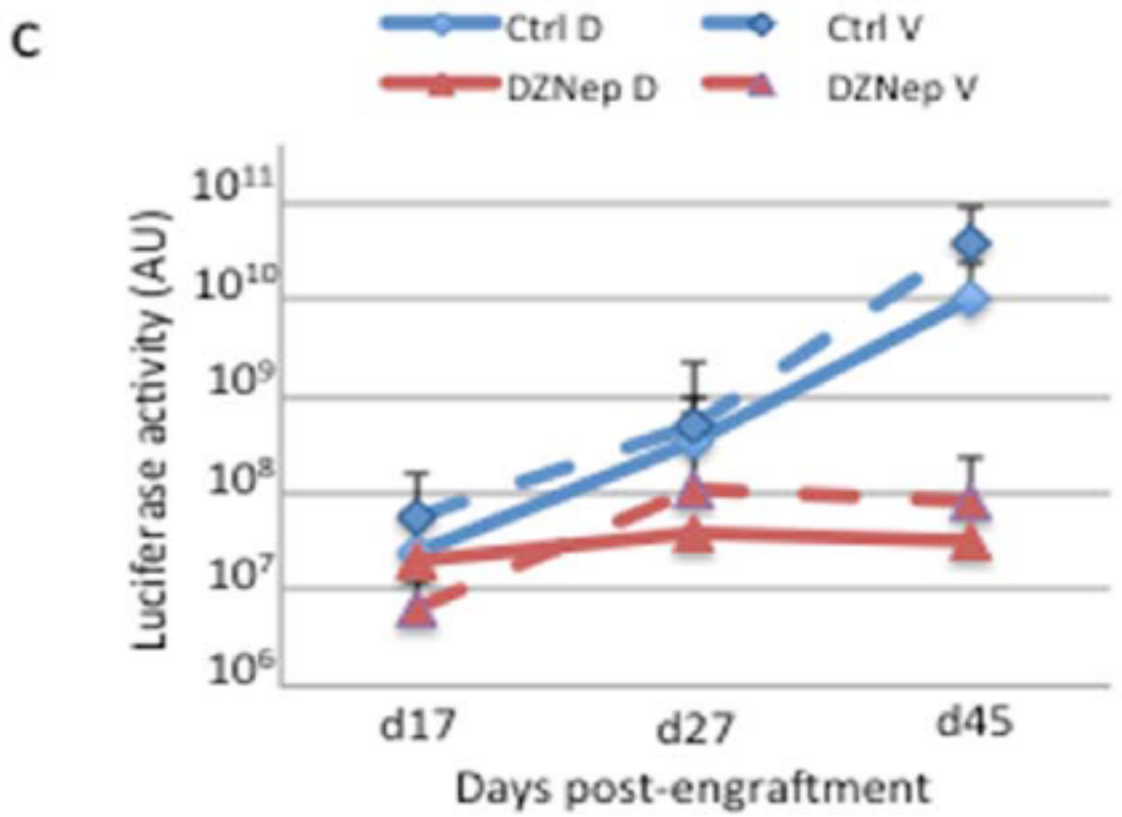


**Figure 5C**. DZNep delays the engrafment and impairs the growth of MM (multiple myeloma) cells in NSG mice. RPMI 8226-GFP-Luc cells were injected into the caudal vein of NSG mice (n = 10) at day 1 (5 x $10^6$ cells were injected per animal). Three days later, mice were separated into two groups (n = 5 in each group), one received vehicle for control; the other was treated with 100 µg DZNep (estimated dose 4 mg/kg) twice a week as indicated in the scheme. (c) At day 17, 27 and 45 the luciferase activity was determined.

**8.11 NEUROBLASTOMA (IN VIVO ANTICANCER ACTIVITY)**

Wang C et al., EZH2 Mediates Epigenetic Silencing of Neuroblastoma Suppressor Genes CASZ1, CLU, RUNX3, and NGFR. Cancer Res2011;72:315–**3**24.

Xenograft model: SMS-KCNR cells were resuspended in Hank's balanced salt solution and Matrigel (Trevigen) and a 100-mL cell suspension containing 2 x $10^6$ cells was placed in the subcutaneous tissue of the right flanks of 4- to 5-week-old nude mice (Taconic). Treatment was initiated when tumors reached approximately 100 mm3. Cohorts of mice (7 mice per group) received DZNep (2.5 mg/kg) or vehicle control (normal saline) twice daily, 3 days per week. The dimensions [length (L) and width (W)] of the tumors were assessed 3 times a week and the volume was calculated as (L x W2)/2. Student t test was used to compare the tumor volume between the DZNep and nontreatment groups, and P values less than 0.05 were considered significant. A preliminary study to explore whether DZNep inhibited the NB xenograft tumor growth indicates a statistically significant (P < 0.05) reduction in the growth of NB tumors in mice treated with DZNep (**Fig. 7F**).

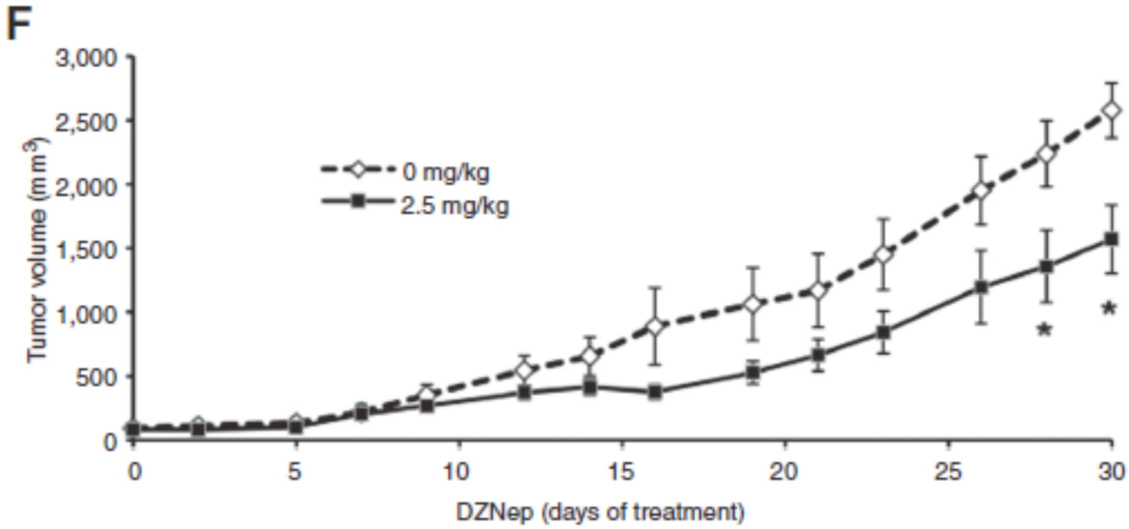


**Figure 7F**. Mice were treated with or without DZNep (2.5 mg/kg) twice a day, 3 days per week for 4 weeks. The mean tumor volumes are plotted using the SEM. The time points with significant differences (P < 0.05) are indicated with an asterisk.

## 8.12 PROSTATE CANCER (IN VIVO ANTICANCER ACTIVITY)

Wee ZN et al., EZH2-mediated inactivation of IFN-γ-JAK-STAT1 signaling is an effective therapeutic target in MYC-driven prostate cancer. Cell Rep. 2014;8(1):204-216.

The authors established DU145 prostate tumor xenografts in athymic mice and treated them with vehicle, DZNep, IFN-gamma, or both DZNep and IFN-gamma. Treatment with DZNep (1 mg/kg, s.c q2d) or IFN-gamma alone slowed down the tumor growth, while the combination treatment resulted in complete tumor growth arrest on average (**Figure 6A**). Throughout the study, both single and combination **treatments were well tolerated in mice without overt signs of toxicity or weight loss of >10%, supporting the potential application of this treatment in the clinic**.

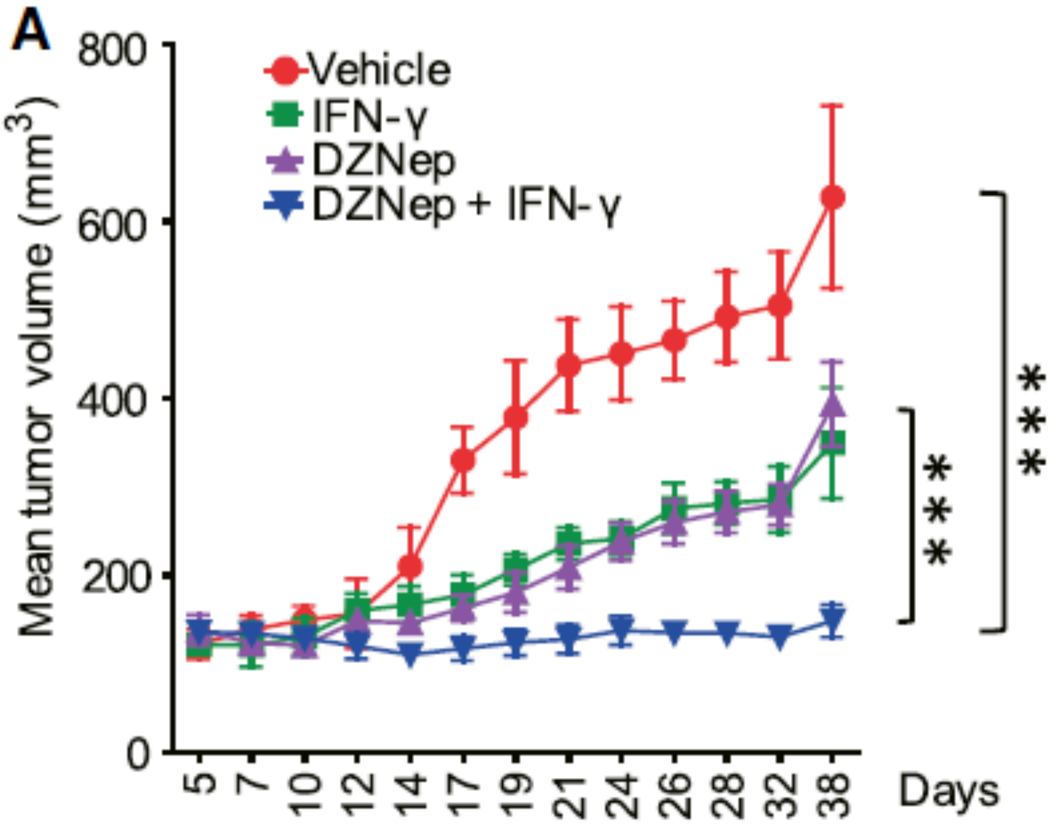


**Figure 6.** Combinatorial Antitumor Effects of DZNep and IFN-gamma In Vivo (**A**) DU145 xenograft prostate tumor growth in male athymic nude mice treated with vehicle (n = 5), IFN-g (1 x $10^7$ IU/kg, daily i.p.; n = 6), DZNep (1 mg/kg, s.c q2d.; n = 7), or both (n = 8). Mean tumor volume ± SEM is shown. q2d = every alternating day

# 9. SUMMARY TABLE OF IN VIVO ANTICANCER ACTIVITY

Momparler R & Côté S. Targeting of cancer stem cells by inhibitors of DNA and histone methylation. Expert Opin. Investig. Drugs 2015;24:8.

**Table 3.** ***In vivo*** **antineoplastic action of 3-deazaneplanocin-A (DZNep).**

| Type of cancer | Cell line | Mouse model | Dose schedule | Antineoplastic activity | Ref. |
|---|---|---|---|---|---|
| Leukemia | HL-60 | NOD/SCID | 1 mg/kg 2 days/wk × 2 wk | 19% increase survival | [47] |
| Leukemia | MOLM-14 | NOD/SCID | 2 mg/kg daily | 79% decrease tumor vol.<br>27% increase survival | [71] |
| Mantle cell lymphoma | Jeko-1 | NOD/CSID | 1 mg/kg × 2 wk | 50% decrease tumor vol. | [80] |
| Hepatic cancer | Huh 7 | NOD/SCID | 1 mg/kg 2x/wk<br>5 mg/kg 2x/wk | 67% decrease tumor vol.<br>91% decrease tumor vol. | [92] |
| Colon cancer (SC) | HT-29/$SOX2^{Hi}$ | NOD/SCID | 5 uM (*ex vivo*) | 50% decrease tumor vol. | [73] |
| Mesothelioma | MES-1 | Nude mice | 2.5 mg/kg b.d.<br>qdx3 q7d | 50% decrease tumor wt. | [86] |
| Glioblastoma | U87 | Nude mice | 0.07 mg/kg days 3, 5, 7<br>followed by 1 × q wk | 50% decrease tumor wt. | [93] |
| Glioblastoma | BT-1 CSC<br>BT-2 CSC<br>BT-3 CSC | Mice xenografts | 5 uM 5 days *ex vivo* | 50% increase survival | [35] |
| Prostate Cancer | DU-145 | Mice xenografts | 1 mg/kg every other day<br>over 38 days | 33% decrease tumor vol. | [91] |

CSC: Cancer stem cells; SC: Stem cells; vol: Volume; wk: Week; wt: Weight.

# 10. COMBINATION CHEMOTHERAPY

## 10.1 LEUKEMIA-1 (COMBINATION CHEMOTHERAPY)

Momparler RL et al., Synergistic antileukemic action of a combination of inhibitors of DNA methylation and histone methylation. Leuk Res. 2012;36:1049-1054.

A remarkable antineoplastic synergy was observed against HL-60 myeloid leukemic cells by 5AZA-CdR (5AZA) in combination with DZNep (DZN) (**Fig. 1**)

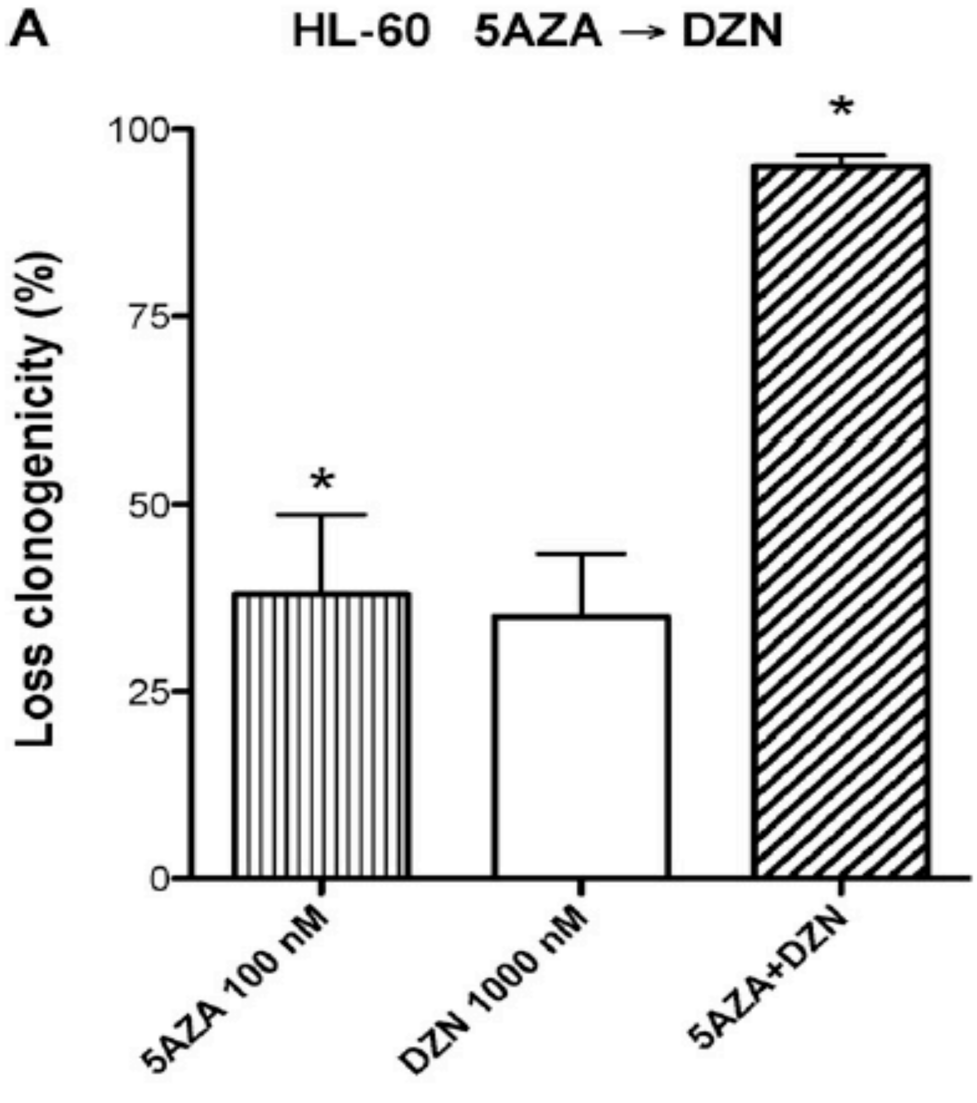


**Figure. 1**. Clonogenic assay of leukemic cells after sequential treatment with 5-AZACdR (5AZA) and DZNep (DZN). (A) HL-60 cells were treated with 100 nM 5AZA and/or at 24 h 1000 nM DZN was added to the medium. At 48 h cells were placed in soft agar medium for colony assay. The results are expressed as mean ± SEM, n = 3.

A remarkable synergistic activation of genes was observed against HL-60 myeloid leukemic cells by 5AZA-CdR (5AZA) in combination with DZNep (DZN) (**Fig. 2**).

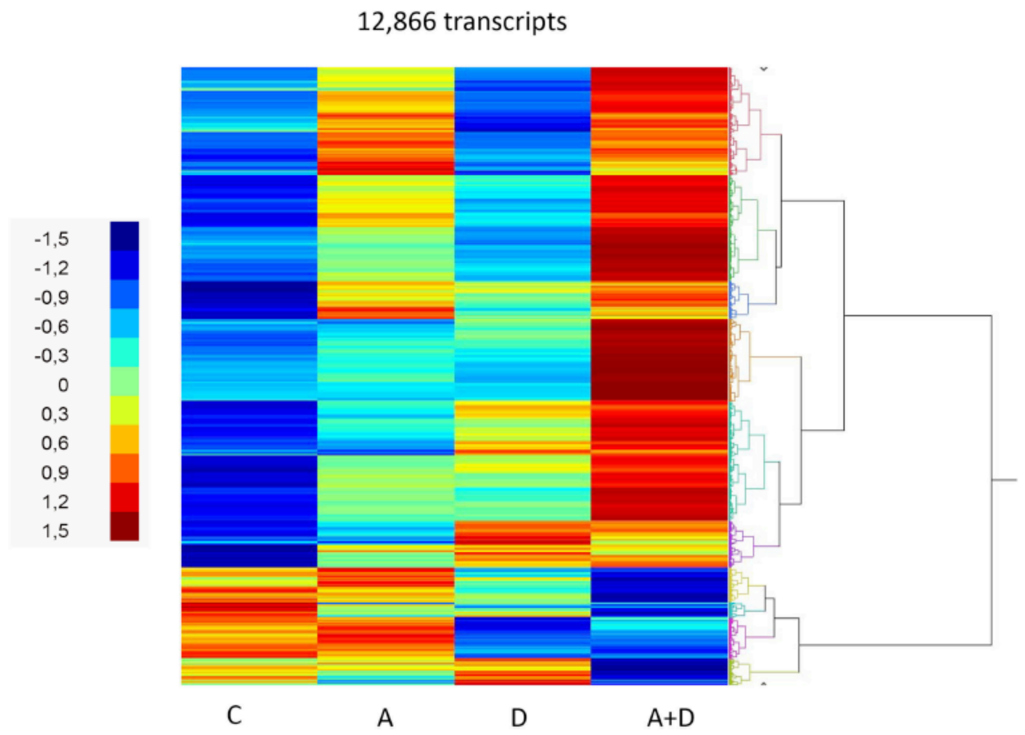


**FIGURE 2 | RNA sequence analysis of global gene expression in HL-60 leukemic cells after treatment with 5-AZA-CdR and/or DZNep.** Heat map of expression of transcripts. HL-60 leukemic cells were treated with 100 nM 5-AZA-CdR (A). At 24 h 1,000 nM DZNep (D) was added to the medium. Drug removal at 48 h. RNA isolation at 72 h as described in Section "Materials and Methods." The pairwise comparisons were control (C) versus A; C versus D; and C versus A + D.

**Figure 2** shows the heat map displaying relative transcript abundances of 12,866 transcripts, each of which is differentially expressed (FDR 5%) at least in one of the pairwise comparisons of the four conditions. Red color, relatively high expression; blue color, relatively low expression. Relative to the control sample, 5-AZA-CdR alone showed a greater increase in the expression of transcripts as compared to DZNep. DZNep increased the activation of many cohorts of genes. The combination of 5-AZA-CdR plus DZNep showed a synergistic increase in expression of hundreds of genes.

## 10.2 LEUKEMIA-2 (COMBINATION CHEMOTHERAPY)

Fiskus W et al. Combined epigenetic therapy with the histone methyltransferase EZH2 inhibitor 3-deazaneplanocin A and the histone deacetylase inhibitor panobinostat against Human AML cells . Blood 2009 114: 2733-2743.

The in vitro antineoplastic activity of DZNep in combination with panobinostat (PS) was investigated in cultured human acute myeloid leukemia (AML) cells: HL-60 and OCI-AML3. DZNep plus PS induced a higher level differentiation of HL-60 and OCI-AML3 cells than either agent alone. The duration of treatment was 72 h (Figure 6C)

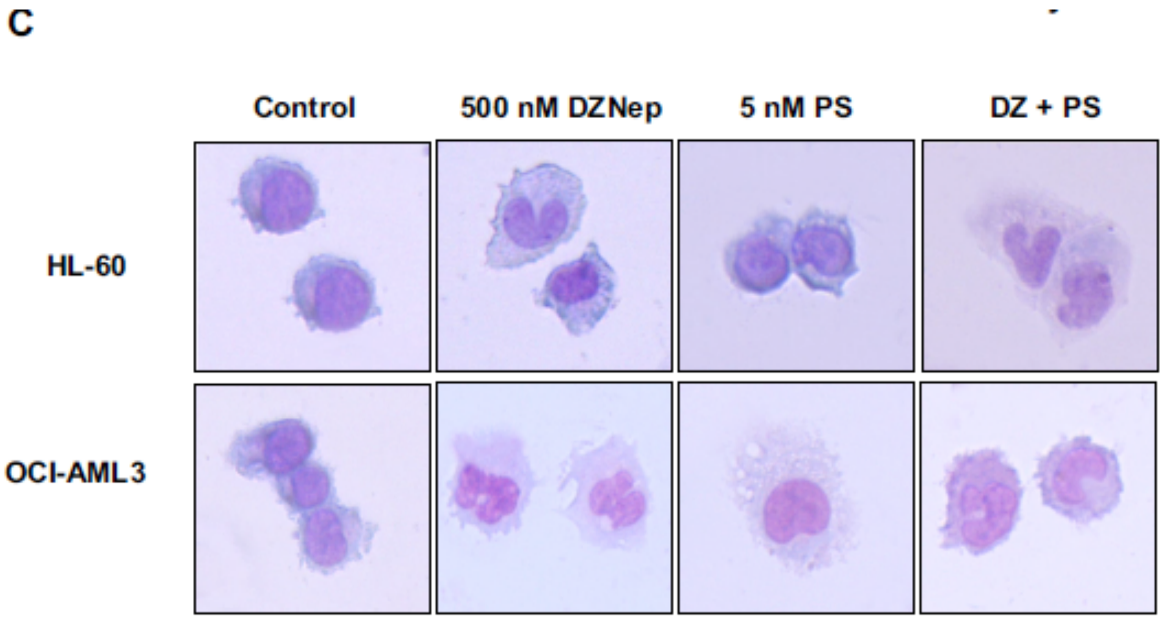


**Figure 6C**: HL-60 and OCI-AML3 cells were treated with the indicated concentrations of DZNep and/or PS (panobinostat) for 72 hours. After treatment, the cells were cytospun onto glass slides, Wright stained, and observed with a microscope to assess cellular morphology.

DZNep plus PS induced a higher level apoptosis of HL-60 and OCI-AML3 cells than either agent alone. (**Figure 4A**)

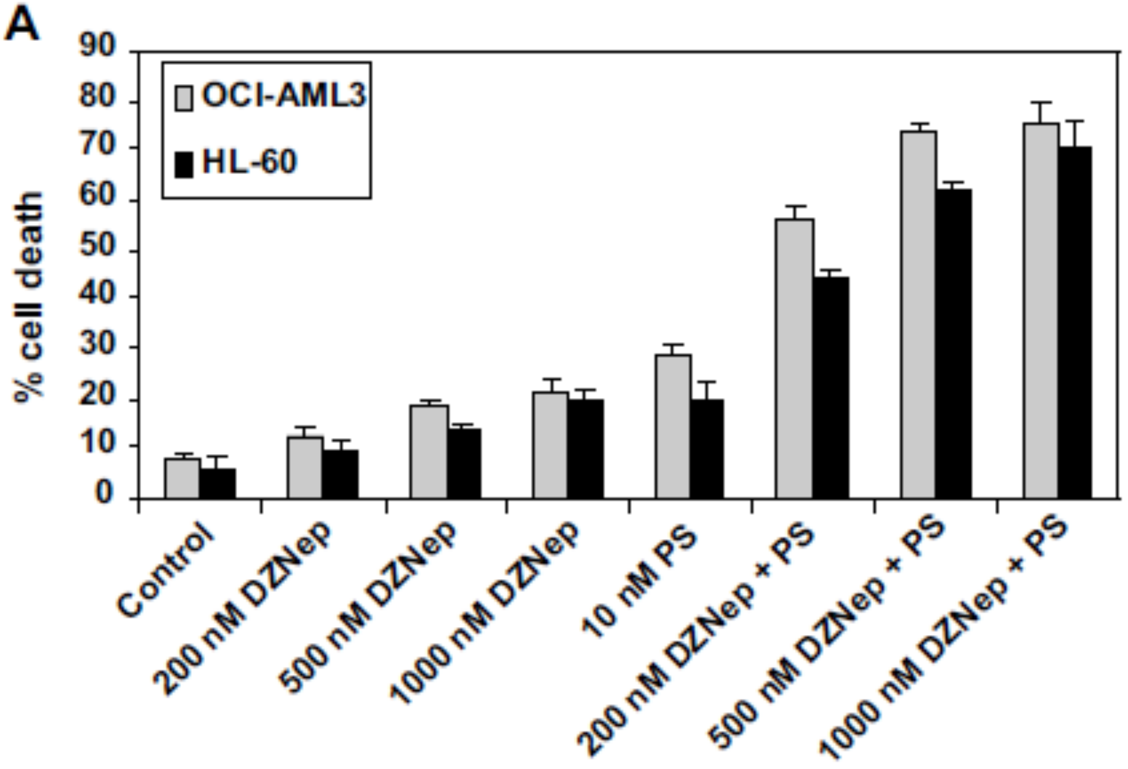


**Figure 4A**. Cotreatment with PS and DZNep synergistically induces apoptosis of cultured AML cells. (A) OCI-AML3 and HL-60 cells were treated with DZNep and/or PS as indicated for 48 hours. At the conclusion of treatment, cell death was assessed by trypan blue dye uptake in a hemocytometer. Bars on columns represent SEM.

DZNep plus PS increased the survival time of mice injected with HL-60 leukemic cells to a greater extent than either agent alone. (**Figure 4D**)

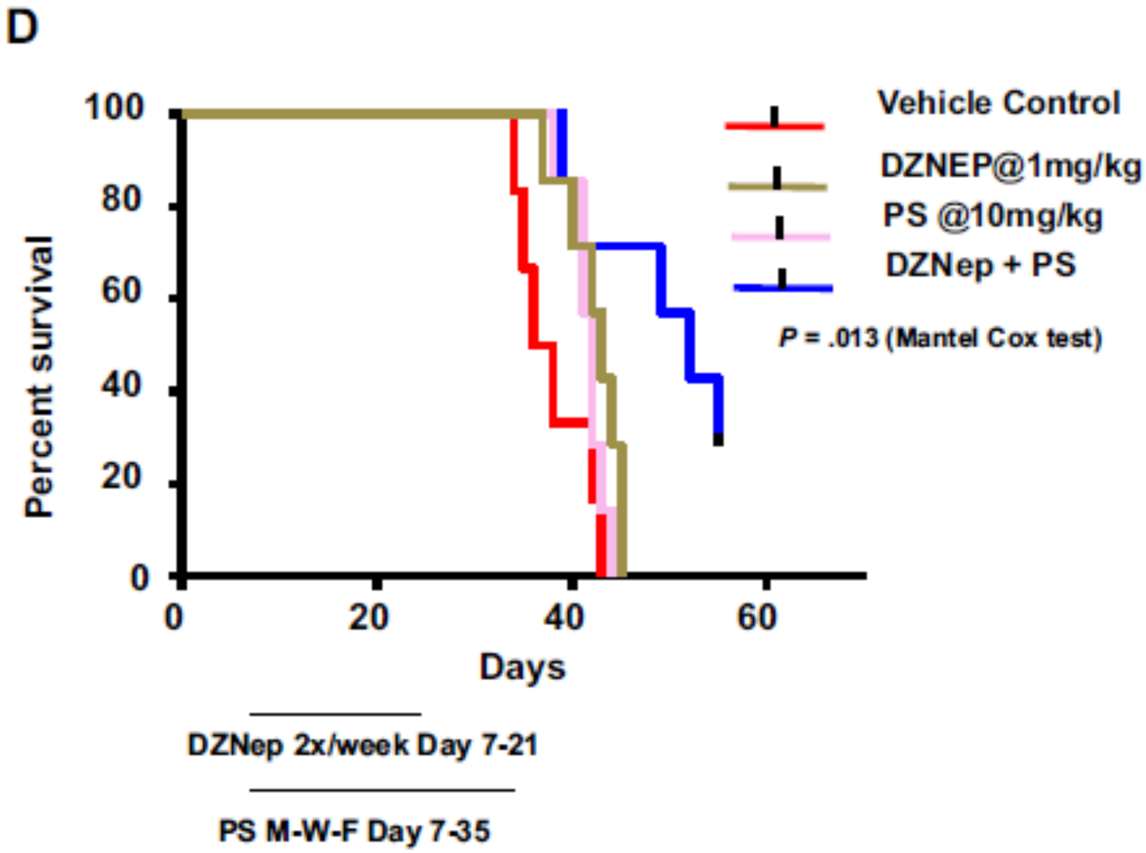


**Figure 4 (D)** Female NOD/SCID mice were injected in the lateral tail vein with HL-60 cells. The cells were allowed to engraft for 7 days before initiation of treatment. Mice were treated intraperitoneally with dimethylsulfoxide, 1 mg/kg DZNep 2 days per week for 2 weeks, and/or 10 mg/kg PS 3 days per week for 4 weeks. n=7 per group. Survival of the mice in all groups (vehicle, DZNep alone, PS alone, and combination) is represented by Kaplan-Meier plot.

## 10.3 LEUKEMIA-3 (COMBINATION CHEMOTHERAPY)

Momparler RL, Côté S, Momparler LF, et al. Epigenetic therapy of acute myeloid leukemia using 5-aza-2'-deoxycytidine (decitabine) in combination with inhibitors of histone methylation and deacetylation. Clinical Epigenetics 2014;6:19.

Myeloid leukemic cell lines: HL-60 and AML-3 were treated with 5-AZA-CdR, DZNep and/or the histone deacetylase inhibitor, trichostatin A (TSA). The triple combination of these agents was more effective to reduce cell survival and increase apoptosis than the double combinations or the single agents.

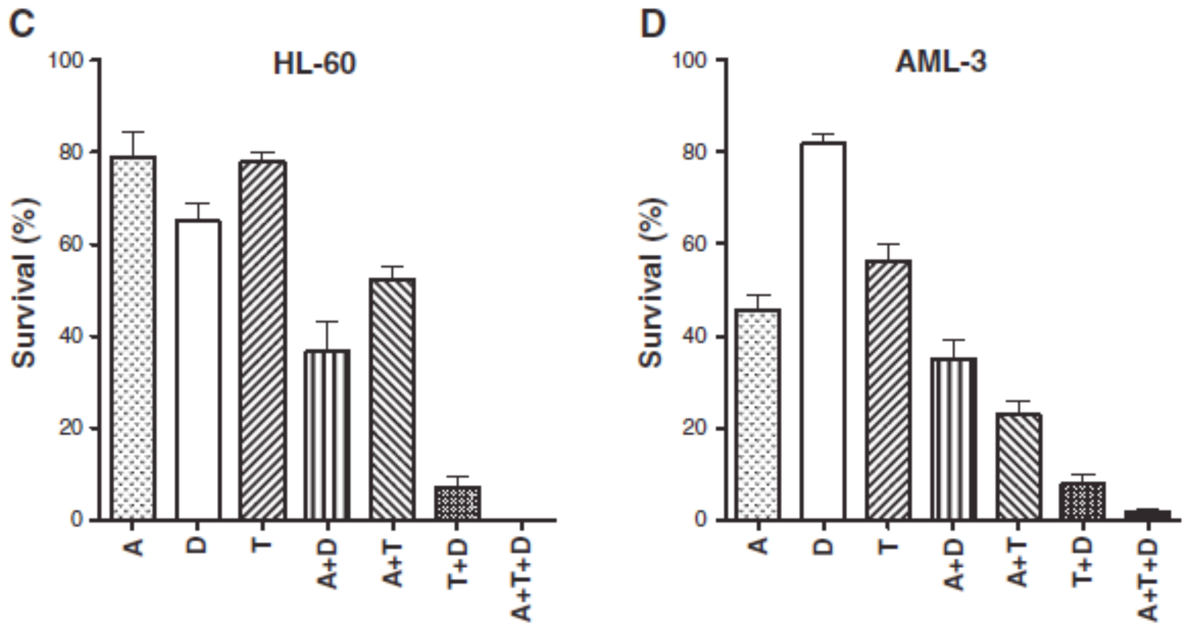


**Figure 1CD** Colony assay of leukemic cells after sequential treatment with 5-AZA-CdR (A), DZNep (D), and/or TSA (T). The leukemic cells were treated with 20 nM 5-AZA-CdR and, at 24 h, 500 nM (AML-3) or 1,000 nM (HL-60) DZNep and/or 40 nM (AML-3) or 80 nM (HL-60) nM TSA were added to the medium. At 48 h the cells were counted and placed in soft agar for colony assay to determine cell survival. The results are expressed as mean ± SEM, n = 3.

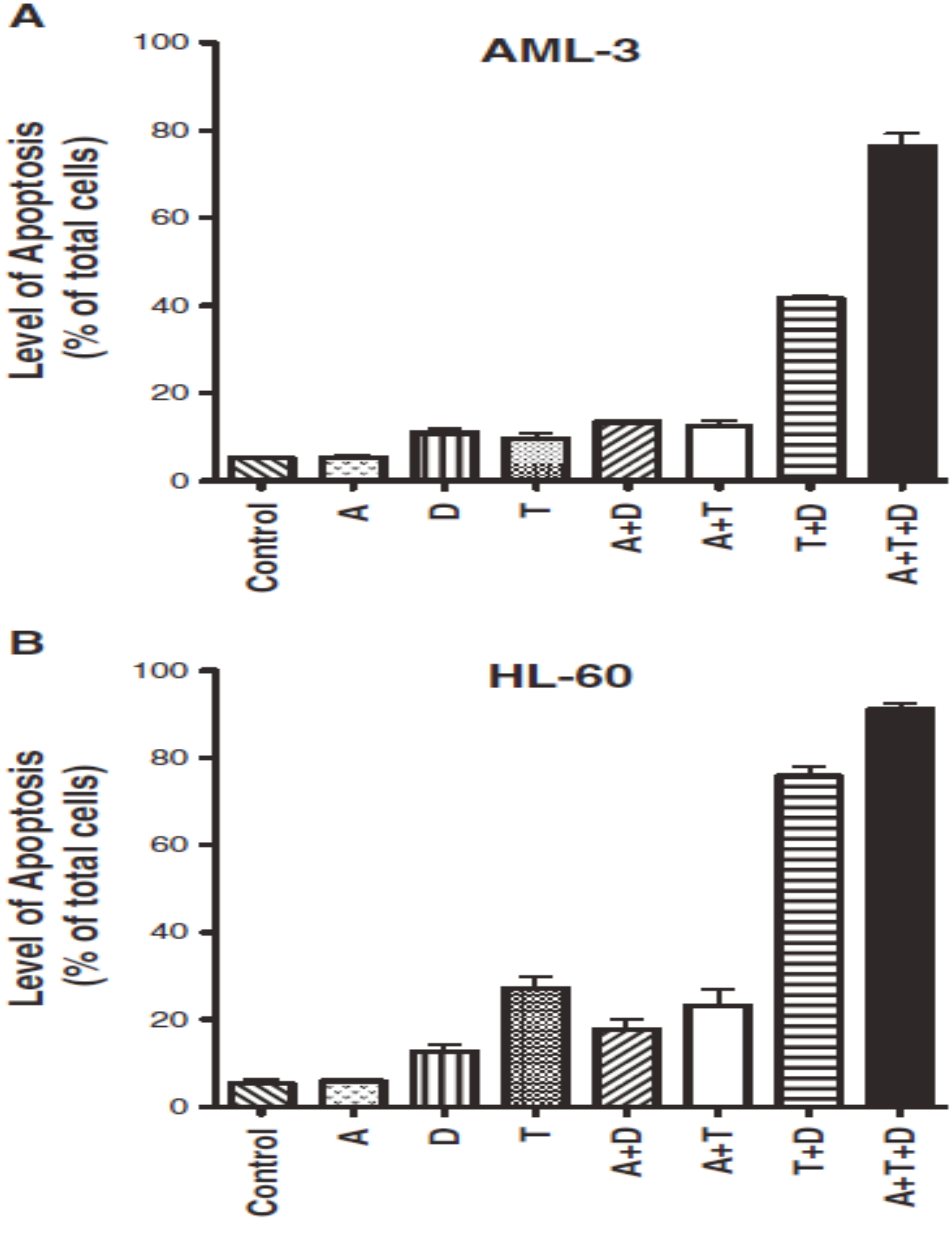


**Figure 2** Induction of apoptosis of leukemic cells after sequential treatment with 5-AZA-CdR (A), DZNep (D), and/or TSA (T). AML-3 cells (**A**) and HL-60 cells (**B**) were treated with 20 nM 5-AZA-CdR and, at 24 h, 500 nM (AML-3) or 1,000 nM (HL-60) DZNep and/or 40 nM (AML-3) or 80 nM (HL-60) nM TSA were added to the medium. At 48 h the drugs were removed and at 72 h the cells were analyzed for induction of apoptosis using Annexin V staining. The results are expressed as mean ± SEM, n = 3.

## 10.4 LUNG CANCER-1 (COMBINATION CHEMOTHERAPY)

Nascimento ASF et al., Synergistic antineoplastic action of 5-aza-2'deoxycytidine (decitabine) in combination with different inhibitors of enhancer of zeste homolog 2 (EZH2) on human lung carcinoma cells. J Cancer Res Ther. 2016, 4(5):42-49.

The anticancer action on A549 lung tumor cells of 5-AZA-CdR in combination with the EZH2 inhbitors: DZNep, Gsk-126 or U-4149 was greater than the anticancer action exhibited by single agent treatment.

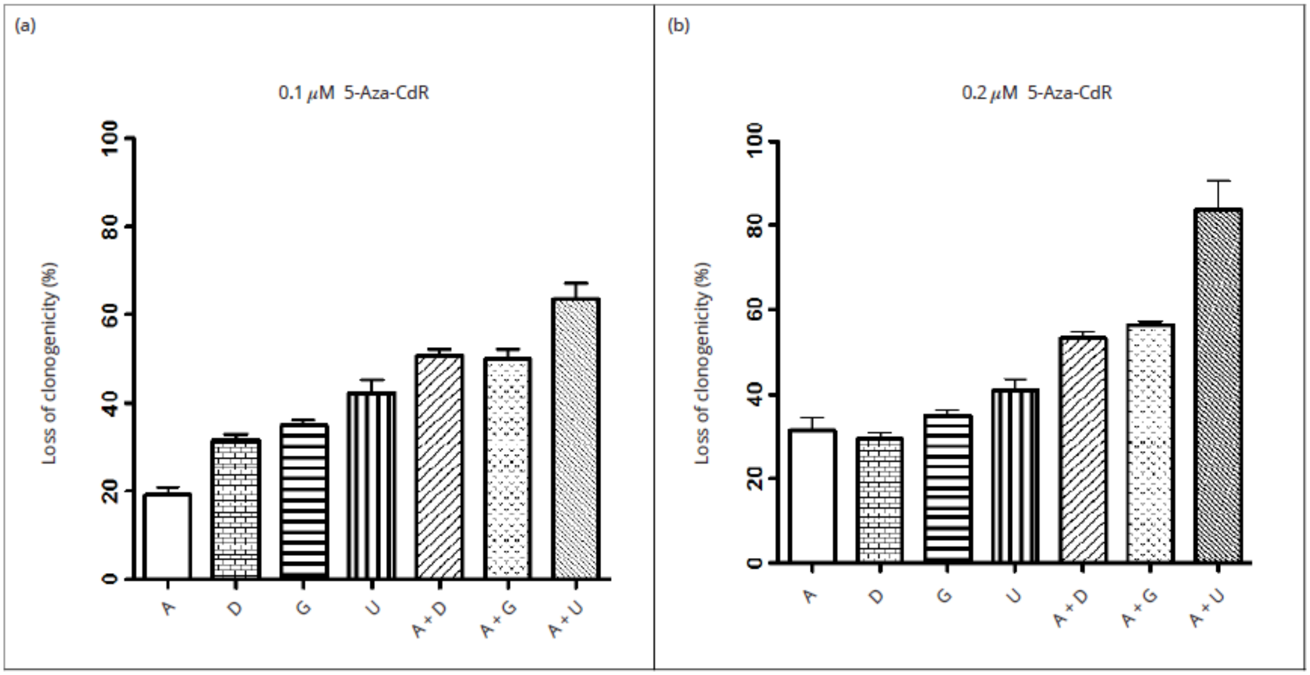


**Figure 3a,b** Colony assay of A549 cells after treatment with 5-Aza-CdR (A), DZNep (D), Gsk-126 (G) and U-4149 (U). Cells were treated at 0 h with 0.1 M 5-Aza-CdR (a) or 0.2 M 5-Aza-CdR (b) alone or in sequential combination by the addition at 24 h of: 1 M DZNep, 2.5 M Gsk-126 or 1 M U-4149. At the end of drug treatment (48 h), the cells were incubated in drug-free medium. The number of colonies was counted after 16-18 days of incubation and the loss of clonogenicity (%) was expressed relative to control cells. Data shown: mean values SEM, n 3.

## 10.5 LUNG CANCER-2 (COMBINATION CHEMOTHERAPY)

Fillmore CM et al., EZH2 inhibition sensitizes BRG1 and EGFR mutant lung tumours to TopoII inhibitors. Nature. 2015;520(7546):239-242.

To determine if the protected and sensitized phenotypes could be observed in vivo, the xenograft-bearing mice were treated with etoposide +/− DZNep. For the sensitized cell line H157, treatment with etoposide and DZNep therapy prevented tumors from forming in 4/6 mice and more efficacious than etoposide or DZNep alone (**Fig. 2a**) In contrast, the protected H23 xenografts that received early dual therapy grew significantly larger than those treated with either DZNep or etoposide alone (**Fig. 2b**).

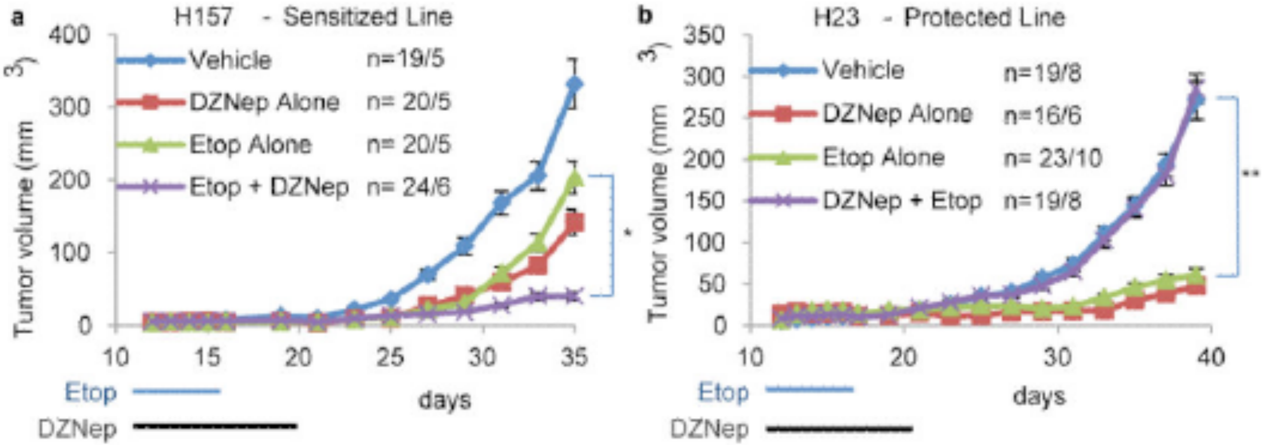


**Figure 2a,b** In vitro sensitivities to DZNep + TopoII inhibition predict in vivo responses
Either the H157 (**a**) or H23 (**b**) NSCLC cell line was injected into the flanks of Nude mice and tumors were allowed to form. On day 12, mice were randomly segregated into cohorts that received either placebo, DZNep, etoposide or dual therapy for 2 weeks, and tumor size +/− s.e.m.

**10.6 PROSTATE CANCER (COMBINATION CHEMOTHERAPY)**

Wee ZN et al., EZH2-mediated inactivation of IFN-γ-JAK-STAT1 signaling is an effective therapeutic target in MYC-driven prostate cancer. Cell Rep. 2014;8(1):204-216.

The authors established DU145 prostate tumor xenografts in athymic mice and treated them with vehicle, DZNep, IFN-gamma, or both DZNep and IFN-gamma. Treatment with DZNep or IFN-gamma alone slowed down the tumor growth, while the combination treatment resulted in complete tumor growth arrest on average (**Figure 6A**). Throughout the study, both single and combination **treatments were well tolerated in mice without overt signs of toxicity or weight loss of >10%, supporting the potential application of this treatment in the clinic**.

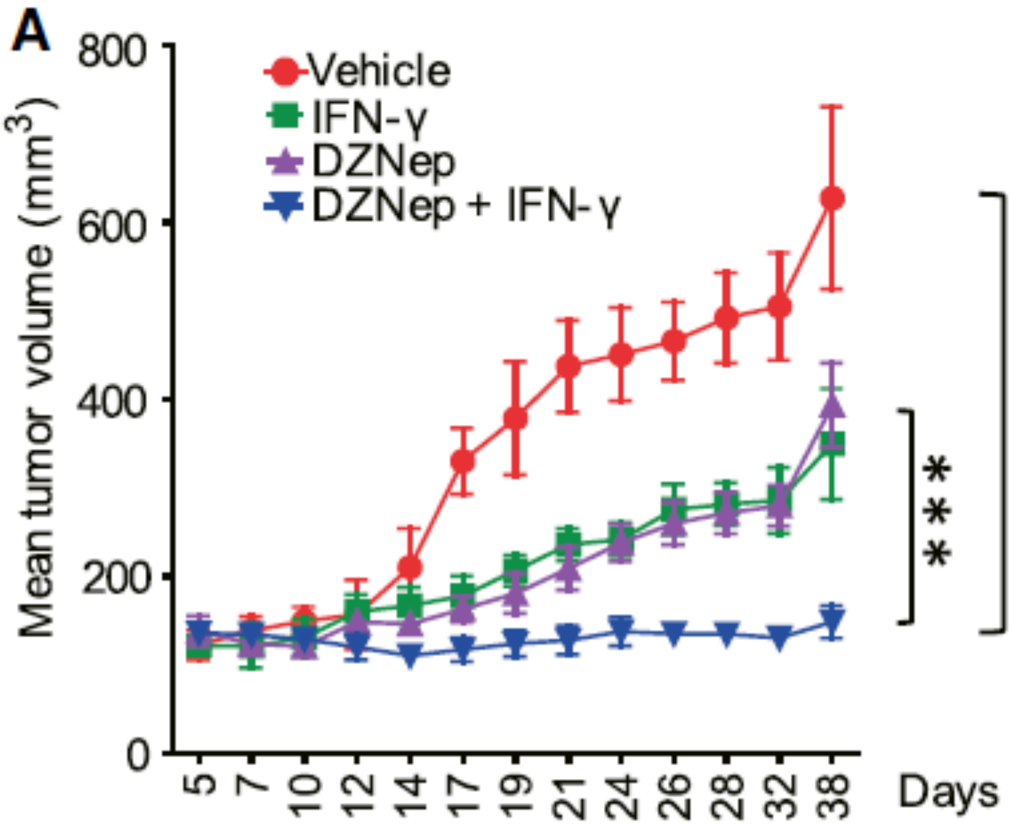


**Figure 6.** Combinatorial Antitumor Effects of DZNep and IFN-gamma In Vivo (**A**) DU145 xenograft prostate tumor growth in male athymic nude mice treated with vehicle (n = 5), IFN-g (1 x $10^7$ IU/kg, daily i.p.; n = 6), DZNep (1 mg/kg, s.c q2d.; n = 7), or both (n = 8). Mean tumor volume ± SEM is shown. q2d = every alternating day

## 10.7 OVARIAN CANCER (COMBINATION CHEMOTHERAPY)

Peng D et al., Epigenetic silencing of TH1-type chemokines shapes tumour immunity and immunotherapy. Nature. 2015;527:249-253.

EZH2-mediated histone H3 lysine 27 trimethylation (H3K27me3) and DNA methyltransferase 1 (DNMT1)-mediated DNA methylation repress the tumour production of T helper 1 (TH1)-type chemokines CXCL9 and CXCL10, and subsequently determine effector T-cell trafficking to the tumour microenvironment. Treatment with epigenetic modulators (5AZA-CdR & DZNep) removes the repression and increases effector T-cell tumour infiltration, slows down tumour progression, and improves the therapeutic efficacy of programmed death-ligand 1 (PD-L1).

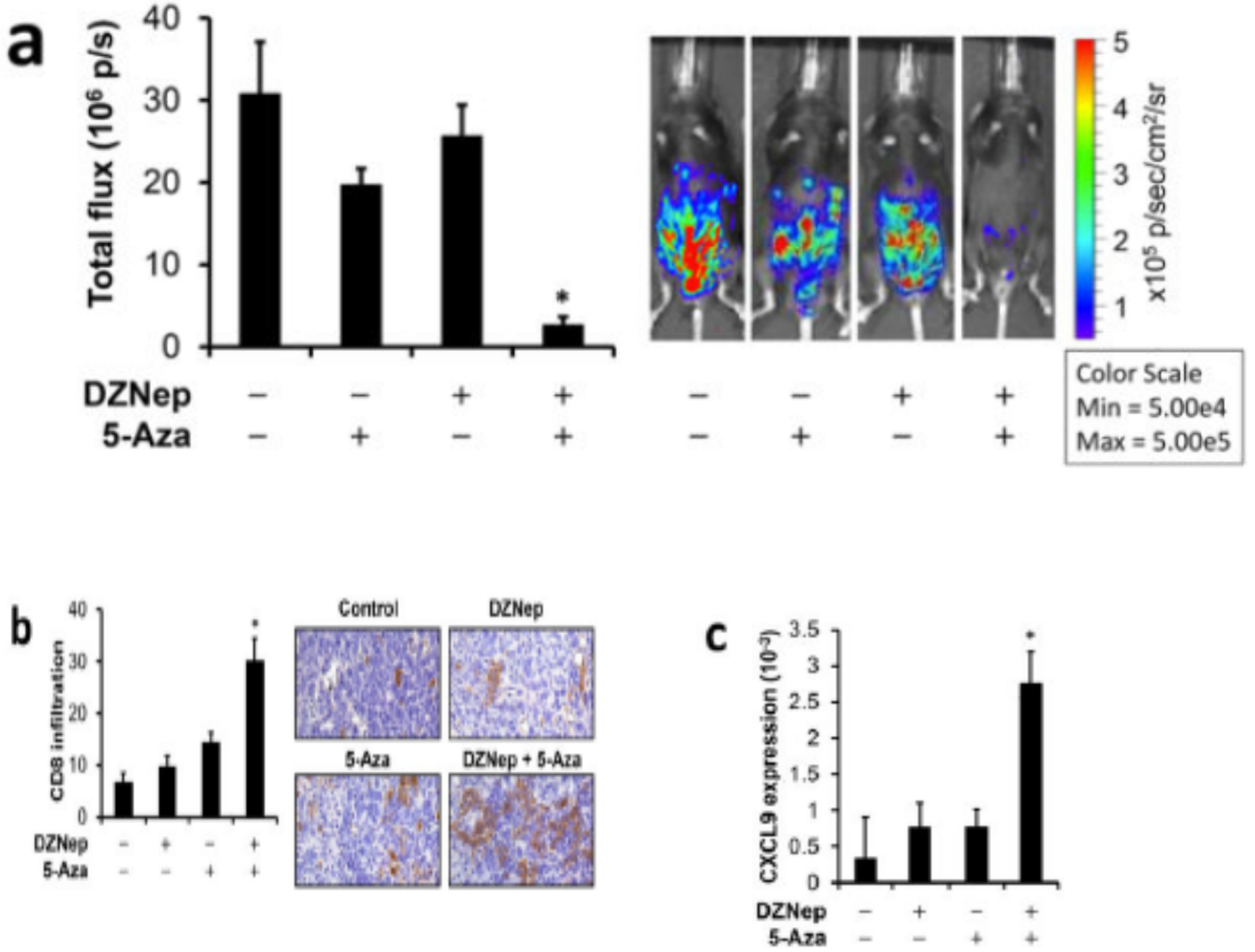


**Figure 1. Epigenetic reprogramming alters immunotherapy**

<u>**a–c,** Effects of DZNep and 5-AZA dC on ID8 mouse ovarian cancer progression</u>. The ID8 tumor bearing mice (C57BL/6) were treated with DZNep and 5-AZA dC. **(a)** Tumor growth was recorded by Bioluminescence imaging. The representative images and tumor volume at day 24 are shown. Day 0: tumor inoculation. **(b)** Tumor-infiltrating CD8+ T cells were quantified by immunohistochemistry staining (IHC). **(c)** Tumor CXCL9 mRNA was quantified by real-time PCR.

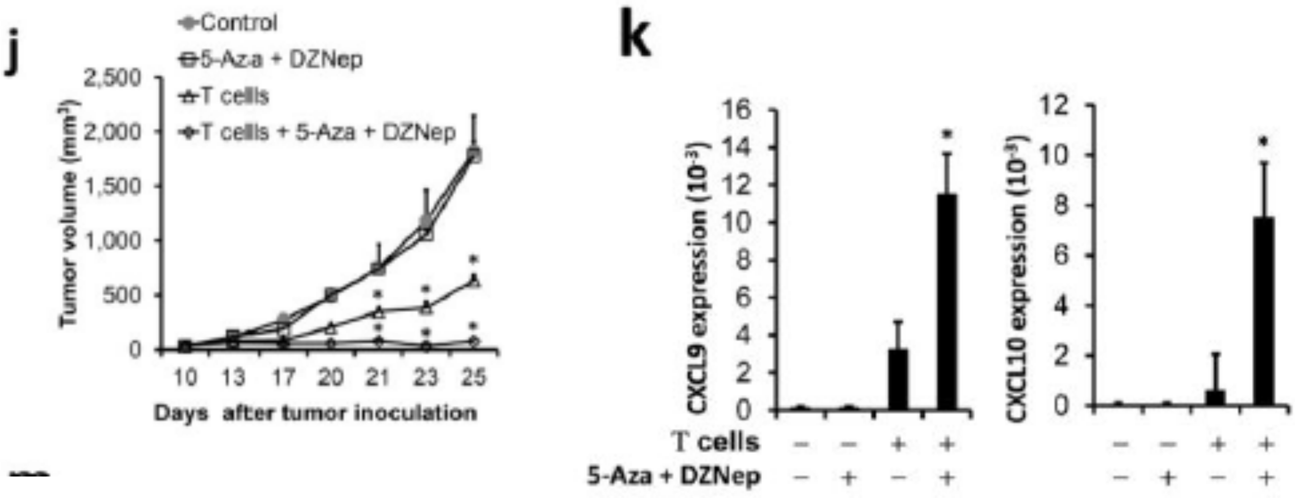


**Figure 1: j–l**, Effects of DZNep and 5-AZA-CdR on T cell immunotherapy. Autologous human tumor-specific human CD8+ T cells were transfused into ovarian cancer-bearing NOD-scid IL2Rγc null (NSG) mice with or without DZNep and 5-AZA-CdR treatment. The mice were treated with anti-CXCR3. Tumor volume was monitored (**j**). Th1-type chemokine expression was quantified by real-time PCR (**k**). (mean/SEM, n = 5 per group, * P < 0.05 Mann-Whitney Test).

DZNep in combination with IFNγ increased remarkably the expression of CXCL9 and CXCL10 in human primary ovarian cancer cells (b). DZNep reduced the levels of EZH2 and H3K27me3 (f).

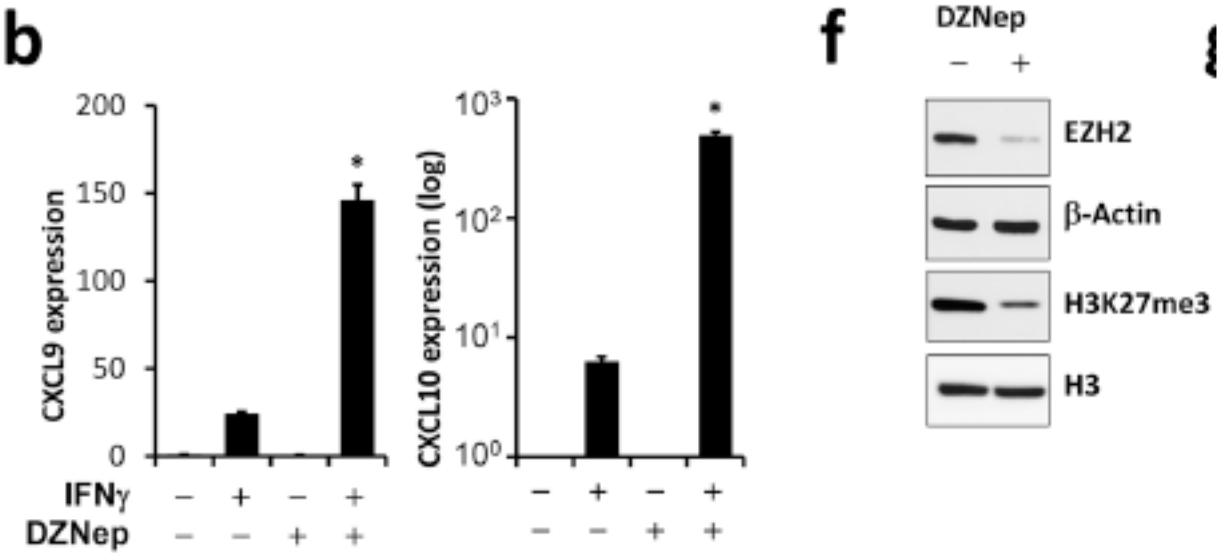


**Figure 2: b** Effects of DZNep on ovarian cancer Th1-type chemokine expression. Human primary ovarian cancer cells were treated for 24 (b) hours with DZNep in the presence of IFNγ. CXCL9 and CXCL10 expression were quantified by real-time PCR (b). **f**, Effects of DZNep on the expression of EZH2 and H3K27me3. Primary ovarian cancer cells were treated with or without DZNep for 24 hours. The levels of EZH2, H3K27me3 were detected by Western blotting.

# 11. BIOCHEMICAL PHARMACOLOGY: GENE EXPRESSION

## 11.1 LEUKEMIA (GENE EXPRESSION)

Momparler RL et al., Inhibition of DNA and Histone Methylation by 5-Aza-2'-deoxycytidine (Decitabine) and 3-Deazaneplanocin-A on Antineoplastic Action and Gene Expression in Myeloid Leukemic Cells. Frontiers Oncol 2017;7:19.

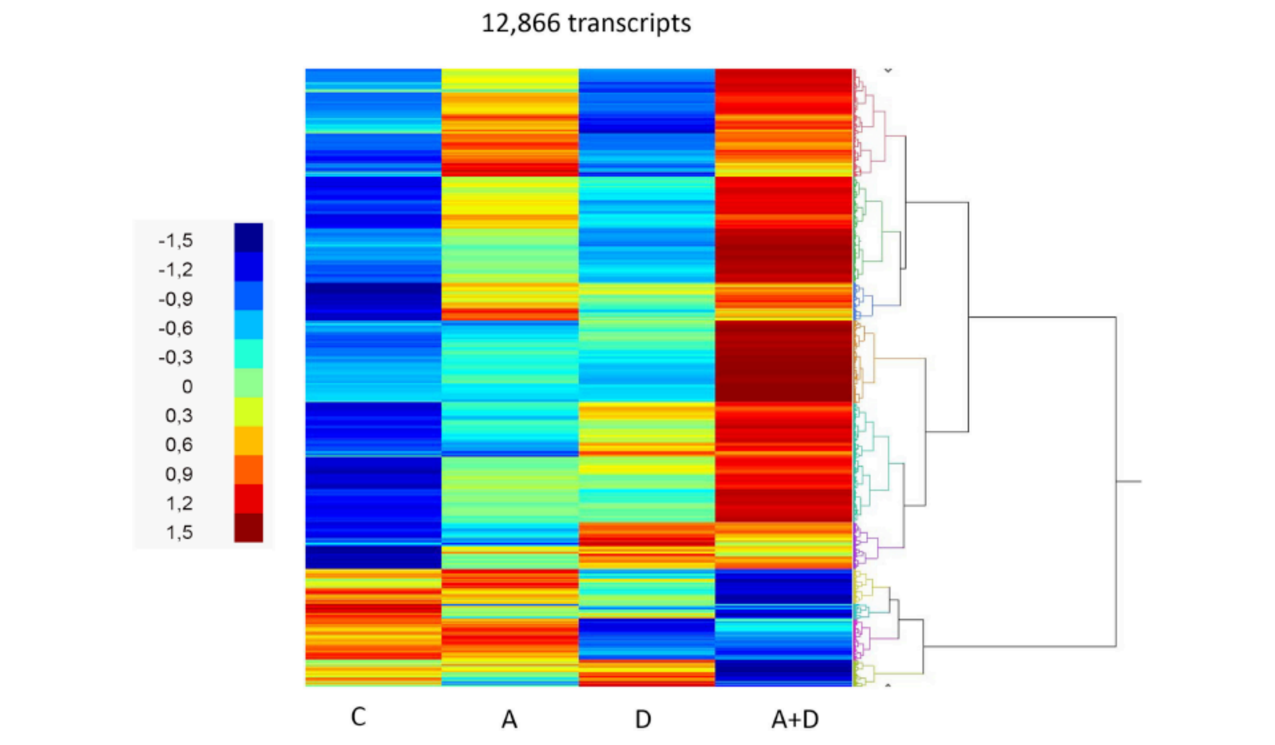


**FIGURE 2 | RNA sequence analysis of global gene expression in HL-60 leukemic cells after treatment with 5-AZA-CdR and/or DZNep.** Heat map of expression of transcripts. HL-60 leukemic cells were treated with 100 nM 5-AZA-CdR (A). At 24 h 1,000 nM DZNep (D) was added to the medium. Drug removal at 48 h. RNA isolation at 72 h as described in Section "Materials and Methods." The pairwise comparisons were control (C) versus A; C versus D; and C versus A + D.

**Figure 2** shows the heat map displaying relative transcript abundances of 12,866 transcripts, each of which is differentially expressed (FDR 5%) at least in one of the pairwise comparisons of the four conditions. Red color, relatively high expression; blue color, relatively low expression. Relative to the control sample, 5-AZA-CdR alone showed a greater increase in the expression of transcripts as compared to DZNep. DZNep increased the activation of many cohorts of genes. The combination of 5-AZA-CdR plus DZNep showed a synergistic increase in expression of hundreds of genes.

## 11.2 BREAST CANCER (GENE EXPRESSION)

Tan J et al. Pharmacologic disruption of Polycomb-repressive complex 2-mediated gene repression selectively induces apoptosis in cancer cells. Genes Dev. 2007;21:1050-1063.

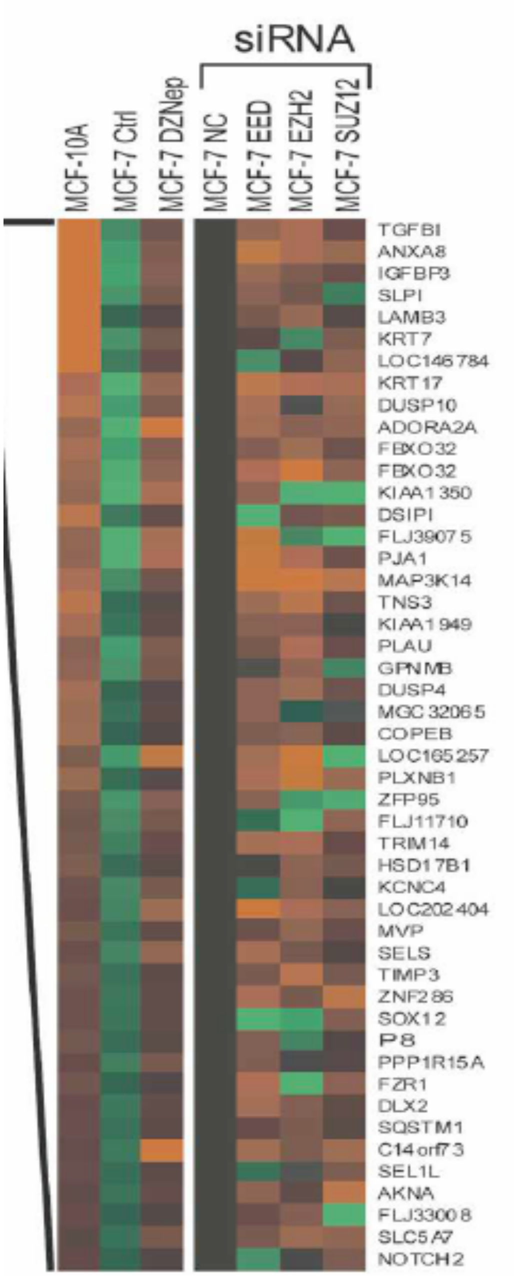


**Figure 3B.** Identification of DZNep-activated PRC2 target genes in breast cancer cells.

Cells were treated with DZNep or individual EZH2, EED, or SUZ12 siRNA for 72 h and gene expression was performed using the Illumina 24K BeadArray system. Genes (751) were up-regulated for twofold or greater upon DZNep treatment. Cluster diagram showing the PRC2 targets expressed differentially in MCF-7 and MCF-10A cells. Genes are rank-ordered by levels of high expression (red) and low expression (green) in MCF-7 cells relative to MCF-10A cells.

# 12. BIOCHEMICAL PHARMACOLOGY: PROTEIN EXPRESSION

## 12.1 LEUKEMIA (PROTEIN EXPRESSION)

Fiskus W et al. Combined epigenetic therapy with the histone methyltransferase EZH2 inhibitor 3-deazaneplanocin A and the histone deacetylase inhibitor panobinostat against Human AML cells . Blood 2009 114: 2733-2743

The in vitro activity of DZNep on protein expression was investigated in cultured human acute myeloid leukemia (AML) cell lines: HL-60 and OCI-AML3 and primary AML cells. DZNep reduced the level of EZH2 and SUZ12 in these AML cells (**Figure 2**).

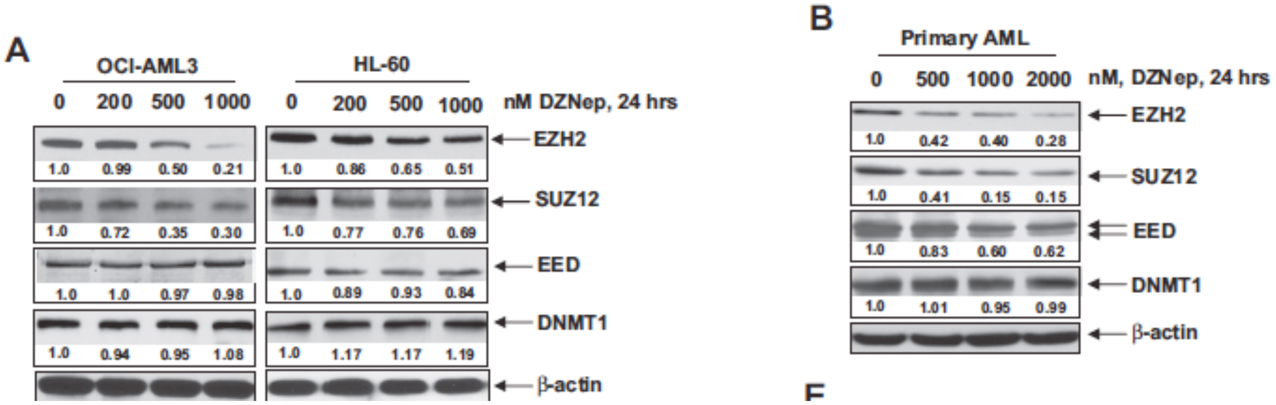


**Figure 2**. Treatment with DZNep depletes expression of polycomb group proteins EZH2, SUZ12, and EED in cultured and primary AML cells. (**A**) OCI-AML3 and HL-60 cells were treated with the indicated concentrations of DZNep for 24 hours. After this, total cell lysates were prepared and immunoblot analysis was performed for EZH2, SUZ12, EED, and DNMT1. The expression levels of β -actin in the lysates served as the loading control. (**B**) Primary AML cells were treated with the indicated concentrations of DZNep for 24 hours. At the end of treatment, cell lysates were prepared and immunoblot analysis was performed for EZH2, SUZ12, EED, and DNMT1. The expression levels of β-actin in the lysates served as the loading control.

DZNep reduced the level of H3K27me3 in HL-60 cells to greater extent that in OCI-AML cells (**Figure 3**).

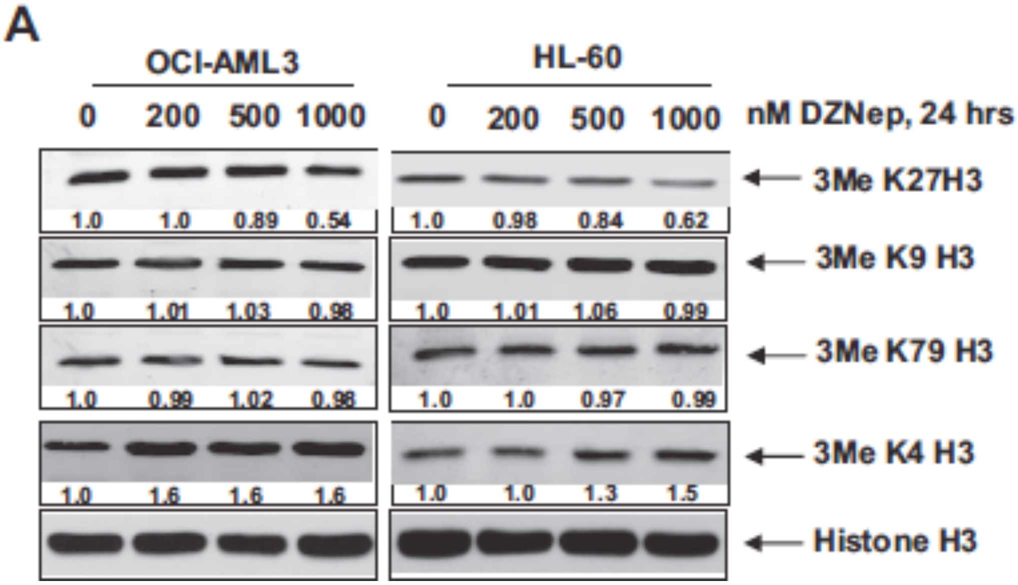


**Figure 3**. DZNep treatment depletes trimethylation of K27 on histone H3 in AML cell lines. (**A)** OCI-AML3 and HL-60 cells were treated with the indicated concentrations of DZNep for 24 hours. After this, nuclear extracts were prepared and immunoblot analysis was performed for 3MeK27 histone H3, 3MeK9 histone H3, 3MeK79 histone H3, and 3MeK4 histone H3. The expression levels of histone H3 in the extracts served as the loading control.

## 12.2 BREAST CANCER (PROTEIN EXPRESSION)

Tan J et al. Pharmacologic disruption of Polycomb-repressive complex 2-mediated gene repression selectively induces apoptosis in cancer cells. Genes Dev. 2007;21:1050-1063.

Treatment of MCF-7 or HCT116 cells with 5 μM DZNep for 48 and 72 h resulted in the dramatic decrease in protein levels of the three PRC2 components: SUZ12, EZH2, and EED. The trimethylation of histone 3 at Lys 27 (H3-K27me3) was strongly reduced in DZNep-treated cells. In contrast, histone H3 trimethylation at Lys 9 (H3-K9me3 was not affected by DZNep treatment. In addition, DZNep treatment did not affect histone H3 acetylation.

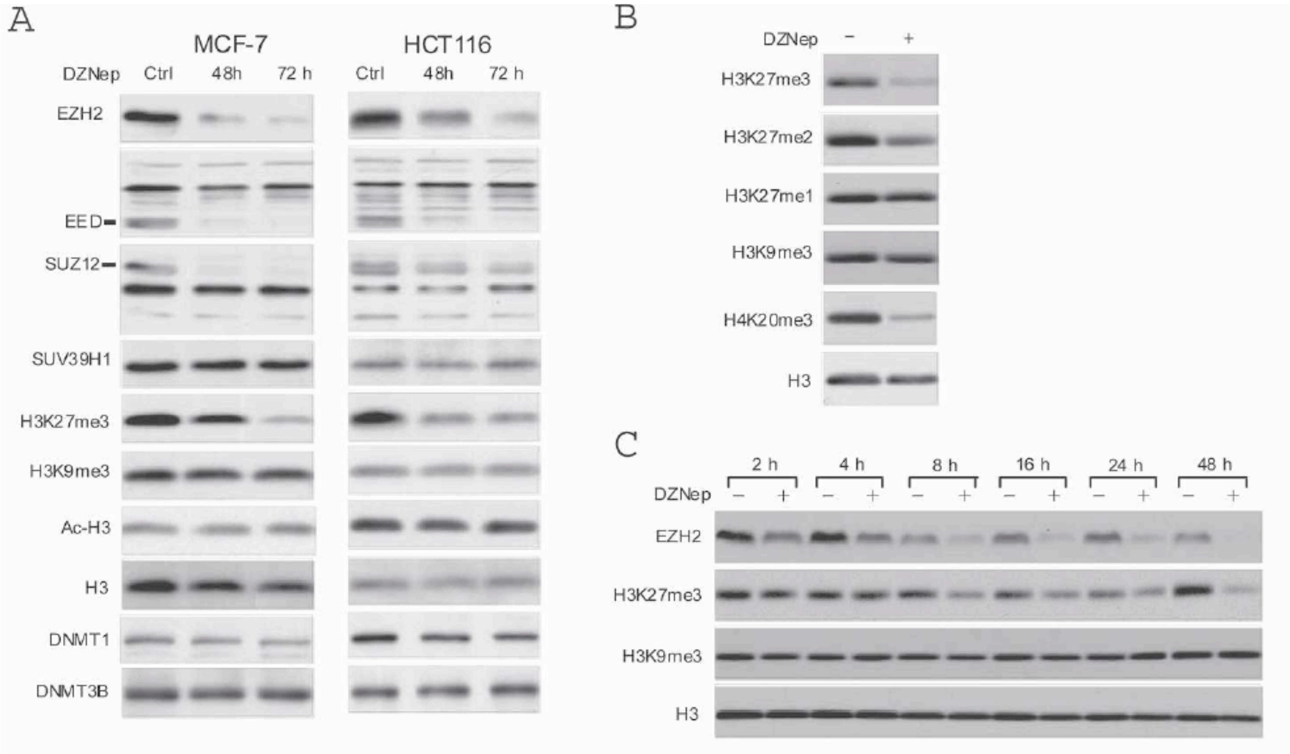


**Figure 2.** Effects of DZNep on PRC2 proteins and histone methylations. (**A**) MCF-7 breast carcinoma cells and HCT116 colon cancer cells were treated with 5 μM DZNep for 48 and 72 h and cells were harvested for Western blot analysis using antibodies detecting the indicated proteins. (**B**) MCF-7 cells were treated with DZNep for 48 h and the levels of indicated histone methylations were examined by Western blot analysis. (**C**) MCF-7 cells were treated with DZNep for the indicated times and cells were harvested for Western blot analysis of the indicated proteins.

To determine if the DZNep-induced depletion of the PRC2 complex is caused by protein degradation, we treated MCF-7 cells with DZNep in the presence or absence of three different proteosome inhibitors (MG132, LLNL, and MG115). As shown in **Figure 2E**, treatment with each proteosome inhibitor at least in part prevented the down-regulation of EZH2 and SUZ12 protein levels in response to DZNep. These results demonstrated that DZNep depleted the PRC2 proteins through increased protein degradation.

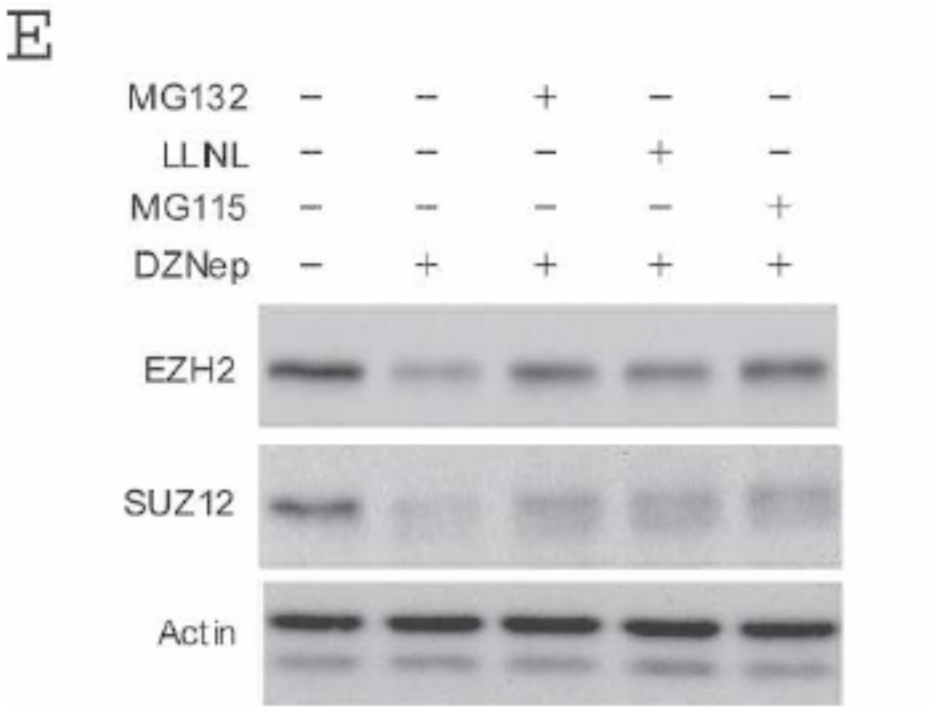


**Figure 2E.** Effects of DZNep on PRC2 proteins and histone methylations. (**E**) MCF-7 cells were treated with DZNep for 18 h, followed by the addition of proteosome inhibitors MG132 (5 μM), LLNL (50μM), or MG115 (20 μM) for 8 h. Cells were harvested for Western blot analysis of EZH2 and SUZ12.

## 12.3 BREAST & BLADDER CANCER (PROTEIN EXPRESSION)

Miranda TB et al. DZNep is a global histone methylation inhibitor that reactivates developmental genes not silenced by DNA methylation. Mol Cancer 2009;8:1579-1588.

DZNep at 10 µM decreased the levels of EZH2 and H3K27me3 inMCF7 breast cancer cells and T24 bladder cancer cells (**Fig 2A**). The authors found that a 72 h exposure to MCF-1 cells at 1 µmol/L DZNep had no effect on EZH2 levels whereas 5 µmol/L DZNep caused a slight reduction in EZH2. DZNep at 1 µM caused a decrease in H3K27me3 and H3K4me3 (**Fig 2B**).

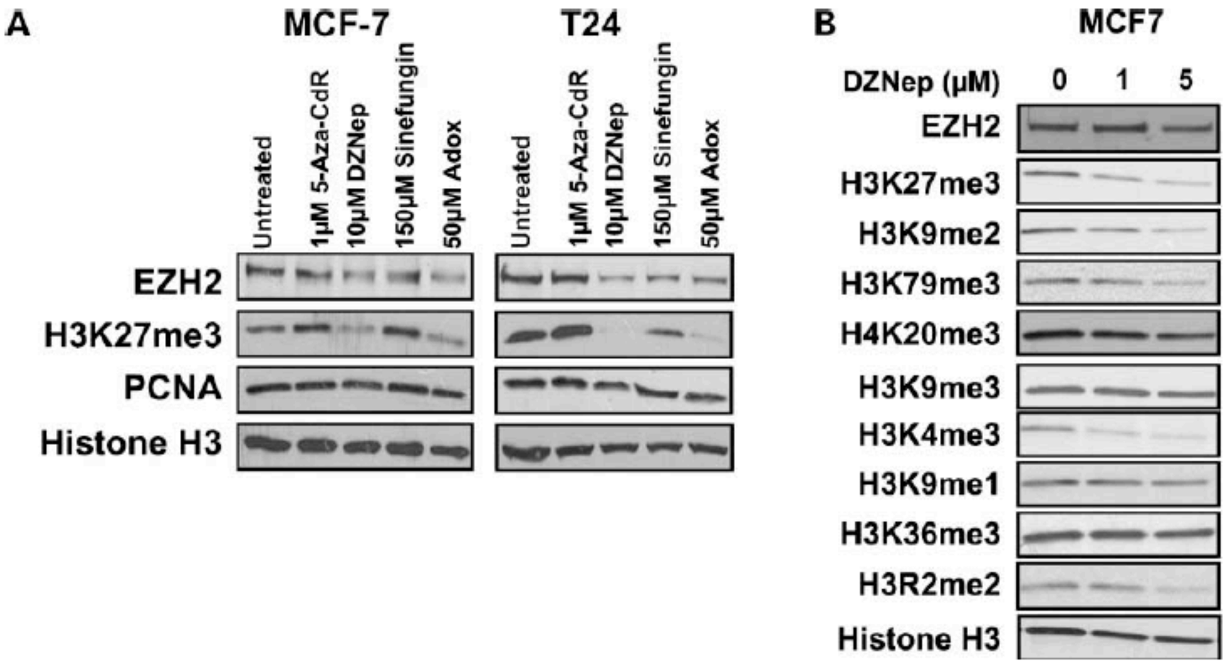


**Figure 2**. The effects of methyltransferase inhibitors on global histone methylation. **A**, MCF7 breast cancer cells and T24 bladder cancer cells were treated with either 1 µmol/L 5-Aza-CdR, 5 or 10 µmol/L DZNep, 150 µmol/L sinefungin, or 50 µmol/L adenosine dialdehyde for 72 h. Cells were harvested, and DNMT1, EZH2, and H3K27me3 levels were measured by Western blot analysis. **B**, MCF-7 cells were treated with either 1 or 5 µmol/L DZNep for 72 h. Cells were harvested and global histone methylation levels were determined by Western blot analysis.

## 12.4 OVARIAN CANCER (PROTEIN EXPRESSION)

Peng D et al., Epigenetic silencing of TH1-type chemokines shapes tumour immunity and immunotherapy. Nature. 2015;527:249-253.

DZNep reduces both the levels of both EZH2 and H3K27me3 in ovarian cancer cells.

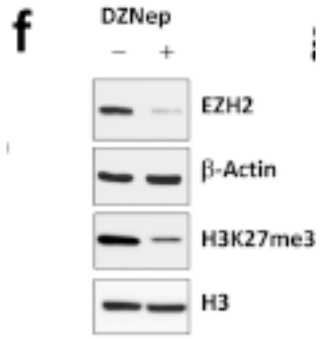


**Extended Data Figure 2f.**
Effects of DZNep on the expression of EZH2 and H3K27me3. Primary ovarian cancer cells were treated with or without DZNep for 24 hours. The levels of EZH2, H3K27me3 were detected by Western blotting and reduced by DZNep treatment.

# 13. ANALYTICAL QUANTIFICATION AND PHARMACOKINETICS

Peer CJ et al. A rapid ultra HPLC-MS/MS method for the quantitation and pharmacokinetic analysis of 3-deazaneplanocin A in mice. J Chromatogr B Analyt Technol Biomed Life Sci. 2013;927:142-6.

## 13.1 Quantification of DZNep by HPLC-mass spectrometric detection.

A rapid method was developed for the detection of DZNep by liquid chromatography-tandem mass spectrometric analysis and extraction of DZNep from 50 µL of plasma. The limit of quantitfication of DZNep in plasma was 5 ng/ml.

## 13.2 Chemical stability

DZNep short-term plasma stability was not a factor, as none of the concentration levels had more than a 15% decrease in calculated concentration after 24 h at room temperature prior to extraction. No significant degradation greater than 15% was observed following two freeze/thaw cycles of plasma samples containing DZNep at plasma concentrations of 9, 900, or 1800 ng/mL. Post-preparative stability demonstrated less than 2% difference between extracted samples analyzed immediately or after 24 h at 4 ◦C. Extraction efficiency was 91.81% and 89.17% for the low (1 ng/mL, n = 3) and high (1000 ng/mL, n = 3) concentrations, respectively.

## 13.3 Linearity of detection; accuracy and precision

The calibration curve showed very good linearity from 5 to 2,100 ng/mL. The deviation for all concentrations from the nominal concentrations was between -4.40% and +4.07%. The precision measured by % coefficient of variation (%CV) ranged from 5.24 to 10.63% (**Table 1**).

**Table 1**
Calibration linearity.

| Nominal (ng/ml) | GM (ng/ml) | S.D. (ng/ml) | DEV (%) | CV (%) | *n* |
|---|---|---|---|---|---|
| 5 | 4.95 | 0.37 | −0.92 | 7.37 | 8 |
| 10 | 10.11 | 0.71 | 1.07 | 7.04 | 8 |
| 50 | 51.58 | 2.70 | 3.16 | 5.24 | 8 |
| 100 | 100.45 | 9.75 | 0.45 | 9.70 | 8 |
| 500 | 520.33 | 42.52 | 4.07 | 8.17 | 8 |
| 1000 | 955.97 | 84.89 | −4.40 | 8.88 | 8 |
| 2000 | 1930.81 | 205.27 | −3.46 | 10.63 | 8 |

*Abbreviations*: GM, grand mean; S.D., standard deviation; DEV (%), relative deviation from nominal value; CV (%), coefficient of variation; *n*, number of replicate observations within each validation run, i.e. two samples at each concentration were run on four separate occasions, for a total (*n*) of eight samples at each concentration.

The deviation from nominal concentration (accuracy) ranged from −4.42% to 8.13% and within-run and between-run precision was all less than 15% (**Table 2**).

## 13.4 Typical chromatogram of HPCL-MS/MS analysis

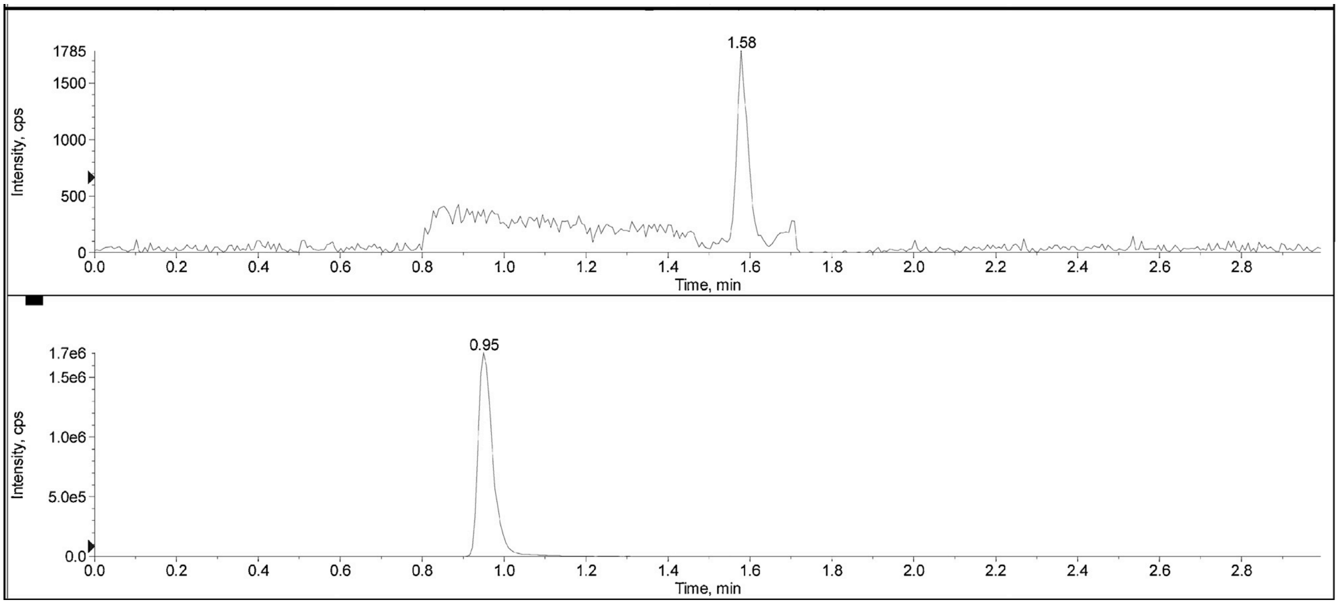


**Figure 2** depicts typical chromatograms resulting from the uHPLC–MS/MS analysis of extracts of 50 µL mouse plasma: The DZNep and tubercidin peaks were chromatographically separated under the optimized conditions, with retention times for tubercidin (bottom) of 0.95, and DZNep (top) of 1.58 min. Tubercidin was used as the internal standard.

**13.5 Example of pharmacokinetic data.**

An example of the data obtained on the pharmacokinetics of DZNep in mice using the HPLC-MS/MS method is shown in **Fig. 3**

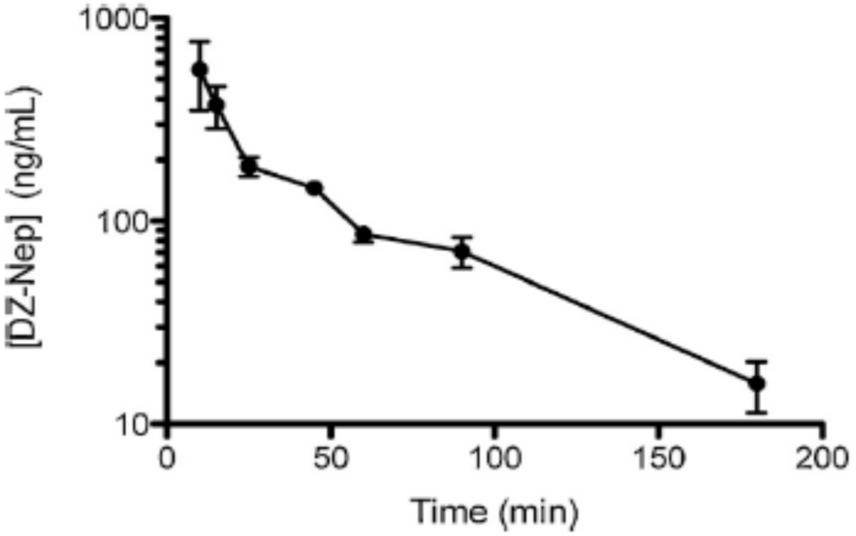


**Fig. 3.** Preclinical concentration–time curves. DZNep was administered intravenously to mice at a dose of 2.5 mg/kg. A two-compartmental model was used to obtain pharmacokinetic parameters.

# 14. PHARMACOKINETICS AND METABOLISM: RATS

Sun F et al., Preclinical pharmacokinetic studies of 3-deazaneplanocin A, a potent epigenetic anticancer agent, and its human pharmacokinetic prediction using GastroPlus™. Euro J Pharmaceutical Sciences 2015;77:290–302.

## 14.1 Abstract

DZNep is a potential epigenetic drug, and exerts potent anti-proliferative and pro-apoptotic effects on broad-spectrum carcinomas via disruption of the EZH2 pathway. Antitumor studies on DZNep have been stuck in the preclinical phase because of the lack of information about its integral pharmacokinetic (PK) properties. To circumvent this problem, we extensively investigated the disposition characteristics of the DZNep in rats. By incorporating the disposition data across species into a whole-body physiologically based pharmacokinetic (PBPK) models using the GastroPlus TM software, we simulated human PK properties of DZNep and determined whether DZNep could be developed for human cancer therapy. Firstly, DZNep was found to cause nephrotoxicity in a dose-dependent manner in rats and its safe dose was determined to be 10 mg/kg. DZNep showed a short plasma elimination half-life (1.1 h) in rats, a low protein binding in plasma (18.5%), a low partitioning to erythrocyte (0.78), and a low intrinsic hepatic clearance in rats and humans. There was extensive tissue distribution and predominant renal excretion (80.3%). The simulated rat PBPK model of DZNep was well-verified with satisfactory coefficients of determination for all the tested tissues (R 2> 0.781). The simulated human PBPK model successfully identified that intravenous administration of DZNep at appropriate dosing regimen could be further developed for human non-small cell lung carcinoma treatments. The present findings provide valuable information regarding experimental PK characteristics of DZNep in rats and humans, which is helpful to guide future studies of DZNep in both preclinical and clinical phases.

## 14.2 Plasma concentration and Pharmacokinetics parameters after i.v. injection

The rats were randomly divided into three groups (each group consisting of six Male Wistar rats). Based on the NOAEL established to be 10 mg/kg, three groups of rats were given intravenous bolus injection of DZNep solution (2 mg/mL in 0.9% NaCl saline) via the tail vein at the doses of 1,5, 10 mg/kg BW, respectively. For all the rats, at sampling times of 0, 0.016, 0.083, 0.17, 0.25, 0.33, 0.5, 0.75, 1, 2, 3, 6, and 9. The concentration–time plots of DZNep in plasma after intravenous bolus injection at doses of 1, 5, and 10 mg/kg were shown in **Fig 2.** Mean DZNep plasma levels fell below the lower limit of quantification (LLOQ) (Sun et al., 2011) 3 h after a single dosing of 1 mg/kg of DZNep and 6 h after that of 5 and 10 mg/kg of DZNep.

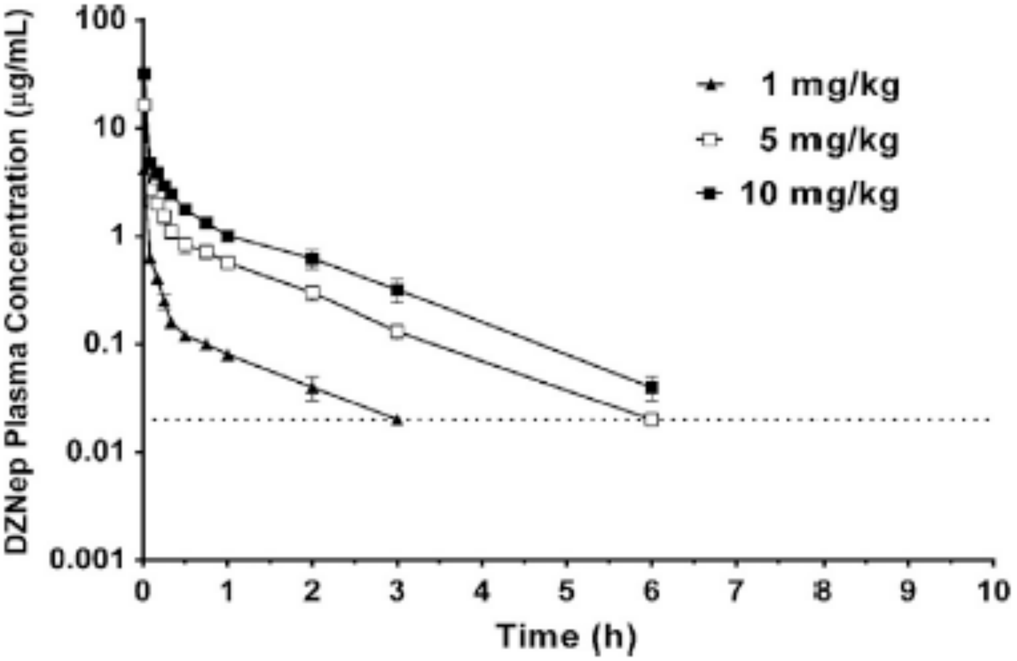


**Figure 2**. Plasma concentration of DZNep in rats after intravenous administration of DZNep solution (0.9% NaCl saline) at dose of 1, 5, 10 mg/kg BW. The mean plasma concentration of DZNep (µg/mL) is expressed as the mean ± SD (n = 6). The lower dotted line indicates the LLOQ (20 ng/mL) of LC–MS/MS method for quantifying DZNep in the rat plasma.

From 0.078 to 0.13 h the terminal half-lives ($t_{1/2,c}$) of DZNep ranged from 1.0 to 1.2 h (**Table 4**). No differences were noted in AUC0–1/dose ($p = 0.45$) and Cmax/dose ($p = 0.23$), across the investigated dosage range (1 mg/kg to 10 mg/kg BW), indicating that the plasma PK of DZNep is a dose-independent after single intravenous bolus dosing. The CL, ranging within 1.8–1.9 L/h/kg, remained unaltered over the dose range studied ($p = 0.40$). In terms of the inter-compartmental distribution between the central and two peripheral compartments, in the dose range, CLD2 ranging within2.1–2.9 L/h/kg was higher than CLD3 ranging within 0.88–1.55 L/h/kg. The Vss, independent of the dose ($p = 0.09$), ranged within 1.5–1.7 L/kg. Since the pharmacokinetics of DZNep appeared to be a dose-independent and linear in rat plasma after the single dosing, the dose of 5 mg/kg DZNep was chosen as the standard dose for the following the disposition study of DZNep in rats.

**Table 4**
Pharmacokinetic parameters for DZNep after an intravenous bolus to rats ($n = 6$).

| Parameter | Units | 1 mg/kg (a) | 5 mg/kg (b) |
|---|---|---|---|
| $t_{1/2,\alpha}$ | h | 0.0063 ± 0.0005 | 0.0062 ± 0.0005 |
| $t_{1/2,\beta}$ | h | 0.078 ± 0.013 | 0.099 ± 0.026 |
| $t_{1/2,\gamma}$ | h | 1.2 ± 0.1 | 1.0 ± 0.1 |
| $AUC_{0-\infty}$ | h * µg/mL | 0.52 ± 0.03 | 2.73 ± 0.25 |
| AUC/dose | h * g/mL | 0.52 ± 0.03 | 0.55 ± 0.05 |
| $C_{max}$ | µg/mL | 19.5 ± 3.0 | 78.5 ± 11.7 |
| $C_{max}$/dose | g/mL | 19.5 ± 3.0 | 15.7 ± 2.3 |
| CL | L/h/kg | 2.14 ± 0.12 | 1.96 ± 0.18 |
| $CL_{D2}$ | L/h/kg | 2.06 ± 0.34 | 2.80 ± 0.29 |
| $CL_{D3}$ | L/h/kg | 0.88 ± 0.23 | 1.55 ± 0.34 |
| $V_{ss}$ | mL/kg | 1742 ± 184 | 2095 ± 267 |

Pharmacokinetic parameters (macroconstants: a, b, c) are obtained from three-compartment model with first order elimination. Data are expressed as mean ± SD. $CL_{D2}$ and $CL_{D3}$ are inter-compartmental distribution between shallow peripheral compartment and central compartment, and between deep peripheral compartment and central compartment, respectively.

Unbound fractions of DZNep in human and rat plasma and blood to-plasma ratio (B/P), erythrocyte partitioning (q) of DZNep in rats. The extent of binding of DZNep to plasma protein was evaluate by an in vitro ultrafiltration method. The extent of non specific binding of DZNep to the Microcon cartridge was negligible (<2%).The unbound fractions of DZNep to rat and human plasma protein(fup) were 81.5 ± 0.8% and 74.6 ± 2.1%, over the concentration range of 50–5000 ng/mL, respectively. The plasma and blood concentrations of DZNep after a single intravenous dose of 5 mg/kg of DZNep in rats are summarized in **Table 2.** The ratio of blood-to-plasma was 0.85, and this was independent of plasma concentrations ranging of 44–3068 ng/mL achieved in vivo(p = 0.27). Furthermore, the affinity of blood cells for DZNep in rat blood (q) estimated was 0.78 (**Table 2**), assuming that the mean rat blood hematocrit was 0.41 (**Table 3**). The ratio of DZNep AUC in blood cells to that in plasma was calculated as 0.64 (qfu) (**Table 5**).

Table 5: unbounding fraction of DZNep in rats

| Time (h) | $C_p$ (ng/mL) | B/P or $C_b/C_p$ | $\rho$ |
|---|---|---|---|
| 0.08 | 3068 ± 249 | 0.89 ± 0.06 | 0.90 ± 0.19 |
| 0.25 | 1558 ± 162 | 0.90 ± 0.06 | 0.94 ± 0.18 |
| 0.5 | 886 ± 111 | 0.84 ± 0.05 | 0.74 ± 0.16 |
| 1 | 298 ± 47 | 0.85 ± 0.08 | 0.77 ± 0.24 |
| 3 | 159 ± 12 | 0.80 ± 0.09 | 0.64 ± 0.28 |
| 6 | 44 ± 9 | 0.83 ± 0.10 | 0.72 ± 0.29 |
| Mean ± SD | | 0.85 ± 0.04 | 0.78 ± 0.12 |
| $p$ | | 0.27 | 0.3 |

### 14.3 Distribution in organs

The time course of DZNep tissue distribution and tissue-to-plasma concentration ratios following an intravenous bolus dose of 5 mg/kg DZNep to rats are shown in **Fig. 3A** and 3**B**. DZNep was detectable almost in all tissues at 5 min, except for brain, with considerable accumulation, particularly in kidney (17.2 ± 2.4 μg/g). Most tissue-to-plasma concentration ratios were greater than 1, suggesting that DZNep was rapidly and widely distributed throughout various tissues in rats but prevented from entering into the brain by the blood–brain barrier. The accumulation of DZNep was greater in well-perfused organs (e.g., kidney) than that in poorly-perfused organs (e.g., adipose) (**Fig. 3A**). Despite initial high accumulation, DZNep rapidly declined over time in the well-perfused organs, e.g. from 17.2 μg/g at 5 min to0.88 μg/g at 6 h with the half-life of 1.5 h in kidney (**Fig. 3A**). By contrast, DZNep decreased slowly in the poorly-perfused organs, e.g. from 0.59 μg/g at 5 min to 0.17 μg/g at 6 h with the half-life of 3.3 h in adipose tissue. Correspondingly, the tissue-to-plasma concentration ratio of DZNep for most of the observed tissues peaked at about 0.5–1 h and then remained relatively stable or slowly declined until 6 h (**Fig. 3B**) suggesting that a fast pseudo-equilibration occurred. The tissue-to-plasma partition coefficients (p) of DZNep were determined in rat organs/tissue during the steady state.

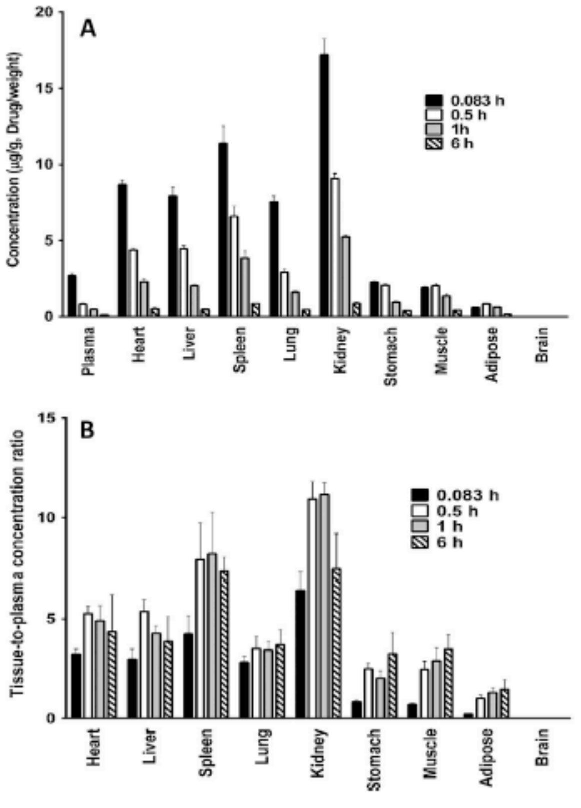


**Fig. 3.** (A) Concentration of DZNep in tissues and plasma (μg/g, Drug/body weight) and (B) tissue-to-plasma concentration ratios at 0.083, 0.5, 1 and 6 h after a single intravenous administration of 5 mg/kg DZNep into rats ($n = 5$). The levels of DZNep are expressed as the mean ± SD.

**Table 6**
Input data used in the GastroPlusTM PBPK model.

| Parameters | Unit | Species | | |
|---|---|---|---|---|
| | | Rat | Men | Women |
| Dose (mg) | mg | 1.5[b] | 71[d] | 63[d] |
| Body weight (kg)[a] | kg | 0.3 | 87.8 | 78.3 |
| Dosing regimen[b] | | iv bolus | iv bolus | iv bolus |
| MW (g/mol)[a] | g/mol | 262.3 | 262.3 | 262.3 |
| Log P at pH 7.4[c] | | −1.1 | −1.1 | −1.1 |
| p$K_a$[c] | | 6.22 | 6.22 | 6.22 |
| Intrinsic Solubility in water, pH = 9.84[a] | mg/mL | 16.97 | 16.97 | 16.97 |
| Blood-to-plasma ratio | | 0.85[b] | 0.85[d] | 0.85[d] |
| Fraction unbound in plasma ($f_{up}$)[b] | | 81.5% | 74.6% | 74.6% |
| Haematocrit | | 0.41[b] | 0.45[a] | 0.45[a] |
| Hepatic $CL_{int,app}$ (L/h)[b] | L/h | 0.123 | 19.49 | 19.49 |
| Hepatic CL (L/h)[d] | L/h | 0.088 | 12.2 | 12.2 |
| Renal CL (L/h)[b] | L/h | 0.41[b] | 26.6[d] | 24.4[d] |
| GFR (L/h)[a] | L/h | 0.078 | 7.2 | 7.2 |
| $V_{ss}$ by PBPK[d] | L | 0.73 | 156.7 | 127.2 |
| $CL_{sys}$ by PBPK[d] | L/h | 0.50 | 38.4 | 38.3 |
| $t_{1/2}$ by PBPK[d] | h | 1.0 | 2.8 | 2.3 |
| $K_{ps}$ | | | | |
| Adipose | | 1.3[b] | 1.2[d] | 1.2[d] |
| Brain | | 0[b] | 0[d] | 0[d] |
| Heart | | 4.8[b] | 4.4[d] | 4.4[d] |
| Kidney | | 11.1[b] | 10.1[d] | 10.1[d] |
| Liver | | 4.8[b] | 4.4[d] | 4.4[d] |
| Lung | | 3.6[b] | 3.2[d] | 3.2[d] |
| Muscle | | 2.9[b] | 2.7[d] | 2.7[d] |
| Red Marrow[a] | | 1.5 | 1.4[d] | 1.4[d] |
| Reproduction[a] | | 1.2 | 1.1[d] | 1.1[d] |
| Skin | | 1 | 0.92[d] | 0.92[d] |
| Spleen | | 7.8[b] | 7.1[d] | 7.1[d] |
| Yellow Marrow | | 1.3[b] | 1.2[d] | 1.2[d] |

[a] Parameter values were predicted from molecular structure using ADMET predictor, version 6.5 or GastroPlus inner database.
[b] Parameter values were from our experiments.
[c] From reference: (Sun et al., 2012).
[d] Parameter values were calculated following the input data from a, b and c.

Hepatic metabolism

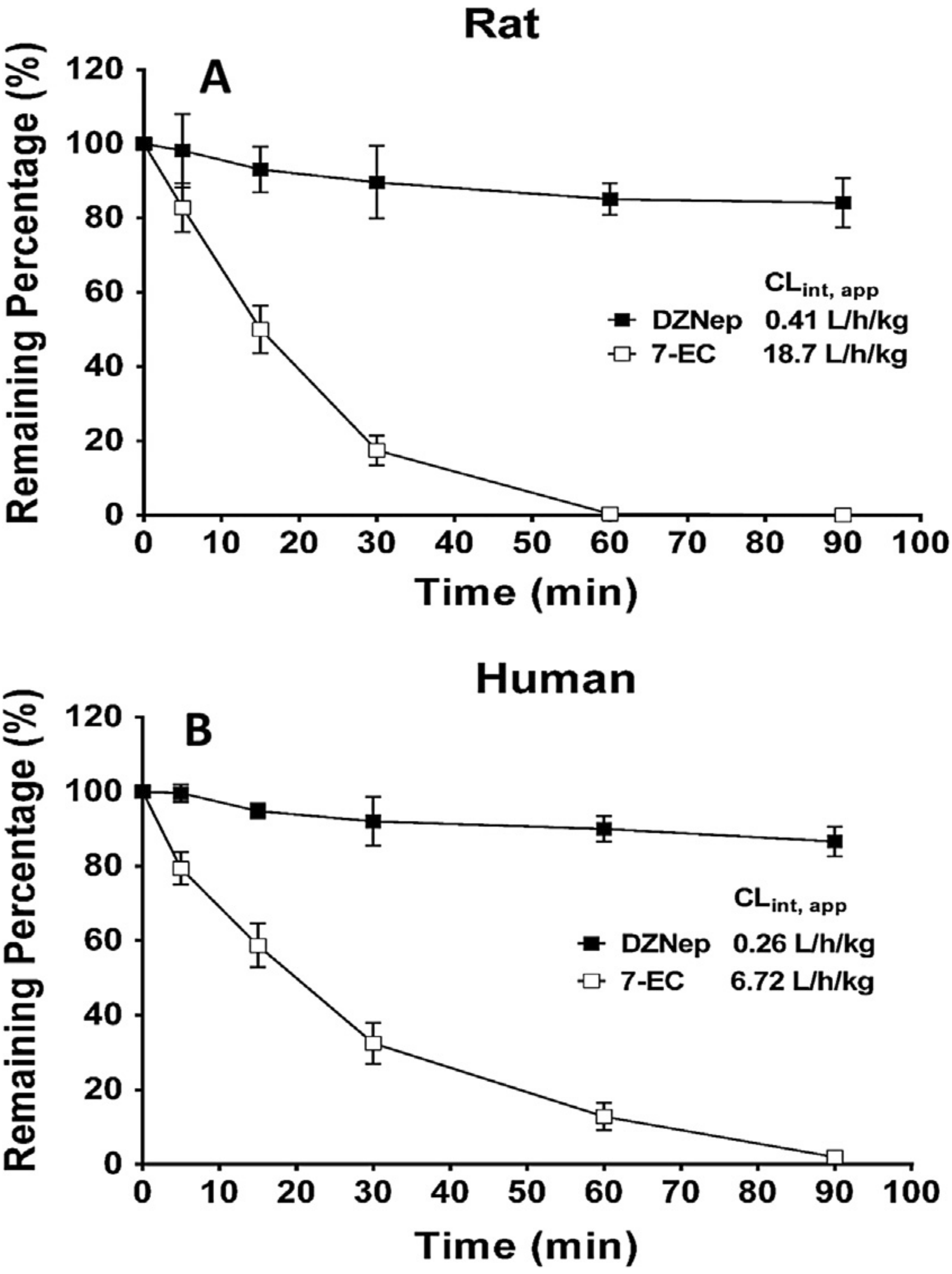


**Figure 6.** Metabolic stability profiles for 1 μg/mL of DZNep and 1 μg/mL of 7-EC in liver microsomes (0.5 mg microsome protein/mL) of rats (**A**), and humans (**B**)

### 14.4 Excretion in urine and feces

In rats given a single intravenous dose of 5 mg/kg DZNep, 69.5%and 79.5% of the drug were excreted unchanged in the urine by 6 and 12 h, respectively (**Fig. 12**). By 72 h, 80.3% of the DZNep administered dose had been recovered unchanged in the urine. In contrast, only 6.5% of DZNep dosed were excreted in the feces by 72 h. Feces was not likely to be contaminated by urine as the DZNep excreted in the feces was detectable after 12 h post dose when DZNep can not be found in urine. Furthermore, by comparing the cumulative amount of DZNep excreted in urine with plasma AUC0–1 for DZNep (**Table 1**), the renal clearance (CLr) was estimated to be 1.35 L/h/kg. In total, 86.8% of unchanged DZNep was recovered from urine and feces over 72 h post pose.

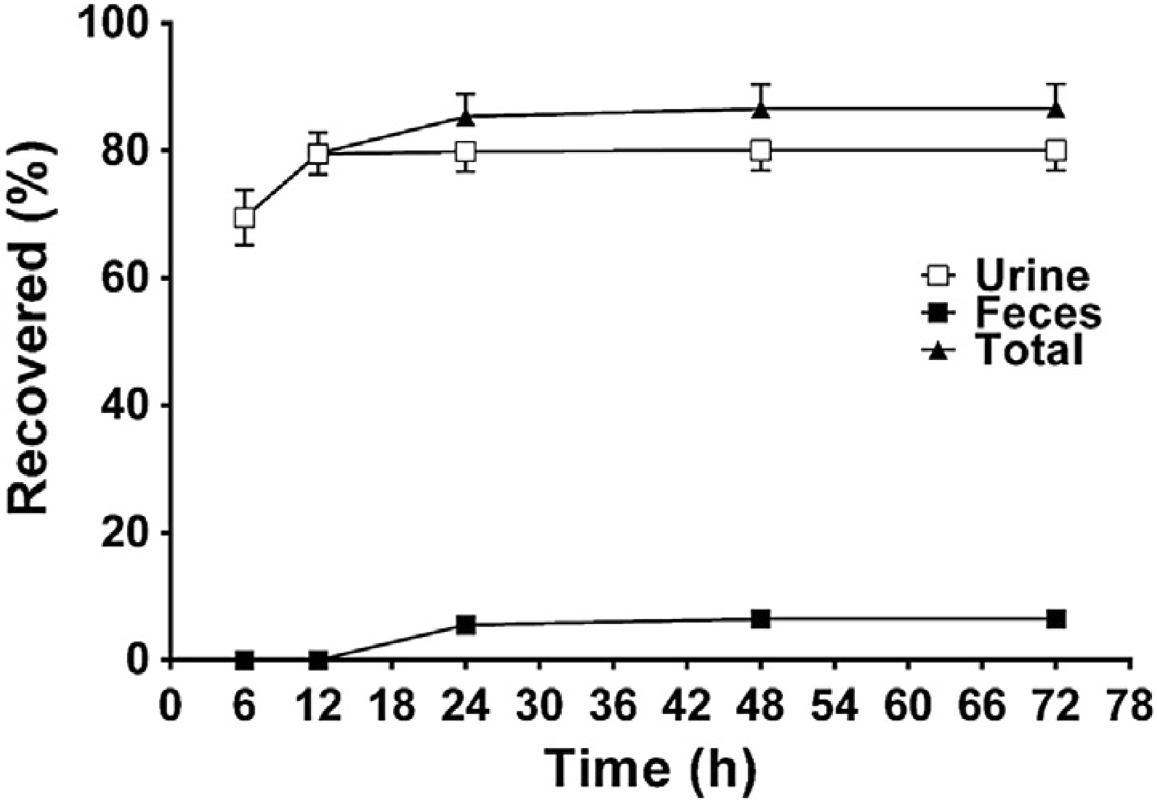


**Figure 4.** Cumulative recovery of DZNep in urine and feces following a single intravenous administration of 5 mg/kg DZNep into rats (n = 6). The levels of DZNep recovered are expressed as the mean ± SD (%).

## 15. GENERAL TOXICITY: RATS

Sun et al. Preclinical pharmacokinetic studies of 3-deazaneplanocin A, a potent epigenetic anticancer agent, and its human pharmacokinetic prediction using GastroPlus™ .
Euro J Pharmaceutical Sciences 2015;77:290–302.

### 15.1 Body Weight

The effect of DZNep on body weight change (**Fig. 1**) and major organ weights on the fourteenth day postdose is summarized in **Table** 1. The ratio of kidney weight to the body weight (expressed as gram per 100 g body weight) was significantly decreased ($p < 0.01$), but other organs were not significantly changed in rats treated with 20 mg/kg of DZNep as compared to the control rats. Additionally, no obvious changes in all organ weight and organ size were detected in rats with the other two dose levels.

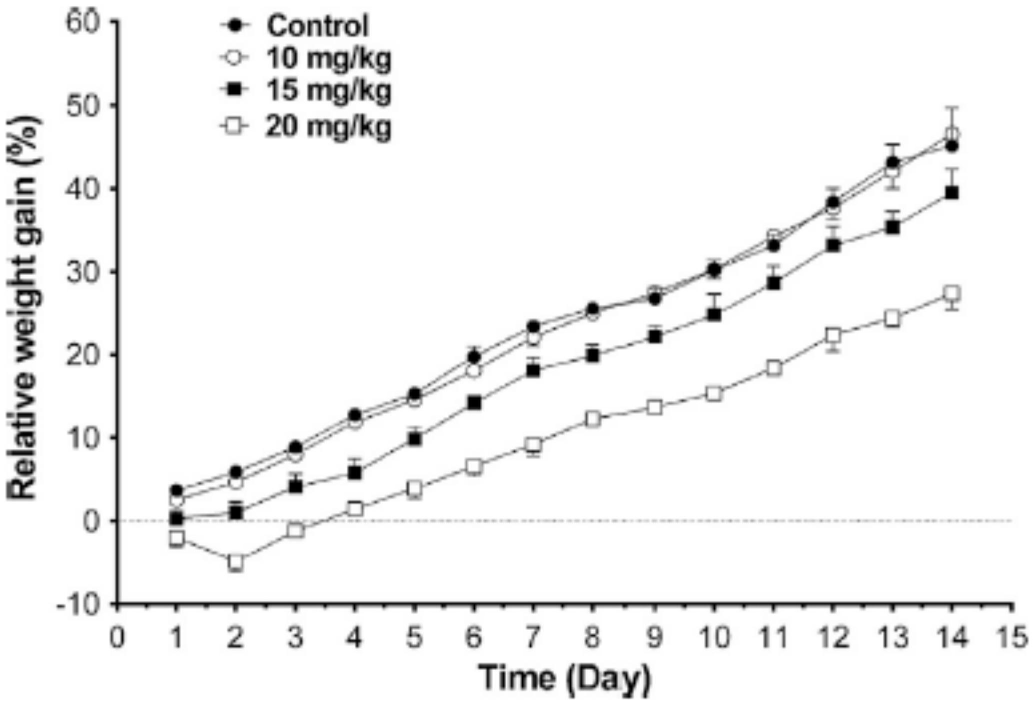


**Fig. 1.** Mean relative body weight gain-time profiles of rats for 14 days following intravenous injection of DZNep solution at the doses of 0 (control), 10, 15, 20 mg/kg BW. Each value represents mean ± SD ($n = 5$). The line (0%) indicates no body weight gain as compared to the initial body weight. The double and single asterisks represent significant difference from control at $p < 0.01$ and $p < 0.05$, respectively.

**Table 1**
Effect of DZNep in various dose levels on relative organ weights in the rats ($n = 5$).

| Dose (g/kg BW) | Relative organ weight (g/100 g BW) | | | | |
|---|---|---|---|---|---|
| | Heart | Liver | Spleen | Lung | Kidney |
| Control | 0.30 ± 0.02 | 4.31 ± 0.21 | 0.24 ± 0.02 | 0.35 ± 0.03 | 0.79 ± 0.06 |
| 10 | 0.28 ± 0.02 | 4.34 ± 0.25 | 0.23 ± 0.03 | 0.34 ± 0.05 | 0.77 ± 0.03 |
| 15 | 0.28 ± 0.01 | 4.42 ± 0.19 | 0.23 ± 0.02 | 0.39 ± 0.04 | 0.73 ± 0.04 |
| 20 | 0.29 ± 0.03 | 4.17 ± 0.15 | 0.23 ± 0.03 | 0.36 ± 0.03 | 0.62 ± 0.05** |

** Significant difference from control at $p < 0.01$.

### 15.2 Blood Chemistry

Blood chemistry analysis: Significant increases in serum BUN and Cr in rats treated with 20 mg/kg DZNep were observed, compared to the control group, on the second day and fourteenth day post-treatment (**Table 2**). In contrast, no significant changes occurred in serum BUN and Cr at the dose of 10 mg/kg DZNep. In addition, other clinical chemistry parameters were within the reference ranges and remained unchanged in rats at various DZNep dose levels.

**Table 2**
Effect of DZNep in various dose levels on clinical chemistry parameters in rats ($n = 5$).

| Item | Unit | Ref. range | Second day | | | |
|---|---|---|---|---|---|---|
| | | | Control | 10 mg/kg | 15 mg/kg | 20 mg/kg |
| ALP | U/L | 70–450 | 294.0 ± 26.9 | 314.3 ± 40.4 | 307.8 ± 44.4 | 310.7 ± 52.3 |
| AST | U/L | 20–100 | 80.4 ± 9.0 | 76.8 ± 8.3 | 70.4 ± 8.1 | 72.4 ± 5.9 |
| ALT | U/L | 10–80 | 48.9 ± 6.5 | 52.1 ± 7.2 | 46.9 ± 8.2 | 47.1 ± 6.8 |
| Total protein | g/L | 50–80 | 58.0 ± 4.4 | 62.7 ± 4.4 | 59.0 ± 7.8 | 57.4 ± 6.4 |
| Albumin | g/L | 30–50 | 36.9 ± 1.3 | 37.6 ± 1.2 | 38.4 ± 1.2 | 38.3 ± 0.8 |
| Bilirubin-D | mg/dL | 0–0.2 | 0.026 ± 0.006 | 0.038 ± 0.013 | 0.029 ± 0.016 | 0.041 ± 0.016 |
| Bilirubin-total | mg/dL | 0.2–0.5 | 0.26 ± 0.02 | 0.27 ± 0.02 | 0.27 ± 0.01 | 0.25 ± 0.02 |
| BUN | mg/dL | 10–21 | 18.7 ± 3.7 | 19.0 ± 1.2 | 24.9 ± 2.1* | 32.5 ± 3.4** |
| Creatinine | mg/dL | 0.1–0.8 | 0.17 ± 0.03 | 0.18 ± 0.03 | 0.24 ± 0.05* | 0.30 ± 0.05** |
| Item | Unit | Ref. range | Fourteenth day | | | |
| | | | Control | 10 mg/kg | 15 mg/kg | 20 mg/kg |
| ALP | U/L | 70–450 | 301.3 ± 38.1 | 293.7 ± 40.3 | 278.5 ± 45.6 | 301.9 ± 38.9 |
| AST | U/L | 20–100 | 68.4 ± 6.3 | 73.4 ± 7.4 | 70.9 ± 10.7 | 75.4 ± 11.3 |
| ALT | U/L | 10–80 | 48.3 ± 5.5 | 48.7 ± 6.7 | 46.9 ± 9.4 | 55.4 ± 8.2 |
| Total protein | g/L | 50–80 | 62.2 ± 4.9 | 60.3 ± 4.8 | 61.5 ± 6.4 | 62.5 ± 6.3 |
| Albumin | g/L | 30–50 | 39.4 ± 1.6 | 38.9 ± 1.1 | 39.4 ± 0.8 | 39.9 ± 2.2 |
| Bilirubin-D | mg/dL | 0–0.2 | 0.037 ± 0.011 | 0.033 ± 0.013 | 0.034 ± 0.014 | 0.037 ± 0.010 |
| Bilirubin-total | mg/dL | 0.2–0.5 | 0.27 ± 0.03 | 0.27 ± 0.01 | 0.26 ± 0.03 | 0.26 ± 0.02 |
| BUN | mg/dL | 10–21 | 17.4 ± 1.4 | 18.3 ± 2.6 | 18.0 ± 4.0 | 24.9 ± 3.1** |
| Creatinine | mg/dL | 0.1–0.8 | 0.20 ± 0.03 | 0.20 ± 0.03 | 0.19 ± 0.05 | 0.27 ± 0.04* |

Ref. range: reference range supplied by the NUS Vivarium at the Centre for Life Science.
** Significant difference from control at $p < 0.01$.
* Significant difference from control at $p < 0.05$.

Based on the various endpoints obtained, including body weight change (**Fig. 1**), organ weight change (**Table 1**), clinical signs, clinical chemistry parameters (**Table 2**) and hematological parameters (**Table 3**), intravenous administration of DZNep to rats at a dose of as high as 10 mg/kg body weight did not cause any obvious signs of toxicity or mortality. Therefore, the NOAEL (no observable adverse effect level) of DZNep determined was 10 mg/kg body weight, the highest single safe dose tested in rats with intravenous bolus injection.

## 15.3 Hematology

DZNep treatments in every group revealed insignificant changes in rat hematological parameters at day 2 and day 14 (**Table 3**). Rats were given intravenous bolus injection of DZNep solution (2 mg/mL in 0.9% NaCl) via the tail vein at the doses of 1, 5, 10 mg/kg body weight respectively.

**Table 3**
Effect of DZNep in various dose levels on Hematological parameters in rats (*n* = 5).

| Item | Unit | Ref. range | Second day | | | |
|---|---|---|---|---|---|---|
| | | | Control | 10 mg/kg | 15 mg/kg | 20 mg/kg |
| WBC | K/μL | 3.1–16.1 | 5.4 ± 0.7 | 4.8 ± 0.7 | 5.3 ± 0.8 | 4.7 ± 1.0 |
| NEU | K/μL | 0–4.2 | 0.40 ± 0.09 | 0.44 ± 0.08 | 0.55 ± 0.10 | 0.62 ± 0.06 |
| LYM | K/μL | 2.2–12.0 | 5.0 ± 1.4 | 4.3 ± 1.7 | 5.1 ± 1.4 | 4.1 ± 1.6 |
| Mono | K/μL | 0–0.7 | 0.33 ± 0.10 | 0.43 ± 0.11 | 0.39 ± 0.09 | 0.35 ± 0.08 |
| EOS | K/μL | 0–0.5 | 0.19 ± 0.05 | 0.21 ± 0.06 | 0.15 ± 0.06 | 0.22 ± 0.09 |
| BASO | K/μL | 0–0.1 | 0.09 ± 0.03 | 0.09 ± 0.04 | 0.10 ± 0.03 | 0.08 ± 0.02 |
| RBC | M/μL | 6.4–8.9 | 6.9 ± 0.3 | 6.9 ± 0.3 | 6.7 ± 0.4 | 6.9 ± 0.6 |
| HGB | g/dL | 12.9–16.4 | 14.1 ± 0.7 | 13.8 ± 0.4 | 13.8 ± 0.6 | 14.5 ± 0.3 |
| HCT | % | 31.2–50.8 | 40.0 ± 4.3 | 39.0 ± 2.8 | 40.4 ± 3.4 | 44.1 ± 3.9 |
| MCV | fL | 52.4–69.1 | 60.2 ± 3.4 | 57.7 ± 3.9 | 58.8 ± 1.8 | 60.2 ± 2.5 |
| MCH | pg | 16.5–22.1 | 21.3 ± 0.4 | 20.7 ± 0.8 | 20.9 ± 0.7 | 21.1 ± 1.2 |
| MCHC | g/dL | 29.6–34.1 | 33.0 ± 0.5 | 33.2 ± 0.6 | 33.1 ± 0.7 | 32.9 ± 0.8 |
| RDW | % | 0–99.9 | 14.2 ± 0.7 | 14.4 ± 0.5 | 14.7 ± 0.6 | 14.1 ± 0.6 |
| Platelet count | K/μL | 595–1424 | 902 ± 94 | 922 ± 49 | 945 ± 87 | 970 ± 90 |
| MPV | fL | 0–99.9 | 5.7 ± 0.4 | 5. 9 ± 0.6 | 5.4 ± 0.6 | 5.5 ± 0.5 |
| Item | Unit | Ref. Range | Fourteenth day | | | |
| | | | Control | 10 mg/kg | 15 mg/kg | 20 mg/kg |
| WBC | K/μL | 3.1–16.1 | 5.9 ± 1.0 | 5.5 ± 1.5 | 5.7 ± 1.1 | 5.7 ± 1.0 |
| NEU | K/μL | 0–4.2 | 0.41 ± 0.04 | 0.43 ± 0.07 | 0.36 ± 0.02 | 0.59 ± 0.11 |
| LYM | K/μL | 2.2–12.0 | 5.3 ± 1.8 | 5.1 ± 1.2 | 6.0 ± 1.5 | 6.2 ± 2.0 |
| Mono | K/μL | 0–0.7 | 0.31 ± 0.08 | 0.34 ± 0.07 | 0.37 ± 0.08 | 0.37 ± 0.07 |
| EOS | K/μL | 0–0.5 | 0.20 ± 0.06 | 0.18 ± 0.04 | 0.19 ± 0.06 | 0.28 ± 0.07 |
| BASO | K/μL | 0–0.1 | 0.09 ± 0.03 | 0.09 ± 0.01 | 0.10 ± 0.02 | 0.10 ± 0.03 |
| RBC | M/μL | 6.4–8.9 | 7.2 ± 0.4 | 7.5 ± 0.5 | 7.3 ± 0.2 | 7.2 ± 0.4 |
| HGB | g/dL | 12.9–16.4 | 14.5 ± 0.6 | 14.2 ± 0.4 | 14.6 ± 0.7 | 14.0 ± 0.7 |
| HCT | % | 31.2–50.8 | 42.5 ± 4.0 | 40.1 ± 6.4 | 41.0 ± 1.6 | 39.1 ± 4.0 |
| MCV | fL | 52.4–69.1 | 58.9 ± 2.5 | 59.5 ± 4.1 | 58.1 ± 2.0 | 60.5 ± 1.8 |
| MCH | pg | 16.5–22.1 | 20.6 ± 0.5 | 20.9 ± 0.6 | 20.9 ± 0.4 | 20.3 ± 1.6 |
| MCHC | g/dL | 29.6–34.1 | 33.0 ± 0.2 | 33.5 ± 1.0 | 33.1 ± 0.8 | 32.6 ± 1.6 |
| RDW | % | 0–99.9 | 14.4 ± 0.9 | 14.8 ± 0.9 | 15.1 ± 0.8 | 14.8 ± 1.1 |
| Platelet count | K/μL | 595–1424 | 964 ± 63 | 939 ± 45 | 1002 ± 107 | 974 ± 74 |
| MPV | fL | 0–99.9 | 5.6 ± 0.5 | 5.9 ± 0.6 | 5.5 ± 0.4 | 6.0 ± 0.5 |

# 16. GENERAL TOXICITY: MICE

## 16.1 Tissue injury and body weight loss in mice by DZNep treatment

Lhussier E et al., Evaluation of the impact of S-adenosylmethionine-dependent methyltransferase inhibitor, 3-deazaneplanocin A, on tissue injury and cognitive function in mice. Oncotarget, 2018;9(29):20698-20708.

The chronic administration of 3-deazaneplanocin A (DZNep) (2 mg/kg; i.p., three times per week during 8 weeks) was investigated in male NMRI mice. Animals displayed a significant decrease in body weight gain after 3 weeks of DZNep treatment, but this decrease was reversible after treatment arrest (Figure 1A). The investigators observed an increase in spleen (Figure 1B). Histological analysis of spleen sections did not show difference between treated or untreated mice (Figure 1C). Histological analysis of spleen did not show difference between treated or untreated mice (Figure 1C). The investigators observed a decrease in testis weight and volume, (Figure 1B). Testis histological sections showed a hyperplasia of Leydig cells and a disappearance of spermatogonia (Figure 1C). No changes were observed in weight nor in histology in other tissues (heart, lung, brain, kidney, liver) (Figure 1D and 1E).

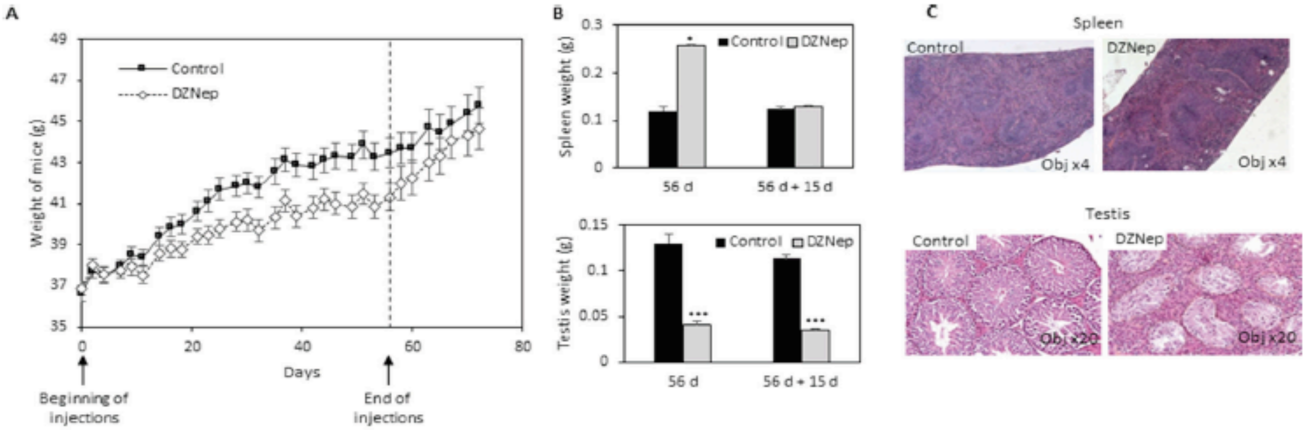


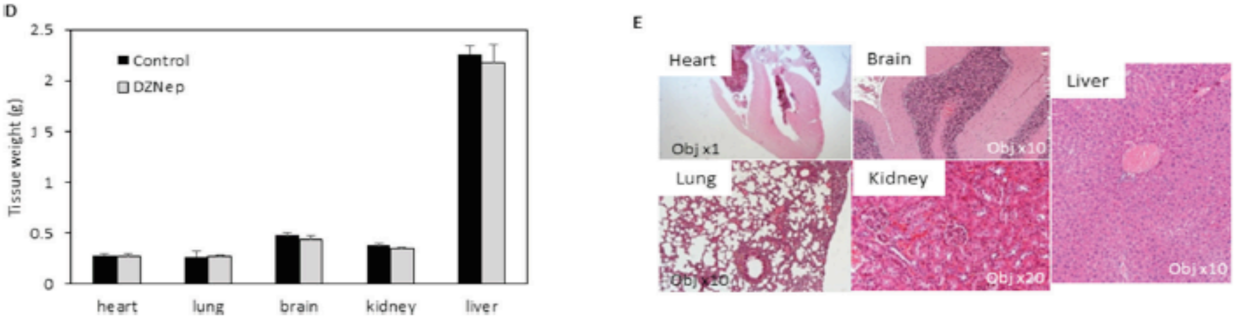


**Figure 1**: Effect of DZNep on body weight, and tissues of immunocompetent mice. NMRI mice were treated for 8 weeks with DZNep (i.p., 2 mg/kg/days, 3 times a week), then kept alive for two supplemental weeks without injection. Weight of mice were measured regularly during experiment (A). At the end of treatment (B, 56 d, and D) and 15 days later (without supplemental injections) (B, 56 d + 15 d), organs were removed and weighted. Data are expressed as means ± SEM (n = 3). Histological sections with HES staining were performed 15 days after the end of DZNep injections (C and E).

## 16.2 Hematological analysis in mice after treatment with DZNep

Hematological analysis did not reveal difference in cell blood count, except a reduction of reticulocyte number (Table 1).

**Table 1: Hematological parameters in NMRI mice**

| | Control | DZNep | *p*-value |
|---|---|---|---|
| WBC (10^9/l) | 2.41 ± 0.32 | 1.91 ± 1.01 | 0.246 |
| RBC (10^12/l) | 7.61 ± 0.14 | 7.24 ± 1.03 | 0.231 |
| HGB (g/dl) | 11.86 ± 0.22 | 11.30 ± 1.65 | 0.258 |
| MCV (fl) | 54.44 ± 0.37 | 54.54 ± 1.94 | 0.875 |
| Platelets (10^9/l) | 540.79 ± 64.99 | 442.24 ± 104.85 | 0.188 |
| PDW (fl) | 6.12 ± 0.09 | 6.25 ± 0.88 | 0.623 |
| Neutrophils (10^9/l) | 0.53 ± 0.04 | 0.46 ± 0.16 | 0.321 |
| Lymphocytes (10^9/l) | 1.64 ± 0.22 | 1.19 ± 0.57 | 0.122 |
| Monocytes (10^9/l) | 0.03 ± 0.01 | 0.03 ± 0.03 | 0.943 |
| Eosinophils (10^9/l) | 0.01 ± 0.01 | 0.00 ± 0.01 | 0.504 |
| Basophils (10^9/l) | 0.00 ± 0.00 | 0.02 ± 0.08 | 0.263 |
| **Reticulocytes (10^9/l)** | **220.91 ± 11.05** | **187.55 ± 33.76** | **0.028** |
| **IRF (%)** | **36.96 ± 2.46** | **30.58 ± 5.57** | **0.038** |
| **Ret-He (pg)** | **18.54 ± 0.15** | **17.98 ± 0.65** | **0.020** |

Mice were treated with DZNep for 8 weeks. Blood analysis were done 2 weeks after the end of injections. Values represents means ± SEM. *n* = 15 for control group; *n* = 14 for DZNep group.
Abbreviations: WBC: white blood cell count, RBC: red blood cell count, HGB: hemoglobin, MCV: mean corpuscular volume, PDW: Platelet distribution Width, IRF: Immature Reticulocyte fraction; Ret-He: reticulocyte hemoglobinemia content.

## 17. GENERAL TOXICITY: MICE

### 17.1 Body weight changes in mice during DZNep treatment

Wee ZN et al., EZH2-mediated inactivation of IFN-γ-JAK-STAT1 signaling is an effective therapeutic target in MYC-driven prostate cancer. Cell Rep 2014;10:204-216.

Treatment with 3-deazaneplanocin A (DZNep), interferon gamma (IFN-γ) alone and in combination were well tolerated in nude mice with DU145 prostate tumor xenografts and without overt signs of toxicity or weight loss of >10%, supporting the potential application of this treatment in the clinic (Figure 6B).

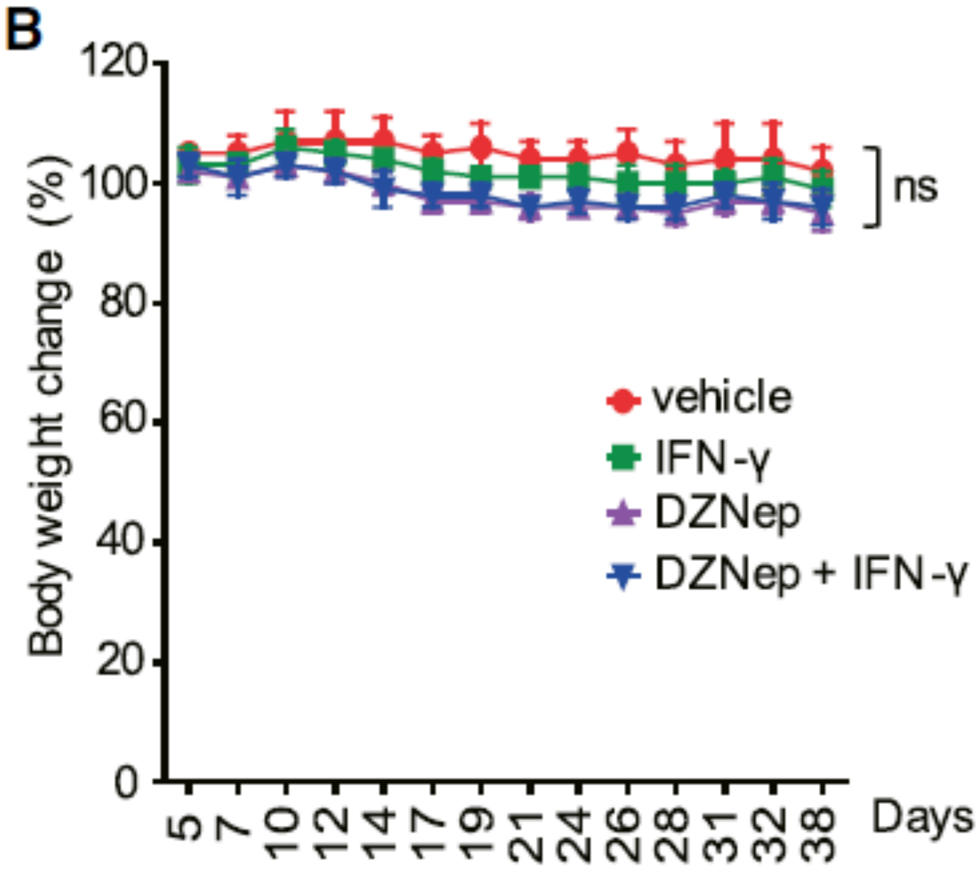


**Figure 6B**. Effects of 3-deazanneplanocin A (DZNep) and/or interferon gamma (IFN- γ) on body weight changes in male athymic nude mice with DU145 prostate tumor xenografts. The mice were treated with vehicle (n = 5), IFN- γ (1 x $10^7$ IU/kg, i.p.; n = 6), DZNep (1 mg/kg, s.c.; n = 7) (every alternating day for 38 days), or both (n = 8). Body weight change (± SEM) are shown during the drug treatment.

# 18. GENERAL TOXICITY: MONKEYS

## 18.1 Experimental Protocol and Letter to Marquez from Bray

Letter (August 28, 2008) of Mike Bray, MD MPI Medical Officer' Integrated Research Facility NIAID/ NIH to Dr Dr. Victor Marquez Chief; Laboratory of Medicinal Chemistry Center for Cancer Research, NCI

The toxicity and antiviral activity against the Ebola Zaire virus of DZNep was investigated in monkeys (cynomolgus macaques) as described below:

Non-infective monkeys were treated with the following doses by intramuscular injection:

1) 0.3 mg/kg daily x 4 days

2) 0,6 mg/kg daily x 4 days

3) 1.2 mg/kg on day 0, followed by 0.6 mg/kg on days 1,2 and 3

4) 1.2 mg/kg daily x 4 days

5) 2 mg/kg on days 0, 2 and 4

6) 2.4 mg/kg on day 0

The animals were observed daily and blood samples were collected every 2-3 days for upto l l days after the begining of treatment. Tests included hemoglobin and hematocrit, total white blood cell count and differential and serum chemistry. None of the treatment regimens produced behavioral changes such as decreased activity or food intake or other signs of toxicity. Hematologic and chemical parameters remained within the nomal range for regimens 1, 2, 3 and 6. However, the monkeys that received repeated doses of 1.2 or 2 mg/kg of drug in regimens 4 and 5 showed modest increases in alanine aminotransferase (ALT) and aspartate aminotransferase (AST) after the commencement of treatment, suggestive of moderate hepatotoxicity. These values returned to normal after cessation of therapy. Alkaline phosphatase, gamma glutamyltranspeptidase and total bilirubin were unaffected. No other abnormalities were observed. This small study provides limited evidence that 3-deazaneplanocin A, given in repeated doses of 1.2 or 2 mg/kg to healthy monkeys, causes transient elevations of some liver-associated enzymes in the serum, suggestive of minor hepatotoxicity.

DEPARTMENT OF HEALTH & HUMAN SERVICES

PUBLIC HEALTH SERVICE
NATIONAL INSTITUTES OF HEALTH
Integrated Research Facility
Office of Clinical Research/NIAID
6700A Rockledge Drive, Room 5128
Bethesda MD 20892
Tel: (301) 451-5123
Fax: (301) 480-2319

August 28th, 2008

Dr. Victor Marquez
Chief, Laboratory of Medicinal Chemistry
Center for Cancer Research, NCI

Dear Victor,

Ten years ago, while working in the laboratory of Dr. John Huggins at the US Army Medical Research Institute of Infectious Diseases (USAMRIID), I discovered that the compound 3-deazaneplanocin A was highly effective in treating mice infected with a mouse-adapted variant of Ebola Zaire virus. This virus causes uniformly fatal disease when inoculated intraperitoneally in adult immunocompetent mice, with the onset of weight loss on day 3 and death by day 6 [1]. Animals treated with thrice-daily doses of 3-deazaneplanocin A, beginning at the time of infection, were protected against illness and death, as were those that received a single injection of 1 mg/kg given on day 0, 1 or 2 with respect to virus challenge [2,3]. Measurement of plasma interferon-$\alpha$ levels indicated that protection was mediated through markedly enhanced interferon production by infected cells; no increase in interferon was detected in uninfected, treated mice [4]. The drug produced no signs of toxicity at any of the doses used.

There is no effective antiviral therapy for Ebola virus infection of humans. Because it would be highly desirable to have a medication that could be given by injection only once or a few times, I decided to evaluate the efficacy of 3-deazaneplanocin A in nonhuman primates lethally infected with wild-type Ebola Zaire virus, using a regimen similar to that which had proved effective in mice. Under USAMRIID protocol V99-06, I first tested the compound for toxicity in uninfected cynomolgus macaques. One or two animals were treated with the following doses by intramuscular injection:

1. 0.3 mg/kg daily x 4 days
2. 0.6 mg/kg daily x 4 days
3. 1.2 mg/kg on day 0, followed by 0.6 mg/kg on days 1, 2 and 3
4. 1.2 mg/kg daily x 4 days
5. 2 mg/kg on days 0, 2 and 4
6. 2.4 mg/kg on day 0

The animals were observed daily and blood samples were collected every 2-3 days for up to 11 days after the beginning of treatment. Tests included hemoglobin and hematocrit, total white blood cell count and differential and a serum chemistry panel.

None of the treatment regimens produced behavioral changes such as decreased activity or food intake or other signs of toxicity. Hematologic and chemical parameters remained within the normal range for regimens 1, 2, 3 and 6. However, macaques that received repeated doses of 1.2 or 2 mg/kg of drug in regimens 4 and 5 showed modest increases in alanine aminotransferase (ALT) and aspartate aminotransferase (AST) after the commencement of treatment, suggestive of hepatotoxicity. These values returned to normal after cessation of therapy. Alkaline phosphatase, gamma glutamyl transpeptidase and total bilirubin were unaffected. No other abnormalities were observed.

After completing these limited toxicity studies, I proceeded to a small-scale trial of 3-deazaneplanocin A in cynomolgus macaques infected with wild-type Ebola Zaire virus isolated during the 1995 Kikwit outbreak. The inoculation of this virus into macaques results in all cases in the onset of visible illness by day 3 and death by day 6 or 7. In an effort to ensure the success of therapy for these expensive animals, I decided not to limit treatment to the single dose I had employed in mice, but to give several doses, beginning on the day after virus challenge. In the first experiment, 3 macaques were inoculated intramuscularly with 1000 plaque-forming units of Ebola virus, then given 1.2 mg/kg of drug on day 1 postinfection, followed by 0.6 mg/kg on days 2-4 (regimen 3 above, which had produced no toxic effects in uninfected macaques). Unfortunately, treatment produced no benefit: a placebo-treated animal died on day 7, while the three macaques that received 3-deazaneplanocin A died on days 7, 7 and 8. Liver function tests (AST, ALT and alkaline phosphatase) were somewhat higher on day 5 postinfection in the three treated animals than in the untreated control, suggesting that hepatic injury from viral infection may have potentiated drug toxicity. Similar results were obtained in a second experiment, in which 3-deazaneplanocin A was administered to 3 virus-infected macaques on days 1, 3 and 5 postinfection. In both experiments, there was no evidence that treatment stimulated interferon production, suggesting that the drug's mechanism of action in mice is not duplicated in nonhuman primates.

This small study provides limited evidence that 3-deazaneplanocin A, given in repeated doses of 1.2 or 2 mg/kg to healthy cynomolgus macaques, causes transient elevations of some liver-associated enzymes in the serum, suggestive of hepatotoxicity. The regimens were modeled on experience with single-dose treatment of mice, and were not optimized for nonhuman primates; it is possible that some degree of protection against Ebola virus might have been achieved with smaller doses of drug given several times a day. However, the absence of enhanced interferon production in infected, treated macaques suggests that the mechanism of protection in mice is not operative in primates.

Sincerely,

Mike Bray, MD MPH
Medical Officer
Integrated Research Facility
NIAID/NIH

## 18.2 DZNep 0.3 mg/kg i.m. qd x 4: blood chemistry, liver function, hematology

CONFIDENTIAL

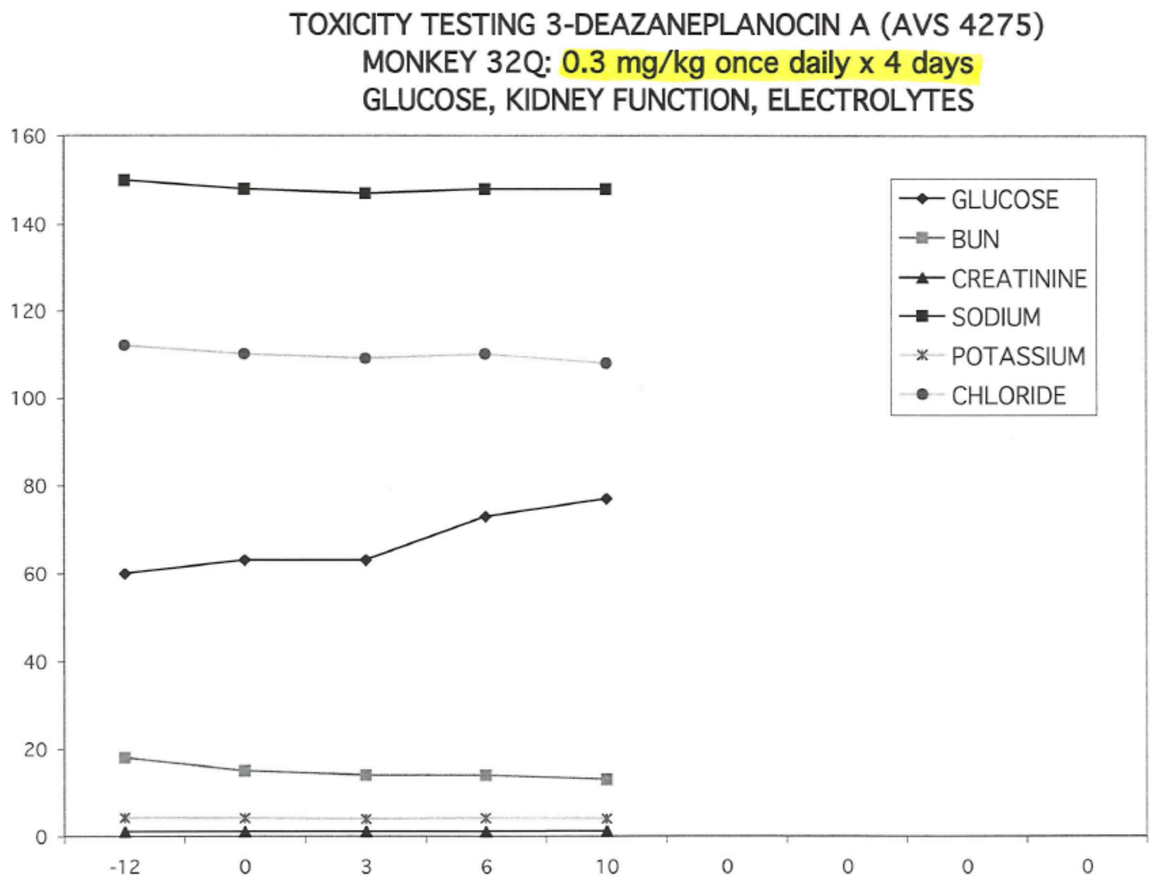

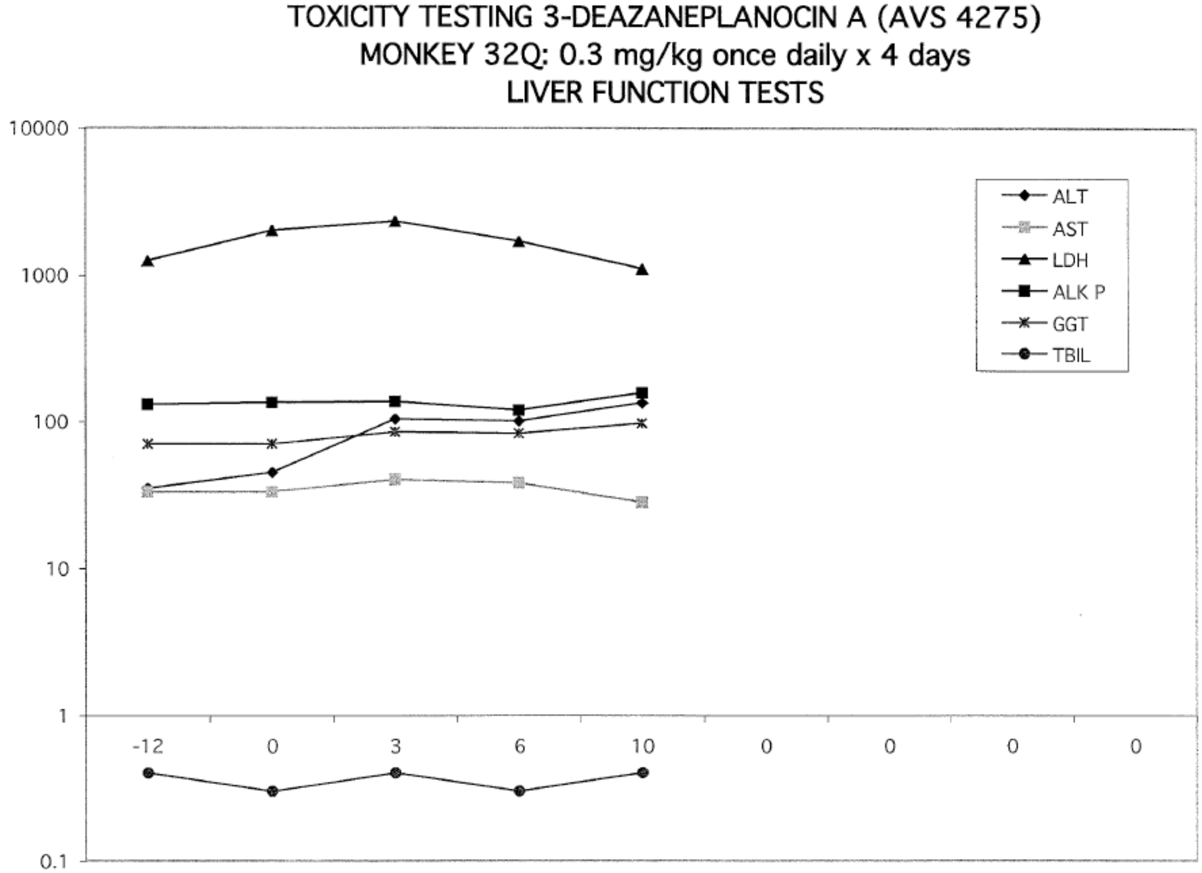

TOXICITY TESTING 3-DEAZANEPLANOCIN A (AVS 4275)
MONKEY 32Q: 0.3 mg/kg once daily x 4 days
LIVER FUNCTION TESTS
10000
1000
100
10
1
0.1
-12
0
3
6
10
0
0
0
0
ALT
AST
LDH
ALK P
GGT
TBIL

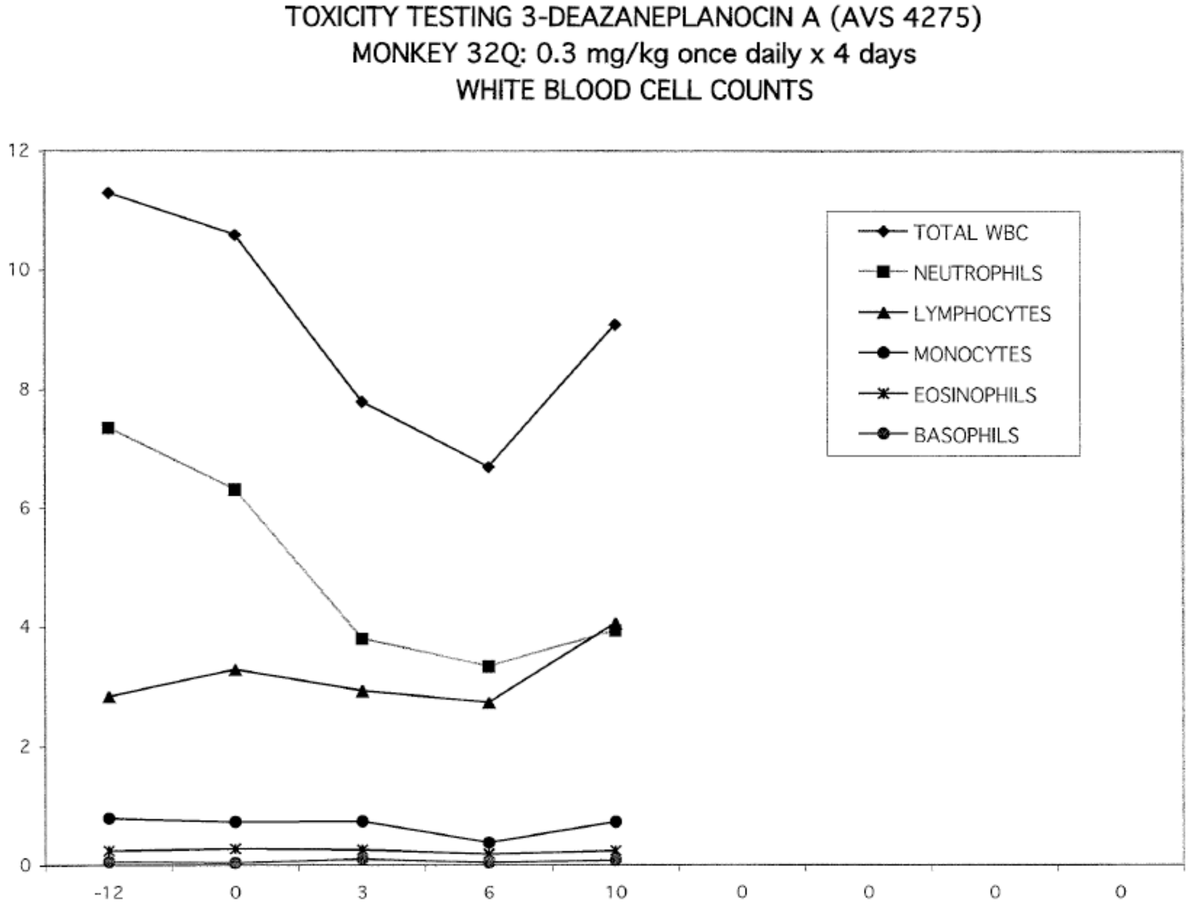
TOXICITY TESTING 3-DEAZANEPLANOCIN A (AVS 4275)
MONKEY 32Q: 0.3 mg/kg once daily x 4 days
WHITE BLOOD CELL COUNTS
12
10
8
6
4
2
0
-12
0
3
6
10
0
0
0
0
TOTAL WBC
NEUTROPHILS
LYMPHOCYTES
MONOCYTES
EOSINOPHILS
BASOPHILS

TOXICITY TESTING 3-DEAZANEPLANOCIN A (AVS 4275)
MONKEY 32Q: 0.3 mg/kg once daily x 4 days
HEMOGLOBIN, HEMATOCRIT, BODY WEIGHT

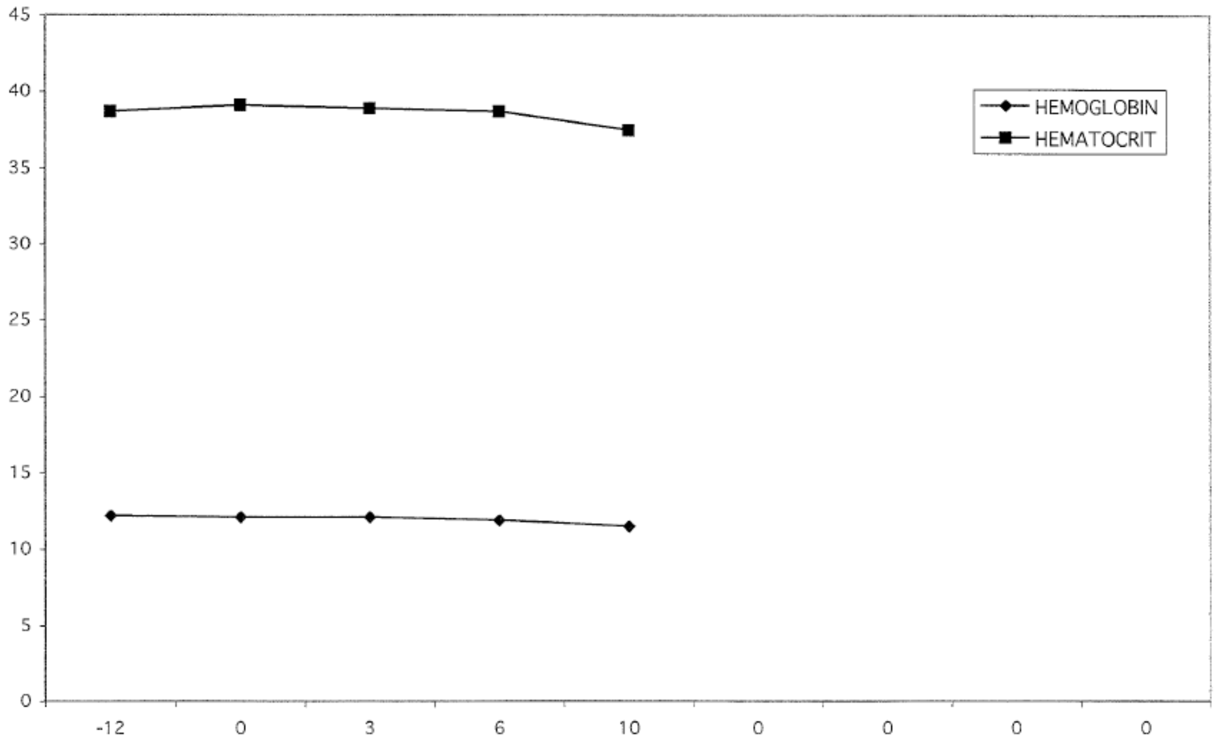

Monkey ID: 32Q

Dose of AVS 4275 QD: 0.3 MG/KG INJECTED I.M. ONCE DAILY X 4 DAYS

| | Prebleed | Day 0 | 1 | 2 | 3 | 4 | 5 | 6 | 7 |
|---|---|---|---|---|---|---|---|---|---|
| Bleed dates | 7/1/99 | 7/13/99 | 7/16/99 | 7/19/99 | 7/23/99 | | | | |
| | -12 | 0 | 3 | 6 | 10 | | | | |
| GLU | 60 | 63 | 63 | 73 | 77 | | | | |
| BUN | 18 | 15 | 14 | 14 | 13 | | | | |
| CREA | 1.1 | 1.1 | 1.1 | 1.1 | 1.2 | | | | |
| NA | 150 | 148 | 147 | 148 | 148 | | | | |
| K | 4.2 | 4.1 | 3.9 | 4.1 | 4 | | | | |
| CL | 112 | 110 | 109 | 110 | 108 | | | | |
| CA | 10.2 | 10.1 | 9.8 | 9.9 | 9.7 | | | | |
| PHOS | 4.4 | 4.8 | 4.8 | 3.7 | 4.3 | | | | |
| TP | 7.3 | 7.2 | 7.4 | 7.1 | 7.3 | | | | |
| ALB | 3.9 | 3.8 | 4 | 3.8 | 4.1 | | | | |
| AST | 33 | 33 | 40 | 38 | 28 | | | | |
| ALT | 35 | 45 | 104 | 101 | 135 | | | | |
| LDH | 1262 | 2032 | 2352 | 1722 | 1111 | | | | |
| ALK P | 132 | 136 | 138 | 120 | 158 | | | | |
| GGT | 70 | 70 | 85 | 83 | 97 | | | | |
| T BIL | 0.4 | 0.3 | 0.4 | 0.3 | 0.4 | | | | |
| CHOL | 145 | 136 | 117 | 123 | 128 | | | | |
| TRIG | 44 | 45 | 36 | 28 | 46 | | | | |
| WBC | 11.3 | 10.6 | 7.8 | 6.7 | 9.1 | | | | |
| RBC | 5.81 | 5.88 | 5.9 | 5.87 | 5.65 | | | | |
| HGB | 12.2 | 12.1 | 12.1 | 11.9 | 11.5 | | | | |
| HCT | 38.7 | 39.1 | 38.9 | 38.7 | 37.5 | | | | |
| MCV | | | | | | | | | |
| MCH | | | | | | | | | |
| MCHC | | | | | | | | | |
| RDW | | | | | | | | | |
| PLT | 422 | 443 | 421 | 404 | 408 | | | | |
| MPV | | | | | | | | | |
| % NEU | | | | | | | | | |
| % LYM | | | | | | | | | |
| % MONO | | | | | | | | | |
| % EOSIN | | | | | | | | | |
| % BASO | | | | | | | | | |
| ABS NEU | 7.35 | 6.31 | 3.8 | 3.34 | 3.94 | | | | |
| ABS. LYMPH | 2.83 | 3.29 | 2.93 | 2.74 | 4.06 | | | | |
| ABS. MONO | 0.79 | 0.73 | 0.74 | 0.38 | 0.73 | | | | |
| ABS. EOSIN | 0.24 | 0.28 | 0.26 | 0.19 | 0.24 | | | | |
| ABS. BASO | 0.055 | 0.034 | 0.095 | 0.046 | 0.075 | | | | |

**18.3 DZNep 0.6 mg/kg i.m. qd x 4: blood chemistry, liver function, hematology**

CONFIDENTIAL

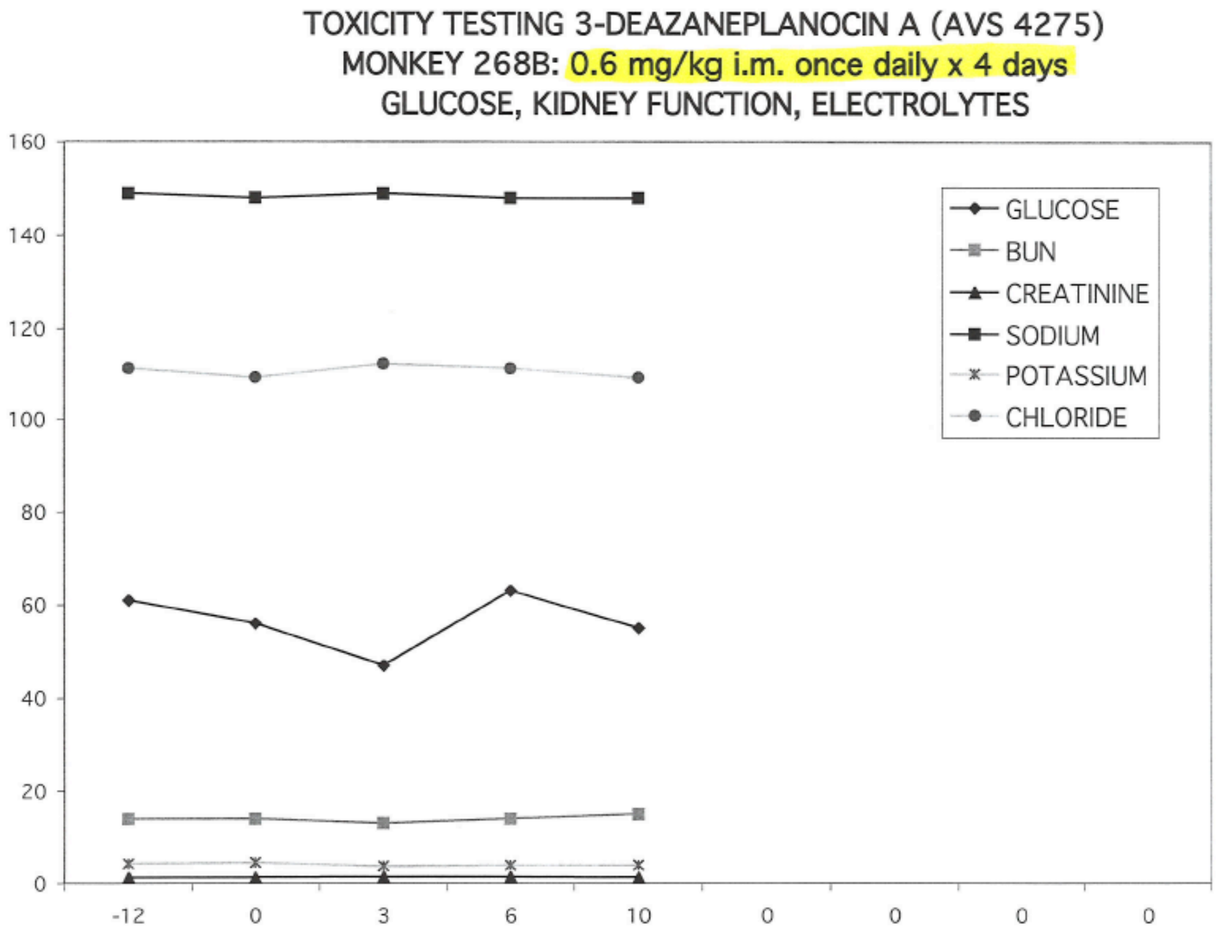

GRAPH 1: GLUCOSE, BUN, CR, ELECTROLYTES

| | GLUCOSE | BUN | CREATININ | SODIUM | POTASSIU | CHLORIDE |
|---|---|---|---|---|---|---|
| -12 | 60 | 18 | 1.1 | 150 | 4.2 | 112 |
| 0 | 63 | 15 | 1.1 | 148 | 4.1 | 110 |
| 3 | 63 | 14 | 1.1 | 147 | 3.9 | 109 |
| 6 | 73 | 14 | 1.1 | 148 | 4.1 | 110 |
| 10 | 77 | 13 | 1.2 | 148 | 4 | 108 |
| 0 | | | | | | |
| 0 | | | | | | |
| 0 | | | | | | |
| 0 | | | | | | |

GRAPH 2: LIVER FUNCTION

| | ALT | AST | LDH | ALK P | GGT | TBIL |
|---|---|---|---|---|---|---|
| -12 | 35 | 33 | 1262 | 132 | 70 | 0.4 |
| 0 | 45 | 33 | 2032 | 136 | 70 | 0.3 |
| 3 | 104 | 40 | 2352 | 138 | 85 | 0.4 |
| 6 | 101 | 38 | 1722 | 120 | 83 | 0.3 |
| 10 | 135 | 28 | 1111 | 158 | 97 | 0.4 |
| 0 | | | | | | |
| 0 | | | | | | |
| 0 | | | | | | |
| 0 | | | | | | |

GRAPH 3: BLOOD CELL COUNTS

| | TOTAL WE | NEUTROPH | LYMPHOC | MONOCYT | EOSINOPH | BASOPHILS |
|---|---|---|---|---|---|---|
| -12 | 11.3 | 7.35 | 2.83 | 0.79 | 0.24 | 0.055 |
| 0 | 10.6 | 6.31 | 3.29 | 0.73 | 0.28 | 0.034 |
| 3 | 7.8 | 3.8 | 2.93 | 0.74 | 0.26 | 0.095 |
| 6 | 6.7 | 3.34 | 2.74 | 0.38 | 0.19 | 0.046 |
| 10 | 9.1 | 3.94 | 4.06 | 0.73 | 0.24 | 0.075 |
| 0 | | | | | | |
| 0 | | | | | | |
| 0 | | | | | | |
| 0 | | | | | | |

GRAPH 4: HEMOGLOBIN, HEMATOCRIT

| | HEMOGLOI | HEMATOCRIT |
|---|---|---|
| -12 | 12.2 | 38.7 |
| 0 | 12.1 | 39.1 |
| 3 | 12.1 | 38.9 |
| 6 | 11.9 | 38.7 |
| 10 | 11.5 | 37.5 |
| 0 | | |
| 0 | | |

CONFIDENTIAL

TOXICITY TESTING 3-DEAZANEPLANOCIN A (AVS 4275)
MONKEY 268B: 0.6 mg/kg i.m. once daily x 4 days
GLUCOSE, KIDNEY FUNCTION, ELECTROLYTES

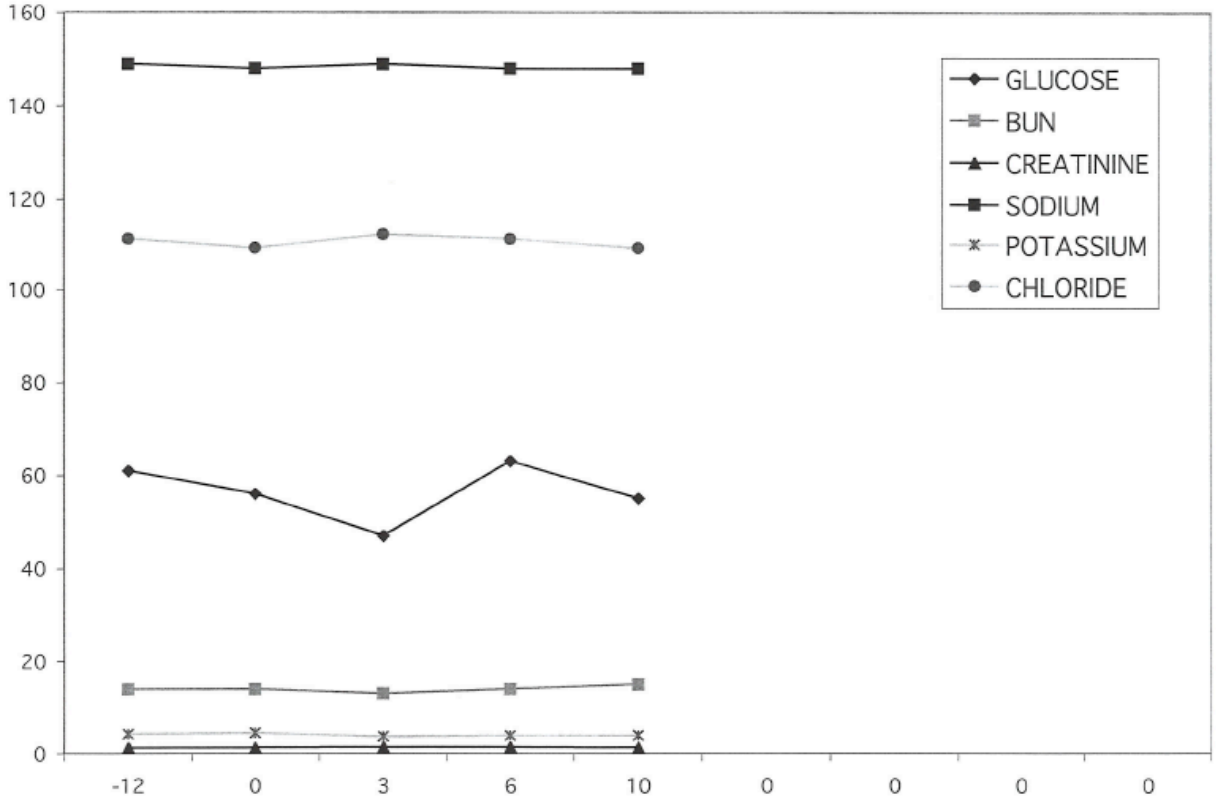

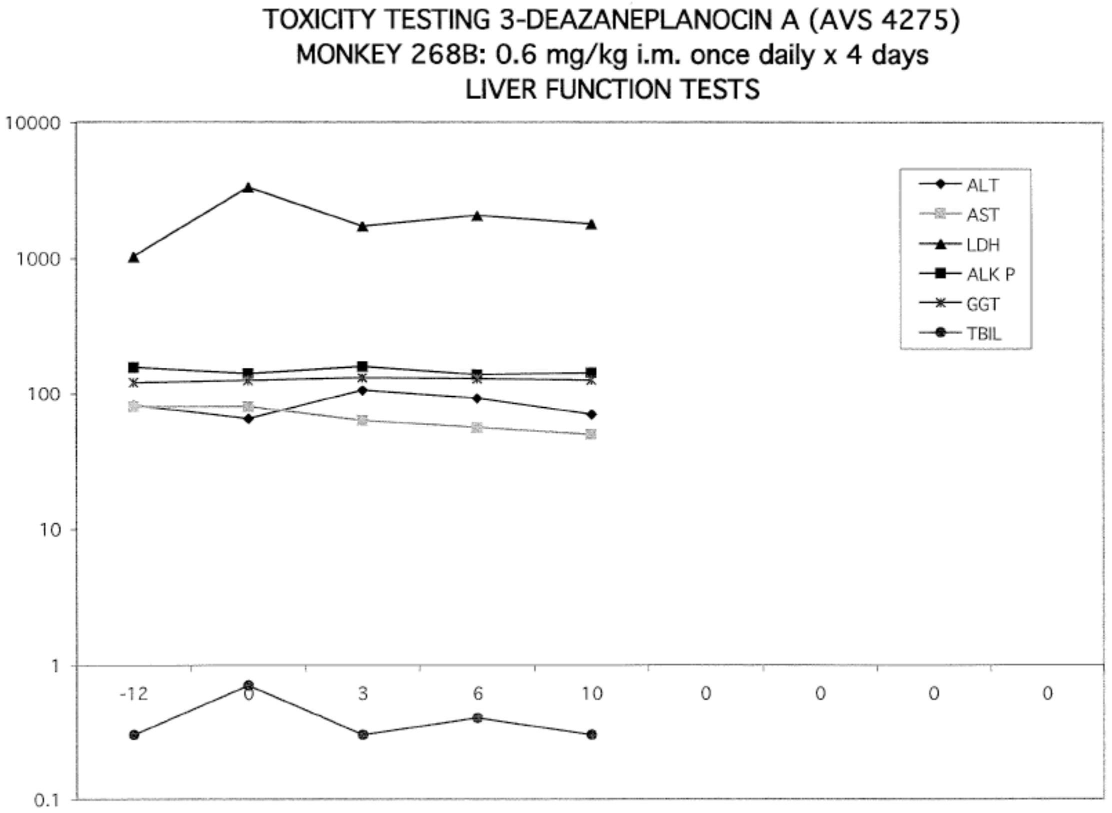
TOXICITY TESTING 3-DEAZANEPLANOCIN A (AVS 4275)
MONKEY 268B: 0.6 mg/kg i.m. once daily x 4 days
LIVER FUNCTION TESTS
10000
1000
100
10
1
0.1
-12
0
3
6
10
0
0
0
0
ALT
AST
LDH
ALK P
GGT
TBIL

TOXICITY TESTING 3-DEAZANEPLANOCIN A (AVS 4275)
MONKEY 268B: 0.6 mg/kg i.m. once daily x 4 days
WHITE BLOOD CELL COUNTS

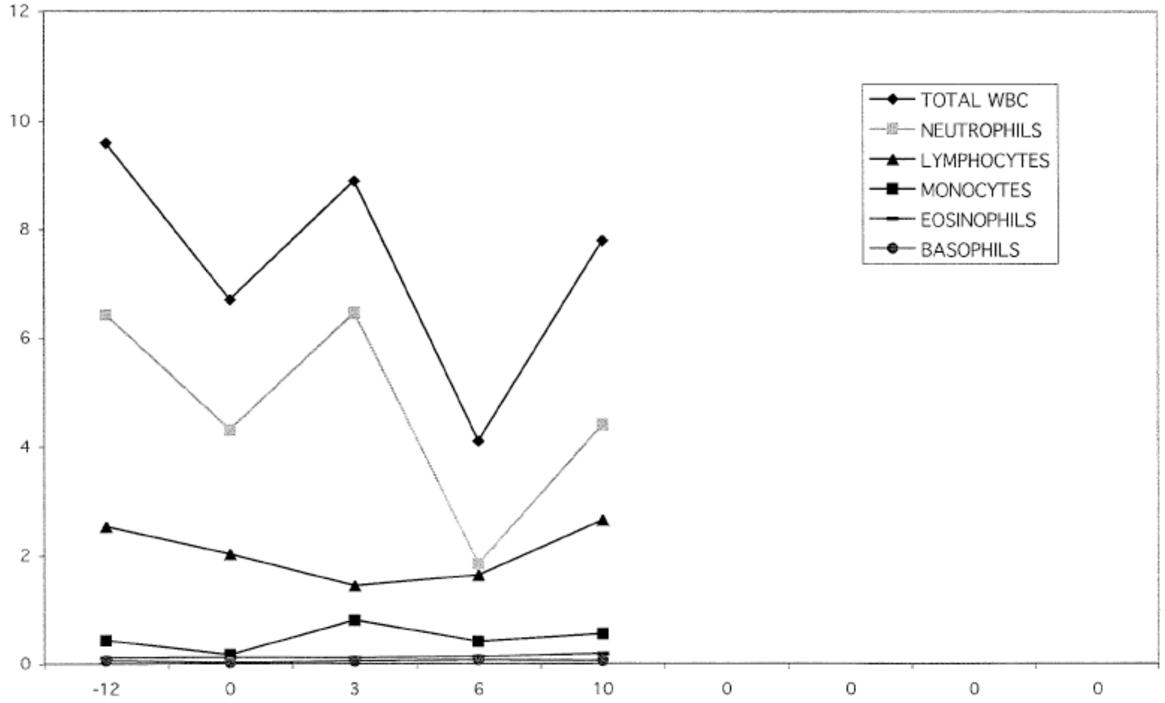

TOXICITY TESTING 3-DEAZANEPLANOCIN A (AVS 4275)
MONKEY 268B: 0.6 mg/kg i.m. once daily x 4 days
HEMOGLOBIN, HEMATOCRIT, BODY WEIGHT

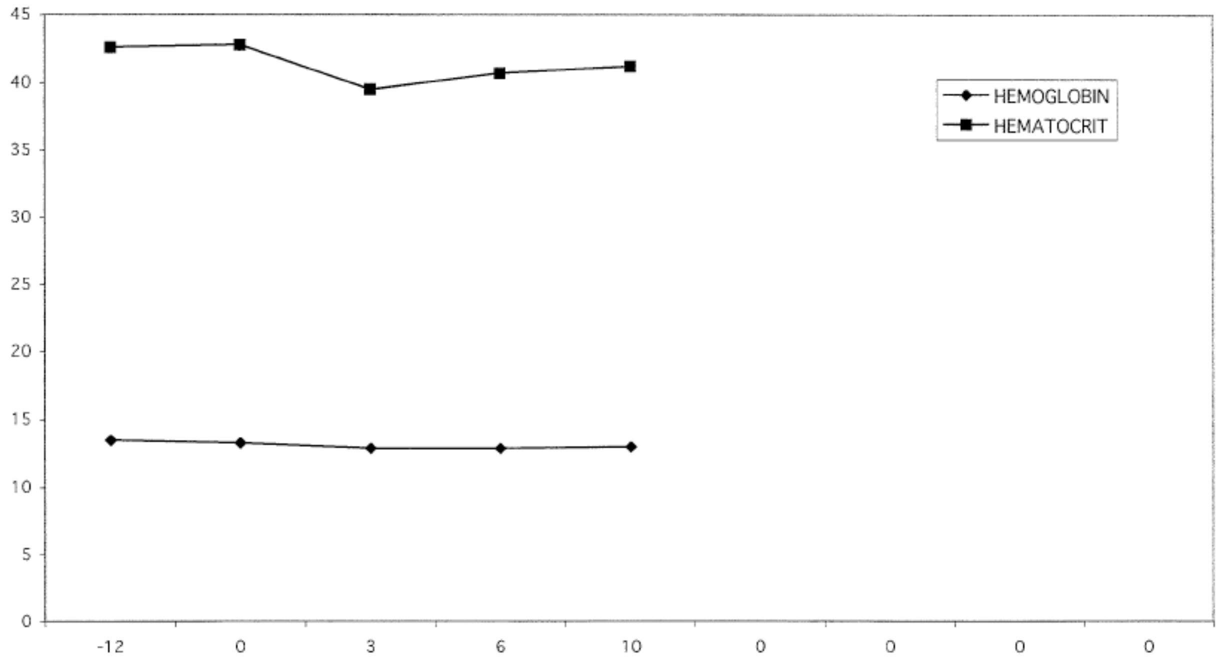

Monkey ID 268B

Dose of AVS 4275 QD 0.6 MG/KG INJECTED I.M. ONCE DAILY X 4 DAYS

| Bleed dates | Prebleed 7/1/99 | Day 0 7/13/99 | 7/16/99 | 7/19/99 | 7/23/99 |
|---|---|---|---|---|---|
| | -12 | 0 | 3 | 6 | 10 |
| GLU | 61 | 56 | 47 | 63 | 55 |
| BUN | 14 | 14 | 13 | 14 | 15 |
| CREA | 1.3 | 1.3 | 1.4 | 1.4 | 1.3 |
| NA | 149 | 148 | 149 | 148 | 148 |
| K | 4.2 | 4.4 | 3.6 | 3.9 | 3.8 |
| CL | 111 | 109 | 112 | 111 | 109 |
| CA | 9.7 | 9.6 | 9.3 | 9.3 | 9.4 |
| PHOS | 2.9 | 3.1 | 4.5 | 2.9 | 3.2 |
| TP | 7.3 | 7.4 | 7.3 | 7.4 | 7.5 |
| ALB | 3.4 | 3.5 | 3.5 | 3.5 | 3.6 |
| AST | 80 | 80 | 63 | 56 | 50 |
| ALT | 82 | 65 | 106 | 92 | 70 |
| LDH | 1031 | 3336 | 1725 | 2082 | 1793 |
| ALK P | 157 | 141 | 159 | 139 | 143 |
| GGT | 120 | 125 | 131 | 129 | 126 |
| T BIL | 0.3 | 0.7 | 0.3 | 0.4 | 0.3 |
| CHOL | 90 | 82 | 54 | 66 | 92 |
| TRIG | 50 | 40 | 31 | 40 | 65 |

| | | | | | |
|---|---|---|---|---|---|
| WBC | 9.6 | 6.7 | 8.9 | 4.1 | 7.8 |
| RBC | 6.33 | 6.37 | 5.9 | 6.07 | 6.11 |
| HGB | 13.5 | 13.3 | 12.9 | 12.9 | 13 |
| HCT | 42.6 | 42.8 | 39.5 | 40.7 | 41.2 |
| MCV | | | | | |
| MCH | | | | | |
| MCHC | | | | | |
| RDW | | | | | |
| PLT | 278 | 244 | 236 | 221 | 246 |
| MPV | | | | | |

| | | | | | |
|---|---|---|---|---|---|
| % NEU | | | | | |
| % LYM | | | | | |
| % MONO | | | | | |
| % EOSIN | | | | | |
| % BASO | | | | | |
| ABS NEU | 6.42 | 4.3 | 6.45 | 1.85 | 4.4 |
| ABS. LYMPH | 2.54 | 2.03 | 1.45 | 1.65 | 2.65 |
| ABS. MONO | 0.44 | 0.17 | 0.81 | 0.42 | 0.56 |
| ABS. EOSIN | 0.11 | 0.12 | 0.11 | 0.13 | 0.19 |
| ABS. BASO | 0.052 | 0.022 | 0.044 | 0.072 | 0.052 |

| | |
|---|---|
| BODY WT | |

GRAPH 1: GLUCOSE, BUN, CR, ELECTROLYTES

| | GLUCOSE | BUN | CREATININ | SODIUM | POTASSIU | CHLORIDE |
|---|---|---|---|---|---|---|
| -12 | 61 | 14 | 1.3 | 149 | 4.2 | 111 |
| 0 | 56 | 14 | 1.3 | 148 | 4.4 | 109 |
| 3 | 47 | 13 | 1.4 | 149 | 3.6 | 112 |
| 6 | 63 | 14 | 1.4 | 148 | 3.9 | 111 |
| 10 | 55 | 15 | 1.3 | 148 | 3.8 | 109 |
| 0 | | | | | | |
| 0 | | | | | | |
| 0 | | | | | | |
| 0 | | | | | | |

GRAPH 2: LIVER FUNCTION

| | ALT | AST | LDH | ALK P | GGT | TBIL |
|---|---|---|---|---|---|---|
| -12 | 82 | 80 | 1031 | 157 | 120 | 0.3 |
| 0 | 65 | 80 | 3336 | 141 | 125 | 0.7 |
| 3 | 106 | 63 | 1725 | 159 | 131 | 0.3 |
| 6 | 92 | 56 | 2082 | 139 | 129 | 0.4 |
| 10 | 70 | 50 | 1793 | 143 | 126 | 0.3 |
| 0 | | | | | | |
| 0 | | | | | | |
| 0 | | | | | | |
| 0 | | | | | | |

GRAPH 3: BLOOD CELL COUNTS

| | TOTAL WE | NEUTROPH | LYMPHOCY | MONOCYT | EOSINOPH | BASOPHILS |
|---|---|---|---|---|---|---|
| -12 | 9.6 | 6.42 | 2.54 | 0.44 | 0.11 | 0.052 |
| 0 | 6.7 | 4.3 | 2.03 | 0.17 | 0.12 | 0.022 |
| 3 | 8.9 | 6.45 | 1.45 | 0.81 | 0.11 | 0.044 |
| 6 | 4.1 | 1.85 | 1.65 | 0.42 | 0.13 | 0.072 |
| 10 | 7.8 | 4.4 | 2.65 | 0.56 | 0.19 | 0.052 |
| 0 | | | | | | |
| 0 | | | | | | |
| 0 | | | | | | |
| 0 | | | | | | |

GRAPH 4: HEMOGLOBIN, HEMATOCRIT, BODY WEIGHT

| | HEMOGLOI | HEMATOC | BODY WEIGHT |
|---|---|---|---|
| -12 | 13.5 | 42.6 | 0 |
| 0 | 13.3 | 42.8 | 0 |
| 3 | 12.9 | 39.5 | 0 |
| 6 | 12.9 | 40.7 | |
| 10 | 13 | 41.2 | |
| 0 | | | |
| 0 | | | |

**18.4 DZNep 1.2 mg/kg i.m. day 1 > 0.6 mg/kg days 2,3,4: blood, liver hematology**

CONFIDENTIAL

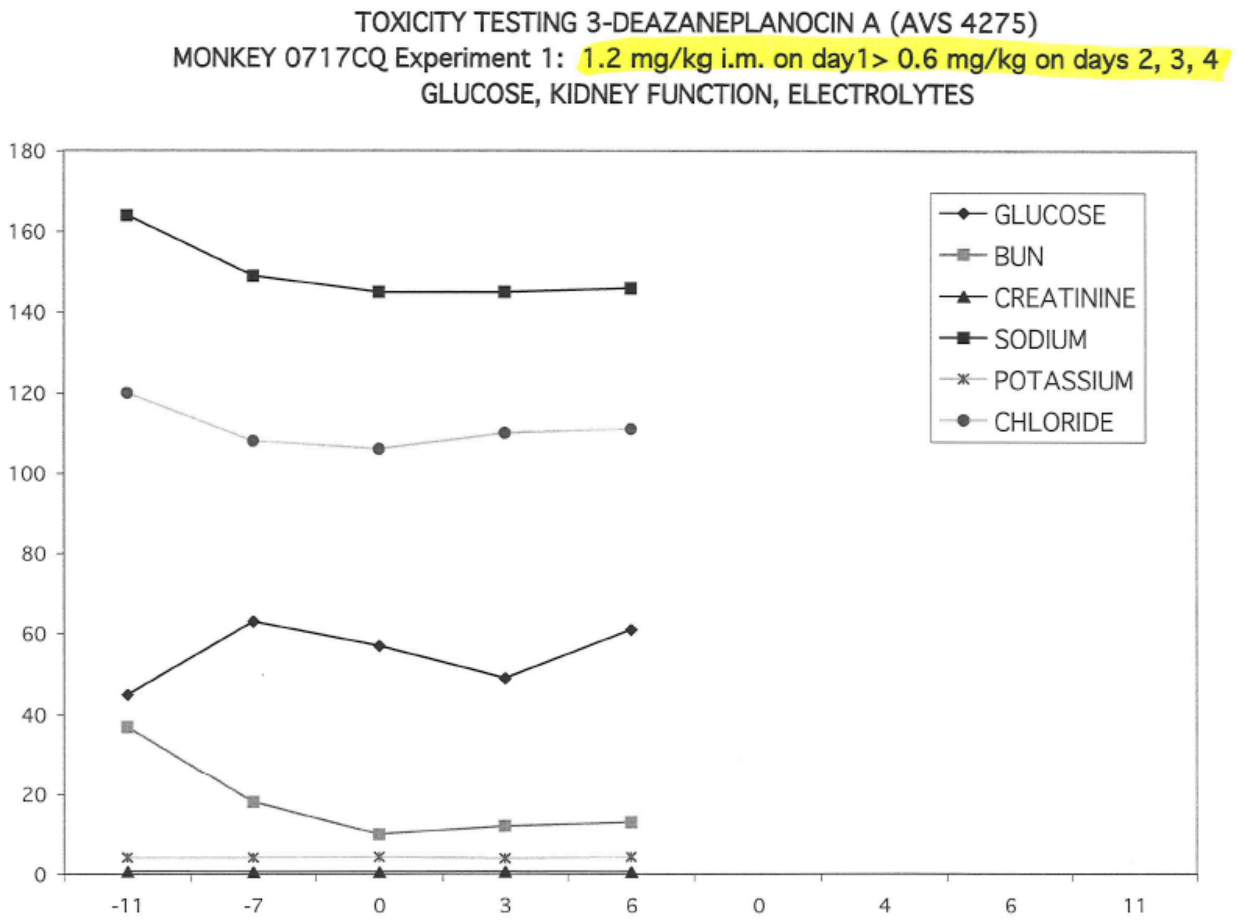

TOXICITY TESTING 3-DEAZANEPLANOCIN A (AVS 4275)
MONKEY 0717CQ Experiment 1: 1.2 mg/kg i.m. on day1> 0.6 mg/kg on days 2, 3, 4
WHITE BLOOD CELL COUNTS

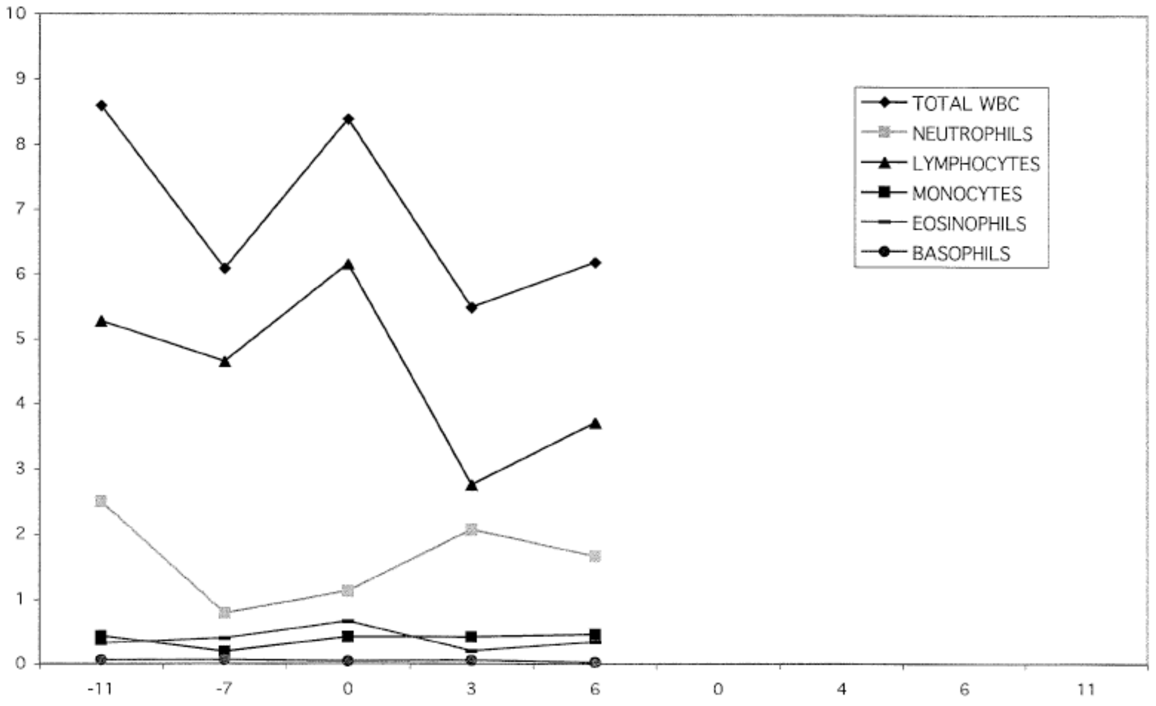

TOXICITY TESTING 3-DEAZANEPLANOCIN A (AVS 4275)
MONKEY 0717CQ Experiment 1: 1.2 mg/kg i.m. on day1 > 0.6 mg/kg on days 2, 3, 4
HEMOGLOBIN, HEMATOCRIT, BODY WEIGHT

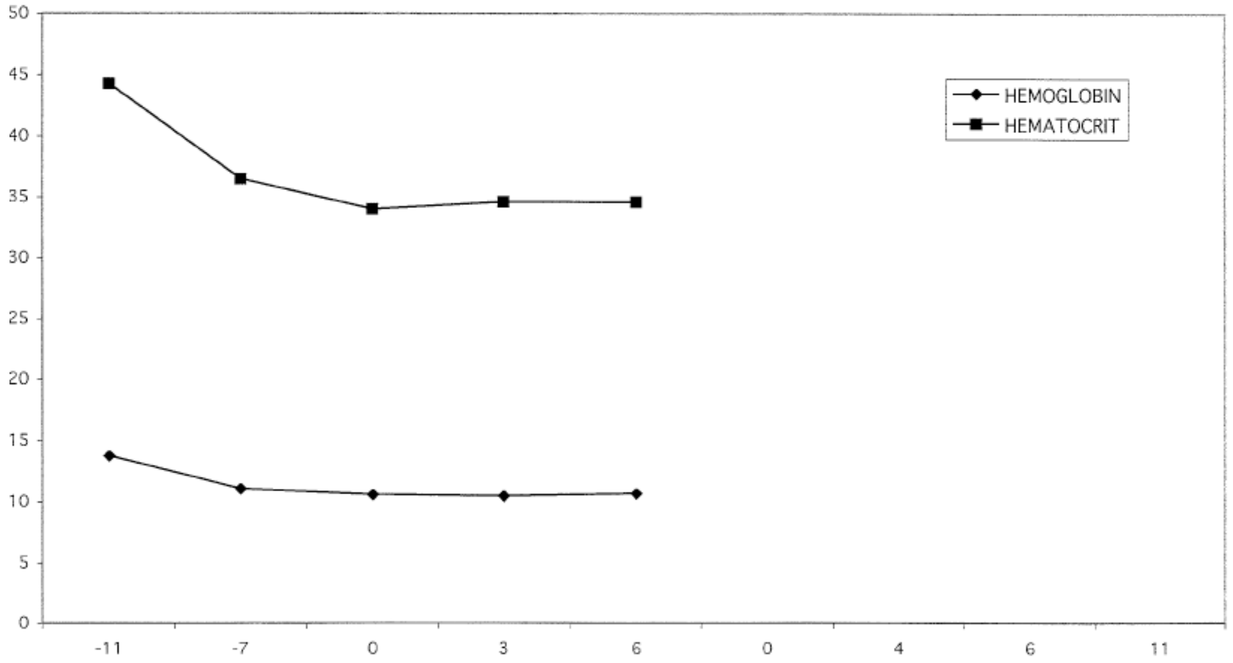

Monkey ID 0717CQ

Dose of AVS 4275 QD Experiment 1: 1.2 mg/kg i.m. on day1> 0.6 mg/kg on days 2, 3, 4
Experiment 2: 2 mg/kg i.m. on days 1, 3, 5

| Bleed dates | 7/23/99 | 7/27/99 | 8/3/99 | 8/6/99 | 8/9/99 | 10/6/99 | 10/8/99 | 10/13/99 |
|---|---|---|---|---|---|---|---|---|
| | -11 | -7 | 0 | 3 | 6 | 4 | 6 | 11 |
| GLU | 45 | 63 | 57 | 49 | 61 | 52 | 60 | 69 |
| BUN | 37 | 18 | 10 | 12 | 13 | 13 | 10 | 10 |
| CREA | 0.7 | 0.6 | 0.6 | 0.6 | 0.6 | 0.6 | 0.6 | 0.9 |
| NA | 164 | 149 | 145 | 145 | 146 | 147 | 145 | 148 |
| K | 4.1 | 4.1 | 4.3 | 3.9 | 4.2 | 4.1 | 4.1 | 4 |
| CL | 120 | 108 | 106 | 110 | 111 | 110 | 107 | 108 |
| CA | 10.4 | 9.5 | 9.4 | 9.4 | 9.6 | 9.3 | 9.7 | 9.6 |
| PHOS | 7.5 | 5.5 | 6.1 | 5.9 | 4.8 | 6.4 | 4.6 | 5.2 |
| TP | 8 | 6.7 | 6.6 | 6.6 | 6.7 | 6.8 | 6.7 | 7.4 |
| ALB | 4.4 | 3.5 | 3.3 | 3.4 | 3.3 | 3.7 | 3.7 | 4.2 |
| AST | 39 | 38 | 31 | 62 | 48 | 38 | 75 | 21 |
| ALT | 25 | 31 | 26 | 119 | 92 | 51 | 79 | 37 |
| LDH | 1763 | 1444 | 1093 | 1555 | 1330 | 1608 | 2273 | 737 |
| ALK P | 528 | 414 | 454 | 556 | 439 | 612 | 527 | 390 |
| GGT | 175 | 133 | 137 | 160 | 158 | 168 | 161 | 128 |
| T BIL | 0.4 | 0.3 | 0.2 | 0.3 | 0.3 | 0.2 | 0.2 | 0.4 |
| CHOL | 186 | 118 | 111 | 83 | 80 | 118 | 106 | 123 |
| TRIG | 36 | 22 | 27 | 29 | 10 | 35 | 25 | 35 |
| WBC | 8.6 | 6.1 | 8.4 | 5.5 | 6.2 | 8.3 | 5.1 | 5.3 |
| RBC | 6.92 | 5.75 | 5.4 | 5.5 | 5.44 | 5.62 | 5.49 | 5.47 |
| HGB | 13.8 | 11.1 | 10.6 | 10.5 | 10.7 | 11.4 | 11.2 | 11.3 |
| HCT | 44.3 | 36.5 | 34 | 34.6 | 34.6 | 36.5 | 35.8 | 35.3 |
| MCV | | | | | | | | |
| MCH | | | | | | | | |
| MCHC | | | | | | | | |
| RDW | | | | | | | | |
| PLT | 396 | 264 | 333 | 281 | 251 | | | |
| MPV | | | | | | | | |
| % NEU | | | | | | | | |
| % LYM | | | | | | | | |
| % MONO | | | | | | | | |
| % EOSIN | | | | | | | | |
| % BASO | | | | | | | | |
| ABS NEU | 2.5 | 0.79 | 1.13 | 2.07 | 1.67 | | | |
| ABS. LYMPH | 5.28 | 4.65 | 6.17 | 2.76 | 3.72 | | | |
| ABS. MONO | 0.44 | 0.19 | 0.42 | 0.42 | 0.47 | | | |
| ABS. EOSIN | 0.32 | 0.4 | 0.66 | 0.2 | 0.34 | | | |
| ABS. BASO | 0.057 | 0.06 | 0.035 | 0.054 | 0.024 | | | |
| BODY WT | | | | | | | | |

GRAPH 1: GLUCOSE, BUN, CR, ELECTROLYTES

| | GLUCOSE | BUN | CREATININ | SODIUM | POTASSIU | CHLORIDE |
|---|---|---|---|---|---|---|
| -11 | 45 | 37 | 0.7 | 164 | 4.1 | 120 |
| -7 | 63 | 18 | 0.6 | 149 | 4.1 | 108 |
| 0 | 57 | 10 | 0.6 | 145 | 4.3 | 106 |
| 3 | 49 | 12 | 0.6 | 145 | 3.9 | 110 |
| 6 | 61 | 13 | 0.6 | 146 | 4.2 | 111 |
| 0 | | | | | | |
| 4 | | | | | | |
| 6 | | | | | | |
| 11 | | | | | | |

GRAPH 2: LIVER FUNCTION

| | ALT | AST | LDH | ALK P | GGT | TBIL |
|---|---|---|---|---|---|---|
| -11 | 25 | 39 | 1763 | 528 | 175 | 0.4 |
| -7 | 31 | 38 | 1444 | 414 | 133 | 0.3 |
| 0 | 26 | 31 | 1093 | 454 | 137 | 0.2 |
| 3 | 119 | 62 | 1555 | 556 | 160 | 0.3 |
| 6 | 92 | 48 | 1330 | 439 | 158 | 0.3 |
| 0 | | | | | | |
| 4 | | | | | | |
| 6 | | | | | | |
| 11 | | | | | | |

GRAPH 3: BLOOD CELL COUNTS

| | TOTAL WI | NEUTROPH | LYMPHOC' | MONOCYT | EOSINOPH | BASOPHILS |
|---|---|---|---|---|---|---|
| -11 | 8.6 | 2.5 | 5.28 | 0.44 | 0.32 | 0.057 |
| -7 | 6.1 | 0.79 | 4.65 | 0.19 | 0.4 | 0.06 |
| 0 | 8.4 | 1.13 | 6.17 | 0.42 | 0.66 | 0.035 |
| 3 | 5.5 | 2.07 | 2.76 | 0.42 | 0.2 | 0.054 |
| 6 | 6.2 | 1.67 | 3.72 | 0.47 | 0.34 | 0.024 |
| 0 | | | | | | |
| 4 | | | | | | |
| 6 | | | | | | |
| 11 | | | | | | |

GRAPH 4: HEMOGLOBIN, HEMATOCRIT, BODY WEIGHT

| | HEMOGLOI | HEMATOCRIT |
|---|---|---|
| -11 | 13.8 | 44.3 |
| -7 | 11.1 | 36.5 |
| 0 | 10.6 | 34 |
| 3 | 10.5 | 34.6 |
| 6 | 10.7 | 34.6 |
| 0 | | |
| 4 | | |

# 19 POTENTIAL OF DZNep FOR IMMUNOTHEREPY OF CANCER

## 19.1 Inhibition of EZH2 synergizes with immunotherapy of cancer

Zingg D et al., The Histone Methyltransferase Ezh2 Controls Mechanisms of Adaptive Resistance to Tumor Immunotherapy. Cell Reports 2017;20:854–867.

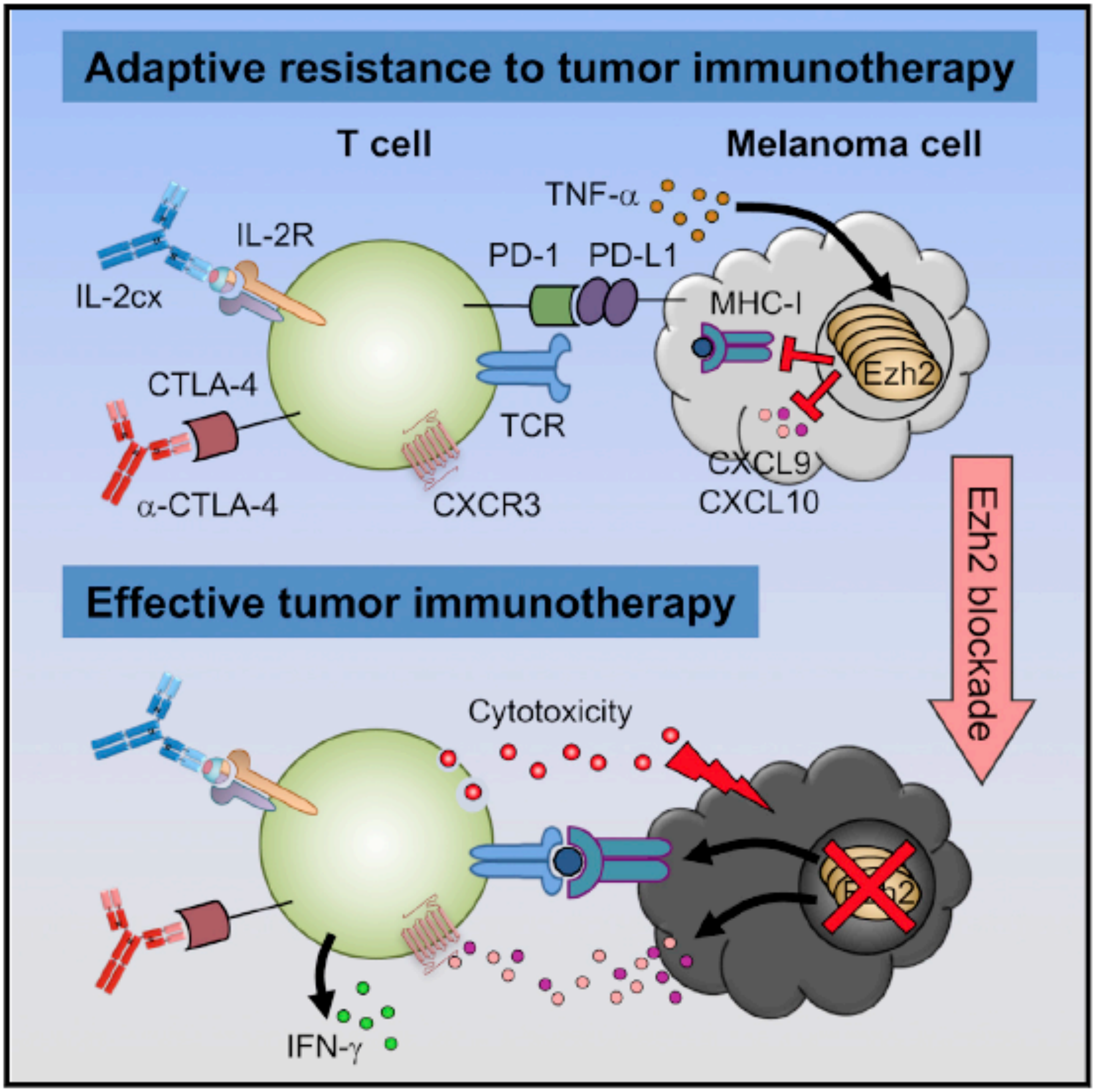


Intratumoral T cells and TNF-a cause Ezh2 upregulation in melanoma cells. Ezh2 silences immunogenicity and antigen presentation in melanoma. Ezh2 blockade reverses melanoma resistance mechanisms. Ezh2 inactivation synergizes with anti-CTLA-4 and IL-2 immunotherapy.

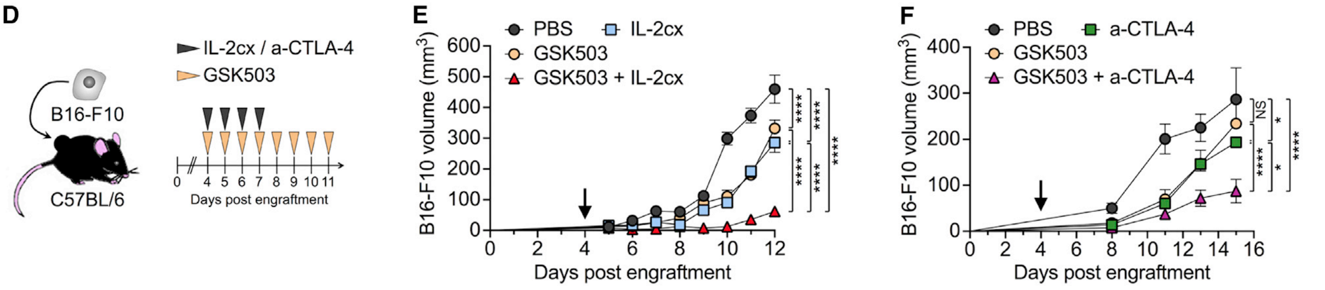


**Figure 3. Ezh2 Inactivation Synergizes with Anti-melanoma Immunotherapy**. Tumor growth kinetics in mice harboring wild type B16-F10 melanoma tumors. (**D**) Dose-schedule used for treatments. (**E**) The mice were treated with PBS, GSK503 (EZH2 inhibitor) and/or IL-2cx (IL-2/anti-IL-2 monoclonal antibody complexes). (**F**) The mice were treated with PBS, GSK503 (EZH2 inhibitor) and/or CTLA-4 (anti-CTLA-4 antibody).

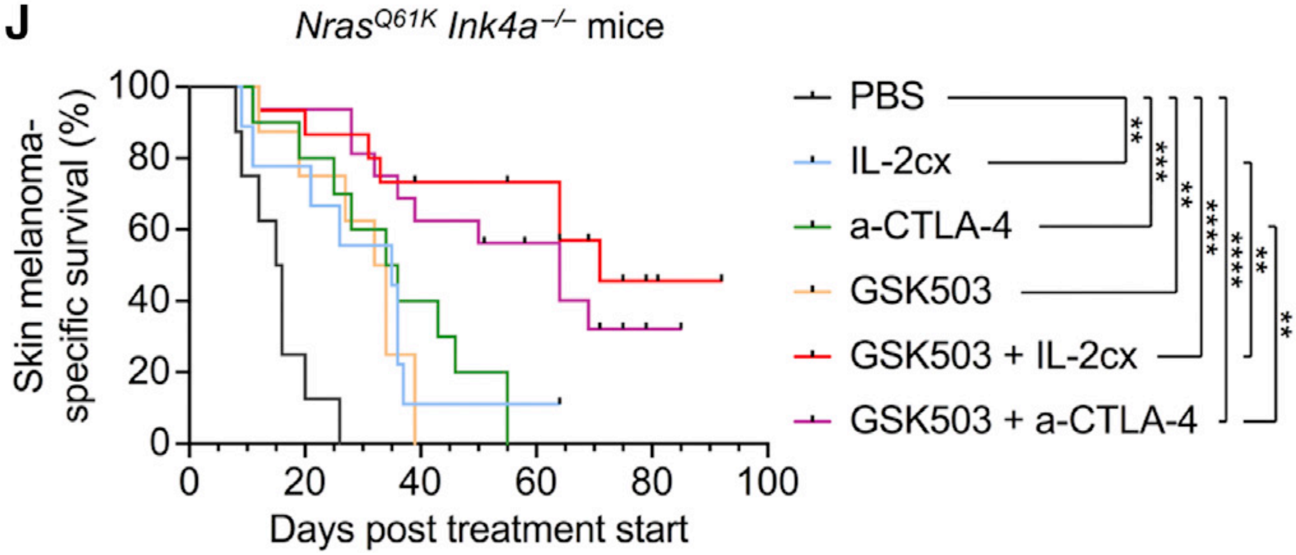


**Figure 3. Ezh2 Inactivation Synergizes with Anti-melanoma Immunotherapy**. (**J**) Kaplan-Meier survival curves comparing melanoma-specific survival of $Nras^{Q61K}$ $Ink4a^{-/-}$ mice treated with PBS, GSK503, IL-2cx, and/or a-CTL-4 using the dose-schedule shown in **(D)**.

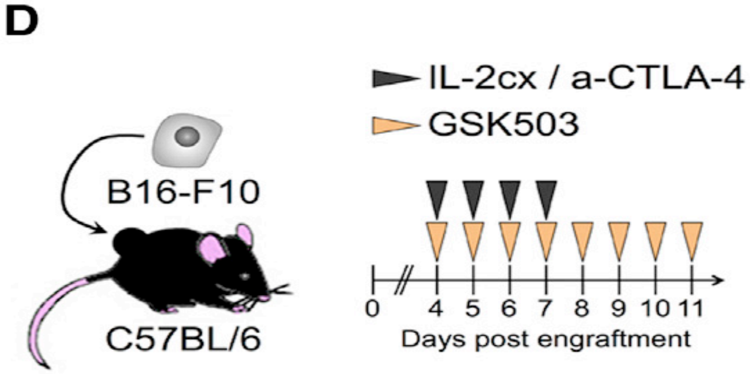


GSK503 ± IL-2cx or a-CTLA4 exhibited greater antitumor activity than single agent treatment.

**Comments on immunotherapy**

During anti-CTLA-4 and IL-2 treatment in models of melanoma. resistance to immunotherapy was due to increased Ezh2 activity that was dependent on T cells and TNF-a, resulting in melanoma dedifferentiation, loss of immunogenicity, and upregulation of the PD-1/PD-L1 axis. Inhibition of Ezh2 restored presentation of several dominant melanoma antigens while downregulating PD-L1 on melanoma cells. PD-1 expression on tumor-antigen specific and polyclonal melanoma-infiltrating CD8+ T cells decreased significantly, thus improving effector functions of these cells, including IFN-g production and cytotoxicity. Upon Ezh2 inactivation, effector CD8+ T cells preferentially accumulated intratumorally in a CXCL9/CXCL10-CXCR3-dependent fashion. Ezh2 inactivation locks melanoma cells in an immunogenic state and facilitates efficient CD8+ T cell-mediated anti-tumor responses during immunotherapy. These data suggest that subtoxic doses of EZH2 inhibitors might suffice to prevent cancer immune resistance during immunotherapy. Hence, targeting of EZH2 is an attractive strategy to combine with cancer immunotherapy.

## 19.2 Inhibition of EZH2 and DNA methylation enhancement of immunotherapy

Peng D et al., Epigenetic silencing of TH1-type chemokines shapes tumour immunity and immunotherapy. Nature. 2015;527:249-253.

EZH2-mediated histone H3 lysine 27 trimethylation (H3K27me3) and DNA methylation repress the tumour production of T helper 1 (TH1)-type chemokines CXCL9 and CXCL10, and subsequently determine effector T-cell trafficking to the tumour microenvironment. Treatment with epigenetic modulators (5AZA-CdR and DZNep) removes the repression and increases effector T-cell tumour infiltration, slows down tumour progression, and improves the therapeutic efficacy of programmed death-ligand 1 (PD-L1).

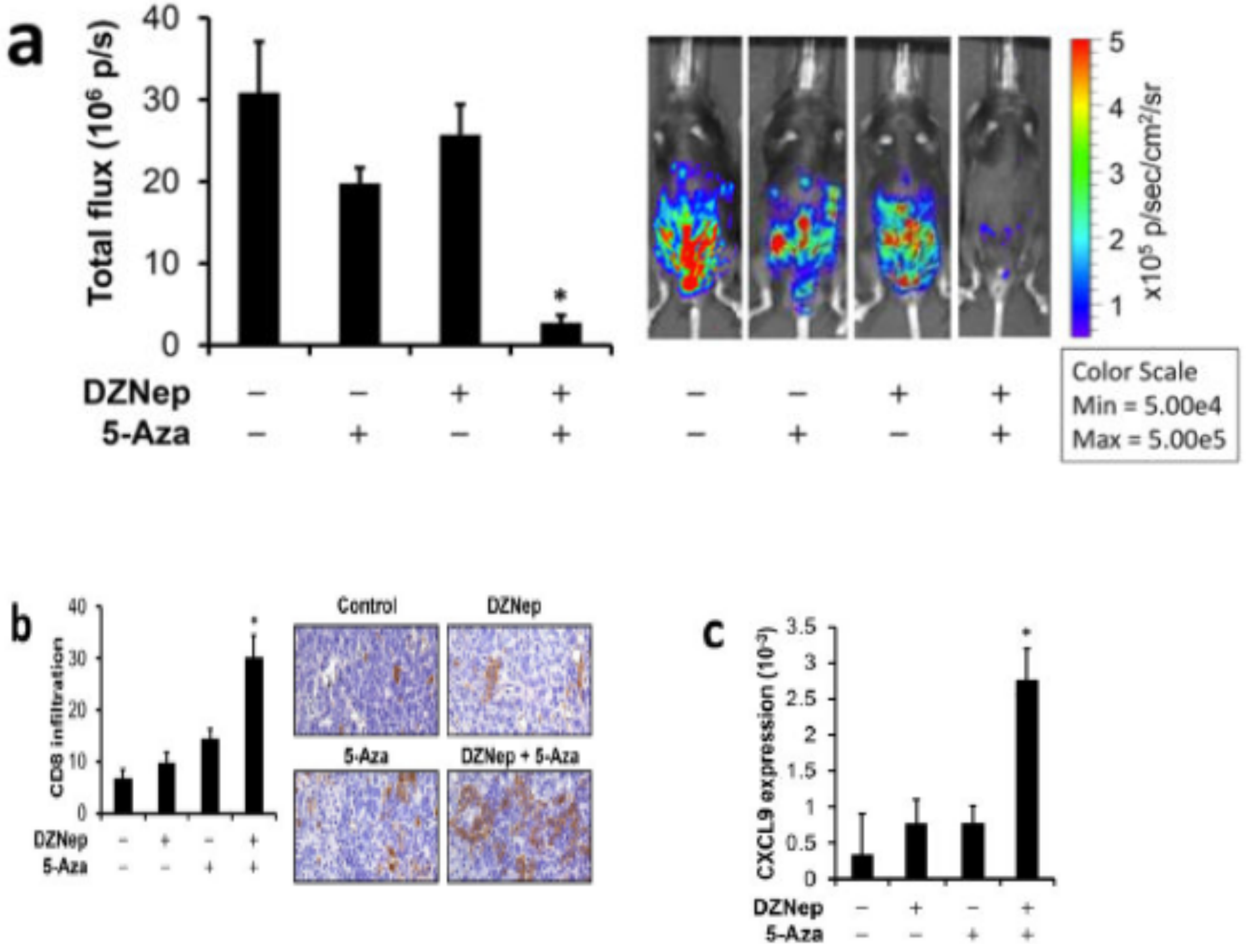


**Figure 1. Epigenetic reprogramming alters immunotherapy**

**a–c,** Effects of DZNep and 5-AZA-dC on ID8 mouse ovarian cancer progression. The ID8 tumor bearing mice (C57BL/6) were treated with DZNep and 5-AZA-dC. **(a)** Tumor growth was recorded by Bioluminescence imaging. The representative images and tumor volume at day 24 are shown. Day 0: tumor inoculation. **(b)** Tumor-infiltrating CD8+ T cells were quantified by immunohistochemistry staining (IHC). **(c)** Tumor CXCL9 mRNA was quantified by real-time PCR.

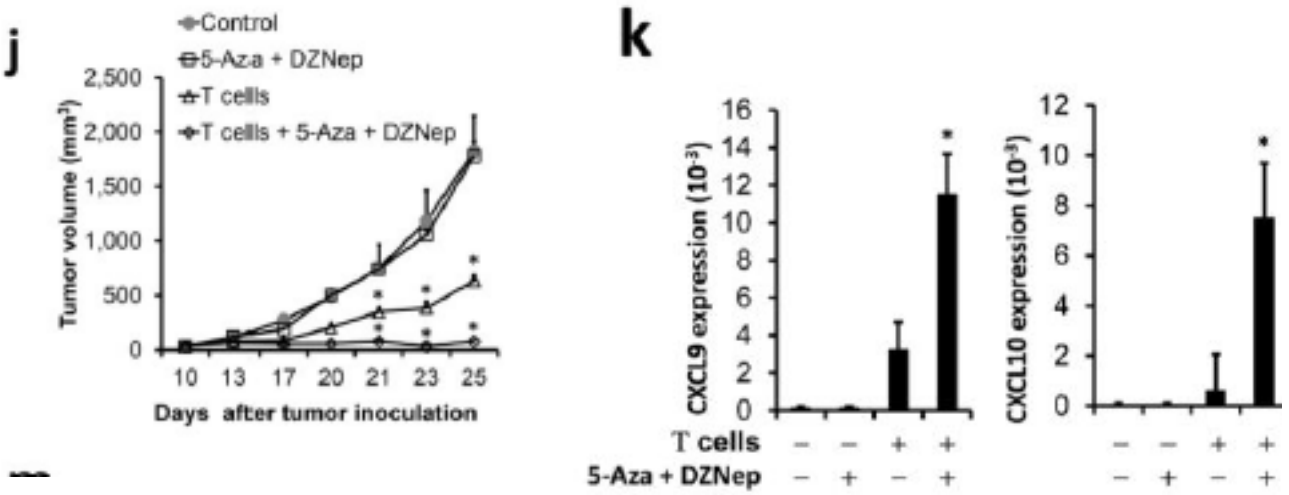


**Figure 1: j, k,** Effects of DZNep and 5-AZA dC on T cell immunotherapy. Autologous human tumor-specific human CD8+ T cells were transfused into ovarian cancer-bearing NOD-scid IL2Rγc null (NSG) mice with or without DZNep and 5-AZA-dC treatment. The mice were treated with anti-CXCR3. Tumor volume was monitored (**j**). Th1-type chemokine expression was quantified by real-time PCR (**k**). (mean/SEM, n = 5 per group, * $P < 0.05$ Mann-Whitney Test).

DZNep in combination with IFNγ increased remarkably the expression of CXCL9 and CXCL10 in human primary ovarian cancer cells (b). DZNep reduced the levels of EZH2 and H3K27me3 (f).

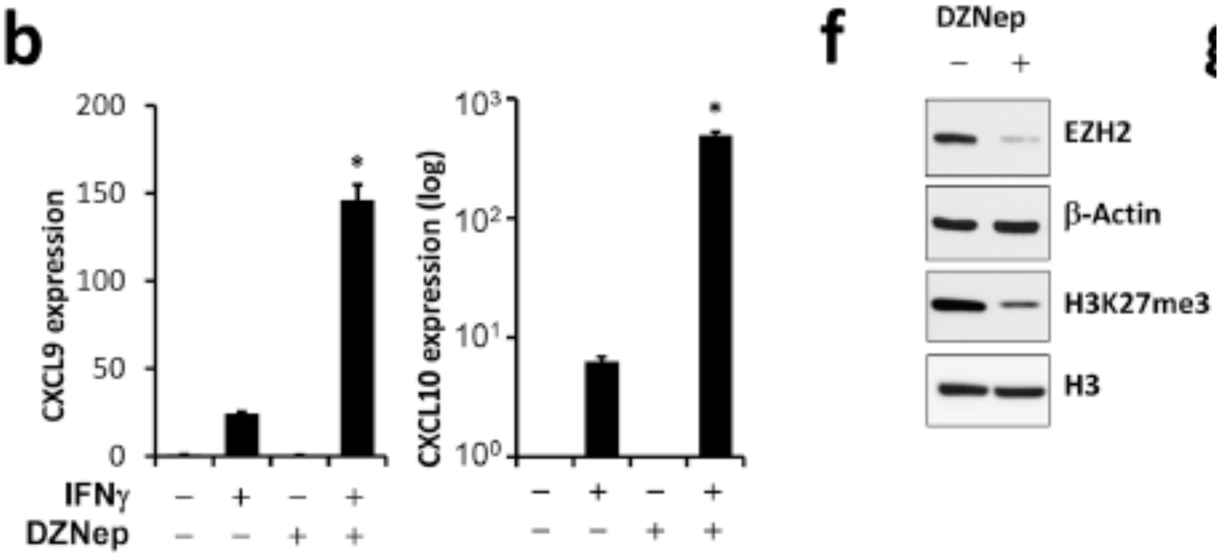


**Figure 2: b** Effects of DZNep on ovarian cancer Th1-type chemokine expression. Human primary ovarian cancer cells were treated for 24 (b) hours with DZNep in the presence of IFNγ. CXCL9 and CXCL10 expression were quantified by real-time PCR (b). **f**, Effects of DZNep on the expression of EZH2 and H3K27me3. Primary ovarian cancer cells were treated with or without DZNep for 24 hours. The levels of EZH2, H3K27me3 were detected by Western blotting.

**Comments on immunotherapy**

EZH2-mediated histone H3 lysine 27 trimethylation (H3K27me3) and DNA methyltransferase 1 (DNMT1)-mediated DNA methylation repress the tumour production of T helper 1 (TH1)-type chemokines CXCL9 and CXCL10, and subsequently determine effector T-cell trafficking to the tumour microenvironment. Treatment with epigenetic modulators (DZNep and 5AZA-dC) removes the repression and increases effector T-cell tumour infiltration, slows down tumour progression, and improves the therapeutic efficacy of programmed death-ligand 1 (PD-L1) checkpoint blockade and adoptive T-cell transfusion in tumour-bearing mice. Moreover, tumour EZH2 and DNMT1 are negatively associated with tumour-infiltrating CD8+ T cells and patient outcome. Epigenetic silencing of TH1-type chemokines is a novel immune-evasion mechanism of tumours. Selective epigenetic reprogramming alters the T-cell landscape in cancer and may enhance the clinical efficacy of cancer therapy.

**19.3. Inhibition of EZH2 enhances cancer-testis antigen expression**

Rao M et al., Inhibition of histone lysine methylation enhances cancer-testis antigen expression in lung cancer cells: implications for adoptive immunotherapy of cancer. Cancer Res. 2011; 71: 4192-4204.

Lung cancer cells were cultured for 72 hours in normal media with or without DZNep (0.5 or 5 mmol/L) followed by analysis after 24 hours. Immunoblot experiments showed that DZNep mediated dose-dependent depletion of KMT6, EED, and SUZ-12 with concomitant reduction in global H3K27Me3 levels in H841 lung cancer cells (Fig. 3A)

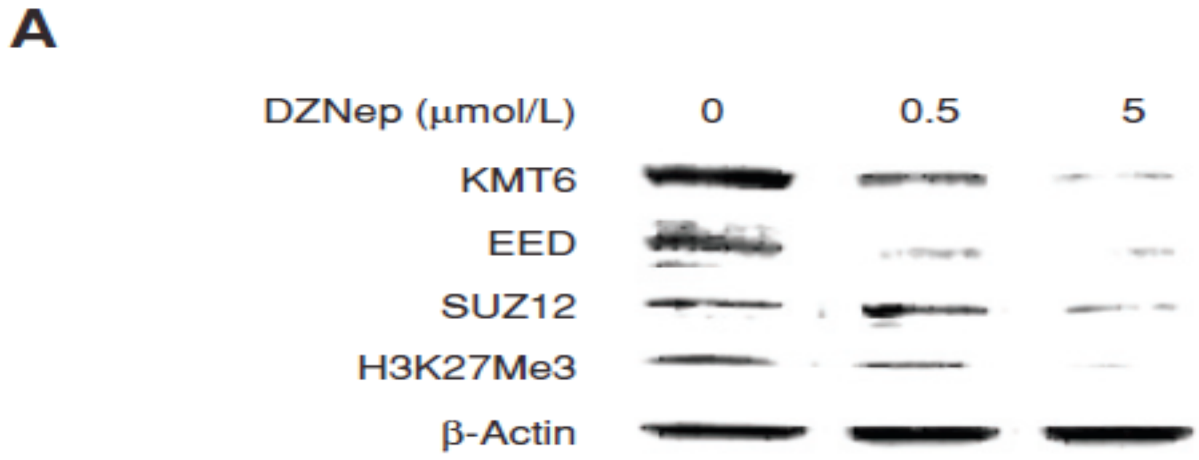


**Figure 3A** .Immunoblot analysis showing dose-dependent depletion of KMT6, EED, and SUZ12 and reduced global H3K27Me3 levels in H841 lung carcinoma cells mediated by DZNep. The cells were treated with DZNep for 72 hr followed by immunoblot analysis at 24 hr later.

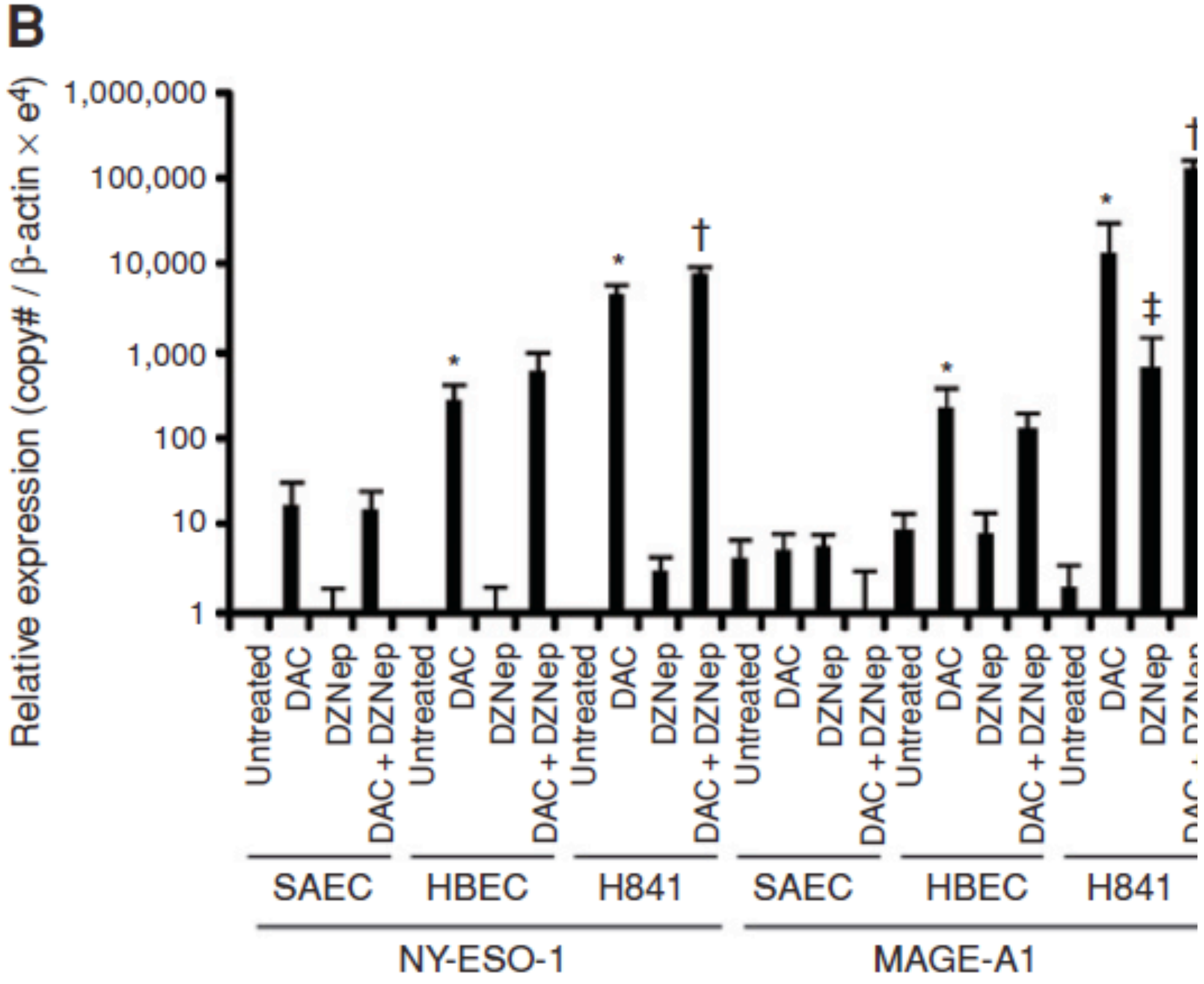


**Figure 3B.** qRT-PCR analysis of NY-ESO-1, MAGE-A1, and MAGE-A3 expression in cultured cells following DAC (5-AZA-CdR), DZNep, or DAC–DZNep treatment. The expression of NY-ESO-1 and MAGE-A1 was significantly greater in the DAC-DZNep treated H841 lung cancer cells as compared to the DAC treated cells.

Immunofluorescence experiments confirmed that DZNep enhanced DAC-mediated expression of NY-ESO-1, MAGE-A1, and MAGE-A3 in H841 lung cancer cells (**Fig. 3C**).

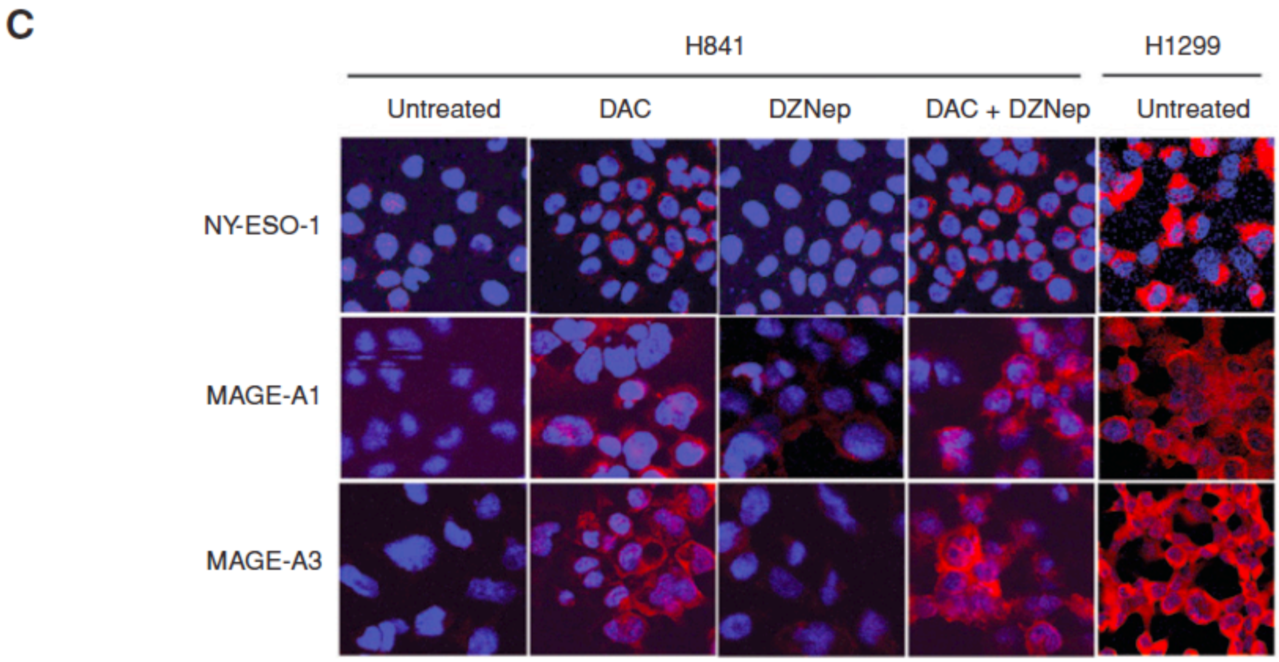


**Figure 3C**. Immunofluorescence analysis of NY-ESO-1, MAGE-A1, and MAGE-A3 expression in H841 lung cancer cells following DAC, DZNep, or DAC– DZNep exposure. H1299 cells were used as a positive control.

The magnitude of DAC–DZNep–mediated CT-X gene induction in lung cancer cells approximated or exceeded that observed following sequential DAC–DP or DAC–TSA treatment; addition of TSA or DP did not consistently improve CT-X gene activation mediated by low-dose DAC–DZNep (**Supplementary Table S5**). HDAC inhibitors: trichostatin A (TSA); romidopsin, depsipeptide (DP).

**Supplementary Table 5: Quantitative RT-PCR analysis of CT-X gene expression in lung cancer cells following exposure to DAC, DZNep, DP, TSA, or various combinations of these agents (copy # / β-actin x $e^4$).**

| | NY-ESO-1 | | | MAGE-A1 | | | MAGE-A3 | | | MAGE-A12 | | |
|---|---|---|---|---|---|---|---|---|---|---|---|---|
| **Treatment** | **A549** | **Calu-6** | **H841** | **A549** | **Calu-6** | **H841** | **A549** | **Calu-6** | **H841** | **A549** | **Calu-6** | **H841** |
| Untreated | 2 | 1 | 6 | 16 | 0 | 1 | 83513 | 962764 | 6 | 642 | 66780 | 7 |
| DAC-0.1µM | 5680 | 564 | 1225 | 12868 | 57 | 8072 | 399462 | 1198200 | 1790 | 8370 | 69819 | 3402 |
| DZNep 0.5 µM | 5 | 2 | 26 | 166 | 1 | 1014 | 197244 | 1454162 | 192 | 855 | 81888 | 344 |
| TSA-300nM | 20 | 5 | 12 | 842 | 2 | 1 | 98762 | 349955 | 3 | 2774 | 62752 | 9 |
| DP-25 ng/ml | 12 | 2 | 11 | 104 | 1 | 2 | 92983 | 891863 | 7 | 1223 | 52139 | 10 |
| DAC+DZNep | 9558 | 748 | 2506 | 51792 | 121 | 54578 | 703627 | 1397798 | 8069 | 19092 | 59975 | 11978 |
| DAC+DP | 1285 | 450 | 1715 | 20299 | 97 | 12230 | 542170 | 1078204 | 3378 | 13742 | 50756 | 7090 |
| DAC+TSA | 3449 | 597 | 1840 | 54403 | 408 | 15150 | 609365 | 472167 | 3124 | 20639 | 44327 | 6773 |
| TSA+DZNep | 7 | 4 | 30 | 1404 | 3 | 1437 | 140505 | 1118143 | 177 | 1621 | 73682 | 448 |
| DP+DZNep | 4 | 1 | 28 | 230 | 0 | 1053 | 128406 | 1130328 | 174 | 1070 | 89789 | 333 |
| DAC+DZNep+DP | 1781 | 977 | 3136 | 45401 | 56 | 73035 | 370008 | 1176560 | 9023 | 14563 | 72779 | 16662 |
| DAC+DZNep+TSA | 3393 | 703 | 3091 | 119226 | 315 | 71846 | 647016 | 793851 | 8817 | 27778 | 73414 | 15735 |

H841 cells transduced with HLA-A*0201 (H841-A2 cells) were exposed to NM, DAC, DZNep, or DAC–DZNep as previously described, and subsequently cocultured with TCR-engineered PBL (peripheral blood lymphocytes) recognizing NY-ESO-1 or MAGE-A3 in the context of HLA-A* 0201.

**Supplementary Table 6: Quantitative RT-PCR analysis of NY-ESO-1, MAGE-A3 and MAGE-A12 expression in lung cancer lines and SAEC targets used for cytokine and chromium release assays (copy # / β-actin x $e^4$). Data are representative of three separate experiments.**

| Gene | Treatment | H1299 | H1299-A2 | H2087 | H841 | H841-A2 | SAEC-A2 |
|---|---|---|---|---|---|---|---|
| NY-ESO-1 | Untreated | 503694 | 763661 | 8 | 1 | 2 | 1 |
| | DAC-0.1µM | ND | ND | 58825 | ND | 11940 | 35 |
| | DZNep-0.5 µM | ND | ND | 6 | ND | 16 | 0 |
| | DAC+DZNep | ND | ND | 106963 | ND | 18178 | 23 |
| MAGE-A3 | Untreated | 479535 | 672931 | 4553 | 1 | 11 | 0 |
| | DAC-0.1µM | ND | ND | 376081 | ND | 34723 | 5 |
| | DZNep-0.5 µM | ND | ND | 4948 | ND | 555 | 0 |
| | DAC+DZNep | ND | ND | 507113 | ND | 442235 | 4 |
| MAGE-A12 | Untreated | 185058 | 271515 | 70 | 8 | 8 | 4 |
| | DAC-0.1µM | ND | ND | 2715 | ND | 3071 | 7 |
| | DZNep-0.5 µM | ND | ND | 587 | ND | 51 | 2 |
| | DAC+DZNep | ND | ND | 11150 | ND | 11150 | 4 |

**Comments on immunotherapy**

Cancer–testis antigens (CTA), such as NY-ESO-1, MAGE-A1, and MAGE-A3, are immunogenic proteins. DNA methylation is the major epigenetic mechanism silencing CT-X genes in normal somatic cells. derepression of CT-X genes during malignant transformation cannot be attributed solely to global DNA demethylation. Chromatin architecture may contribute to repression/activation of CT-X genes. This study examined the feasibility of modulating histone lysine methylation as a strategy to enhance CT-X gene activation by DNA-demethylating agents. DZNep has been shown to deplete KMT6 (EZH2) and decrease global H3K27Me3 levels, a gene repression marker. DZNep alone did not activate NY-ESO-1, MAGE-A1, or MAGE-A3 expression in lung cancer cells, but significantly enhanced DAC-mediated induction of these CT-X genes. These observations that DZNep enhances DAC-mediated upregulation of NY-ESO-1 and MAGE-A family members, and markedly augments recognition and lysis of lung cancer cells by T cells specific for these CTAs, have direct translational implications about the development of gene-induction regimens for cancer immunotherapy. These data support further development of DZNep for cancer immunotherapy.

**19.4. Epigenetic modulation of immunotherapy**

Hong YK et al., Epigenetic modulation enhances immunotherapy for hepatocellular carcinoma. Cell Immunol 2019;336:66-74.

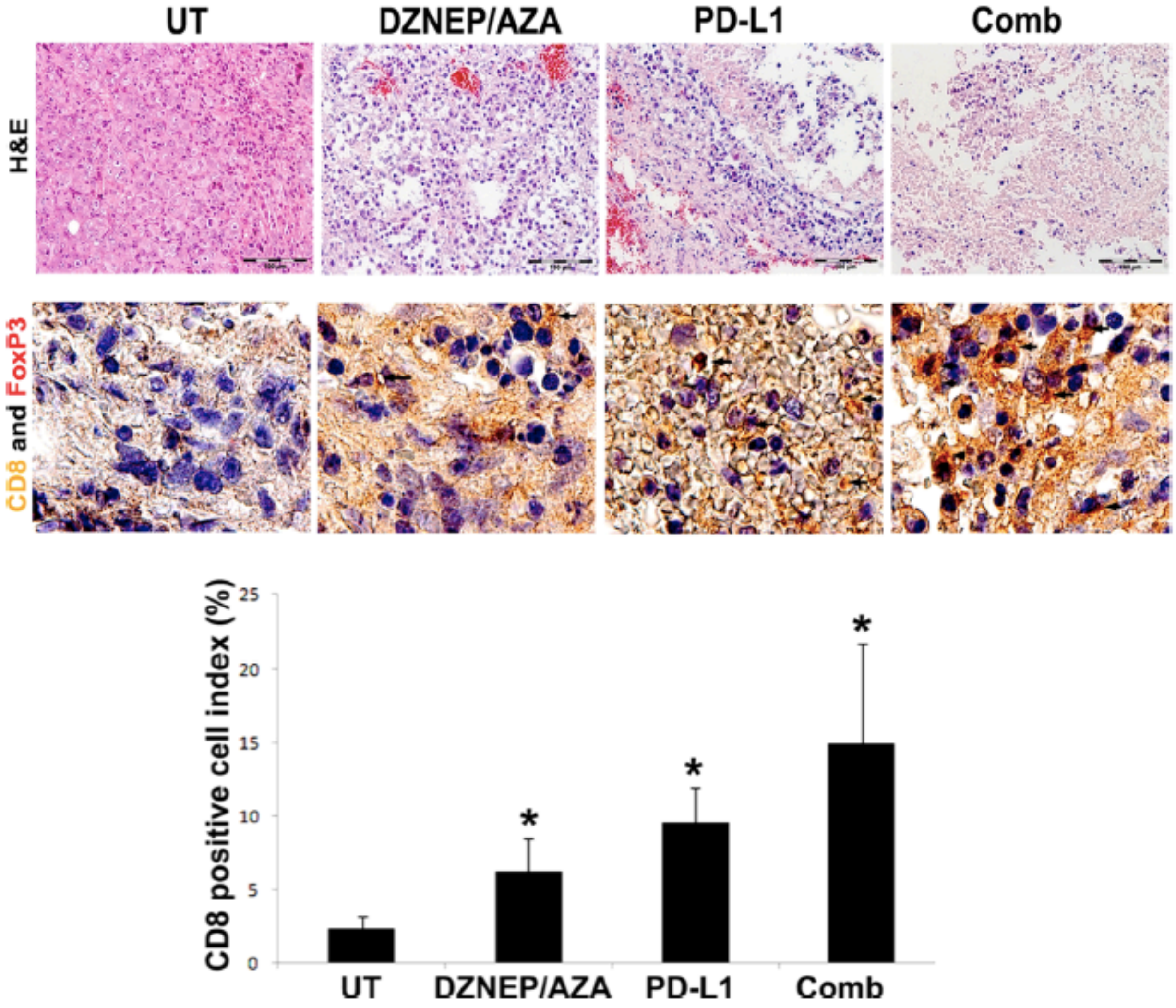


**Fig. 4.** Immunohistochemistry with hematoxylin and eosin demonstrating increased CD8+(brown)/FOXp3 (red) lymphocyte ratio with a combination treatment of epigenetic therapy (DZNEP/AZA) combined with PD-L1 inhibitor compared to control.

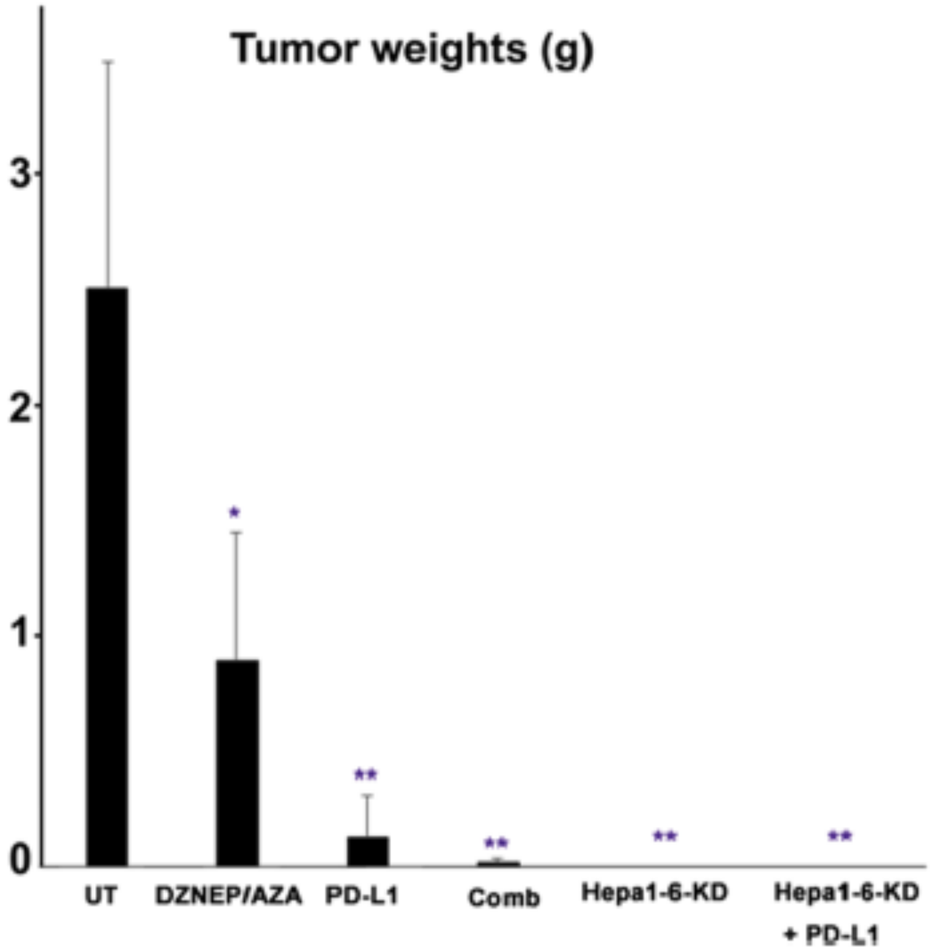


**Fig. 6.** To demonstrate epigenetic modulation by inhibition of EZH2 and DNMT1 in combination with immunotherapy using anti-PDL-1was responsible for the tumor regression, we performed flank injection of $5\times10^5$ Hepa1-6 cells in C56/LJ mice. After tumor inoculation was confirmed, we performed intraperitoneal injections of epigenetic drugs DZNep/AZA with or without anti-PDL-1 inhibitor three times per week (Monday, Wednesday, Friday). After 3 weeks of treatment, the animals were sacrificed and tumor weight measured. There was a moderate response to the epigenetic drug but significant response to PDL-1 alone and further response in the combination of the epigenetic drug with PDL-1 antibody.

**Comments on immunotherapy**

There was significantly increased trafficking of cytotoxic T-lymphocytes into the tumor for the combination treatment group compared to epigenetic or immunotherapy alone. The in vivo model also demonstrated statistically significant tumor regression in the combination treatment group (DZNep/AZA plus PD-L1 inhibitor) compared to epigenetic therapy or immunotherapy alone with untreated control. This study demonstrates that epigenetic modulation could be a novel potential strategy to augment immunotherapy for HCC by stimulating T cell trafficking into tumor microenvironment.

## 20. COMPARISON OF ANTICANCER ACTIVITY OF DZNep WITH INHIBITORS OF EZH2

Momparler RL, Côté S, Momparler LF, Marquez VE. Comparison of the antineoplastic action of 3-deazaneplanocin-A and inhibitors that target the catalytic site of EZH2 histone methyltransferase. Cancer Rep Rev 2020;3:1-4.

DZNep blocks the metabolism of methionine resulting in global inhibition of HMTs, including EZH2. It is of interest to compare the anticancer activity of DZNep with specific inhibitors that target the catalytic site of EZH2: GSK-126, GSK-343, CPI-1205, and tazemetostat (EPZ-6438). Several of these inhibitors are in clinical trial and exhibit promising activity against lymphomas. The preclinical in vivo data indicate that these specific EZH2 inhibitors require a longer duration of treatment than DZNep to exhibit significant anticancer activity. In order to understand more fully the relative anticancer activity of these inhibitors in the study cited above the in vitro anticancer activity of DZNep was compared to that of GSK-126, GSK-343, CPI-1205, and tazemetostat. DZNep exhibited much more potent anticancer activity against HL60 leukemic cells than any of these specific EZH2 inhibitors as determined by a growth inhibition assay (Fig. 1) and a colony assay (Fig. 2). Such a difference in anticancer potency may be explained in part by the limited penetration into cells of the specific EZH2 inhibitors due to their large complex molecular structure as compared to the smaller molecular size of DZNep. An additional explanation is that DZNep has several targets in the cell which contribute to its anticancer action: deregulation of methionine metabolism, proteosomal degradation of EZH2, and activation of miRNAs with TSG function. These observations indicate that DZNep also merits clinical investigation in patients with cancer.

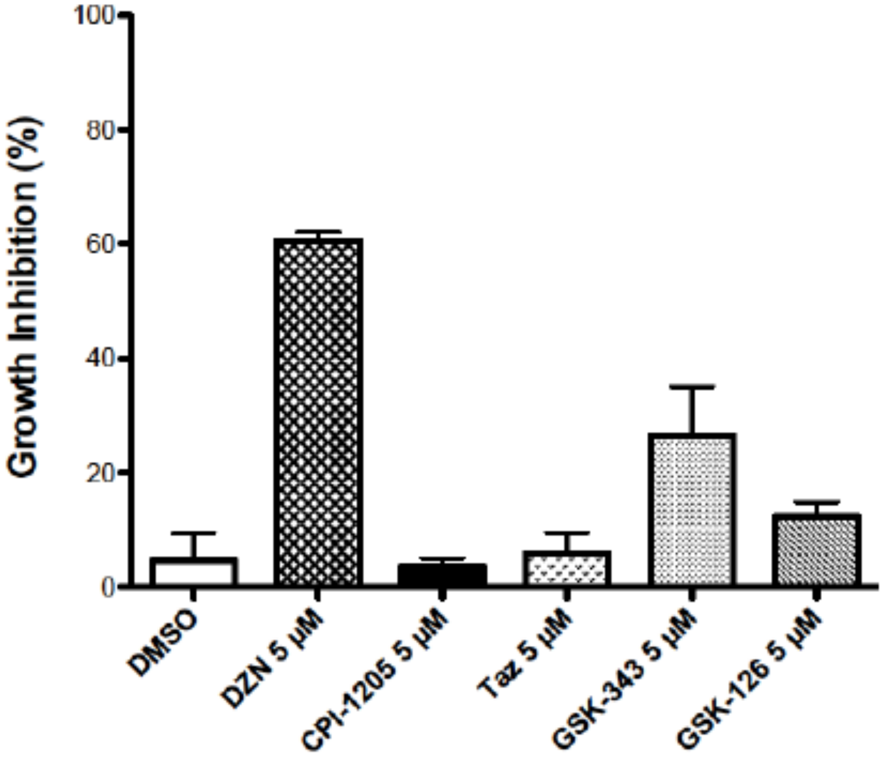


**Figure 1.** Effect of DZNep (DZN) and specific EZH2 inhibitors: CPI-1205, tazemetostat (TAZ), GSK343 and GSK126 on inhibition of the growth of HL-60 leukemic cells. The inhibitors were added at a concentration of 5 µM. A cell count was made at 48 h using Coulter electronic cell counter. The mean ± S.E. values for each of the inhibitors are indicated (n=3-4). DZNep was a more potent inhibitor than any of the specific EZH2 inhibitors ($p<0.05$)

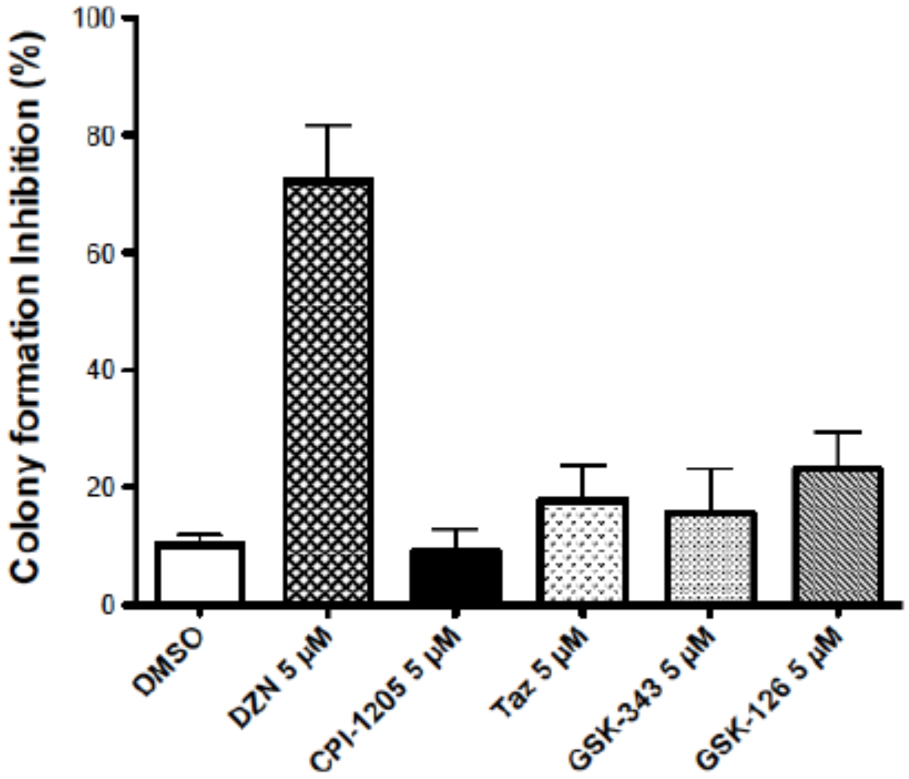


**Figure 2.** Effect of DZNep (DZN) and specific EZH2 inhibitors: CPI-1205, tazemetostat (TAZ), GSK343 and GSK126 on inhibition of colony formation in soft agar medium of HL-60 leukemic cells. The inhibitors were added at a concentration of 5 µM. A cell count was made at 48 h using Coulter electronic cell counter. An aliquot of 100 cells were placed in soft agar growth medium. A colony count was made on day 18-21. The mean ± S.E. values for each of the inhibitors are indicated (n=3-4). DZNep was a more potent inhibitor than any of the specific EZH2 inhibitors ($p<0.05$)

## 21. DISCUSSION

DZNep exhibits remarkable antineoplastic activity against many different types of cancer in both in vitro and in vivo models. In animal models DZNep displays very moderate toxicity which will facilitate the introduction of this nucleoside analogue for clinical investigation in patients with advanced cancer. The key mechanism of action of DZNep is the activation of genes that suppress malignancy by its inhibition and reduction in the level of EZH2, which removes the gene-silencing marker, H3K27me3, from tumor suppressor gene (TSG). This observation indicates the epigenetic alteration that silences gene expression by the methylation of H3K27 indicates that EZH2 plays an important role in the development of cancer. However, if the TSG contains a second epigenetic marker that silences gene expression, such as DNA methylation, the curative potential of the DZNep is limited. This means that the future of this epigenetic therapy should focus on the use of two inhibitors, one that targets EZH2, and the other that targets DNA methylation. This situation also exists for therapy with 5-AZA-CdR. The curative potential of this cytosine nucleoside analogue is limited because its removal of 5-methylcytosine from the target gene will not result in gene activation if it also contains the H3K72me3 silencing marker. The combination of 5-AZA-CdR and DZNep removes the "double lock" mechanism (Figure 3) and exhibits a remarkable synergistic antineoplastic activity against AML (Momparer et al., 2012) and lung cancer (Fernandes et al., 2017).

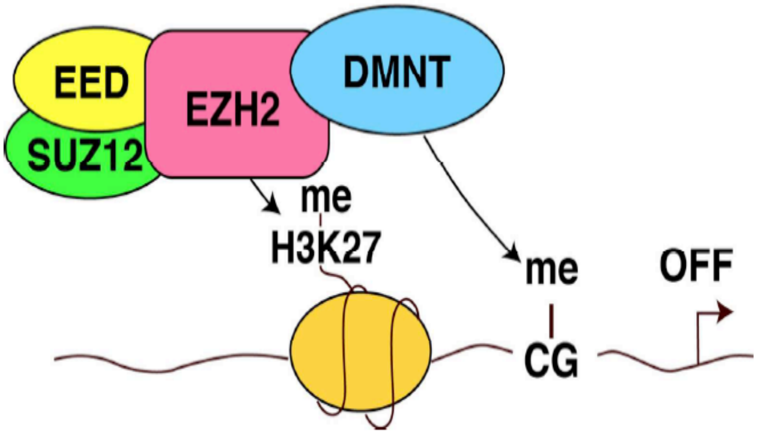


**Figure 3.** Epigenetic silencing of genes that block differentiation by the "double lock" mechanism of DNA methylation and H3K27me3. Since both these epigenetic marks are reversible, they are interesting targets for chemotherapeutic intervention (Momparler et al., 2015).

Curative therapy of cancer requires the complete eradication of cancer stem cells (CSCs) (Pattabiraman et al., 2014). In order to retain its self-renewal phenotype the CSC has to silence the genes that program differentiation. The target genes in normal stem cells that are silenced by EZH2 are 12-fold more likely to exhibit cancer cell-specific promoter DNA hypermethylation than non-target genes (Widschwendter et al., 2007). These observations support a stem cell origin for cancer in which reversible gene repression by EZH2 (H3K27me3)

is complemented by permanent silencing (DNA methylation), thereby locking the cell into a perpetual state of self-renewal. The role of EZH2 in promoting self-renewal and impeding normal stem cell differentiation suggests that similar events can occur in CSCs. DNA hypermethylation of CSCs may involve the recruitment of DNMTs to EZH2 target genes (Viré et al., 2006.; Schlesinger et al., 2007). Therefore, the genes that program terminal differentiation in CSCs can be silenced by two different epigenetic mechanisms: aberrant DNA methylation and H3K27 methylation by EZH2 (**Figure 3**). The interaction between these epigenetic events provides a good rationale to use a combination of inhibitors of DNA and histone methylation to target CSCs. In addition, 5-AZA-CdR in combination with DZNep can increase the expression of tumor-associated antigens and has the potential to enhance the effectiveness of immunotherapy of cancer (Schrump, 2012).

5-AZA-CdR (decitabine) is approved for the clinical treatment of leukemia and is under investigation in patients with solid tumors. In order to get approval for clinical investigation of DZNep in combination with 5-AZA-CdR, a phase I study must be performed on DZNep as a single agent in patients with cancer. Patients with advanced cancer that become resistant to standard chemotherapy have a very poor prognosis. For this reason, the Food & Drug Administration (FDA) has a “fast track” program to accelerate the introduction of promising anticancer agents in to clinical investigation in patients with terminal cancer. In addition, the FDA has reduced the requirements of in depth toxicology studies on new promising anticancer agents. This report contains sufficient data on DZNep to support an Investigational Drug Application (IND) for a phase I study on DZNep in patients with cancer.

Analysis of the antileukemic action of DZNep in mice can provide valuable information for the selection of the starting dose of this epigenetic agent for clinical investigation. Mice with L1210 leukemia treated with twice with DZNep at a dose of 2.5 mg/kg at an interval of 5 h (total dose 5 mg/kg) exhibited an increase in life span (ILS) of 22% (Momparler et al., 2012). Mice with HL-60 leukemia treated with DZNep at a dose of 1 mg/kg twice per week for 2 weeks (total dose 4 mg/kg) exhibited an estimated 20% ILS. These preclinical studies suggest that a single day treatment with a moderate dose of DZNep is as effective as a two-week treatment at a lower dose. Patients with relapsed leukemia that show resistance to conventional chemotherapy will be use as candidates for a phase I study. For the phase I study on DZNep we recommend a single day treatment at administered at a 6 h interval. The recommended starting dose of DZNep is 1 mg/kg per i.v. injection x 2 for a total dose of 2 mg/kg. Three patients will be treated at this dose of DZNep. For the next cohort of 3 patients the dose of DZNep will be increased to 2 mg/kg per injection x 2 for a total dose of 4 mg/kg. The dose level of DZNep for the subsequent 3 patients will then be increased to 4 mg/kg per injection x 2 for a total dose of 8 mg/kg. In order to evaluate the toxicity of the DZNep treatments, on days 0, 7 and 21 analysis of the blood chemistry, hematology and physical examination will be performed. If any serious adverse events occur at a specific dose level, additional treatments with DZNep will be performed at the previous lower dose level. Hematologic analysis will be used to evaluate the antileukemic action of DZNep, Reduction in tumor size will be used to evaluate the antitumor activity of DZNep. We think that it is not necessary to determine the maximal tolerated dose (MTD) of DZNep since our long-term

objective is to determine if DZNep in combination with 5-AZA-CdR (decitabine) exhibits a potent synergistic action against the leukemic cells. This synergistic antineoplastic action may occur at

moderate doses of DZNep. It is also possible that the MTD of DZNep when used in combination with 5-AZA-CdR may result in unacceptable toxicity. Determination of the optimal dose-schedule of this combination of epigenetic agents may require a step-by-step increase in the dose level of each agent until the MTD is reached. It is possible that curative therapy of cancer may occur at dose levels below the MTD of 5-AZA-CdR and DZNep.

It is of interest to compare the anticancer activity of DZNep with specific inhibitors that target the catalytic site of EZH2: GSK-126, GSK-343, CPI-1205, and tazemetostat (EPZ-6438). Several of these inhibitors are in clinical trial and exhibit promising activity against lymphomas. The preclinical data indicate that DZNep is a more important antineoplastic agent than these specific EZH2 inhibitors. The large complex molecular structure of the specific EZH2 inhibitors limits their distribution and penetration into malignant cells as compared to the simple molecular structure of DZNep. In addition, DZNep has several different molecular targets in cells, each which can contribute to its antineoplastic action. These observations indicate that DZNep merits clinical investigation with high priority which have the potential to prove its superior clinical effectiveness as compared to the specific EZH2 inhibitors.

## 21. REFERENCES


Baylin SB, Jones PA. A decade of exploring the cancer epigenome — biological and translational implications. Nature Rev Cancer 2011;11:726-734.

Benoit YD et al., Inhibitors of enhancer of zeste homolog 2 (EZH2) activate tumor-suppressor microRNAs in human cancer cells. Exp Cancer Res 2013;319:1463-1470.

Cheng LL et al., TP53 genomic status regulates sensitivity of gastric cancer cells to the histone methylation inhibitor 3-deazaneplanocin A (DZNep). Clin Cancer Res 2012;18:4201-4212.

Chiba T et al. 3-Deazaneplanocin A is a promising therapeutic agent for the eradication of tumor-initiating hepatocellular carcinoma cells. Int J Cancer. 2012;130:2557-2567.

Choi WJ, Jeong LS et al., Preparative and Stereoselective Synthesis of the Versatile Intermediate for Carbocyclic Nucleosides: Effects of the Bulky Protecting Groups to Enforce Facial Selectivity J. Org. Chem 2004;69:2634-2636.

Ciarapica R et al., Pharmacological inhibition of EZH2 as a promising differentiation therapy in embryonal RMS. BMC Cancer 2014, 14:139.

Crea F et al. Pharmacologic disruption of Polycomb Repressive Complex 2 inhibits tumorigenicity and tumor progression in prostate cancer. Mol Cancer. 2011;10:40.

Fiskus W et al. Combined epigenetic therapy with the histone methyltransferase EZH2 inhibitor 3-deazaneplanocin A and the histone deacetylase inhibitor panobinostat against Human AML cells. Blood 2009 114: 2733-2743.

Fiskus W et al. Superior efficacy of a combined epigenetic therapy against human mantle cell lymphoma cells. Clinical Cancer Res 2012;18:6227-6238.

Fujiwara T et al., 3-Deazaneplanocin A (DZNep), an inhibitor of S-adenosylmethionine-dependent methyltransferase, promotes erythroid differentiation. J Biol Chem 2014; 289 (12):8121-8134.

Gaudichon J et al., Deazaneplanocin a is a promising drug to kill multiple myeloma cells in their niche. PLoS One. 2014;9(9):e107009.

Girard N et al., 3-Deazaneplanocin A (DZNep), an inhibitor of the histone methyltransferase EZH2, induces apoptosis and reduces cell migration in chondrosarcoma cells. PLoS One. 2014;9(5):e98176.

Glazer RI et al., 3-Deazaneplanocin A: a new inhibitor of S-adenosylhomocysteine synthesis and its effects in human colon carcinoma cells. Biochem. Pharmacol. 1986a; 35:4523-4527.

Glazer RI, et al. 3-Deazaneplanocin: a new and potent inhibitor of S-adenosylhomocysteine hydrolase and its effects on human promyelocytic leukemia cell line HL-60. Biochem Biophys Res Commun 1986b;135:688-694.

Hibino S et al., Inhibitors of enhancer of zeste homolog 2 (EZH2) activate tumor-suppressor microRNAs in human cancer cells. Oncogenesis 2014;*3*:e104

Hong YK et al., Epigenetic modulation enhances immunotherapy for hepatocellular Carcinoma. Cell Immunol 2019;336:66-74.

Kalushkova A  et al., Polycomb target genes are silenced in multiple myeloma. PLoS One. 2010;5(7):e11483.

Kemp CD, Rao M, Xi S, Inchauste S, Mani H, Fetsch P, et al. Polycomb repressor complex-2 is a novel target for mesothelioma therapy. Clinical Cancer Res 2012;18:77-90.

Kikuchi J et al., Epigenetic therapy with 3-deazaneplanocin A, an inhibitor of the histone methyltransferase EZH2, inhibits growth of non-small cell lung cancer cells. Lung cancer. 2012;78 (2):138-143.

Kim KH, Roberts CWM. Targeting EZH2 in cancer. Nat Med 2016;22:128-134.

Li et al., The polycomb group protein EZH2 is a novel therapeutic target in tongue cancer. Oncotarget 2013; 4:2532-2549.

Lhussier E et al., Evaluation of the impact of S-adenosylmethionine-dependent methyltransferase inhibitor, 3-deazaneplanocin A, on tissue injury and cognitive function in mice. Oncotarget, 2018;9(29):20698-20708.

Miranda TB et al. DZNep is a global histone methylation inhibitor that reactivates developmental genes not silenced by DNA methylation. Mol Cancer 2009;8:1579-1588.

Momparler RL, Idaghdour Y, Marquez VE, Momparler LF. Synergistic antileukemic action of a combination of inhibitors of DNA methylation and histone methylation. Leuk Res. 2012;36:1049-54.

Momparler RL, Côté S, Momparler LF, et al. Epigenetic therapy of acute myeloid leukemia using 5-aza-2'-deoxycytidine (decitabine) in combination with inhibitors of histone methylation and deacetylation. Clinical Epigenetics 2014;6:19.

Momparler R & Côté S. Targeting of cancer stem cells by inhibitors of DNA and histone methylation. Expert Opin. Investig. Drugs 2015;24:8.

Momparler RL et al., Inhibition of DNA and Histone Methylation by 5-aza-2'-deoxycytidine (Decitabine) and 3-deazaneplanocin-A on antineoplastic action and gene expression in myeloid leukemic cells. Frontiers Oncol 2017;7:19.

Momparler RL, Côté S, Momparler LF, Marquez VE. Comparison of the antineoplastic action of 3-deazaneplanocin-A and inhibitors that target the catalytic site of EZH2 histone methyltransferase. Cancer Rep Rev 2020;3:1-4.

Nascimento ASF et al., Synergistic antineoplastic action of 5-aza-2'deoxycytidine (decitabine) in combination with different inhibitors of enhancer of 40. Peng D et al., Epigenetic silencing of TH1-type chemokines shapes tumour immunity and immunotherapy. Nature. 2015;527:249-253.

Pattabiraman DR, Weinberg RA. Tackling the cancer stem cells-what challenges do they pose? Nat Rev Drug Discov 2014;13:497-512.

Peer CJ et al. A rapid ultra HPLC-MS/MS method for the quantitation and pharmacokinetic analysis of 3-deazaneplanocin A in mice. J Chromatogr B Analyt Technol Biomed Life Sci. 2013;927:142-146.

Peng D et al., Epigenetic silencing of TH1-type chemokines shapes tumour immunity and immunotherapy. Nature. 2015;527:249-253.

Puppe J et al., BRCA1-deficient mammary tumor cells are dependent on EZH2 expression and sensitive to Polycomb Repressive Complex 2-inhibitor 3-deazaneplanocin A. Breast Cancer Res 2009;11:R63.

Rao M et al., Inhibition of histone lysine methylation enhances cancer-testis antigen expression in lung cancer cells: implications for adoptive immunotherapy of cancer. Cancer Res. 2011; 71: 4192-4204.

Schlesinger Y et al. Polycomb-mediated methylation on Lys27 of histone H3 pre-marks genes for de novo methylation. Nat Genet 2007; 39:232-236.

Schrump DS. Targeting epigenetic mediators of gene expression in thoracic malignancies. Biochim Biophys Acta. 2012;1819(7):836-845.

Sha M et al., DZNep inhibits the proliferation of colon cancer HCT116 cells by inducing senescence and apoptosis. Acta Pharmaceutica Sinica B. 2015;5:(3):188–193.

Shen L et al., 3-Deazaneplanocin A is a Promising Therapeutic Agent for Ovarian Cancer Cells. Asian Pac J Cancer Prev. 2013;14(5):2915-2918.

Shen J et al., Growth Inhibition Accompanied by MOB1 Upregulation in Human Acute Lymphoid Leukemia Cells by 3-Deazaneplanocin A. Biochem Genet (2015) 53:268–279.

Smits M et al., miR-101 is down-regulated in glioblastoma resulting in EZH2-induced proliferation, migration, and angiogenesis. Oncotarget 2010;1:710-720.

Sun F et al., Preclinical pharmacokinetic studies of 3-deazaneplanocin A, a potent epigenetic anticancer agent, and its human pharmacokinetic prediction using GastroPlus™. Euro J Pharmaceutical Sciences 2015;77:290–302.

Sun R et al., Overexpression of EZH2 is associated with the poor prognosis in osteosarcoma and function analysis indicates a therapeutic potential. Oncotarget. 2016;7(25):38333-38346.

Suvà M-L et al., EZH2 Is Essential for Glioblastoma Cancer Stem Cell Maintenance. Cancer Res 2009; 69:(24):9211–9218

Tan J, Yang X, Zhuang L, Jiang X, Chen W, Lee PL, et al. Pharmacologic disruption of Polycomb-repressive complex 2-mediated gene repression selectively induces apoptosis in cancer cells. Genes Dev. 2007;21:1050-1063.

Tseng CK, Marquez VE et al., Synthesis of 3-deazaneplanocin A, a powerful inhibitor of S-adenosylhomocysteine hydrolase with potent and selective in vitro and in vivo antiviral activities. J Med Chem. 1989;32:1442-1446.

Ueda K et al., Inhibition of histone methyltransferase EZH2 depletes leukemia stem cell of mixed lineage leukemia fusion leukemia through upregulation of p16. Cancer Sci 2014;105:512–519.

Viré E, Brenner C, Deplus R, et al. The polycomb group protein EZH2 directly controls DNA methylation. Nature 2006;439:871-874.

Wang C et al., EZH2 mediates epigenetic silencing of neuroblastoma suppressor genes CASZ1, CLU, RUNX3, and NGFR. Cancer Res2011;72:315–324.

Wee ZN et al., EZH2-mediated inactivation of IFN-γ-JAK-STAT1 signaling is an effective therapeutic target in MYC-driven prostate cancer. Cell Rep. 2014;8(1):204-216.

Widschwendter M et al. Epigenetic stem cell signature in cancer. Nat Genet 2007;39:157-158.

Xie Z et al., Determinants of sensitivity to DZNep induced apoptosis in multiple myeloma cells. PLoS ONE 6(6):e21583.

Yaginuma S et al., Studies on neplanocin A, new antitumor antibiotic. I. Producing organism, isolation and characterization. J Antibiot Tokyo 1981;34:359-366.

Zingg D et al., The histone methyltransferase Ezh2 controls mechanisms of adaptive resistance to tumor immunotherapy. Cell Reports 2017;20:854–867.

Zoabi M et al. PRAJA1 is a ubiquitin ligase for the polycomb repressive complex 2 proteins. Biochem Biophys Res Commun 2011; 408:393–398.

## 23. ACKNOWLEDGEMENTS

Jean-Jacques Mbwakongo, M.Sc., provided valuable assistance in the preparation of this report.
